# On Silylated Oxonium and Sulfonium Ions and Their Interaction with Weakly Coordinating Borate Anions

**DOI:** 10.1002/chem.201904403

**Published:** 2020-01-24

**Authors:** Kevin Bläsing, Rene Labbow, Dirk Michalik, Fabian Reiß, Axel Schulz, Alexander Villinger, Svenja Walker

**Affiliations:** ^1^ Anorganische Chemie, Institut für Chemie Universität Rostock A.-Einstein-Str. 3a 18059 Rostock Germany; ^2^ Materialdesign Leibniz-Institut für Katalyse A.-Einstein-Str. 29a 18059 Rostock Germany

**Keywords:** chalcogen, oxonium, silylium, sulfonium

## Abstract

Attempts have been made to prepare salts with the labile tris(trimethylsilyl)chalconium ions, [(Me_3_Si)_3_E]^+^ (E=O, S), by reacting [Me_3_Si‐H‐SiMe_3_][B(C_6_F_5_)_4_] and Me_3_Si[CB] (CB^−^=carborate=[CHB_11_H_5_Cl_6_]^−^, [CHB_11_Cl_11_]^−^) with Me_3_Si‐E‐SiMe_3_. In the reaction of Me_3_Si‐O‐SiMe_3_ with [Me_3_Si‐H‐SiMe_3_][B(C_6_F_5_)_4_], a ligand exchange was observed in the [Me_3_Si‐H‐SiMe_3_]^+^ cation leading to the surprising formation of the persilylated [(Me_3_Si)_2_(Me_2_(H)Si)O]^+^ oxonium ion in a formal [Me_2_(H)Si]^+^ instead of the desired [Me_3_Si]^+^ transfer reaction. In contrast, the expected homoleptic persilylated [(Me_3_Si)_3_S]^+^ ion was formed and isolated as [B(C_6_F_5_)_4_]^−^ and [CB]^−^ salt, when Me_3_Si‐S‐SiMe_3_ was treated with either [Me_3_Si‐H‐SiMe_3_][B(C_6_F_5_)_4_] or Me_3_Si[CB]. However, the addition of Me_3_Si[CB] to Me_3_Si‐O‐SiMe_3_ unexpectedly led to the release of Me_4_Si with simultaneous formation of a cyclic dioxonium dication of the type [Me_3_Si‐μO‐SiMe_2_]_2_[CB]_2_ in an anion‐mediated reaction. DFT studies on structure, bonding and thermodynamics of the [(Me_3_Si)_3_E]^+^ and [(Me_3_Si)_2_(Me_2_(H)Si)E]^+^ ion formation are presented as well as mechanistic investigations on the template‐driven transformation of the [(Me_3_Si)_3_E]^+^ ion into a cyclic dichalconium dication [Me_3_Si‐μE‐SiMe_2_]_2_^2+^.

## Graphical abstract

**Labile chalconium ions** of the type [(Me_3_Si)_3_E]^+^ (E=O, S) were generated by reacting [Me_3_Si‐H‐SiMe_3_][B(C_6_F_5_)_4_] and Me_3_Si[CB] (CB^−^=carborate=[CHB_11_H_5_Cl_6_]^−^, [CHB_11_Cl_11_]^−^) with Me_3_Si‐E‐SiMe_3_. A ligand exchange in case of the oxonium species, leading to the surprising formation of the persilylated [(Me_3_Si)_2_(Me_2_(H)Si)O]^+^ oxonium ion, was observed. With Me_3_Si[CB] as silylating reagent, a cyclic dioxonium dication of the type [Me_3_Si‐μO‐SiMe_2_]_2_[CB]_2_ was isolated in an anion‐mediated reaction.

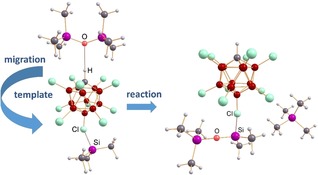

## Introduction

According to the IUPAC recommendations (Goldbook),^1^ onium compounds are those cations (e.g., H_4_E^+^, H_3_E^+^, and H_2_E^+^), which formally form by adding of a hydron (H^+^) to neutral binary hydrogen main group species (H_3_E, H_2_E, HE; with E=elements of groups 15–17). Starting from these parent compounds, derivatives can be generated by successive substitution of protons with monovalent groups.^1^, ^2^, ^3^ Classical hydrogen onium species of all three groups (H_4_E^+^ with E=N, P, As, and Sb;^4^, ^5^ H_3_E^+^ with E=O, S;^4^, ^6^, ^7^, ^8^ H_2_E^+^ with E=F, Cl)^9^, ^10^ were already reported. Since silylium ions, in particular, the [Me_3_Si]^+^, are often referred to as large protons,^11^, ^12^, ^13^, ^14^, ^15^, ^16^ they have also been used to synthesize onium ions to stabilize them kinetically. Ever since the pioneering silylium ion work by the groups of Lambert, Reed, Oestreich, and Müller,^17^, ^18^, ^19^, ^20^, ^21^ salts bearing homoleptic trimethylsilyl substituted cations ([T_4_E]^+^ with E=N,^22^ P, and As;^23^ [T_2_E]^+^ with E=F–I^12^ and T=Me_3_Si) have been in the focus of main group chemistry, however, there is hardly anything known about persilylated chalconium ions of the type [T_3_E]^+^.

**Scheme 1:**
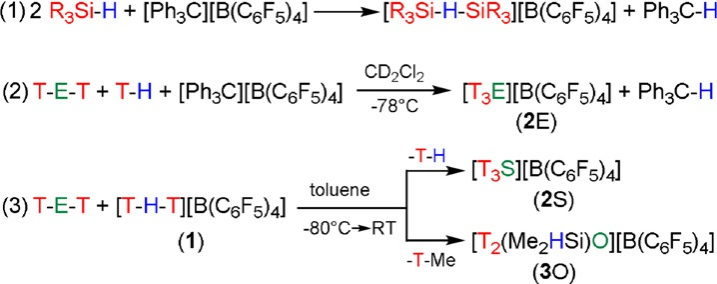
Synthesis of silylated chalconium salts with [B(C_6_F_5_)_4_]^−^ as counterion (E=O, S; T=Me_3_Si, R=alkyl).

In 1992, Kira et al. reported NMR data of some [R^1^R^2^R^3^SiOEt_2_]^+^ ions (R^1,2,3^=alkyl, aryl), for example, [Me_3_SiOEt_2_]^+^,^24^ while Olah and Prakash et al. already described transient trisilyloxonium ions including [T_3_O]^+^ by solution NMR techniques. In situ generated [T_3_O]^+^ was shown to be highly reactive, initiating polymerization of T‐O‐T, yielding different types of polysiloxanes.^25^ As shown in Scheme 1 [Eq. (2)], both [T_3_O]^+^ and [T_3_S]^+^ were generated in situ by treating Me_3_SiH in the presence of one equivalent of [Ph_3_C][B(C_6_F_5_)_4_] in CD_2_Cl_2_ at −78 °C,^26^ but again no isolation in the solid state was achieved. This prompted us to attempt the preparation, isolation and full characterization of salts containing [T_3_E]^+^ cations (E=O, S). Therefore, we started from trityl salts with weakly coordinating anions (wca)^27^ as counterions. For example [CHB_11_H_5_Cl_6_]^−^, [CHB_11_Cl_11_]^−^ and [B(C_6_F_5_)_4_]^−[28, 29]^ usually allow the isolation of highly reactive cations.^19^, ^20^, ^28^, ^29^, ^31^, ^32^, ^33^, ^34^, ^35^, ^36^, ^37^, ^38^, ^39^, ^40^, ^41^, ^42^, ^43^, ^44^, ^45^, ^46^, ^47^, ^48^, ^49^, ^50^ Here we report the straightforward synthesis and full characterization of salts containing the trimethylsilylsulfonium ion [T_3_S]^+^ and about the failure of synthesizing salts with the [T_3_O]^+^ ion that finally led to the isolation of unusual oxonium borate salts of the type [T_2_(Me_2_(H)Si)O][B(C_6_F_5_)_4_] and [T‐μO‐SiMe_2_]_2_[CB]_2_, respectively, depending on the weakly coordinating anion utilized. It should be noted that, as early as 1963, Corey and West^51^ used the Lewis acid assisted hydrogen/halogen exchange Bartlett‐Schneider‐Condon^52^ reaction for the first time in silicon chemistry. Thirty years later Lambert used a borate ([B(C_6_F_5_)_4_]^−^) as weakly coordinating anion in the reaction of Ph_3_C[B(C_6_F_5_)_4_] with hydridosilanes (R_3_SiH) and published a general synthetic approach to trialkylsilylium cations [R_3_Si]^+^ for the first time [cf. Scheme 1, Eq. (1)].^53^ However, (18 years later) Nava and Reed^44^ experimentally proved that the commonly used, supposed [R_3_Si][B(C_6_F_5_)_4_] salt (R=Et) does not exist at all, but always exists as a hydride‐bridged silane adduct [R_3_Si‐H‐SiR_3_]^+^ ion when [B(C_6_F_5_)_4_]^−^ is used as a counterion (and R=small substituent, for example, alkyl); an issue that is also addressed in detail in this report. The group of Knapp–Jenne reported the synthesis, spectroscopic and structural characterization of silylium cations [R_3_Si]^+^ (R=Me, Et, *i*Pr) stabilized by the perchlorinated weakly coordinating dianion [B_12_Cl_12_]^2−^.^49^

## Results and Discussion

### Synthesis of silylated chalconium ions and their reactivity towards borate and carborate anions

**Figure 1:**
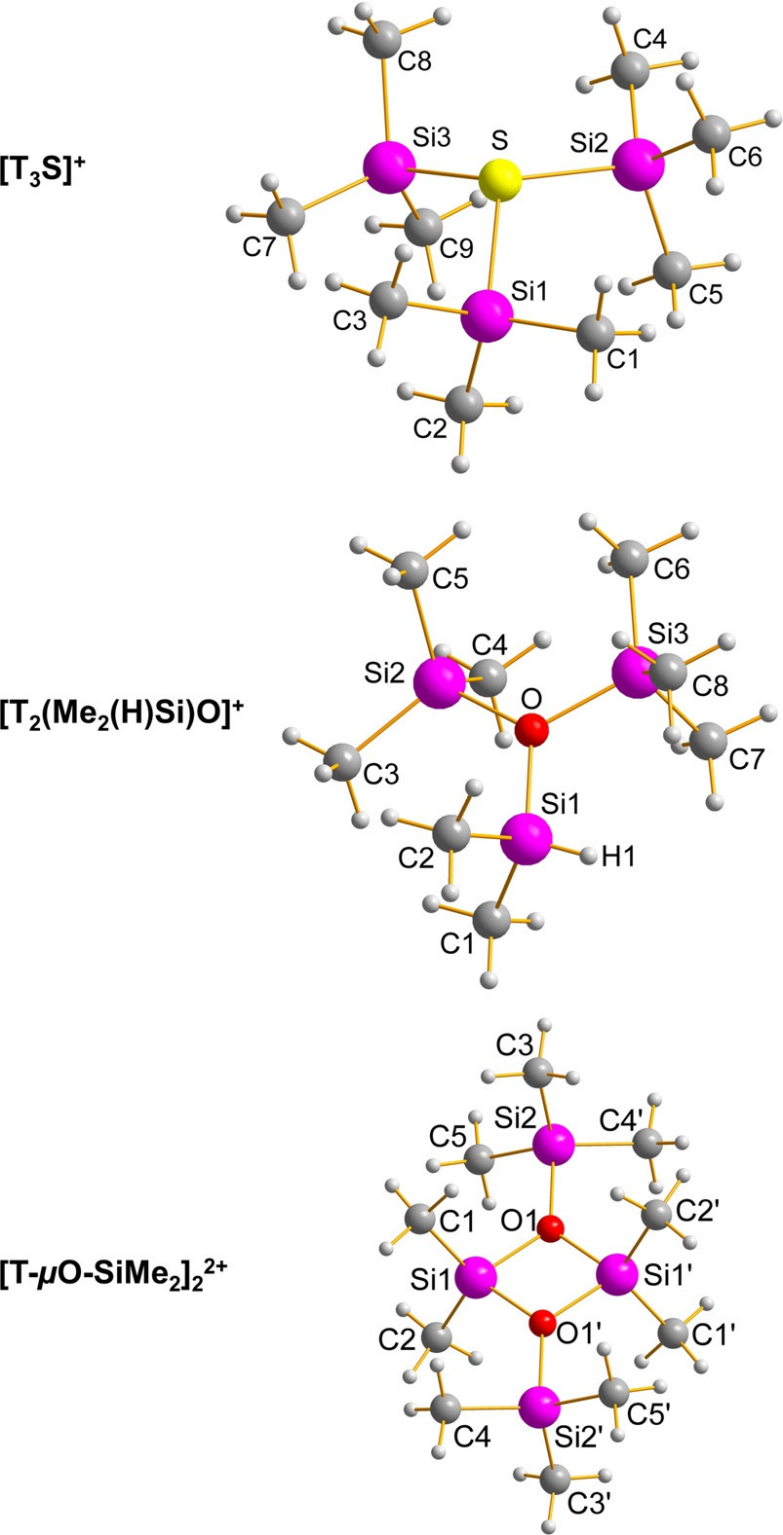
Molecular structure of [T_3_S][CHB_11_H_5_Cl_6_] (**4**S, top), [T_2_(Me_2_(H)Si)O][B(C_6_F_5_)_4_] (**3**O, middle), and [T‐μO‐SiMe_2_]_2_[CHB_11_Cl_11_]_2_ (**6**O, bottom) in the crystal. Anions and disorder omitted for clarity. Selected structural data are listed in Table 1. [T_3_S][B(C_6_F_5_)_4_] was also crystallized but is not shown here (see Supporting Information).

**[B(C_6_F_5_)_4_]^−^ salts**: We began this project with the synthesis of a suitable trimethylsilylium ion source, [T‐H‐T][B(C_6_F_5_)_4_] (T=Me_3_Si), which can be generated from the trityl salt and Me_3_SiH [Scheme 1, Eq. (1), R=Me]. However, in contrast to Olah and Prakash et al.,^26^ we have isolated this salt prior to the reaction with T‐E‐T, but not the T^+^‐salt, since [T‐H‐T][B(C_6_F_5_)_4_] always forms, when [B(C_6_F_5_)_4_]^−^ is the counterion.^44^ With the [T‐H‐T][B(C_6_F_5_)_4_] salt in hand, T‐E‐T was added to a suspension of [T‐H‐T][B(C_6_F_5_)_4_] in toluene at −80 °C [E=O, S; Scheme 1, Eq. (3)]. After warming to ambient temperatures, single crystals suitable for X‐ray structure elucidation were grown from this solution overnight. While in the case of T‐S‐T, the desired product [T_3_S][B(C_6_F_5_)_4_] (**2**S) could be isolated in good yields (46 %, Figure 1), the reaction with T‐O‐T afforded surprisingly [T_2_(Me_2_(H)Si)O][B(C_6_F_5_)_4_] [**3**O, Scheme 1, Eq. (3), Figure 1] in yields between 40–50 %. We would like to point out that this reaction was repeated many times and always only [T_2_(Me_2_(H)Si)O][B(C_6_F_5_)_4_], as proved by several X‐ray studies, could be isolated, never [T_3_O][B(C_6_F_5_)_4_]. However, note that always 11–12 % of a fluoronium salt [T‐F‐T][B(C_6_F_5_)_4_] co‐crystallized with **3**O, indicating slow decomposition of the [B(C_6_F_5_)_4_]^−^ anion because of the action of the strong Lewis acid [Me_3_Si]^+^. The structure and analytical data of [T‐F‐T][B(C_6_F_5_)_4_] as well as the degradation path of the starting material [T‐H‐T][B(C_6_F_5_)_4_], affording [T‐F‐T][B(C_6_F_5_)_4_], B(C_6_F_5_)_3_ and “C_6_F_4_”, which can be trapped with CS_2_, have already been reported earlier by our group.^12^, ^34^ A similar degradation of the [B(C_6_F_5_)_4_]^−^ ion has been reported before by Müller et al. in naphthyl‐based silylium ions.^31^ Moreover, we were able to prove the presence of [T_2_(Me_2_(H)Si)O]^+^ besides [T(H)(SiMe_2_H)O]^+^, and [(Me_3_Si)_2_OH]^+^ by CI^+^ mass spectroscopy experiments indicating a dynamic ligand exchange process in solution (see below).

**Scheme 2:**
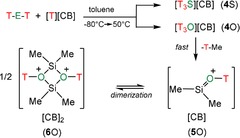
Synthesis of silylated chalconium salts with carborates as counterion (E=O, S; T=Me_3_Si; CB^−^=[CHB_11_H_5_Cl_6_]^−^, [CHB_11_Cl_11_]^−^).

**Carborate salts**: Obviously, Me_3_SiH, as well as the [B(C_6_F_5_)_4_]^−^ ion, are not innocent in the reaction mixture of [T‐H‐T][B(C_6_F_5_)_4_]/T‐E‐T. It was experimentally proven that the strong Lewis acid T^+^ initiates a ligand exchange as discussed before. So we had to change the silylating reagent, in particular, the cation that should allow the formation of a formally naked T^+^ counterion without any coordinated donor solvent molecule. For this reason, we synthesized T[CB] ([CB]^−^=[CHB_11_H_5_Cl_6_]^−^, [CHB_11_Cl_11_]^−^),^54^ bearing a formally naked T^+^ ion although strongly stabilized by a donor‐acceptor interaction with the carborate anion (vide infra, see section structure and bonding). We studied both carborate salts, T[CHB_11_H_5_Cl_6_] and T[CHB_11_Cl_11_], in reactions with T‐E‐T (Scheme 2). Indeed, the addition of T‐S‐T to a stirred suspension of [Me_3_Si][CB] in toluene and gentle warming (50 °C) led to a typical biphasic system, from which single crystals of [T_3_S][CB] suitable for X‐ray structure elucidation were grown upon slow cooling to ambient temperatures (yield 60–70 %). The analogous reaction with T‐O‐T, however, again resulted in a surprise, since not the desired [T_3_O][CB] salts could be isolated from toluene but colorless crystals of an unusual cyclic dioxonium salt of the type [T‐μO‐SiMe_2_]_2_[CB]_2_ (yield 52 %, Scheme 2 species **6**O) as evidenced by single‐crystal structure elucidation (Figure 1 bottom). It should be noted that this reaction is rather slow. For this reason, the [Me_3_Si][CB]/toluene suspension has been stirred for 5 minutes and treated with ultrasound prior to the addition of T‐O‐T. After adding of T‐O‐T, the two‐phase system was gently heated up to 70 °C for 30 min. Thermally, all [T_3_S]^+^ salts were stable up to over 150 °C, decomposing without melting at this temperature, while **3**O, as well as **6**O, decomposed already above 90 °C.

^29^Si NMR studies for all considered chalconium species are rather difficult or even completely hampered since often highly dynamic equilibria depending on solubility, temperature, solvent, side‐reactions (e.g., reactions with the solvent or anion) and concentrations were observed, even when pure crystals were dissolved. ^29^Si NMR resonances were observed for T_2_S between 12.9 and 15.3 ppm depending on the solvent (see ESI), which is shifted to lower field for [T_3_S]^+^ (31.7 (**2**S) −39.3 (**4**S) ppm) in accord with NMR studies of Olah and Prakash.^26^ As expected a high‐field shift along the series [T_3_S]^+^ (31.7, CD_2_Cl_2_), T_2_S (14.6, CD_2_Cl_2_) and TS^−^ (−0.9 ppm, thf) was detected. For the reaction of T‐O‐T with a formal “T^+^” salt, we carried out a series of different temperature variable experiments (see NMR experiments 1–9 in the Supporting Information file). When isolated crystals of **3**O were suspended in toluene, no resonance of the [T_2_(Me_2_(H)Si)O]^+^ ion was detected indicating a rather low solubility even at ambient temperatures. The same holds true for the reaction in benzene (NMR experiments 3–4, see Supporting Information). To increase the solubility, crystals of **3**O were suspended (partly dissolved) in a mixture of toluene/1,2‐dichlorobenzene at −20 and 25 °C (experiments 1.1 and 1.2), which gave rise to several resonances. On the basis of computations and coupling patterns, we assigned the signals at 31.5 (dsept, ^1^ *J*(^29^Si−^1^H)=230 Hz, ^2^ *J*(^29^Si−^1^H)=7.3 Hz) to the Me_2_(H)Si group and 53.8 ppm (multiplet) to both T groups in **3**O next to some unidentified (decomposition) species. However, at 25 °C an increasing amount of Me_4_Si and other side products could be detected (experiment 1.2). Moreover, at 25 °C, the coupling pattern of the signal at 53.8 ppm was observed to be a doublet of a decet (^3^ *J*(^29^Si−^1^H)=2.7 Hz and ^2^ *J*(^29^Si−^1^H)=6.8 Hz) as expected for a T_2_ group as in **3**O. When isolated crystals of **3**O were suspended in CD_2_Cl_2_ (experiment 2, see Supporting Information), a variety of signals were detected, which did not allow an unequivocal assignment. Finally, we tried to repeat the reaction of T‐O‐T with in situ generated [T‐H‐T][B(C_6_F_5_)_4_] [from Ph_3_C^+^/Me_3_Si‐H, see Scheme 1 Eq. (1)] as published by Olah and Prakash. For this reason, [Ph_3_C][B(C_6_F_5_)_4_] was treated with T‐O‐T and T‐H without further solvent and in CH_2_Cl_2_ (experiments 5–6, see Supporting Information). The ^29^Si spectra were recorded at different temperatures from −60 to 25 °C. In no case, we were able to verify the results by Olah and Prakash who reported a resonance at 51.1 ppm in CD_2_Cl_2_ at −70 °C for [T_3_O]^+^. In all of our experiments, we only observed the starting materials T‐O‐T as well as T‐H at low temperatures besides the fact that the solubility of any oxonium salt should be rather low. On increasing temperatures, the amount of T‐H decreases while the formation of Me_4_Si is dramatically increased due to decomposition and formation of **3** ^+^. To rule out the strong influence of the [B(C_6_F_5_)_4_]^−^ ion and the excess of T‐H from the [T‐H‐T]^+^ salt formation on the decomposition process, we also used the carborate salts but again no resonance for a [T_3_O]^+^ salt could be detected but only T‐O‐T (experiment 8) caused by a bad solubility of all considered carborate salts even at 25 °C. When CH_2_Cl_2_ was used to increase the solubility of the carborate salts (experiment 9), also no resonance for a [T_3_O]^+^ ion was detected but slow decomposition. In conclusion, we believe that it is not possible to generate larger amounts of [T_3_O]^+^ in solution due to a rather bad solubility, reaction with the solvent (e.g., chloride abstraction from CH_2_Cl_2_) and its tendency to decompose (see formation of **3**O and [T‐F‐T]^+^) as well as the transformation to [T‐μO‐SiMe_2_]_2_[CB]. The latter is only formed upon raising the temperature up to 70 °C. When crystals of **6**O were suspended in dmso, which is needed to dissolve at least a little amount of **6**O, four main resonances were detected [*δ*(^29^Si)=−17.4 (septet, Si(CH_3_)_2_), 42.6 (decet, Si(CH_3_)_3_, and 1.5 (septet, Si(CH_3_)_2_), and 9.0 (decet, Si(CH_3_)_3_,], which might indicate a monomer‐dimer equilibrium.

### X‐ray structure analysis

**[T_3_S][B(C_6_F_5_)_4_] and [T_3_S][CHB_11_H_5_Cl_6_]⋅toluene**: [T_3_S][B(C_6_F_5_)_4_] crystallizes in the monoclinic space group *P*2_1_/*c* and [T_3_S][CHB_11_H_5_Cl_6_]⋅toluene in *P*2_1_/*n*, both with four formula units per cell. In both salts, there are neither significant cation‐anion nor anion‐anion contacts. The observed molecular structure exhibits the expected slightly distorted trigonal pyramidal coordination environment around the sulfur atom with Si‐S‐Si angles between 107° and 111° (Table 1, Figure 1, cf. 108° in T‐S‐T),^55^ which is also supported by the sum of all Si‐S‐Si angles with 329.0° and 326.6°, respectively. The Si−S bond lengths of both salts (ranging between 2.24–2.31 Å, average 2.256 and 2.251 Å) are in good agreement with those observed in T‐S‐T (2.152(2) Å) and T−S^−^ (2.05–2.07,^56^, ^57^ cf. Σ*r*_cov_(Si−S)=2.19 Å).^58^ Interestingly, there are two slightly different Si−S bond lengths, which, according to computations, can be attributed to small cation‐anion interactions (see below, Table 1 and S42).

**Table 1:** Selected structural data from single‐crystal X‐ray studies (bond lengths in Å, angles in °, atom labels according to Figure 1 and Scheme 1, Scheme 2).^[a]^

	**2**S	**4**S	**3**O	**6**O
Si1‐E^[e]^	2.228(4)	2.2431(9)	1.763(1)	1.741(1)
Si2‐E	2.229(4)	2.2629(9)	1.77(1)	1.818(1)
Si3‐E	2.310(4)	2.2468(9)	1.78(2)	1.747(1)^[b]^
Ø(Si‐E)	2.256	2.251	1.77	1.769
Si1‐E‐Si2	110.7(2)	107.2(3)	122.4(1)	130.27(7)^[c]^
Si2‐E‐Si3	109.3(2)	109.8(4)	117.4(8)	96.73(6)^[d]^
Σ∡E	329.0	326.6	359.3	358.7

[a] [CHB_11_H_5_Cl_6_]^−^ salt. [b] Si1‐O1′. [c] Si1‐O1‐Si1′. [d] O1‐Si1‐O1′ 83.27(6); X‐ray data of the [B(C_6_F_5_)_4_]^−^ salt are given in the Supporting Information (Table S2). [e] For comparison, we have also crystallized [K[18]crown‐6][O‐SiMe_3_] and [K[18]crown‐6][S−SiMe_3_] (see Supporting Information, Table S1): *d*(Si−S)=2.025(1) and *d*(Si−O)=1.580(2) Å.

**[T_2_(Me_2_(H)Si)O][B(C_6_F_5_)_4_]**: For [T_2_(Me_2_(H)Si)O][B(C_6_F_5_)_4_] (**3**O) five different data sets were obtained from five different experiments. All measurements unequivocally proved the presence of **3**O⋅toluene, however, in all structures, the cation position was partially occupied (11–12 %) by the fluoronium cation [T‐F‐T]^+^. The fluorine atom of the [T‐F‐T]^+^ ion is always located on the H(Si) positon of the A/B‐Me_2_SiH groups of the oxonium ion [T_2_(Me_2_(H)Si)O]^+^ (see Figure S1). Therefore, a refinement without any restraints was not possible. Another indication for the presence of [T‐F‐T]^+^ (besides NMR data, *δ*[^19^F]=132 ppm in C_6_D_6_)^12^, ^34^ is that also the occupation of toluene, which is always present in conjunction with the oxonium cation, correlates with the occupation of the [T‐F‐T]^+^ ion. So if the slightly larger [T‐F‐T]^+^ ion is included, one of the SiMe_3_ groups is approximately located at the toluene position, leaving no accessible void for the toluene molecule (Figure S1). Also, the [T_2_(Me_2_(H)Si)O]^+^ ion is strongly disordered. **3**O⋅toluene crystallized in the monoclinic space group *P*2_1_/*c* with four formula units per cell. Besides weak van der Waals interactions between the ions, there are neither significant cation‐anion nor anion‐anion contacts. The most prominent structural feature is the planarity of the oxygen environment (Σ∡(Si‐O‐Si)=359.3°, Table 1) in contrast to the sulfonium ion structures (Figure 1). Due to the smaller space requirement, the O‐Si(H)Me_2_ bond is slightly shorter (1.76 Å) than the two O‐SiMe_3_ bonds (1.78 Å), with the H atom lying in the Si1‐O‐Si3 plane (dihedral angle ∡(H‐Si1‐O‐Si3)=0.6°).

**[T‐μO‐SiMe_2_]_2_[CHB_11_Cl_11_]_2_**: Compound [T‐μO‐SiMe_2_]_2_[CHB_11_Cl_11_]_2_ (**6**O) crystallizes in the triclinic space group *P* $\bar{1}$ as toluene solvate. While the toluene is coordinated by the one H atom of the [CHB_11_Cl_11_]^−^ in a η^6^ manner, the closest Si−Cl distances amounts to 3.9958(6) Å indicative for a weak van der Waals type interaction (Σ*r*_vdW_(Si−Cl)=3.85 Å).^59^ The molecular structure of the centrosymmetric cyclic cation (Figure 1 bottom) is characterized by a planar 4‐membered Si_2_O_2_ ring, featuring two tricoordinated oxonium atoms in a planar environment as expected for an oxonium cation (Σ∡O)=358.7°, Table 1). The Si−O bond lengths (1.741(1) and 1.747(1) Å) within the ring are slightly shorter compared to the terminal Si−O bonds (1.818(1) Å, cf. (Σ*r*_cov_(Si−O)=1.79 Å) and the Si1‐O‐Si1′ angles within the ring are significantly smaller with 96.73(6)° compared to 130.7(7)° and 131.6(7)° for both exocyclic Si‐O‐Si angles. These structural features are slightly different from those observed for T‐O‐T, exhibiting smaller Si−O bond lengths (1.631(6) Å) and a large Si‐O‐Si angle (142.2(3)°).^60^

### Thermodynamic and kinetic considerations of the chalconium ion formation depending on the counterion

**Scheme 3:**
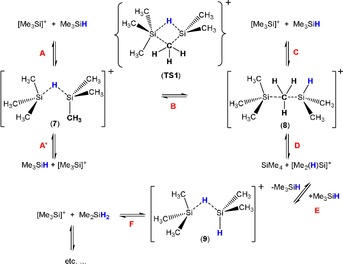
Silylium ion catalyzed scrambling that leads to a redistribution of the substituents at Me_3_SiH ([B(C_6_F_5_)_4_]^−^ not shown for clarity, TS=transition state, T=Me_3_Si; toluene adduct formation of the naked silylium ions not shown).

**Figure 2:**
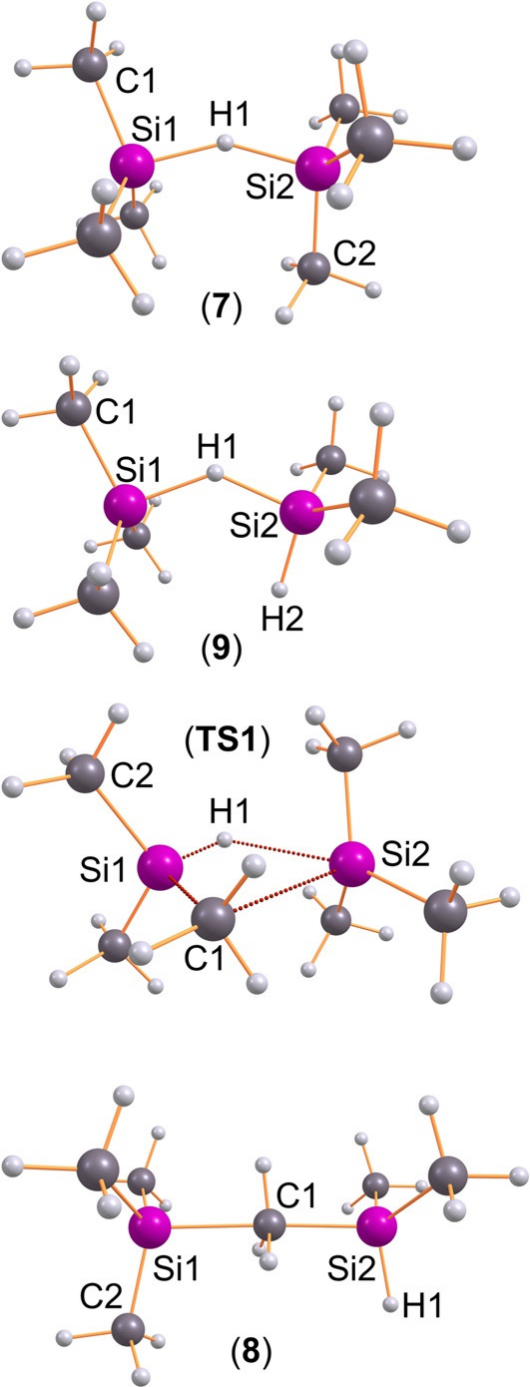
Computed structures that play an essential role in the silylium ion catalyzed scrambling process. Selected structural data are listed in Table S40.

As pointed out above, the reaction of T‐O‐T with [T‐H‐T][B(C_6_F_5_)_4_] always led only to the formation of [T_2_(Me_2_(H)Si)O]^+^ but not the desired [T_3_O]^+^ [B(C_6_F_5_)_4_]^−^ salt. Similarly, but conversely, in reactions with T‐S‐T, we were always able to isolate only [T_3_S][B(C_6_F_5_)_4_], but never [T_2_(Me_2_(H)Si)S] [B(C_6_F_5_)_4_]. This different reaction behavior led to a more detailed investigation of this problem by quantum mechanical calculations at the PBE1PBE/aug‐cc‐pVDZ level of theory including dispersion correction.^61^ As depicted in Scheme 3, there are two ways how the [Me_2_(H)Si]^+^ ion can be produced, which is necessary to form the [T_2_(Me_2_(H)Si)E]^+^ ion in the reaction with T‐E‐T. As a strong Lewis acid, naked T^+^ reacts with any possible neutral donor, which is the reason, why always bridged adducts such as [T‐H‐T]^+^ (**7**) and [T‐Me‐Si(H)Me_2_]^+^ (**8**; Figure 2) are formed in exergonic reactions with free Me_3_SiH in almost barrier‐free reactions (Table 2, Scheme 3, equilibria **A**, **C**, and **D**). Also conceivable would be the formation of T^+^⋅toluene adducts as starting materials, which is not shown in Scheme 3 for clarity but the Gibbs energies are also listed in Table 2. These data clearly suggest that toluene adduct formation plays an essential role in the equilibrium chemistry of silylium ion reactions. Starting from [T⋅toluene]^+^ ions the formation of **7** as well as **9** are true equilibria with Gibbs energies close to zero (−0.35 and 0.00 kcal mol^−1^), while the formation of **8**, featuring a bridging methyl unit, is endergonic (**C**: 12.46 kcal mol^−1^). Notably, **8** represents a low‐lying intermediate (stable minimum at the potential energy surface) that can give easily [Me_2_(H)Si⋅toluene]^+^ cations in an exergonic process (**D**: −8.56 kcal mol^−1^) or [T⋅toluene]^+^ ions (**C**). Besides, also an intramolecular process (**B**) via a 4‐membered cyclic transition state, which is associated with a barrier of 23.8 kcal mol^−1^, was found for the generation of **8**. Thermodynamically, the formation of **7**, as well as **9**, is favored over **8** by 12.8 and 8.6 kcal mol^−1^, respectively. However, these results do not explain the difference in the reaction behavior of T‐E‐T with **1** affording either **2**E or **3**E depending on the chalcogen (Scheme 1).

**Table 2:** Calculated Gibbs energies (Δ*G*°_298_ in kcal mol^−1^) for the ligand scrambling (Scheme 3) and chalconium ion formation (Scheme 4).

Reaction	**A**: →**7** ^[a]^	**B**: **7**→**8** ^[b]^	**C**: **8**→^[c]^	**D**: **8**→^[d]^
gas phase	−23.78	12.82	10.99	22.60
toluene^[f]^	−0.35	12.82	−12.46	−8.56
reaction	**E**: →**9**	**F**: **9**→^[e]^		
gas phase	−31.2	−19.26		
toluene^[f]^	0.00	4.20		
reaction	Equation (4)	Equation (5)	Equation (6)	Equation (7)
E=O, gas	−36.03	−49.94	–	–
toluene^[f]^	−12.58	−18.78	−12.22	−14.53
E=S, gas	−43.17	−54.05	–	–
toluene^[f]^	−19.71	−22.89	−19.36	−18.64

[a] Formation of **1** starting from T^+^ and Me_3_SiH. [b] Formation of **2** starting from **1** via **TS1**. [c] Formation of T^+^ and HSiMe_3_. [d] Formation of [T_2_(Me_2_(H)Si]^+^ and SiMe_4_. [e] Formation of Me_2_SiH_2_ and T^+^. [f] All non‐bridged cations were fully optimized as toluene adducts: [T⋅toluene]^+^, [Me_2_(H)Si⋅toluene]^+^. No stable toluene adducts were found for all bridged species.

**Scheme 4:**
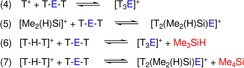
Calculated reactions for the formation of [T_3_E]^+^ and [T_2_(Me_2_(H)Si)E]^+^.

To understand the difference in product formation, one has to look more closely at the thermodynamic data of the formation of [T_3_E]^+^ and [T_2_(Me_2_(H)Si)E]^+^ (Scheme 4, Table 2). As expected, all reactions starting from the naked, toluene‐ and the Me_3_SiH‐coordinated cations are exergonic [Table 2, Eq. (4)–(7), Scheme 4]. However, for the reaction of naked cations with T‐E‐T, the reactions leading to the formation of [T_2_(Me_2_(H)Si)E]^+^ are more exergonic for both elements [E=O, S; cf. Eq. (4), (5)], but the Gibbs energy for reactions starting from the Me_3_SiH‐coordinated cations, [T‐H‐T]^+^ [Scheme 4, Eq. (6), (7)], with T‐E‐T changes the situation. While for E=S, the formation of [T_3_S]^+^ is thermodynamically preferred [Eq. (6): −19.36 vs. Eq. (7): −18.64 kcal mol^−1^], the situation is exactly the other way around for E=O, for which now the formation of [T_2_(Me_2_(H)Si)O]^+^ is thermodynamically favored [Eq. (6): −12.22 vs. Eq. (7): −14.53 kcal mol^−1^], in accord with our experimental observations. Additionally, the formation of either **2**S or **3**O, respectively, and its precipitation as [B(C_6_F_5_)_4_]^−^ salt superimposes the described exchange equilibria (Schemes 3, 4) leading to a new equilibrium adjustment. The different thermodynamic stability of **2**E versus **3**E for the chalcogens oxygen and sulfur is because of a significantly stronger Pauli repulsion in **2**O. This leads to a preference for species **3**O with reduced repulsion due to the smaller substituent (H versus Me, vide infra). For the significantly larger sulfur atom, this plays a subordinate role.

**Scheme 5:**
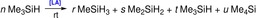
Lewis acid‐catalyzed scrambling process for Me_3_SiH, which occurs, when catalytic amounts of a Lewis acid (LA) are present.^22^

Formally, the transformation of a Me_3_Si group to a Me_2_(H)Si group represents a silylium ion catalyzed methyl/hydrogen exchange reaction that has been experimentally observed before in the reaction of NT_3_ with [T‐H‐T][B(C_6_F_5_)_4_] affording the unexpected [T_3_(Me_2_(H)Si)N][B(C_6_F_5_)_4_] but not the desired [T_4_N]^+^ salt.^22^ Such a Lewis acid catalyzed scrambling always occurs when an excess of silanes, such as Me_3_SiH, is present in the formation of silylium cations, which is always the case, when the [T‐H‐T][B(C_6_F_5_)_4_] salt is used in a silylium ion transfer reaction. For example, when Me_3_SiH is stirred at ambient temperatures in the presence of catalytic amounts of a Lewis acid (e.g., R_3_Si^+^, R=Me, Et, etc.), the whole series of alkyl silanes R_*x*_SiH_*y*_ (*x*=1–4, *y*=0–3) was observed as depicted in Scheme 5 for R=Me.^22^ Therefore, the formation of [T_2_(Me_2_(H)Si)O][B(C_6_F_5_)_4_] is not really surprising, since [Me_2_(H)Si]^+^/Me_2_SiH_2_ were also generated by this catalytic process in accord with our computation (Scheme 3, Table 1, equilibria **E** and **F**). As displayed in Scheme 3, both formation reactions of **9** are exergonic with −31.2 and −19.3 kcal mol^−1^ in the gas phase (0.0 and 4.2 kcal mol^−1^ in toluene, Table S8, S9), theoretically manifesting the thermodynamically possible formation of **9** in this dynamic equilibrium chemistry. Similar exchange reactions have been observed by Müller and co‐workers^21^, ^62^ and Oestreich et al.,^63^ who described substituent scrambling in the formation of the arene silylium cation [(Mes)_3_Si]^+^ or the ferrocene‐substituted species [*i*PrSi(Fc)_2_]^+^, respectively. Furthermore, Brookhart et al. described the transformation of Me_2_EtSiH with the transition metal complex Et_3_Si(H)_2_Ir(μ‐SiEt_2_)_2_Ir(H)_2_SiEt_3_ as catalyst in the presence of hydrogen and observed a substituent redistribution affording Et_2_MeSiH, Me_2_EtSiH, Me_3_SiH and Et_3_SiH.^64^ The structure of [Et_3_Si‐H‐SiEt_3_][B(C_6_F_5_)_4_] was described by Heinekey and co‐workers along with the observation of hydrogen release and the formation of Et_4_Si in benzene or toluene indicating substituent redistribution, too.^65^ Similar H‐silane activation mechanisms by B(C_6_F_5_)_3_ have been reported in literature.^66^, ^67^

### [CHB_11_Cl_11_]^−^ anion: [T‐μE‐SiMe_2_]_2_^2+^ versus [T_3_E]^+^ salt formation

**Figure 3:**
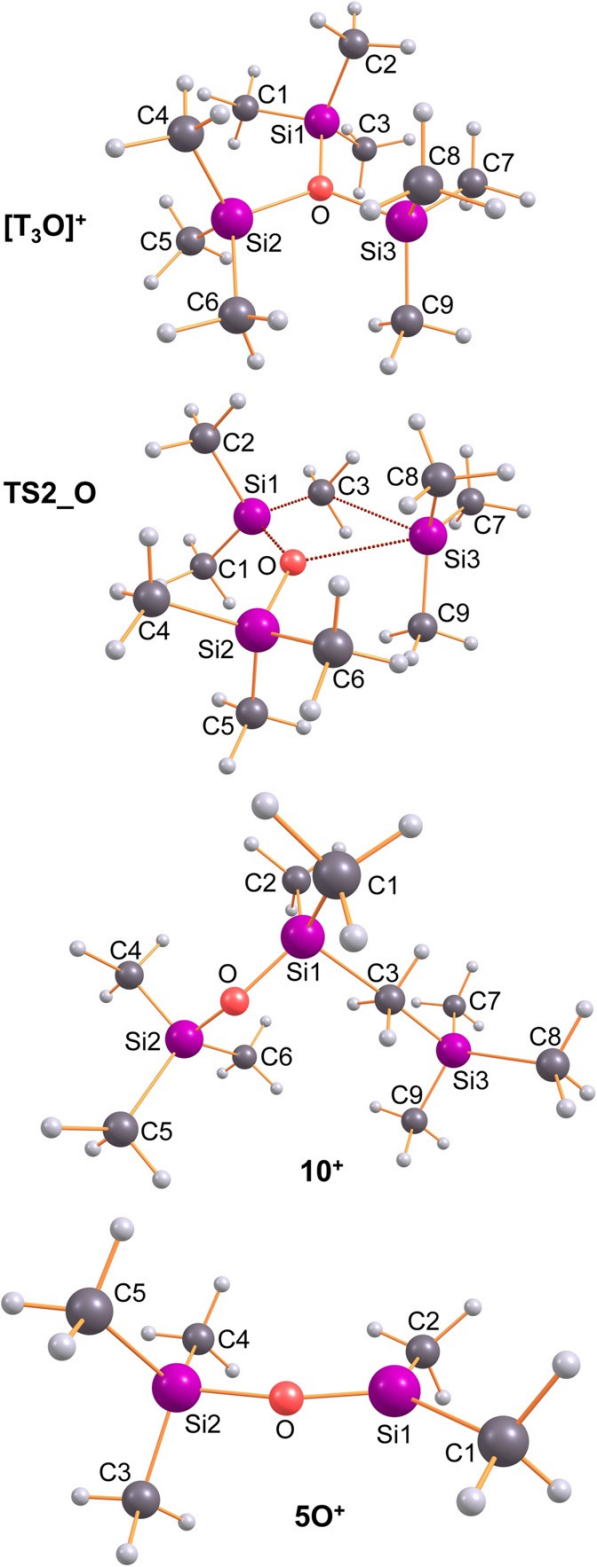
Computed structures that play an essential role in the formation of the monocation **5**O^+^ that dimerizes to give the observed dication **6**O^2+^ (Figure 1). Selected structural data are listed in Table S42.

**Scheme 6:**
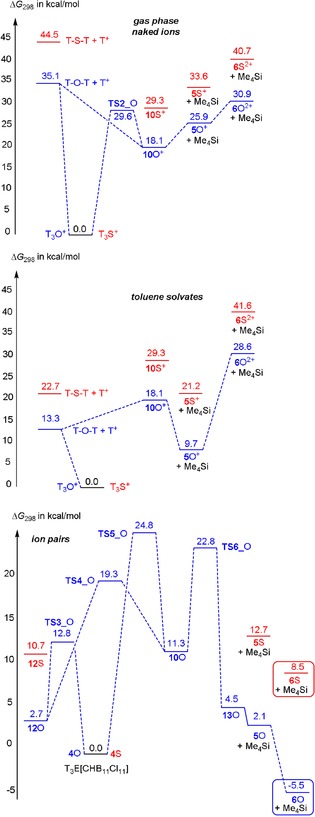
Calculated reaction profile for the formation of [T‐μE‐SiMe_2_]_2_^2+^ (**6**E^2+^). Top: naked ions in the gas phase (structures are shown in Figure 3), middle: toluene solvates, bottom: as ion pairs with the [CHB_11_Cl_11_]^−^ as the counterion (Structures are shown in Figures 4 and 5).

**Figure 4:**
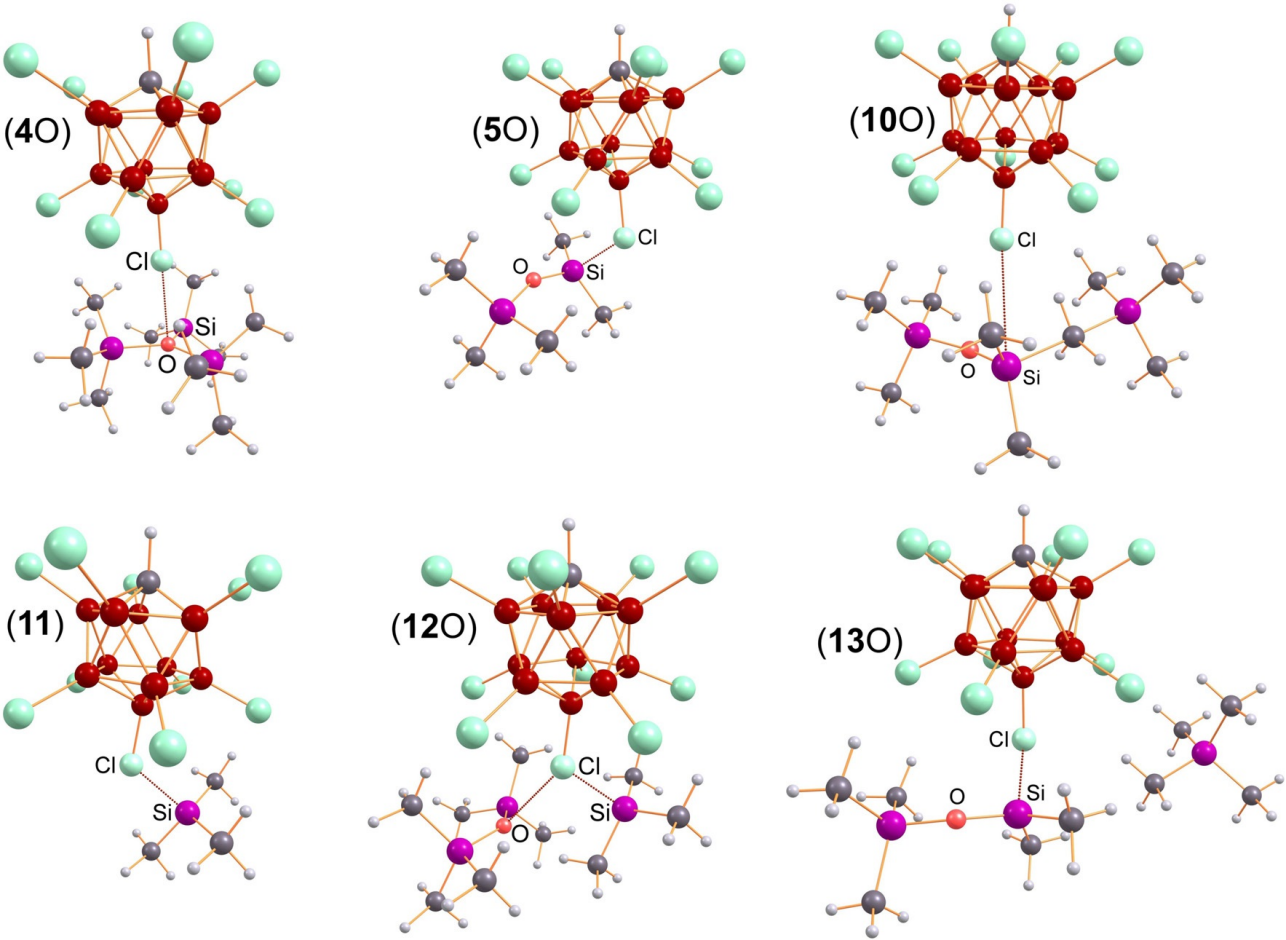
Computed structures that play an essential role in the formation of the monocation containing salt **5**O (=**5**O^+^[CHB_11_Cl_11_]^−^) that dimerizes to give the observed dication **6**O (=**6**O^2+^[CHB_11_Cl_11_]_2_^−^). Selected structural data are listed in Table S42, S43.

**Figure 5:**
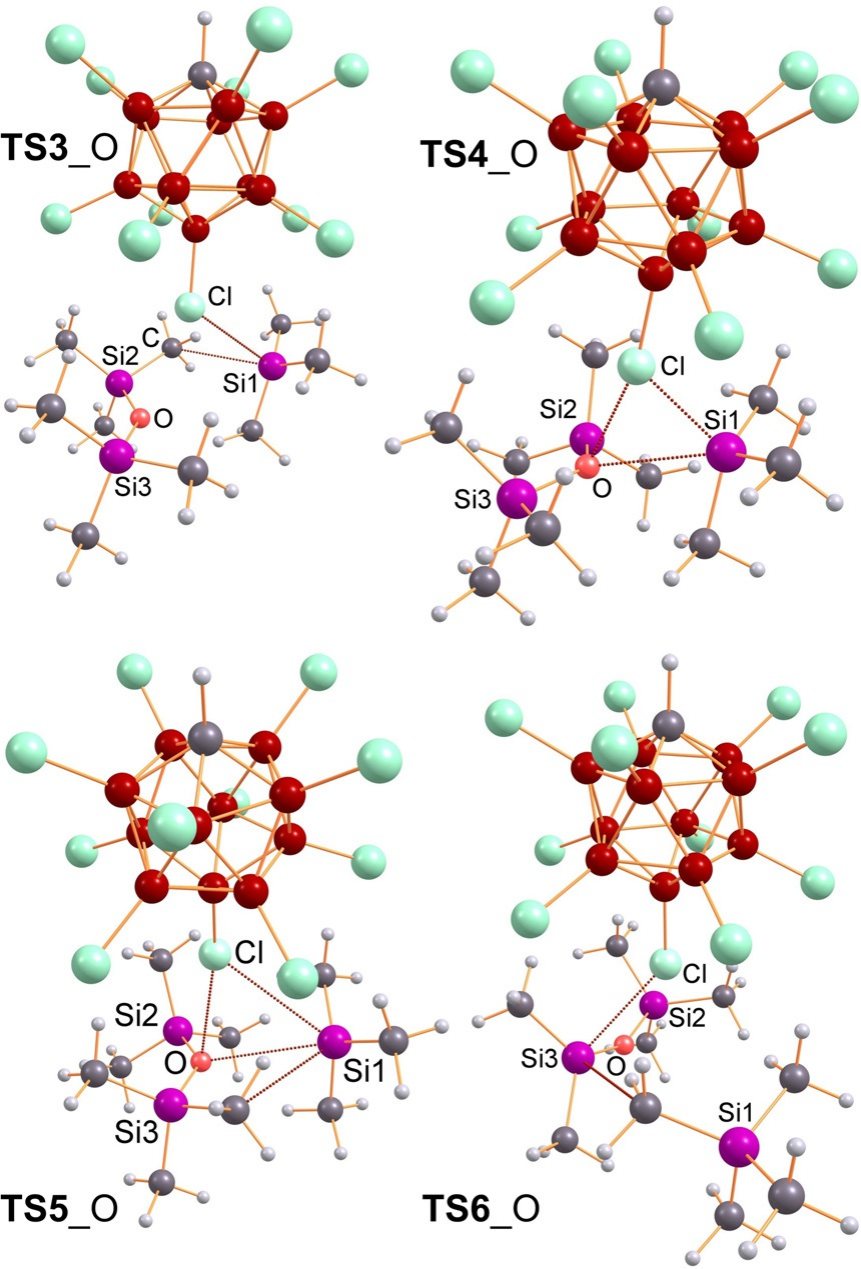
Computed transition states (TS) that play an essential role in the formation of the monocation containing salt **5**O (see also Scheme 6 and Figure 4).

**Figure 6:**
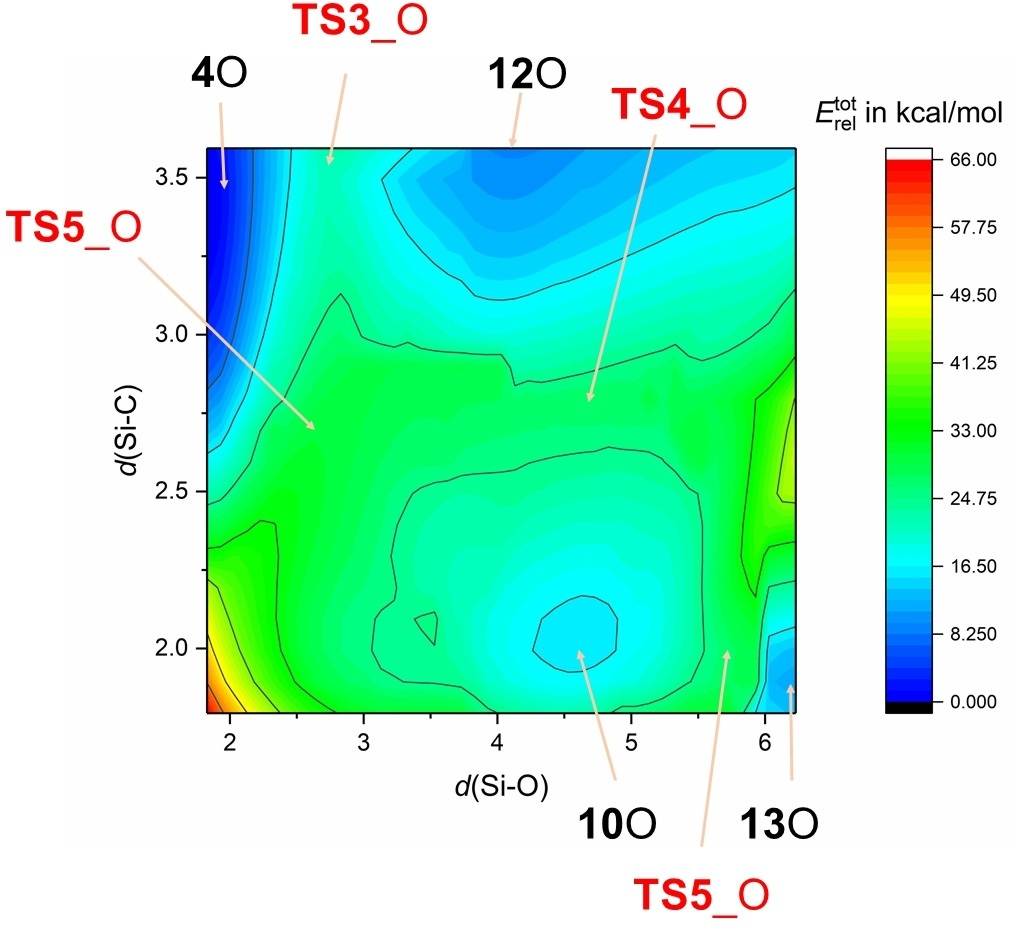
Computed 2‐dimensional heat map displaying all relevant stationary points for the formation of [T_3_Si−O=SiMe_2_][CHB_11_Cl_11_]⋅Me_4_Si (**13**O, *d* in Å) that dimerizes affording **6**O upon Me_4_Si release.

To understand the different reaction channels when carborate anions such as [CHB_11_Cl_11_]^−^ were utilized as counterion, we need to have a closer look first at the reaction in the gas phase. First of all, dication **6**E^2+^ is the dimerization product of **5**E^+^, which can only be formed from **10**E^+^ by the release of Me_4_Si (see Figure 3 and Scheme 6 top). In contrast to [T_3_E]^+^, **10**E^+^, which features no tri‐coordinated silylium ion but a bridging methyl group, is thermodynamically much less favored for both oxygen and sulfur compared to [T_3_E]^+^ (O: 18.1, S: 29.3 kcal mol^−1^). Starting from T‐E‐T and T^+^, both [T_3_E]^+^ and **10**E^+^ can be formed in an exergonic process without any barrier to overcome. However, when [T_3_E]^+^ is formed it needs to be transformed into **10**E^+^ in an endergonic reaction and also the reaction to the monocation **5**E^+^ as well as the dimerization affording **6**E^2+^ are all endergonic. Since the reaction of T‐E‐T and T^+^ was carried out in toluene, the reaction profile was also computed utilizing the corresponding toluene adducts. In this case the formation of the dication **6**E^2+^ is still an endergonic process, while for both chalcogens the formation of the [T_3_E]^+^ ion represents the thermodynamically favored reaction but the process for the formation of [T_3_O]^+^ is less exergonic compared to [T_3_S]^+^ (O: −13.3 vs. S: −22.7 kcal mol^−1^), which is also the case for the naked ion reaction (−35.1 vs. −44.5 kcal mol^−1^). As depicted in Figures 4 and 5, the situation changes significantly, when the whole process is computed utilizing ion pairs with [CHB_11_Cl_11_]^−^ as counterion (Scheme 6 bottom). Since the cations can be attached to different positions at the carborate anion,^54^ many isomers for each class of intermediates were found and activation barriers needed to be localized for each reaction step along the reaction path. Moreover, difficulties to localize true minima (Figure 4) and transition states (Figure 5) arose from the fact that very flat potential energy surfaces were found around the carborate anion, as depicted in the two‐dimensional heat map of Figure 6. In the following, only the thermodynamically most stable isomers are discussed (for further isomers see Supporting Information). The reaction starts with **12**E that describes the T^+^/[CHB_11_Cl_11_]^−^ ion pair along with the weakly bound T‐E‐T molecule. Both reactants are already very close to each other. There are also isomers of **12**E with much larger distances between T^+^ and T‐E‐T (see Supporting Information). Now the exergony of the T_3_E[CHB_12_Cl_12_] (**4**E) formation drops strongly for both oxygen (−2.7 kcal mol^−1^) and sulfur (−10.7 kcal mol^−1^) species but is still larger for sulfur. The most important change, however, is the fact that the formation of the CB/ dication/ CB ion pair **6**E is now thermodynamically favored compared to **4**E for oxygen but not for sulfur (O: −5.5 vs. S: +8.5 kcal mol^−1^), in accord with our experimental findings. For this reason, we had a closer look at the reaction path along the formation of **6**O. Both **4**O and **10**O can be formed directly starting from **12**O with barriers to overcome of 12.8 (**TS3**_O) and 19.3 kcal mol^−1^ (**TS4**_O), respectively. Since only **10**O can decompose affording the monocation stabilized as ion‐pair **13**O, we also computed the activation barrier (**TS5**_O) for the transformation of **4**O to **10**O which amounts to 24.8 kcal mol^−1^. Once **10**O (with a preformed Me_4_Si molecule and a bridging methyl group) is formed, it can easily split one bridging Si−C bond affording the monocation and Me_4_Si (**13**O) in an exergonic process with an activation energy of 11.5 kcal mol^−1^ (**TS6**_O). However, the Me_4_Si is still weakly coordinated to the ion pair. Finally, the release of Me_4_Si leads to **5**O, which can dimerize to give **6**O in an exergonic process. Therefore, the whole process might be regarded as an anion‐mediated transformation.

### Structure and Bonding

**[T‐H‐T]^+^ and [T‐Me‐Si(H)Me_2_]^+^**: Even though we have not isolated the bridging cations shown in Figure 2, but discussed them mechanistically (see the chapter on the ligand scrambling), it is worthwhile to take a closer look at a few structural and bond theory issues. *C*_2_‐symmetric [T‐H‐T]^+^ features two elongated Si−H bonds (1.637, cf. Σ*r*_cov_(Si−H)=1.48 Å)^58^ and a rather large Si‐H‐Si angle (146.6°). Due to the formal hydrogen coordination both Si centers are not planar, exhibiting an averaged Si‐C‐Si angle of 116° (Σ∡Si=348.2°). NBO (natural bond orbital)^68^ analysis localizes a 2‐electron‐3‐center bond along the Si‐H‐Si moiety, which, however, is mainly located at the bridging H atom (67 %) but only with 16.5 % at each Si atom, in accord with computed relatively large negative net charge of −0.31 *e* for the bridging hydrogen atom and MO considerations (Figure S49, S50, MO=molecular orbital). It should be noted that the hydride bridged [Et_3_Si‐H‐SiEt_3_]^+^ ion has been reported by Reed and Nava (vide supra).^44^ NRT (natural resonance theory)^69^, ^70^, ^71^ describes the bonding within [T‐H‐T]^+^ as a resonance between T‐H T^+^↔T^+^ H‐T↔T^+^ H^−^ T^+^. While the first two formulae are by far the most important ones, Lewis formulae like the last one with a hydride H^−^ sandwiched between two T^+^ are at least present in the resonance with a weight of roughly 5 % indicating a non‐negligible hydride character for the ionic hydrogen bridge.

**Scheme 7:**
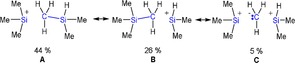
Important NRT Lewis representations of the [T‐Me‐Si(H)Me_2_]^+^ ion.

The [T‐Me‐Si(H)Me_2_]^+^ ion (**8**) shows also a bridging bond, however, a methyl group bridging two Si centers in an asymmetric fashion since both Si centers are differently substituted (Figure 2). Hence a shorter and a slightly longer Si−C_bridge_ distance are observed (2.013 vs. 2.140 Å, cf. Σ*r*_cov_(Si−C)=1.91 Å),^58^ with the shorter bond length to the Si(H)Me_2_ group. The Si−C−Si unit is slightly bent (177.8°) and both Si centers, as well as the bridging methyl group, are slightly pyramidalized (Σ∡Si=345.0 and 341.4, Σ∡C_bridge_=356.8°). The computed partial charges reveal a negatively charged, bridging methyl group (−0.367 *e*) and similar to the situation in [T‐H‐T]^+^, NBO analysis, as well as NRT, find a 2‐electron‐3‐center bond along the Si‐Me‐Si moiety (Scheme 7, resonance between **A** and **B**), in accord with MO and ELF computations (Figure S51, S52, ELF=electron localization function). The Si−Me_bridge_ bond is highly polarized with a localization of 82.5 % at the carbon atom of the methyl group, that means also Lewis representations with a bridging methanide ion (CH_3_^−^) can be discussed (Scheme 7, Lewis formula **C**).

**[T_3_E]^+^**: In agreement with experiment, both naked [T_3_O]^+^ and [T_2_(Me_2_(H)Si)O]^+^ cations were calculated to be almost planar around the oxygen atom in the gas phase (Σ∡(Si‐O‐Si)=358.5° and 359.1°, Table S41), while the analogous sulfur cations exhibit a trigonal pyramidal arrangement (Σ∡(Si‐S‐Si)=329.1°). Interestingly, while for both gas‐phase species all E−Si bonds are equally long, the computed structures of the ion pairs [T_3_E][CB] feature two significantly different E−Si bond lengths in accord with experiment (cf. Table 2 and Table S42). It is well‐known that the smallest oxonium ion [H_3_O]^+^ is a trigonal pyramidal species,^8^ however, successive substitution of H by T results (almost) in planarity for [TH_2_O]^+^, [T_2_HO]^+^ and finally [T_3_O]^+^, while all sulfur ions of the type [T_*n*_H_*m*_S]^+^ (*n*+*m*=3; *n*, *m*=0–3) remain trigonal pyramidal (in accord with the experimentally known [H_3_S]^+^,^4^, ^6^, ^7^ Table S41).^72^, ^73^ Of course, also for sulfur, as can be seen from the structural data, increases the angle sum around the S atom with an increasing number of T groups ([H_3_S]^+^: 284, [TH_2_S]^+^ 293, [T_2_HS]^+^ 311, and [T_3_S]^+^ 329°), which, however, is still far away from 360° that would indicate a planar species. In accordance with these findings, the lone pair located at the chalcogen atom has larger s‐character (smaller *p*‐character, Table S34) with an increasing number of H substituents, which even increases from oxygen to sulfur in accordance with Bent's rule.^74^, ^75^ Obviously, the larger s‐character of the heavier chalcogen atom sulfur favors the pyramidal structure. As seen by the donor‐acceptor energies, the delocalization energies (due to hyperconjugation) increase with the number of T substituents and in all considered chalconium ions (except [H_3_S]^+^), the chalcogen atom E is always negatively charged but as expected oxygen is more negative than sulfur. With an increasing number of T groups, the partial charge at the chalcogen atoms becomes considerably more negative (cf. *Q*(O) −0.77 [H_3_O]^+^ vs. −1.22 [T_3_O]^+^ or 0.17 [H_3_S]^+^ vs. −0.56 *e* [T_3_S]^+^), which is corroborated by an increasing charge transfer from the T groups (Table S34). Hence, within the concept of trimethylsilylium (T^+^) being a large proton (H^+^), it should be noted that besides the larger steric strain, which is introduced upon substitution of H by T,^16^, ^76^ also a larger charge transfer needs to be considered as well as the fact that Si is more electropositive than hydrogen with all the implications according to Bent's rule.

**Figure 7:**
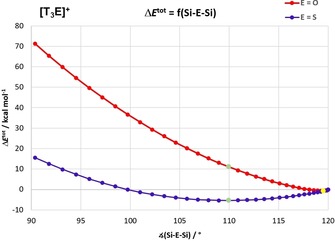
Profiles of the total energy difference with respect to the Si‐E‐Si angles in *C*_3_‐symmetric [T_3_E]^+^ (reference: ∡(Si‐E‐Si)=120.00°). Green dots correspond to ∡(Si‐E‐Si)=109.94° (**geom2**, minimum for E=S) and yellow dots to ∡(Si‐E‐Si)=119.52° (**geom1**, minimum for E=O).

To study the steric influence on the pyramidalization within the [T_3_E]^+^ cations, we first computed the potential energy profile Δ*E* ^tot^ as a function of the Si‐E‐Si angle (between 90 and 120°) in exact *C*_3_ symmetry (Figure 7, Table 3, Table 4). Both species do not adopt exact *C*_3_ symmetry in its lowest‐lying isomer but their metrical parameters almost obey *C*_3_ symmetry. Moreover, the Si_3_O skeleton is not exactly planar (Σ∡(Si‐O‐Si)=358.5°) but all three Si‐O‐Si angles are very close to 120 °C. For this reason, we have defined the exact *C*_3_ symmetry geometry with 120° angles of both species to be the reference for the computation of relative energy contributions (Table 3, Table 4 and S38, S39). In accord with experiment, these computations revealed that for [T_3_O]^+^ the optimized structure (**geom1**) is favored over the exact planar reference state by −0.63 kcal mol^−1^, but the trigonal pyramidal geometry (**geom2**, with Σ∡(Si‐O‐Si)=109.9°) is less favored by 11.07 kcal mol^−1^, which even further increases the smaller the Si‐O‐Si angles becomes (>71 kcal mol^−1^ for 90°). This situation changes for [T_3_S]^+^, which shows a much flatter potential with a shallow minimum at 109.9° (**geom2**) that lies −5.36 kcal mol^−1^ below the reference geometry, representing a true minimum (no imaginary frequencies). Further pyramidalization to ∡(Si‐S‐Si)=90° significantly increases the relative energy to +15.55 kcal mol^−1^.

**Table 3:** Computed structural, NBO and SLA data of [T_3_O]^+^ adopting different structures (energies in kcal mol^−1^).^[a]^

[T_3_O]^+^	**Ref** ^[b]^	**Geom1** ^[b]^	**Geom2** ^[b]^
*d*(Si‐O)/Å	1.822	1.823	1.856
∡(Si‐O‐Si)/°	120.0	119.5	109.9
Σ∡O/°	360.0	358.6	329.8
*Q*(O)/e	−1.220	−1.219	−1.165
*Q*(Si)/e	1.933	1.932	1.894
*Q*(T)	0.740	0.740	0.722
*Q*_CT_(T)/e^[c]^	0.260	0.260	0.278
LP *p*‐AO/%	99.94	99.47	78.95
Loc_(O),σSi‐O_/%	89.24	89.22	88.59
BO_cov_ (Si‐O)	0.202	0.202	0.212
BO_ion_ (Si‐O)	0.750	0.749	0.736
*E* ^ster,NBO^	641.20	641.97	668.57
Δ*E* ^ster,NBO^	0.00	0.77	27.37
Δ*E*(L)^NBO^	0.00	1.07	54.2
Δ*E*(NL)^NBO^	0.00	−1.65	−43.2
*E*(LP)^NBO,del^	30.26	30.16	29.98
Δ*E*_tot_^SCF^	0.00	−0.63	11.07
Δ*E*_s_^SLA^	0.00	−8.11	−23.76
Δ*E*_e_^SLA^	0.00	−0.64	6.10
Δ*E*_q_^SLA^	0.00	8.17	28.75
Δ*E*_Pauli_^SLA[d]^	0.00	8.46	20.75
Δ*E_x_* ^SLA^	0.00	0.05	12.59

[a] Level of theory: PBE1PBE/def2svp including dispersion correction. [b] **Ref**=reference geometry in *C*_3_ symmetry with all Si‐O‐Si angles fixed at 120.0° but all other parameters were freely optimized, **Geom1**=optimized minimum structure of [T_3_O]^+^ with respect to the Si‐O‐Si angle in *C*_3_ symmetry, **Geom2**=optimized minimum structure of [T_3_S]^+^ with respect to the Si‐S‐Si angle in *C*_3_ symmetry (for the O species all other parameters were freely optimized). [c] *Q*_CT_(T)=1‐*Q*(T)=charge transfer onto each (formal) Me_3_Si^+^ ion. [d] *E_q_*[*ρ*]=*E_xc_*[*ρ*]+*E*_Pauli_[*ρ*]^79^ with *E_xc_*[*ρ*]=*E_x_*[*ρ*]+*E_c_*[*ρ*]

**Table 4:** Computed structural, NBO and SLA data of [T_3_S]^+^ adopting different structures (energies in kcal mol^−1^).^[a]^

[T_3_S]^+^	**Ref** ^[b]^	**Geom1** ^[b]^	**Geom2** ^[b]^
*d*(Si‐S)/Å	2.255	2.253	2.271
∡(Si‐S‐Si)/°	120.0	119.5	109.9
Σ∡S/°	360.0	358.6	329.8
*Q*(S)/e	−0.630	−0.626	−0.561
*Q*(Si)/e	1.687	1.685	1.662
*Q*(T)	0.543	0.542	0.520
*Q*_CT_(T)/e^[c]^	0.457	0.458	0.480
LP *p*‐AO/%	99.99	97.76	69.07
Loc_(O),σSi‐S_/%	79.11	79.03	77.71
BO_cov_ (Si−S)	0.395	0.397	0.420
BO_ion_ (Si−S)	0.566	0.564	0.533
*E* ^ster,NBO^	512.07	514.50	551.50
Δ*E* ^ster,NBO^	0.00	2.43	37.00
Δ*E*(L)^NBO^	0.00	−1.34	−2.63
Δ*E*(NL)^NBO^	0.00	0.61	−2.70
*E*(LP)^NBO,del^	30.50	30.12	23.96
Δ*E* ^tot,SCF^	0.00	−0.76	−5.36
Δ*E*_s_^SLA^	0.00	−1.93	−51.34
Δ*E*_e_^SLA^	0.00	−3.08	−4.83
Δ*E*_q_^SLA^	0.00	4.29	50.84
Δ*E* ^Pauli,SLA^	0.00	4.35	50.84
Δ*E* ^xc,SLA[d]^	0.00	0.07	2.31

[a] Level of theory: PBE1PBE/def2svp including dispersion correction. [b] **Ref**=reference geometry in *C*_3_ symmetry with all Si‐S‐Si angles fixed at 120.0° but all other parameters were freely optimized, **Geom1**=optimized minimum structure of [T_3_O]^+^ with respect to the Si‐O‐Si angle in *C*_3_ symmetry (for the S species all other parameters were freely optimized), **Geom2**=optimized minimum structure of [T_3_S]^+^ with respect to the Si‐S‐Si angle in *C*_3_ symmetry. [c] *Q*_CT_(T)=1‐*Q*(T)=charge transfer onto each (formal) Me_3_Si^+^ ion. [d] *E_q_*[*ρ*]=*E_xc_*[*ρ*]+*E*_Pauli_[*ρ*]^79^ with *E_xc_*[*ρ*]=*E_x_*[*ρ*]+*E_c_*[*ρ*].

To determine the origin of the different minimum structures (almost planar [T_3_O]^+^ vs. trigonal pyramidal [T_3_S]^+^), we performed NBO analyses along the energy profile for the corresponding different Si‐E‐Si angles. Natural steric analysis as implemented in the NBO6 program^77^ expresses steric exchange repulsion as the energy difference due to orbital orthogonalization.^78^ The absolute values, as well as the relative values, increase with decreasing Si‐E‐Si angle, however, the steric strain with respect to the reference geometry was calculated to be considerably larger for the sulfur species (cf. S: **geom1**: 2.4/ **geom2**: 37.0 vs. O: 0.8/ 27.4 kcal mol^−1^, Table 3, Table 4). Obviously, there must be a second effect that overcompensates the increased steric strain in the pyramidal sulfur geometry. For this reason, we looked at delocalization effects using standard NBO deletion techniques.

For oxygen, the localized *E*(L) value favors the planar coordination over the pyramidal (**geom2**) by 54.2, while the delocalized contribution, *E*(NL) with −43.2 kcal mol^−1^ is in favor of the pyramidal structure, which, however, does not compensate the localization contribution. The stability of the planar structure can, therefore, be attributed to the electronic localization energy *E*(L). This picture clearly changes for the sulfur species for which both contributions, *E*(L) and *E*(NL), favor the pyramidal geometry by −2.6 and −2.7 kcal mol^−1^. Moreover, both energy values are much smaller compared to those of the oxygen species (Table 3, Table 4). As expected the differences between the reference geometry and **geom1** are much less pronounced for both chalcogen species.

**Scheme 8:**
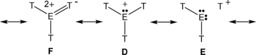
Lewis representations of [T_3_E]^+^.

Interestingly, the delocalization of the lone pair located at the chalcogen atom is the main contributor to the delocalization effect (hyperconjugation), which, however, does not much change upon decreasing the Si‐O‐Si angle from 120 to 109° (ca. 30 kcal mol^−1^). Hence, hyperconjugation due to lone pair (LP) delocalization is not the main reason for the energetically favored planar arrangement of the Si_3_O skeleton in [T_3_O]^+^ but the decreased steric repulsion and the favourable localization energy. In case of [T_3_S]^+^ for the 120° reference species, also a value of 30.5 kcal mol^−1^ was found for the delocalization of the LP, which means the hyperconjugative effect is as large as for [T_3_O]^+^, but this delocalization effect considerably decrease upon pyramidalization by ca. 6.5 kcal mol^−1^. Besides wave function‐based methods (e.g., as in NBO anaylsis) to study the steric influence within a molecule, there are density functional theory (DFT) based methods, which are completely different in their approach and may even lead to qualitatively different results as those found by wave function‐based methods. In 2007 Shubin Liu introduced an interesting DFT based approach for a new energy decomposition analysis (SLA=Shubin Liu analysis^80^ as implemented by Tian Lu in MULTIWFN,^81^ Table 3, Table 4) that can be used to study steric effects as shown by his group in a series of papers.^80^, ^82^, ^83^, ^84^, ^85^, ^86^, ^87^ According to SLA, the total energy density functional is expressed as the sum of steric, electrostatic and quantum effects that represent independent energy contributions: *E*[*ρ*]=*E_s_*[*ρ*]+*E_e_*[*ρ*]+*E_q_*[*ρ*]. According to this expression, Liu could demonstrate that the steric effect has to do with the energetic contribution from the minimal space upheld by atoms in molecules with all other effects (such as electrostatic and quantum) totally excluded. According to this definition, the steric contribution *E_s_*[ρ] has nothing to do with the Pauli repulsion, since the Pauli energy^79^ is included in *E_q_*[ρ], the fermionic quantum energy, which includes both the potential and kinetic contributions due to the exchange‐correlation interactions in a system. Appling SLA for [T_3_O]^+^ and [T_3_S]^+^ with a planar and trigonal pyramidal Si_3_E geometry (Table 3, Table 4), we found that for [T_3_O]^+^ both steric repulsion (Δ*E*_s_^SLA^) as well as quantum effects (Δ*E*_q_^SLA^) are the major contributors while electrostatics (Δ*E*_e_^SLA^) plays a minor role. However, while for **geom1** (the almost planar minimum structure of [T_3_O]^+^) both major contributions almost cancel each other (−8.1 vs. 8.2 kcal mol^−^), the small electrostatic contribution stabilizes the small deviation from planarity (−0.6 kcal mol^−1^). The situation changes for the pyramidal structure **geom2**, which still shows a smaller steric repulsion compared to the planar reference structure, but this effect is now overcompensated by quantum contributions. In addition, electrostatics also favors planarity, which results in a less favorable pyramidal structure. In accord with NBO analysis, the major contribution to the quantum energy is the Pauli energy, which significantly increases with a smaller Si‐O‐Si angle. In contrast to [T_3_O]^+^, [T_3_S]^+^ prefers the pyramidal structure, which is energetically favored by 5.4 kcal mol^−1^ over the planar reference structure. Again steric and quantum effects are the major contributors and they also almost cancel each other (−51.3 vs. 50.8 kcal mol^−1^) but in contrast to [T_3_O]^+^ the much smaller electrostatic effect favors the pyramidal structure by −4.8 kcal mol^−1^. In summary, both steric as well as quantum effects are the main effects according to DFT, however, they cancel each other to a large extent. Hence, the smaller electrostatic contribution, which favors the pyramidal structure in [T_3_S]^+^ but the planar arrangement in [T_3_O]^+^, becomes important. According to natural resonance theory (Scheme 8), the best Lewis structure of all considered [T_3_E]^+^ geometries is always a structure with three highly polarized Si−E bonds (for degree of polarization see Table 3, Table 4) and one lone pair located on the chalcogen in a pure *p*‐type atomic orbital (>99 % p character in the planar case). Lewis formula **D** includes between 55–61 % weight, while structures with a neutral T‐E‐T and a formal T^+^ fragment possess less than 12 % (**E**). Therefore, all these species are indeed formal chalconium ions of the type [T_3_E]^+^ rather than T‐E‐T stabilized silylium ions: T_2_E→T^+^. Lewis representations of type **F** describe delocalization effects of the lone pair (e.g., into σ*(Si−C) orbitals, vide infra) and amount to 3 (E=S) −5 % (E=O).

**Scheme 9:**
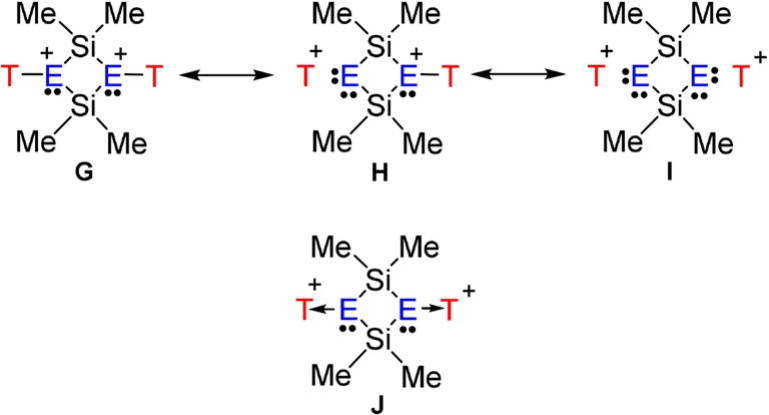
Lewis representations of 6E^2+^ (G and H describe pnictonium species while I and J feature silylium ions stabilized by a donor‐acceptor bond.

**[T‐μE‐SiMe_2_E]_2_** ^**2+**^: In agreement with the X‐ray data, the [T‐μO‐SiMe_2_O]_2_^2+^ ion is characterized by a centrosymmetric planar 4‐membered Si_2_O_2_ ring featuring two tricoordinated oxonium atoms in a planar environment (see Figure 1, Table 1 and S42), while the terminal T groups are slightly bent out of the ring plane (8.4°). Within the ring, both Si−O bond lengths are slightly shorter compared to both terminal distances (1.770 vs. 1.859 Å). Similar structural features are found for the sulfur species, however, the bending out of the Si_2_O_2_ ring plane of the terminal T groups is much stronger pronounced (59.6°). Hence, again the oxygen is an almost trigonal environment (Σ∡(Si‐O‐Si)=359.9°), while sulfur prefers a pyramidal arrangement (Σ∡(Si‐S‐Si)=325.0°). The difference in the Si−E bond lengths within the ring compared to the terminal distances is best explained by a strong hyperconjugative effect of the lone pairs localized at both tricoordinated chalcogen atoms in a *p*‐type atomic orbital as indicated by NBO investigations. Within the ring system, this delocalization effect [LP(E)→σ*(Si‐C)] is much stronger compared to that with the terminal Si−C bonds (O: 35.0 vs. 10.3 and S: 17.8 vs. 7.2 kcal mol^−1^) and introduces even partial Si−O double bond character. However, this type of hyperconjugation is the main contribution to the overall delocalization effect that is associated with the two chalcogen lone pairs (O: 65.0 and S: 47.6 kcal mol^−1^). According to NBO analysis, a Lewis representation with three highly polarized Si−O bonds is favored (Scheme 9, formula **G**). However, there are also smaller hyperconjugative effects, which can be associated with Lewis representations such as **H** and **I**. It is therefore not completely out of place to bring a possible donor‐acceptor adduct notation (formula **J**, a cyclo‐disila‐dichalcotane doubly silylated) into play, although Lewis formula **G** is certainly the best description in the picture of localized bonding orbitals.^49^ It should be noted that there are a variety of computational studies on oxonium and sulfonium species in literature.^49^, ^66^, ^72^, ^73^, ^74^, ^87^, ^88^, ^89^, ^90^, ^91^, ^92^

**Ion‐pairs**: As discussed before, ion‐pair formation stabilizes all silylium ions mentioned here. In particular, the highly reactive T^+^ ion in [T]CB is strongly stabilized as can be seen from the short Si‐Cl distance of 2.273 Å (cf. 2.54 Å in [*i*Pr_3_SiOH_2_]_2_[B_12_Cl12] or 2.317–2.355 Å in [(Et_3_Si)_2_][B_12_Cl_12_]),^49^ which is in the range of a typical polarized Si−Cl single bond (cf. Σ*r*_cov_(Si‐Cl)=2.17 and Σ*r*_vdW_(Si⋅⋅⋅Cl)=3.85 Å),^59^, ^94^ and the rather large charge transfer to the T^+^ group (0.40 *e*, Table 5). With respect to the charge transfer and shortest Si_cation_⋅⋅⋅Cl_anion_ distances, both chalcogen monocations **5**E^+^ are also significantly bound to the anion (*Q*_CT_=0.38 (O) and 0.39 *e* (S)), while both [T_3_E]^+^ and dications **6**E^2+^ display rather large interionic (Si−Cl) distances and a considerably smaller charge transfer (0.11/0.12 *e*).

**Table 5:** Calculated shortest Si_cation_⋅⋅⋅Cl_anion_ distance (Å), cation and chalcogen (E) charges (*e*) as well the charge transfer, *Q*_CT_ (*e*), from NBO analysis.

Ion pairs	*d*_s_(Si−Cl)^[a]^	*q*(cat)^[b]^	*Q*_CT_^[c]^	*q*(E)
[T]CB	2.273	0.599	0.401	–
[T_3_O]CB (**4**O)	3.299	0.885	0.115	−1.212
[T_3_S]CB (**4**S)	3.332	0.881	0.119	−0.585
**5**O	2.309	0.621	0.379	−1.243
**5**S	2.303	0.607	0.393	−0.574
**6**O	3.438	0.887	0.114	−1.248
**6**S	3.507	0.880	0.120	−0.530

[a] *d*_s_=shortest Si−Cl distances. [b] *q*(CB)=−*q*(cat). [c] Charge transfer (*Q*_CT_) from the anion to the cation=1−*q*(cat).

## Conclusion

The reaction of bis(trimethyl)silylether and ‐thioether, T‐E‐T (E=O, S; T=Me_3_Si), with trimethylsilylium ions (T^+^) in the presence of weakly coordinating anions has been investigated experimentally and theoretically in detail. In the case of T‐S‐T, the reaction with [T‐H‐T][B(C_6_F_5_)_4_] led to the formation of the previously unknown persilylated sulfonium cation, [T_3_S][B(C_6_F_5_)_4_], in a straightforward silylation reaction. When, however, T‐O‐T is reacted with [T‐H‐T][B(C_6_F_5_)_4_], kinetic stress, introduced by less space around the oxygen atom, leads to a ligand exchange reaction, which resulted in the in situ formation of the smaller [(Me_2_(H)Si]^+^ ion. This, in turn, generates in an exergonic reaction with T‐O‐T the correspondingly smaller oxonium ion, [T_2_(Me_2_(H)Si)O]^+^ with [B(C_6_F_5_)_4_]^−^ as counterion, which could be isolated as salt and fully characterized. This rather surprising reaction is thermodynamically unfavorable for the analogous sulfur species. To prevent the ligand scrambling reaction with Me_3_Si‐H, both T‐E‐T compounds were also reacted with Me_3_Si[CB] salts (CB^−^=[CHB_11_H_5_Cl_6_]^−^ and [CHB_11_Cl_11_]^−^). While the reaction with T‐S‐T again led to the formation of the corresponding [T_3_S]^+^ carborate salt upon release of Me_4_Si, the formation of a [T−O=SiMe_2_]^+^ monocation was observed in the solution for the reaction with T‐O‐T. The monocation [T−O=SiMe_2_]^+^ easily dimerizes upon crystallization and salts bearing the cyclic dication [T‐μO‐SiMe_2_]_2_^2+^ could be isolated and fully characterized. Theoretical studies on the formation of the dication [T‐μO‐SiMe_2_]_2_^2+^ showed that an anion‐mediated reaction similar to a template reaction on an anion is a prerequisite for the formation of this dication, since all reaction intermediates are stabilized by a considerable charge transfer from the anion. In addition, DFT studies show that all oxonium ions prefer an almost planar structure around the oxygen atom, while sulfonium ions favor a trigonal pyramidal structure even in the cyclic dication.

## Experimental Section

Experimental and computational details can be found in the Supporting Information.

## Conflict of interest

The authors declare no conflict of interest.

## Supporting information

As a service to our authors and readers, this journal provides supporting information supplied by the authors. Such materials are peer reviewed and may be re‐organized for online delivery, but are not copy‐edited or typeset. Technical support issues arising from supporting information (other than missing files) should be addressed to the authors.

Supplementary
