# Supplementary material for: On Silylated Oxonium and Sulfonium Ions and Their Interaction with Weakly Coordinating Borate Anions
Source: Chemistry. 2020 Jan 24;26(7):1640–52. doi: 10.1002/chem.201904403 (PMC7028070; doi:10.1002/chem.201904403)
Supplement: Supplementary file 1 — Supplementary [file CHEM-26-1640-s001.pdf]

CHEMISTRY
A European Journal

Supporting Information

On Silylated Oxonium and Sulfonium Ions and Their Interaction with Weakly Coordinating Borate Anions

Kevin Bläsing,[a] Rene Labbow,[a] Dirk Michalik,[a, b] Fabian Reiß,[a, b] Axel Schulz,*[a, b] Alexander Villinger,[a] and Svenja Walker[a]

chem_201904403_sm_miscellaneous_information.pdf

This file includes

Contents

1 Experimental 2

2 Structure Elucidation 4

3 Starting and reference materials 8

3.1 Hexamethyldisilathiane – $(Me_3Si)_2S$ 8

3.2 Hexamethyldisiloxane – $(Me_3Si)_2O$ 9

4 Synthesis of compounds 10

4.1 Potassium [18]crown-6 trimethylsilylsulfide – [K·[18]crown-6][$SSiMe_3$] 10

4.2 Potassium [18]crown-6 trimethylsilyloxid – [K·[18]crown-6][$OSiMe_3$] 11

4.3 Synthesis and characterization of tris(trimethylsilyl)sulfonium *closo*-7,8,9,10,11,12-hexachloropentahydroundecaborate – $[(Me_3Si)_3S][CHB_{11}H_5Cl_6]$ 12

4.4 Synthesis and characterization of tris(trimethylsilyl)sulfonium *closo*-2,3,4,5,6,7,8,9,10,11,12-undecachloroundecaborate – $[(Me_3Si)_3S][CHB_{11}Cl_{11}]$ 13

4.5 Synthesis and characterization of tris(trimethylsilyl)sulfonium tetrakis(pentafluorophenyl)borate – $[(Me_3Si)_3S][B(C_6F_5)_4]$ 14

4.6 Synthesis and characterization of bis(trimethylsilyl)-mono-(dimethylsilyl)oxonium tetrakis(pentafluorophenyl)borate – $[T_2(Me_2(H)Si)O][B(C_6F_5)_4][B(C_6F_5)_4]$ 16

4.7 Synthesis and characterization of bis-pentamethyldisilyloxonium undecachloroundecaborate – $[Me_3Si\text{-}\mu\text{-}OSiMe_2]_2[CHB_{11}Cl_{11}]_2$ 34

5 NMR, IR and Raman spectra 35

6 Computational Details 81

7 References 275

1 Experimental

General Information. All manipulations were carried out under oxygen- and moisture-free conditions under argon using standard Schlenk or drybox techniques.

The trimethylsilane adduct of trimethylsilylium tetrakis(pentafluorophenyl)borate $[(Me_3Si)_2H][B(C_6F_5)_4]$ was prepared according to a reported literature procedure.[1,2] Trimethylsilylium *closo*- 7, 8, 9, 10, 11, 12 - hexachloropentahydroundecaborate $[Me_3Si][CHB_{11}H_5Cl_6]$ and trimethylsilylium *closo*- 2, 3, 4, 5, 6, 7, 8, 9, 10, 11, 12- undeca-chloroundecaborate $[Me_3Si][CHB_{11}Cl_{11}]$ were prepared according to reported literature procedure.[4–9] Hexamethyldisiloxane $(Me_3Si)_2O$ (99 %, Merck) was dried over Na and freshly distilled prior to use. Trimethylsilylchloride Me_3SiCl (99 %, Merck) was dried over CaH_2 and freshly distilled prior to use. Lithium Li (≥99 %, Merck) and sulfur S_8 (VEB Laborchemie Apolda) were used as received. Potassium *tert*-butoxide $K[OC(CH_3)_3]$ (>97 %, Fluka) was sublimed *in vacuo* ($1\cdot10^{-3}$ mbar) at 220 °C. 1,4,7,10,13,16-Hexaoxa-*cyclo*-octadecane [18]crown-6 (99 %, Fluka) was recrystallized twice from acetonitrile and afterwards twice from THF. Hexamethyldisilathiane $(Me_3Si)_2S$ was prepared according to modified reported literature procedure (*vide infra*).[3] Toluene and Tetrahydrofuran THF were dried over Na/benzophenone and freshly distilled prior to use. *n*-Pentane was dried over Na/benzophenone/tetraglyme and freshly distilled prior to use. 1,2-dichlorobenzene 1,2-DCB was dried over P_4O_{10} and afterwards over CaH_2 and was freshly distilled prior to use.

NMR: ^{29}Si INEPT; ^{29}Si{^{1}H}; ^{19}F{^{1}H}; ^{17}O; ^{13}C{^{1}H}; ^{11}B and ^{1}H NMR spectra were obtained on a Bruker AVANCE 250, AVANCE 300 or AVANCE 500 and were referenced internally to the deuterated solvent (^{13}C, CD_2Cl_2: $\delta_{ref.}$ = 54 ppm, acetone-[D_6]: $\delta_{ref.}$ = 29.84 ppm, toluene-[D_8]: $\delta_{ref.}$ =.43 ppm, benzene-[D_6]: $\delta_{ref.}$ = 128.06 ppm, THF-[D_8]: $\delta_{ref.}$ = 25.31 ppm, DMSO-[D_6]: $\delta_{ref.}$ = 39.52 ppm) or to protic impurities in the deuterated solvent (^{1}H, $CDHCl_2$: $\delta_{ref.}$ = 5.32 ppm, acetone-[D_5(H)] $\delta_{ref.}$ = 2.05 ppm, toluene-[D_7(H)] $\delta_{ref.}$ = 2.08 ppm, benzene-[D_5(H)] $\delta_{ref.}$ = 7.16 ppm, THF-[D_7(H)] $\delta_{ref.}$ = 1.72 ppm, DMSO-[D_5(H)]: $\delta_{ref.}$ = 2.50 ppm).[10] ^{29}Si, ^{19}F{^{1}H}, ^{17}O and ^{11}B NMR spectra were referenced externally (^{29}Si: δ_{Me_4Si} = 0 ppm, ^{19}F: δ_{CCl_3F} = 0 ppm, ^{17}O: δ_{H_2O} = 0 ppm, ^{11}B: $\delta_{BF_3\cdot OEt_2}$ = 0 ppm, Deuterated dichloromethane CD_2Cl_2 was distilled over P_4O_{10} and afterwards freshly distilled over CaH_2 prior to use. Deuterated acetone acetone-[D_6] and DMSO-[D_6] was stored over molecular sieves (3 Å). Deuterated toluene toluene-[D_8], deuterated benzene benzene-[D_6] and deuterated tetrahyrodufran THF-[D_8] were

freshly distilled over sodium prior to use. THF-[D_8] was stored over molecular sieves (3 Å) at 5 °C.

Measurement of [$(Me_3Si)_3S$][$B(C_6F_5)_4$] in toluene or 1,2-DCB were performed with CD_2Cl_2 or acetone-[D_6] as external reference (denoted as ext. ref. CD_2Cl_2 or acetone-[D_6]); $\delta_{ref.}$ *vide supra.*), which was added in a second smaller inner precision glass tube.

IR: A Nicolet 380 FT-IR with a Smart Orbit ATR device was used.

ICP: IRIS INTREPID II from Thermo Fisher was used.

Raman: a) LabRAM HR 800 Horiba Jobin YVON Raman spectrometer equipped with an Olympus BX41 microscope with variable lenses was used. The samples were excited by an air-cooled infrared diode laser (785 nm) or a red HeNe laser (633 nm). b) Bruker VERTEX 70 FT-IR with RAM II FT-Raman module equipped with a Nd:YAG laser (1064 nm) was used.

CHN analyses: Analysator Flash EA 1112 from Thermo Quest was used.

Melting point: Melting points are uncorrected. EZ-Melt, Stanford Research Systems was used (Heating rate 20 °C·min^{-1}, clearing-points are reported).

DSC: DSC 823e from Mettler Toledo was used (Heating rate 5 °C·min^{-1}, peaks are reported).

MS: Finnigan MAT95-XP from Thermo Electron was used.

2 Structure Elucidation

X-ray Structure Determination: X-ray quality crystals were selected in Fomblin YR-1800 perfluoroether (Alfa Aesar) at ambient temperature. The samples were cooled to 123(2) K (**[K [18]crown-6]S-SiMe$_3$**, **[K·[18]crown-6]O-SiMe$_3$** and **[T-μ-O-SiMe$_2$]$_2$[CHB11Cl11]** or 173(2) K (**[T$_3$S][CHB$_{11}$H$_5$Cl$_6$]**, **[T$_3$S][B(C$_6$F$_5$)$_4$]** and **[T$_2$(Me$_2$(H)Si)O][B(C$_6$F$_5$)$_4$]**) during measurement. The data was collected on a Bruker Apex Kappa-II CCD diffractometer or a Bruker D8 Quest Diffraktometer using graphite monochromated Mo-Kα radiation (λ = 0.71073 Å). The structures were solved by direct methods (*SHELXS-2013*)[11] and refined by full-matrix least squares procedures (*SHELXL-2013*).[12] Semi-empirical absorption corrections were applied (*SADABS*).[13] All non-hydrogen atoms were refined anisotropically, hydrogen atoms were included in the refinement at calculated positions using a riding model. Disorder problems are commented in the cif files. A figure of the complex disorder in **[T$_2$(Me$_2$(H)Si)O][B(C$_6$F$_5$)$_4$]·toluene** is shown in Figure S1.

Figure S1: Graphical representation of the disorder in **[T$_2$(Me$_2$(H)Si)O][B(C$_6$F$_5$)$_4$]·toluene**. Hydrogen atoms of the aryl and methyl groups not shown.

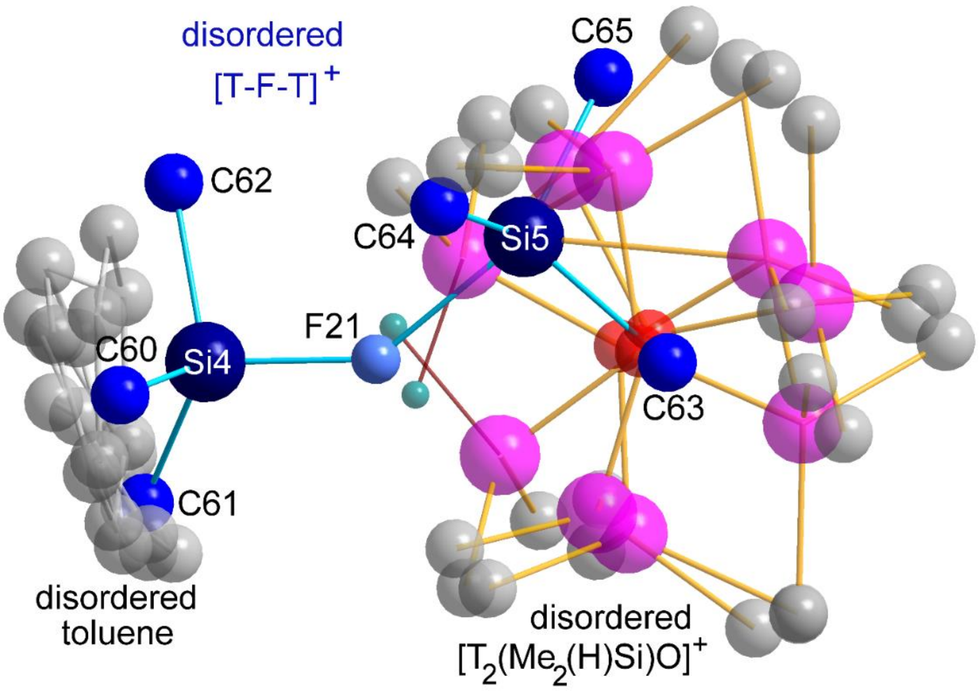

Table S1: Crystallographic details of **[K·[18]crown-6]SiMe$_3$** and **[K·[18]crown-6]O-SiMe$_3$**.

	**[K·[18]crown-6]S-SiMe$_3$**	**[K·[18]crown-6]O-SiMe$_3$**
Chem. formula	$C_{15}H_{33}KO_6SSi$	$C_{15}H_{33}KO_7Si$
M [g·mol^{-1}]	408.66	392.60
Color	colorless	colorless
Cryst. system	orthorhombic	orthorhombic
Space group	$P2_12_12_1$	*Fdd2*
a [Å]	8.3604(5)	30.565(2)
b [Å]	14.4338(12)	34.690(2)
c [Å]	18.3651(14)	8.0438(3)
α [°]	90	90
β [°]	90	90
γ [°]	90	90
V [Å^3]	2216.2(3)	8528.8(7)
Z	4	16
ρ [g·cm^{-3}]	1.225	1.223
μ [mm^{-1}]	0.41	0.33
$\lambda_{MoK\alpha}$ [Å]	0.71073	0.71073
T [K]	123	123
Measured reflections	21825	52527
Independent reflections	5889	6731
Reflections with $I > 2\sigma(I)$	4764	5532
$R_{int.}$	0.078	0.039
$F(000)$	880	3392
R_1 (R[$F^2 > 2\sigma(F^2)$])	0.042	0.044
wR_2 (F^2)	0.077	0.111
Goof	1.024	1.04
Parameters	286	220
CCDC #	1927630	1937577
Data block name	is_rl318	kbrl05b

Table S2: Crystallographic details of **$[T_3S][B(C_6F_5)_4]$** and **$[T_3S][CHB_{11}H_5Cl_6]$**·toluene.

	**$[T_3S][B(C_6F_5)_4]$**	**$[T_3S][CHB_{11}H_5Cl_6] \cdot C_7H_8$**
Chem. formula	$C_{33}H_{27}BF_{20}SSi_3$	$C_{17}H_{40.91}B_{11}Cl_{6.09}SSi_3 \cdot C_7H_8$
M [g·mol^{-1}]	930.68	696.45
Color	colorless	colorless
Cryst. system	monoclinic	monoclinic
Space group	$P2_1/c$	$P2_1/n$
a [Å]	25.307(2)	10.3018(5)
b [Å]	15.926(2)	12.9930(6)
c [Å]	19.700(2)	27.1470(13)
α [°]	90	90
β [°]	91.977(4)	90.554(3)
γ [°]	90	90
V [Å^3]	7935.0(10)	3633.5(3)
Z	8	4
ρ [g·cm^{-3}]	1.558	1.273
μ [mm^{-1}]	0.29	0.65
$\lambda_{MoK\alpha}$ [Å]	0.71073	0.71073
T [K]	173	173
Measured reflections	63968	53382
Independent reflections	12520	10606
Reflections with $I > 2\sigma(I)$	6789	6344
$R_{int.}$	0.109	0.084
$F(000)$	3744	1438
R_1 (R[$F^2 > 2\sigma(F^2)$])	0.092	0.049
wR_2 (F^2)	0.235	0.123
Goof	1.02	1.02
Parameters	1166	416
CCDC #	1927632	1927629
Data block name	is_frl172	is_rl55_1

Table S3: Crystallographic details of **$[T_2(Me_2(H)Si)O][B(C_6F_5)_4]$**·toluene and **$[T\text{-}\mu O\text{-}SiMe_2]_2[CHB_{11}Cl_{11}]_2$**·4 toluene.

	**$[T_2(Me_2(H)Si)O][B(C_6F_5)_4]$**	**$[T\text{-}\mu O\text{-}SiMe_2]_2[CHB_{11}Cl_{11}]$**
Chem. formula	$C_{24}BF_{20}^{-}\cdot 0.873(C_8H_{25}OSi_3^{+})\cdot 0.126(C_6H_{18}FSi_2^{+})\cdot 0.803(C_7H_8)$	$2(CHB_{11}Cl_{11}^{-})\cdot C_{10}H_{30}O_2Si_4^{2+}\cdot 4(C_7H_8)$
M [g·mol^{-1}]	963.04	1706.99
Color	colorless	colorless
Cryst. system	monoclinic	triclinic
Space group	$P2_1/c$	P_1
a [Å]	11.2015(4)	11.2506(6)
b [Å]	24.2654(9)	12.2528(7)
c [Å]	16.6551(6)	15.9664(8)
α [°]	90	99.822(2)
β [°]	107.597	107.109(2)
γ [°]	90	107.158(2)
V [Å^3]	4315.2(3)	1928.5(2)
Z	4	1
ρ [g·cm^{-3}]	1.482	1.470
μ [mm^{-1}]	0.22	0.88
$\lambda_{MoK\alpha}$ [Å]	0.71073	0.71073
T [K]	173	123
Measured reflections	44809	11247
Independent reflections	8457	11247
Reflections with $I > 2\sigma(I)$	5663	7954
$R_{int.}$	0.040	0.048
$F(000)$	1943	860
R_1 (R[$F^2 > 2\sigma(F^2)$])	0.077	0.048
wR_2 (F^2)	0.233	0.077
Goof	1.02	1.07
Parameters	641	417
CCDC #	1927633	1927631
Data block name	av_frl149	is_kbrl03

3 Starting and reference materials

3.1 Hexamethyldisilathiane – $(Me_3Si)_2S$

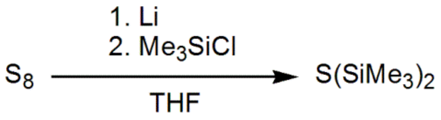

Finley powdered sulfur S_8 (2 g, 62 mmol) was suspended in 40 mL THF and cooled to 0 °C. Also finely powdered lithium Li (0.9 g, 125 mmol) was added to this suspension. The flask was set with a pressure-equalized reflux condenser and a dropping funnel. Trimethylsilylchloride Me_3SiCl (13.6 g, 125 mmol) was added dropwise at temperatures between 0 °C and 5 °C over a period of 2.5 h to the immediately reddish brown darkened solution. THF was removed by thermal distillation. The crude product was fractional distilled *in vacuo* (47 mbar) at 74 °C, yielding in 9.2 g (52 mmol, 83 %) colorless hexamethyldisilathiane $(Me_3Si)_2S$.

$C_6H_{18}SSi_2$ (178.44 g/mol): **bp**. 74 °C (47 mbar). **^{1}H NMR** (25 °C, CD_2Cl_2, 300.13 MHz): δ = 0.34 (s, 18H, SiC*H*$_3$, $^2J(^1H\text{-}^{29}Si)$ = 6.8 Hz, $^1J(^1H\text{-}^{13}C)$ = 120.6 Hz). **^{1}H NMR** (25 °C, 1,2-DCB, ref. ext. acetone-[D_6], 300.13 MHz): δ = 0.37 (s, SiC*H*$_3$, $^1J(^1H\text{-}^{13}C)$ = 120 Hz). **^{1}H NMR** (25 °C, THF-[D_8], 300.13 MHz): δ = 0.32 (s, SiC*H*$_3$, $^1J(^1H\text{-}^{29}Si)$ = 6.9 Hz, $^1J(^1H\text{-}^{13}C)$ = 120 Hz). **^{13}C{^{1}H} NMR** (25 °C, CD_2Cl_2, 75.47 MHz): δ = 4.46 (s, Si*C*H$_3$, $^1J(^{13}C\text{-}^{29}Si)$ = 54 Hz). **^{13}C{^{1}H} NMR** (25 °C, THF-[D_8], 75.47 MHz): δ = 4.44 (s, Si*C*H$_3$, $^1J(^{13}C\text{-}^{29}Si)$ = 54 Hz). **^{29}Si INEPT NMR** (25 °C, CD_2Cl_2, 59.62 MHz): δ = 14.61 (dec, *Si*CH$_3$, $^2J(^{29}Si\text{-}^1H)$ = 6.9 Hz). **^{29}Si INEPT NMR** (25 °C, THF-[D_8], 59.62 MHz): δ = 14.03 (dec, *Si*CH$_3$, $^1J(^1H\text{-}^{29}Si)$ = 6.9 Hz) **IR** (ATR, 8 scans, 25 °C, cm^{-1}): 2956 (w), 2898 (w), 1448 (w), 1403 (w), 1321 (w), 1247 (s), 1062 (w), 925 (w), 835 (s), 815 (s), 752 (m), 690 (m), 624 (s). **Raman** (632 nm, 12 mW, 20 s, 10 acc., 25 °C, cm^{-1}): 2960 (3), 2899 (8), 1448 (1), 1411 (1), 1263 (1), 1250 (1), 863 (1), 844 (1), 754 (1), 693 (1), 638 (9), 487 (1), 438 (10), 240 (2), 220 (2), 181 (3), 164 (2).

3.2 Hexamethyldisiloxane – $(Me_3Si)_2O$

$C_6H_{18}OSi_2$ (162.38 g/mol): **bp**. 101 °C. **^{1}H NMR** (25 °C, CD_2Cl_2, 300.13 MHz): δ = 0.17 (s, SiCH_3, $^2J(^1H-^{29}Si)$ = 6.7 Hz, $^1J(^1H-^{13}C)$ = 118 Hz). **^{1}H NMR** (25 °C, C_6D_6, 300.13 MHz): δ = 0.04 (s, SiCH_3, $^2J(^1H-^{29}Si)$ = 6.8 Hz $^1J(^1H-^{13}C)$ = 118 Hz). **^{1}H NMR** (25 °C, toluene-[D_8], 300.13 MHz): δ = 0.07 (s, SiCH_3, $^1J(^1H-^{29}Si)$ = 6.7 Hz, $^1J(^1H-^{13}C)$ = 118 Hz). **$^{13}C\{^1H\}$ NMR** (25 °C, CD_2Cl_2, 75.47 MHz): δ = 3.17 (s, SiCH_3, $^1J(^{13}C-^{29}Si)$ = 59.5 Hz). **$^{13}C\{^1H\}$ NMR** (25 °C, C_6D_6, 75.47 MHz): δ = 2.05 (s, SiCH_3, $^1J(^{13}C-^{29}Si)$ = 59.5 Hz). **$^{13}C\{^1H\}$ NMR** (25 °C, toluene-[D_8], 75.47 MHz): δ = 2.02 (s, SiCH_3, $^1J(^{13}C-^{29}Si)$ = 59.5 Hz). **^{17}O NMR** (25 °C, CD_2Cl_2, 67.82 MHz): δ = 43.8 (s, $\Delta\nu_{1/2}$ = 50 Hz). **^{29}Si INEPT NMR** (25 °C, CD_2Cl_2, 59.62 MHz): δ = 6.81 (dec, *Si*CH_3, $^2J(^{29}Si-^1H)$ = 6.7 Hz). **^{29}Si INEPT NMR** (25 °C, C_6D_6, 59.62 MHz): δ = 6.69 (dec, *Si*CH_3, $^2J(^{29}Si-^1H)$ = 6.7 Hz). **^{29}Si INEPT NMR** (25 °C, toluene-[D_8], 59.62 MHz): δ = 6.93 (dec, *Si*CH_3, $^1J(^1H-^{29}Si)$ = 6.7 Hz) **IR** (ATR, 8 scans, 25 °C, cm^{-1}): 2956 (m), 2900 (w), 1575 (w), 1438 (w), 1411 (w), 1301 (w), 1251 (s), 1049 (s), 835 (s), 819 (s), 754 (s), 686 (m), 619 (m). **Raman** (473 nm, 4.5 mW, 10 s, 10 acc., 25 °C, cm^{-1}): 3095 (1), 2946 (6), 2885 (10), 2474 (1), 1391 (1), 1244 (1), 872 (1), 819 (1), 732 (1), 664 (1), 643 (2), 499 (2), 311 (1), 230 (1), 194 (1), 15 (2).

4 Synthesis of compounds

4.1 Potassium [18]crown-6 trimethylsilylsulfide – [K·[18]crown-6][SSiMe$_3$]

$$K[OCMe_3] + [18]crown\text{-}6 + S(SiMe_3)_2 \xrightarrow[-Me_3SiOCMe_3]{THF} [K \bullet [18]crown\text{-}6][SSiMe_3]$$

[18]crown-6 (100 mg, 0.4 mmol) and potassium *tert*-butoxide K[OCMe$_3$] (42 mg, 0.4 mmol) were dissolved in 5 mL of THF. Hexamethyldisilathiane (Me$_3$Si)$_2$S (68 mg, 0.4 mmol) was added *via* µL-syringe to this clear solution. The solvent was removed *in vacuo* until crystallization occurred. Single crystals suitable for X-ray structure elucidation were grown from a warm solution which was cooled slowly at ambient temperature over night. The crystals were washed three times with a small amount of *n*-pentane. The remaining colorless crystals were dried *in vacuo* at 50 °C, yielding in 130 mg (0.32 mmol, 80 %) of [K·[18]crown-6][SSiMe$_3$].

C$_{15}$H$_{33}$KO$_6$SSi (408.67 g/mol): **mp**. 215 °C. **EA** calc. (found), %: C, 44.08 (43.48); H, 8.14 (7.53); S, 7.85 (7.43). **^{1}H NMR** (25°C, THF-[D$_8$], 300.13 MHz): δ = 0.02 (s, 9H, SiC*H*$_3$, $^1J(^1H\text{-}^{13}C)$ = 116.4 Hz, $^2J(^1H\text{-}^{29}Si)$ = 6.4 Hz), 3.65 (s, 24H, C*H*$_2$$^{[18\text{-crown-6}]}$, $^1J(^1H\text{-}^{13}C)$ = 141.2 Hz). **^{13}C{^{1}H} NMR** (25°C, THF-[D$_8$], 75.47 MHz): δ = 9.39 (s, Si*C*H$_3$), 71.01 (s, *C*H$_2$$^{[18\text{-crown-6}]}$). **^{29}Si INEPT NMR** (25°C, THF-[D$_8$], 59.63 MHz): δ = –0.86 (dec, *Si*CH$_3$). **IR** (ATR, 16 scans, 25°C, cm^{-1}): 2937 (w), 2894 (w), 2871 (w), 2823 (w), 1631 (w), 1475 (w), 1457 (w), 1436 (w), 1365 (w), 1348 (m), 1303 (w), 1282 (w), 1236 (m), 1228 (m), 1137 (m), 1097 (s), 983 (w), 962 (s), 873 (w), 837 (m), 815 (s), 738 (m), 661 (m), 640 (s), 590 (w), 528 (m). **Raman** (473 nm, 5 mW, 20 s, 10 scans, 25°C, cm^{-1}): 2960 (9), 2943 (9), 2900 (9), 2877 (10), 2809 (2), 2722 (1), 1472 (3), 1449 (1), 1408 (1), 1364 (1), 1270 (3), 1234 (2), 1134 (1), 1108 (1), 1079 (2), 1029 (1), 912 (3), 870 (3), 829 (2), 806 (1), 746 (1), 659 (1), 642 (1), 548 (1), 508 (7), 363 (1), 306 (1), 278 (1), 242 (1), 197 (2). **ESI$^+$** (M$_{calc}$, (M$_{found}$)): 302.12045 (303.12059) [K·[18]crown−6]$^+$. **ESI$^-$** (M$_{calc}$, (M$_{found}$)): 105.01997 (105.02049) [Me$_3$Si]$^-$.

4.2 Potassium [18]crown-6 trimethylsilyloxid – [K·[18]crown-6][OSiMe$_3$]

$$\mathrm{K[OCMe_3]} + \text{[18]crown-6} + \mathrm{O(SiMe_3)_2} \xrightarrow[\text{- Me}_3\text{SiOCMe}_3]{\text{THF}} \text{[K [18]crown-6][OSiMe}_3\text{]}$$

[18]crown-6 (395 mg, 1.5 mmol) and potassium *tert*-butoxide K[OCMe$_3$] (168 mg, 1.5 mmol) were dissolved in 5 mL of THF. Hexamethyldisiloxan (Me$_3$Si)$_2$O (243 mg, 1.5 mmol) was added *via* µL-syringe to this clear solution. The solvent was removed *in vacuo* until crystallization occurred. Single crystals suitable for X-ray structure elucidation were grown from a warm solution which was cooled slowly at ambient temperature over night. The crystals were washed three times with a small amount of *n*-pentane. The remaining colorless crystals were dried *in vacuo* at 50 °C, yielding in 500 mg (1.3 mmol, 85 %) of [K·[18]crown-6][OSiMe$_3$].

$C_{15}H_{33}KO_7Si$ (392.41 g/mol): **mp**. 108 °C. **EA** $C_{15}H_{33}KO_7Si$ calc. (found) for **[K·[18]crown-6]2$^-$**·H_2O, %: C, 44.08 (44.19); H, 8.14 (8.62). **^{1}H NMR** (25°C, THF-[D$_8$], 300.13 MHz): δ = −0.15 (s, 9H, SiC*H*$_3$, $^1J(^1\text{H-}^{13}\text{C})$ = 115.2 Hz, $^2J(^1\text{H-}^{29}\text{Si})$ = 6.2 Hz), 3.61 (s, 24H, C*H*$_2^{[18\text{-crown-}6]}$, $^1J(^1\text{H-}^{13}\text{C})$ = 140.8 Hz). **^{13}C{^{1}H} NMR** (25°C, THF-[D$_8$], 75.47 MHz): δ = 5.10 (s, Si*C*H$_3$), 71.26 (s, *C*H$_2^{[18\text{-crown-}6]}$). **^{17}O NMR** (25°C, THF-[D$_8$], 33.91 MHz): not observed. **^{29}Si INEPT NMR** (25°C, THF-[D$_8$], 59.63 MHz): δ = –15.83 (dec, *Si*CH$_3$). **IR** (ATR, 32 scans, 25°C, cm^{-1}): 406 (w), 423 (w), 464 (w), 536 (w), 569 (w), 596 (w), 643 (w), 668 (w), 686 (w), 728 (w), 756 (w), 808 (m), 833 (m), 857 (m), 880 (m), 944 (m), 963 (m), 989 (m), 1026 (m), 1041 (m), 1057 (m), 1101 (s), 1239 (m), 1294 (w), 1334 (w), 1352 (m), 1393 (m), 1610 (w), 1676 (w), 1750 (w), 2861 (w), 2877 (w), 2894 (w), 2943 (w). **Raman** (633 nm, 8 mW, 20 s, 20 scans, 25°C, cm^{-1}): 78 (2), 174 (1), 217 (2), 280 (2), 314 (1), 326 (1), 370 (1), 547 (1), 597 (7), 645 (1), 730 (1), 806 (1), 830 (2), 873 (7), 990 (1), 1031 (0), 1069 (1), 1083 (1), 1111 (1), 1138 (2), 1148 (2), 1218 (1), 1234 (1), 1247 (2), 1277 (2), 1293 (1), 1368 (1), 1411 (1), 1456 (1), 1472 (3), 2687 (1), 2693 (1), 2706 (1), 2735 (1), 2755 (1), 2793 (1), 2814 (3), 2822 (3), 2851 (5), 2898 (10), 2948 (4), 3017 (1), 3020 (1).

4.3 Synthesis and characterization of tris(trimethylsilyl)sulfonium *closo*-7,8,9,10,11,12-hexachloropentahydroundecaborate – $[(Me_3Si)_3S][CHB_{11}H_5Cl_6]$

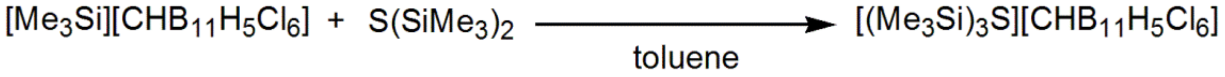

To a stirred suspension of trimethylsilylium *closo*-7,8,9,10,11,12-hexachloropentahydro-undecaborate $[Me_3Si][CHB_{11}H_5Cl_6]$ (84 mg, 0.2 mmol) in 1.5 mL toluene hexamethyl-disilathiane $(Me_3Si)_2S$ is added quickly via syringe. Slight warming (50 °C) led to a typical biphasic system. Single crystals suitable for X-ray structure elucidation were grown from this solution over night by slow cooling to room temperature. The supernatant was removed by 70 °C syringe and the colorless crystals were washed with a small amount of *n*-pentane and dried *in vacuo* at 60 °C, yielding in 82 mg (0.14 mmol, 68 %) of tris(trimethylsilyl)sulfonium *closo*- 7, 8, 9, 10, 11, 12- hexachloropentahydroundecaborate $[(Me_3Si)_3S][CHB_{11}H_5Cl_6]$.

$C_{10}H_{33}B_{11}Cl_6SSi_3$ (601.33 g/mol): **mp**. 169 °C (dec.). **EA** calc. (found), %: C, 19.97 (19.88); H, 5.53 (5.29); S, 5.33 (5.21). **^{1}H NMR** (25 °C, CD_2Cl_2, 300.13 MHz): δ = −0.42 (s, 27H, SiCH_3, $^2J(^1H-^{29}Si)$ = 6.9, $^1J(^1H-^{13}C)$ = 120.8 Hz), 0.92-3.21 (m, 6H, C*H*/B*H*). **^{11}B NMR** (25°C, CD_2Cl_2, 96.29 Hz): δ = −23.25 (d, B^{2-6}H, $^1J(^{11}B-^1H)$ = 164 Hz), −6.04 (s, B^{7-11}Cl), 0.52 (s, B^{12}Cl). **^{13}C{^{1}H} NMR** (25 °C, CD_2Cl_2, 75.47 MHz): δ = 3.47 (s, Si*C*H_3, $^1J(^{13}C-^{29}Si)$ = 57.6 Hz), 32.84 (b, *C*H). **^{29}Si INEPT NMR** (25 °C, CD_2Cl_2, 59.62 MHz): δ = 31.66 (dec, *Si*CH_3, $^2J(^{29}Si-^1H)$ = 6.9 Hz). **IR** (ATR, 8 scans, 25 °C, cm^{-1}): 3056 (w), 3025 (w), 2958 (w), 2906 (w), 2601 (m), 2472 (w), 1602(w), 1494 (w), 1462 (w), 1455 (w), 1415 (w), 1382 (w), 1317 (w), 1259 (m), 1178 (w), 1132 (m), 1078 (w), 1031 (m), 1016 (s), 989 (m), 941 (m), 910 (w), 862 (s), 810 (s), 767 (m), 748 (m), 734 (m), 696 (m), 649 (m), 621 (m), 570 (w).

4.4 Synthesis and characterization of tris(trimethylsilyl)sulfonium *closo*-2,3,4,5,6,7,8,9,10,11,12-undecachloroundecaborate – $[(Me_3Si)_3S][CHB_{11}Cl_{11}]$

$$[Me_3Si][CHB_{11}Cl_{11}] + S(SiMe_3)_2 \xrightarrow{\text{toluene}} [(Me_3Si)_3S][CHB_{11}Cl_{11}]$$

The reaction of trimethylsilylium *closo*-2,3,4,5,6,7,8,9,10,11,12-undecachloroundecaborate $[Me_3Si][CHB_{11}Cl_{11}]$ with hexamethyldisilathiane $(Me_3Si)_2S$ shows the same reactivity like the trimethylsilylium *closo*-7,8,9,10,11,12-hexachloropentahydroundecaborate $[Me_3Si][CHB_{11}H_5Cl_6]$ and give the identical results and products (For further information see ESI section 4.2).

^{1}H NMR (25 °C, CD_2Cl_2, 300.13 MHz): δ = −0.42 (s, 27H, SiC*H*$_3$, $^{2}J(^{1}H\text{-}^{29}Si)$ = 6.9, $^{1}J(^{1}H\text{-}^{13}C)$ = 120.8 Hz). **^{11}B NMR** (25 °C, DMSO-[D_6], 96.29 MHz): δ = –13.0 (s, *B*$_{2\text{-}6}$Cl), –9.9 (s, *B*$_{7\text{-}11}$Cl), –2.8 (s, *B*$_{12}$Cl). **^{29}Si INEPT NMR** (25 °C, CD_2Cl_2, 59.62 MHz): δ = 31.66 (dec, *Si*CH$_3$, $^{2}J(^{29}Si\text{-}^{1}H)$ = 6.9 Hz).

4.5 Synthesis and characterization of tris(trimethylsilyl)sulfonium tetrakis(pentafluorophenyl)borate – $[(Me_3Si)_3S][B(C_6F_5)_4]$

$$[(Me_3Si)_2H][B(C_6F_5)_4] + S(SiMe_3)_2 \xrightarrow[-Me_3SiH]{\text{toluene, -80°C} \rightarrow \text{rt}} [(Me_3Si)_3S][B(C_6F_5)_4]$$

The trimethylsilane adduct of trimethylsilylium tetrakis(pentafluorophenyl)borate $[(Me_3Si)_2H][B(C_6F_5)_4]$ (659 mg, 0.8 mmol) was suspended in 0.6 mL toluene. The suspension was degasified three times by a freeze-pump-thaw procedure. Hexamethyldisilathiane $(Me_3Si)_2S$ (142 mg, 0.8 mmol) was added to this suspension at −80 °C. After warming to ambient temperatures the typical biphasic system was concentrated to a minimum. Single crystals suitable for X-ray structure elucidation were grown from this solution over night. They were unfortunately heavily disordered. The supernatant was removed by syringe and the colorless crystals were washed with a small amount of cold toluene and dried *in vacuo* at 50 °C, yielding in 340 mg (0.37 mmol, 46 %) of $[(Me_3Si)_3S][B(C_6F_5)_4]$.

$C_{33}H_{27}BF_{20}SSi_3$ (930.67 g/mol): **mp**. 158 °C (dec.). **EA** calc. (found), %: C, 42.59 (42.24); H, 2.92 (2.18). **^{1}H NMR** (25 °C, 1,2-DCB, ref. ext. acetone-[D$_6$], 300.13 MHz): δ = −1.02 (s, 27H, SiC*H*$_3$, $^2J(^1\text{H}-^{29}\text{Si})$ = 6.5, $^1J(^1\text{H}-^{13}\text{C})$ = 123 Hz), 5.52 (m, 2H, *o*-C*H*), 5.78 (m, 2H, *m*-C*H*). **^{1}H NMR** (25 °C, toluene, ref. ext. CD_2Cl_2, 300.13 MHz): δ = 0.01 (s, 27H, SiC*H*$_3$, $^1J(^1\text{H}-^{13}\text{C})$ = 123.1 Hz), 1.97 (s, 3H, C*H*$_3$, $^1J(^1\text{H}-^{13}\text{C})$ = 126.3 Hz), 6.73-6.93 (m, 5H, C*H*-Ph). **^{11}B NMR** (25 °C, 1,2-DCB, 96.29 Hz): δ = −17.57 (s, *B*(C$_6$F$_5$)$_4$, $\Delta\nu_{1/2}$ = 23 Hz). **^{11}B NMR** (25 °C, toluene, 96.29 Hz): δ = −16.41 (s, *B*(C$_6$F$_5$)$_4$, $\Delta\nu_{1/2}$ = 25 Hz). **^{13}C{^{1}H} NMR** (25 °C, 1,2-DCB, ref. ext. acetone-[D$_6$], 75.47 MHz): δ = −2.09 (s, Si*C*H$_3$), 124.16 (b, *ipso*-*C*^{F}), 127.09 (s, *m*-*C*H), 129.82 (s, *o*-*C*H), 131.88 (s, *ipso*-*C*^{Cl}), 135.88 (dm, *m*-*C*F, $^1J(^{13}\text{C}-^{19}\text{F})$ = 245 Hz), 137.84 (dm, *p*-*C*F, $^1J(^{13}\text{C}-^{19}\text{F})$ = 245 Hz), 148.18 (dm, *o*-*C*F, $^1J(^{13}\text{C}-^{19}\text{F})$ = 258 Hz). **^{13}C{^{1}H} NMR** (25 °C, toluene, ref. ext. CD_2Cl_2, 75.47 MHz): δ = 2.02 (s, Si*C*H$_3$, $^1J(^{13}\text{C}-^{29}\text{Si})$ = 58 Hz), 20.99 (s, *C*H$_3$), 125.13 (b, *ipso*-*C*^{F}), 125.53 (s, *p*-*C*H), 128.48 (s, *m*-*C*H), 129.29 (s, *o*-*C*H), 136.85 (dm, *m*-*C*F, $^1J(^{13}\text{C}-^{19}\text{F})$ = 247 Hz), 138.01 (s, *ipso*-*C*^{H}), 138.53 (dm, *p*-*C*F, $^1J(^{13}\text{C}-^{19}\text{F})$ = 245 Hz), 148.97 (dm, *o*-*C*F, $^1J(^{13}\text{C}-^{19}\text{F})$ = 241 Hz). **^{19}F{^{1}H} NMR** (25 °C, 1,2-DCB, 282.38 Hz): δ = −167.38 (t, *m*-C*F*, $^1J(^{19}\text{F}-^{13}\text{C})$ = 245 Hz), −163.60 (t, *p*-C*F*, $^1J(^{19}\text{F}-^{13}\text{C})$ = 245 Hz), −132.99 (d, *o*-C*F*, $^1J(^{19}\text{F}-^{13}\text{C})$ = 258 Hz). **^{19}F{^{1}H} NMR** (25 °C, toluene, 282.38 MHz): δ = −167.07 (t, *m*-C*F*, $^1J(^{19}\text{F}-^{13}\text{C})$ = 245 Hz), −163.35 (t, *p*-C*F*, $^1J(^{19}\text{F}-^{13}\text{C})$ = 246 Hz), −132.08 (d, *o*-C*F*, $^1J(^{19}\text{F}-^{13}\text{C})$ = 245 Hz). **^{29}Si**

INEPT NMR (25 °C, 1,2-DCB, 59.63 MHz): δ = 38.14 (dec, *Si*CH_3, $^{2}J(^{29}Si-^{1}H)$ = 6.5 Hz). **$^{29}Si\{^{1}H\}$ NMR** (25 °C, toluene, 59.63 MHz): δ = 39.29 (s, *Si*CH_3). **Raman** (764 nm, 33 mW, 80 s, 3 acc., 7200 s photobleach, 25 °C, cm^{-1}): 2972 (1), 2912 (2), 1644 (1), 1417 (1), 1375 (1), 1269 (1), 821 (2), 768 (1), 756 (1), 698 (1), 683 (1), 634 (7), 583 (10), 575 (2), 490 (5), 473 (4), 447 (5), 421 (4), 393 (4), 356 (2), 283 (1), 275 (1), 245 (2), 240 (2). **MS** (CI^+, m/z (%)): 73 (1) $[Me_3Si]^+$, 91 (3) $[Me_3SiF - H]^+$, 149 (16) $[Me_3SiSSiMe]^+$, 163 (100) $[Me_3SiSSiMe_2]^+$, 221 (7) $[(Me_3Si)_2SSiMe]^+$, 237 (15) $[(Me_3Si)_2SSiMe_2]^+$, 512 (10) $[B(C_6F_5)_3]^+$. **MS** (ESI, calc. m/z, (found m/z)): $[B(C_6F_5)_4]^-$ 678.97791 (678.98935).

4.6 Synthesis and characterization of bis(trimethylsilyl)-mono-(dimethylsilyl)oxonium tetrakis(pentafluorophenyl)borate – [$T_2(Me_2(H)Si)O$][$B(C_6F_5)_4$][$B(C_6F_5)_4$]

$$[(Me_3Si)_2H][B(C_6F_5)_4] + O(SiMe_3)_2 \xrightarrow[\text{-}Me_3SiH]{\text{toluene, -80°C -> RT}} [(Me_3Si)_2O(SiMe_2H)][B(C_6F_5)_4] + SiMe_4$$

The trimethylsilane adduct of trimethylsilylium tetrakis(pentafluorophenyl)borate [$(Me_3Si)_2H$][$B(C_6F_5)_4$] (172 mg, 0.2 mmol) was suspended in 2 mL toluene. The suspension was degasified three times by a freeze-pump-thaw procedure. Hexamethyldisiloxane $(Me_3Si)_2O$ (34 mg, 0.2 mmol) was added to this suspension at −80 °C via µL syrringe. After warming to ambient temperatures the typical biphasic system was slightly concentrated. Single crystals suitable for X-ray structure elucidation were grown from this solution by slow cooling to −20 °C over night. They were unfortunately heavily disordered. The supernatant was removed by syringe and the colorless crystals were washed with a small amount of cold toluene and dried *in vacuo* at −20 °C, yielding in 82 mg (0.08 mmol, 43 %) of **[$T_2(Me_2(H)Si)O$][$B(C_6F_5)_4$]**.

$C_{32}H_{25}BF_{20}OSi_3$ (900.58 g/mol): **mp**. >90 °C (dec.). **EA** calc. (found) for **[$T_2(Me_2(H)Si)O$][$B(C_6F_5)_4$]**·toluene, %: C, 47.19 (47.25); H, 3.35 (3.41)$^+$. **^{1}H NMR** (25 °C, CD_2Cl_2, 300.13 MHz): δ = 0.43 (s, 6H, SiC*H*$_3$, $^2J(^1H\text{-}^{29}Si)$ = 7.5), δ = 0.65 (s, 9H, SiC*H*$_3$, $^2J(^1H\text{-}^{29}Si)$ = 7.5), 6.08 (s, 1H, Si*H*). **^{11}B NMR** (25 °C, CD_2Cl_2, 96.29 Hz): δ = −16.24 (s, *B*$(C_6F_5)_4$, $\Delta\nu_{1/2}$ = 24 Hz). **^{13}C{^{1}H} NMR** (25 °C, CD_2Cl_2, 75.47 MHz): δ = 1.28 (s, HSi(*C*H_3)$_2$), 3.47 (s, Si(*C*H_3)$_3$), 125.13 (b, *ipso*-*C*^{F}), 136.85 (dm, *m*-*C*F, $^1J(^{13}C\text{-}^{19}F)$ = 247 Hz), 138.53 (dm, *p*-*C*F, $^1J(^{13}C\text{-}^{19}F)$ = 245 Hz), 148.97 (dm, *o*-*C*F, $^1J(^{13}C\text{-}^{19}F)$ = 241 Hz). **^{19}F{^{1}H} NMR** (25 °C, toluene, 282.38 MHz): δ = −167.07 (t, *m*-C*F*, $^1J(^{19}F\text{-}^{13}C)$ = 245 Hz), −163.35 (t, *p*-C*F*, $^1J(^{19}F\text{-}^{13}C)$ = 246 Hz), −132.08 (d, *o*-C*F*, $^1J(^{19}F\text{-}^{13}C)$ = 245 Hz). **^{29}Si INEPT NMR** in the following experiments. **MS** (CI^+, m/z (%)): 149 (29) [$(Me_3Si)O(H)SiMe_2H$]$^+$, 163 (85) [$(Me_3Si)_2OH$]$^+$, 221 (19) [$(Me_3Si)_2OSiMe_2H$]$^+$, 297 (100), 371 (28).

$^+$ Note EA was calculated for pure **[$T_2(Me_2(H)Si)O$][$B(C_6F_5)_4$]**·toluene; according to X-Ray analysis, **[$T_2(Me_2(H)Si)O$][$B(C_6F_5)_4$]**·toluene co-crystallizes with small amount of **[$Me_3Si{-}F{-}SiMe_3$][$B(C_6F_5)_4$]**.

* Rapid hydrolysis during sample preparation. Prolonged exposition to ambient temperatures and moisture lead to decomposition of **[T_2(Me_2(H)Si)O][B(C_6F_5)$_4$]**.

The crystals are rather instable, low soluble in aromatic solvents and decomposed in solution. As a result, different product mixtures are obtained. The data given above are the best results of many attempts.

Experiment 1.1 ($[Me_3Si{-}H{-}SiMe_3][B(C_6F_5)_4]$ + $(Me_3Si)_2O$ + Toluene):

The isolated crystals were dissolved in toluene / 1,2-DCB, at −20 °C.

^{29}Si INEPT NMR (−20 °C, toluene-[D$_8$], 49.69 MHz): δ = 1.3 (sep, $^2J(^{29}Si\text{-}^1H)$ = 8.0 Hz), 14.4 (sep, $^2J(^{29}Si\text{-}^1H)$ = 6.5 Hz), 31.5 (dsep, $^1J(^{29}Si\text{-}^1H)$ = 230 Hz, $^2J(^{29}Si\text{-}^1H)$ = 7.3 Hz), 48.2 (m, $^2J(^{29}Si\text{-}^1H)$ = 7.3 Hz), 53.8 (m, $^2J(^{29}Si\text{-}^1H)$ = 7.6 Hz).

Figure S2: *NMR-spectra of experiment 1.1 in toluene-[D$_8$].*

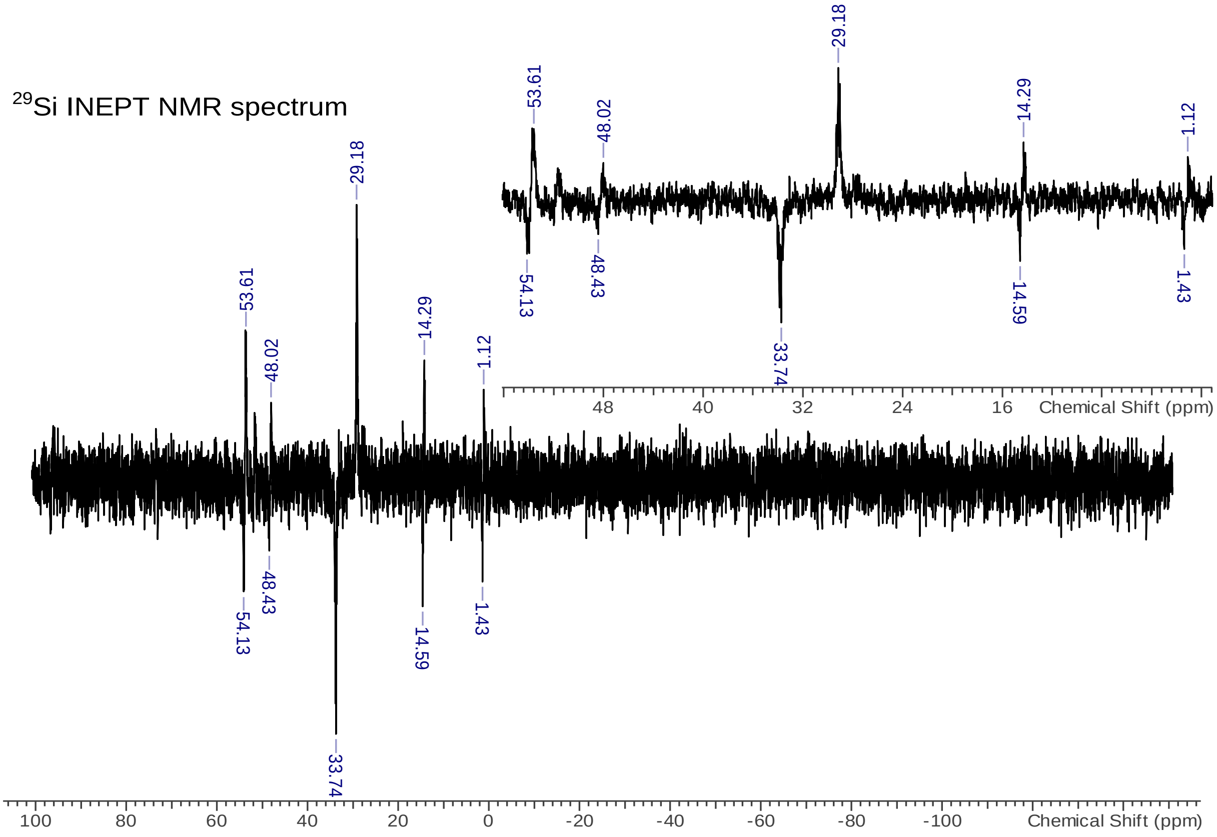

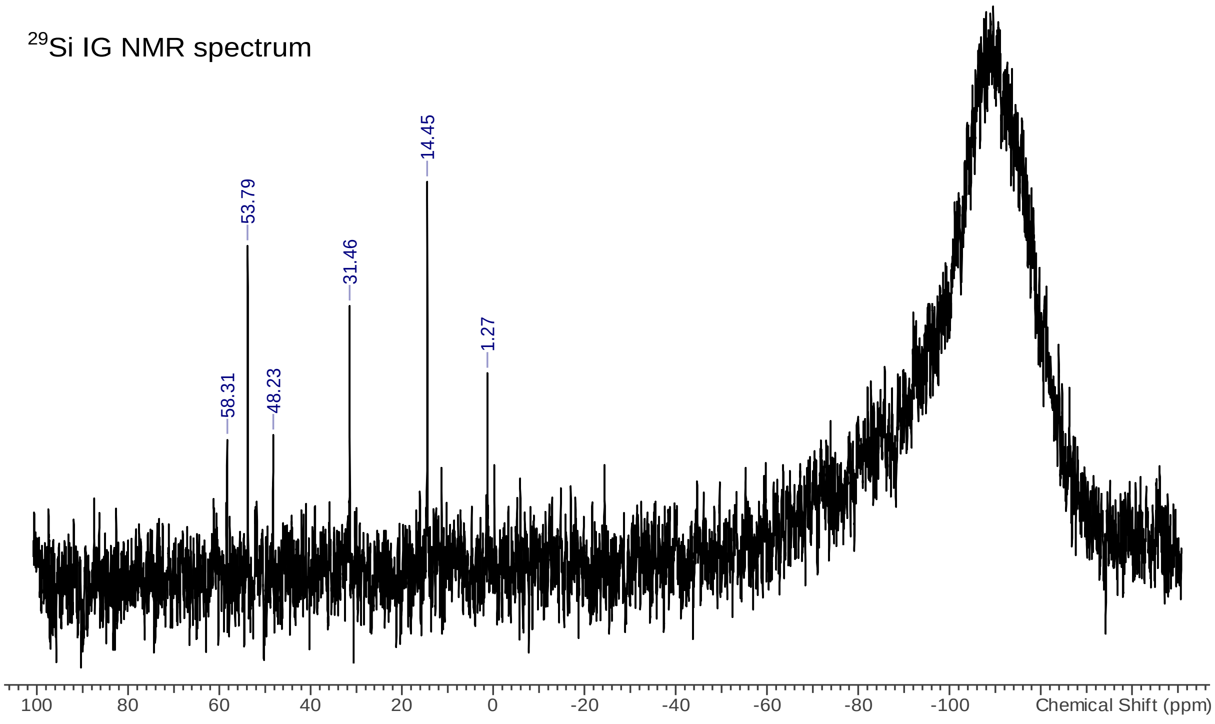
29Si IG NMR spectrum
58.31
53.79
48.23
31.46
14.45
1.27
100
80
60
40
20
0
-20
-40
-60
-80
-100
Chemical Shift (ppm)

Experiment 1.2 ($[Me_3Si{-}H{-}SiMe_3][B(C_6F_5)_4]$ + $(Me_3Si)_2O$ + Toluene):

The isolated crystals were dissolved in toluene / 1,2-DCB, at 25 °C.

^{29}Si INEPT NMR (25 °C, toluene-[D_8], 49.69 MHz): δ = 1.3 (sep, $^2J(^{29}Si{-}^1H)$ = 7.4 Hz), 11.0 (m, $^2J(^{29}Si{-}^1H)$ = 5.4 Hz), 12.2 (m, $^2J(^{29}Si{-}^1H)$ = 6.8 Hz), 13.9 (sep, $^2J(^{29}Si{-}^1H)$ = 7.4 Hz), 14.5 (sep, $^2J(^{29}Si{-}^1H)$ = 7.4 Hz), 15.1 (m, $^2J(^{29}Si{-}^1H)$ = 7.4 Hz, $^3J(^{29}Si{-}^1H)$ = 2.7 Hz), 18.2 (m, $^2J(^{29}Si{-}^1H)$ = 6.8 Hz), 19.3 (dec, $^2J(^{29}Si{-}^1H)$ = 6.8 Hz), 20.7 (m, $^2J(^{29}Si{-}^1H)$ = 6.8 Hz), 31.6 (dsep, $^1J(^{29}Si{-}^1H)$ = 230 Hz, $^2J(^{29}Si{-}^1H)$ = 6.8 Hz), 48.4 (m, $^2J(^{29}Si{-}^1H)$ = 6.8 Hz), 50.8 (dec, $^2J(^{29}Si{-}^1H)$ = 6.8 Hz), 52.0 (dec, $^2J(^{29}Si{-}^1H)$ = 6.8 Hz), 54.0 (ddec, $^2J(^{29}Si{-}^1H)$ = 6.8 Hz, $^3J(^{29}Si{-}^1H)$ = 2.7 Hz).

Figure S3: *NMR-spectra of experiment 1.2 in toluene-[D_8].*

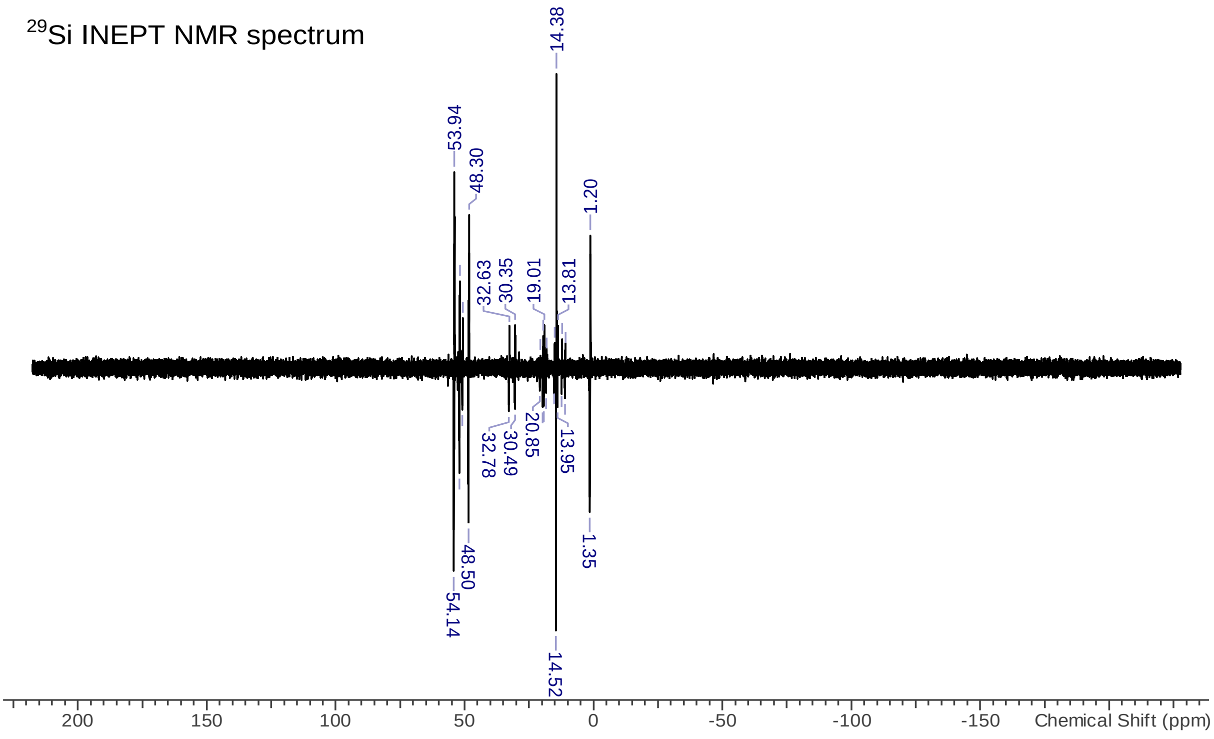

^{29}Si INEPT NMR spectrum

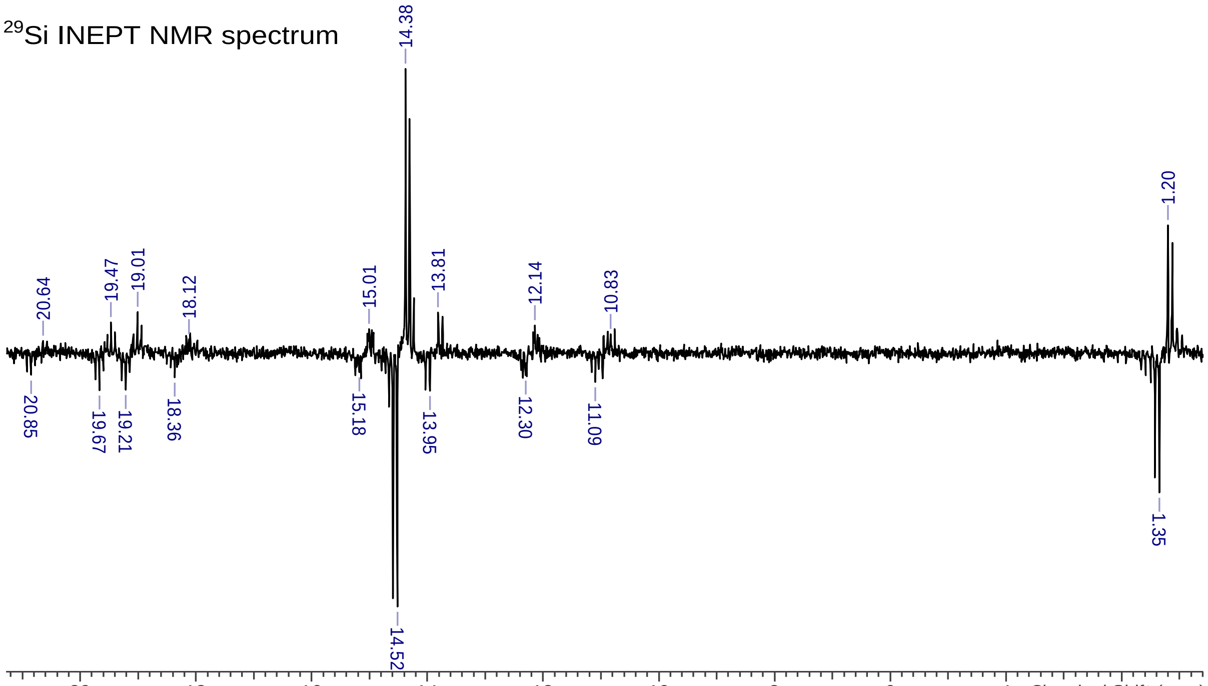

^{29}Si INEPT NMR spectrum

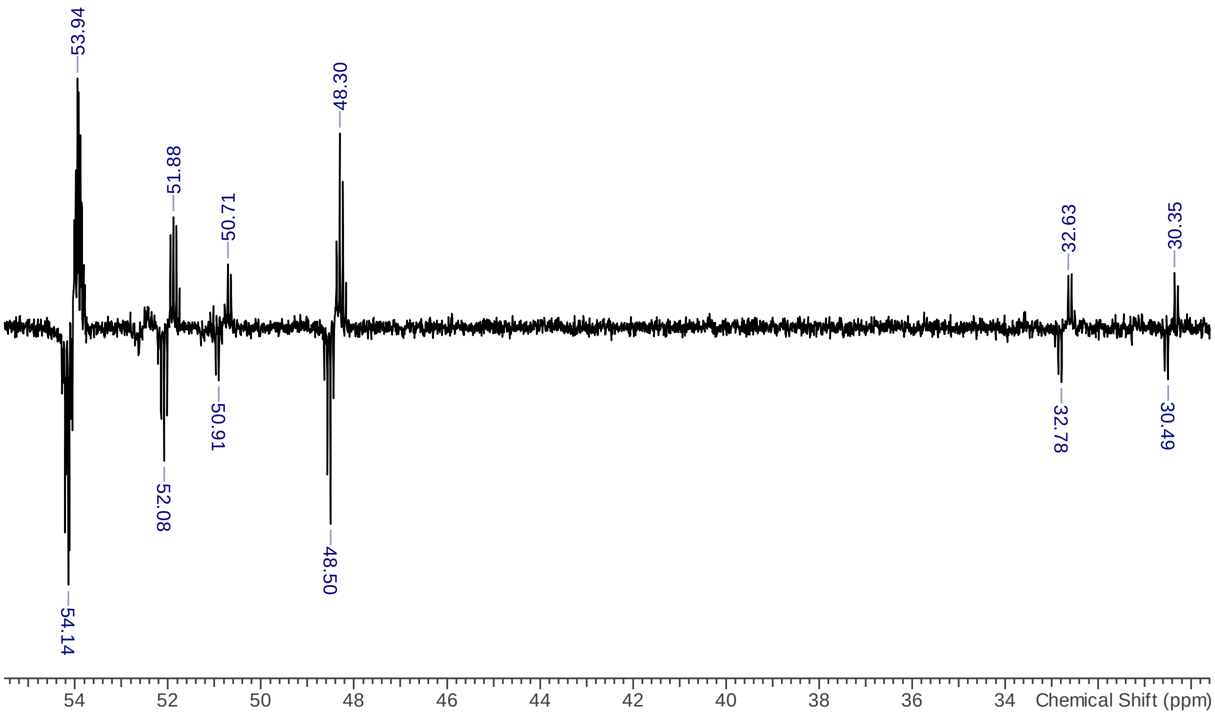

^{29}Si IG NMR spectrum

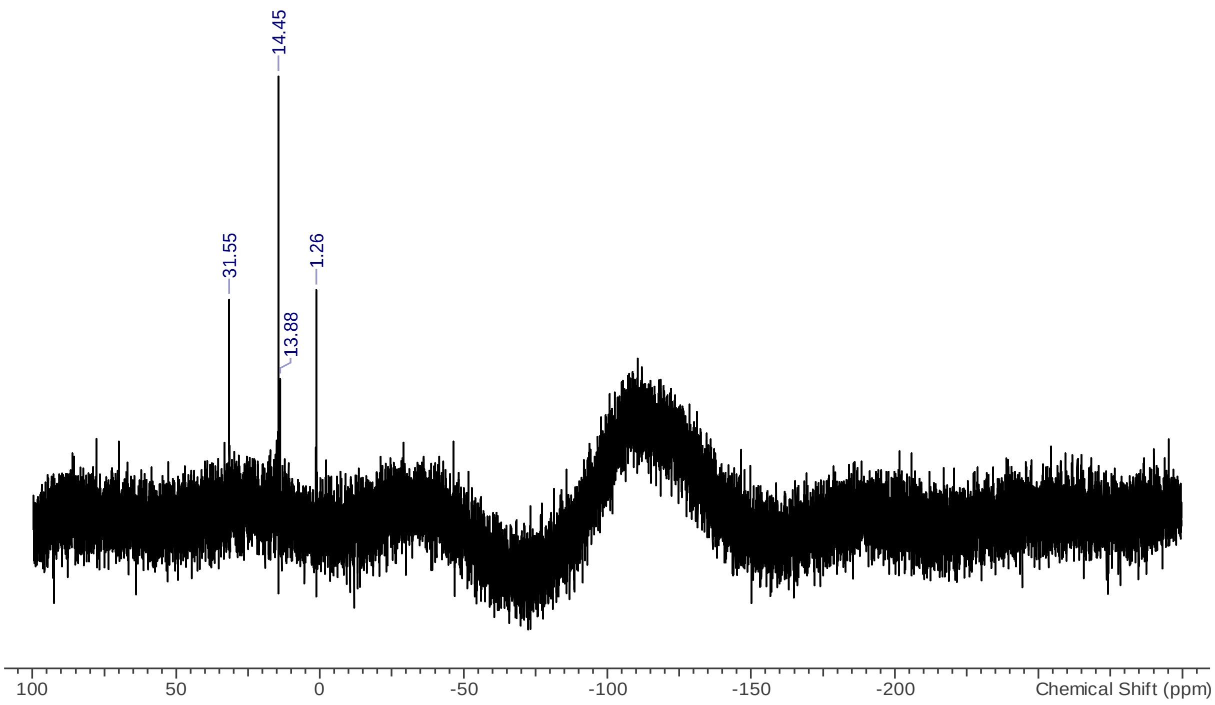

Experiment 2 ($[Me_3Si{-}H{-}SiMe_3][B(C_6F_5)_4]$ + $(Me_3Si)_2O$ + Toluene):

The isolated crystals were dissolved in CD_2Cl_2. Decomposition upon solvation, including reaction with solvent.

^{29}Si INEPT NMR (25 °C, CD_2Cl_2, 59.63 MHz): δ = −2.72 (sep, $^2J(^{29}Si\text{-}^1H)$ = 7.3 Hz), −1.05 (m, $^2J(^{29}Si\text{-}^1H)$ = 7.3 Hz), 0.30 (sep, $^2J(^{29}Si\text{-}^1H)$ = 7.3 Hz), 2.13 (sep, $^2J(^{29}Si\text{-}^1H)$ = 7.3 Hz), 6.68 (q, $^2J(^{29}Si\text{-}^1H)$ = 7.3 Hz), 12.63 (sep, $^2J(^{29}Si\text{-}^1H)$ = 7.3 Hz), 20.82 (dec, $^2J(^{29}Si\text{-}^1H)$ = 6.5 Hz), 31.66 (dec, *Si*CH_3, $^2J(^{29}Si\text{-}^1H)$ = 7.3 Hz), 59.43 (dec, *Si*CH_3, $^2J(^{29}Si\text{-}^1H)$ = 6.5 Hz).

Figure S4: *NMR-spectra of experiment 2 in CD_2Cl_2.*

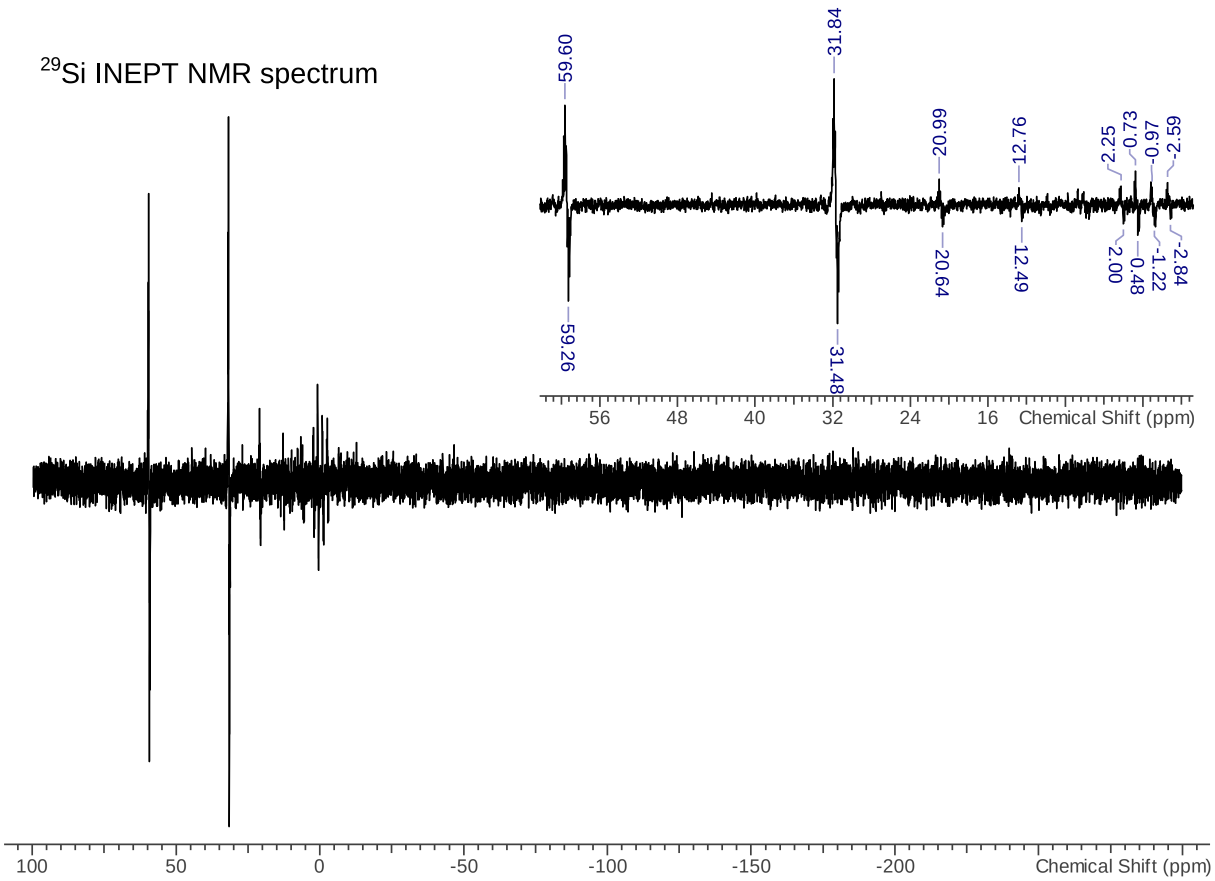

Experiment 3 ([$Me_3Si-H-SiMe_3$][$B(C_6F_5)_4$] + $(Me_3Si)_2O$ + Toluene):

The isolated crystals were suspended in toluene.

^{29}Si INEPT NMR (25 °C, toluene-[D_8], 59.63 MHz): $\delta = -0.37$ (dec, $^{2}J(^{29}Si-^{1}H) = 6.5$ Hz, Me_4Si).

Figure S5: *NMR-spectra of experiment 3 in toluene-[D_8].*

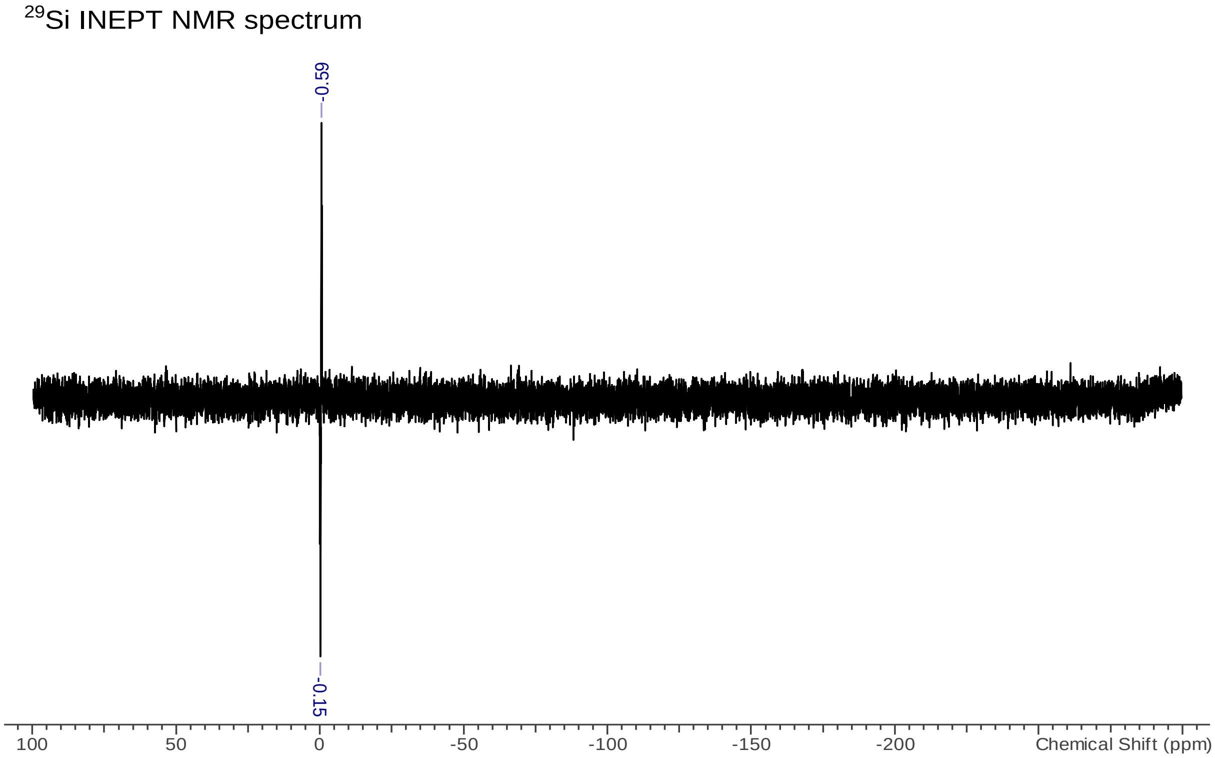

Experiment 4 ([Me_3Si-H-$SiMe_3$][$B(C_6F_5)_4$] + $(Me_3Si)_2O$ + Toluene):

The isolated crystals were suspended in benzene.

^{29}Si IG NMR (25 °C, C_6D_6, 99.36 MHz): $\delta = -0.52$ (Me_4Si).

Figure S6: *NMR-spectra of experiment 4 in benzene-[D_6].*

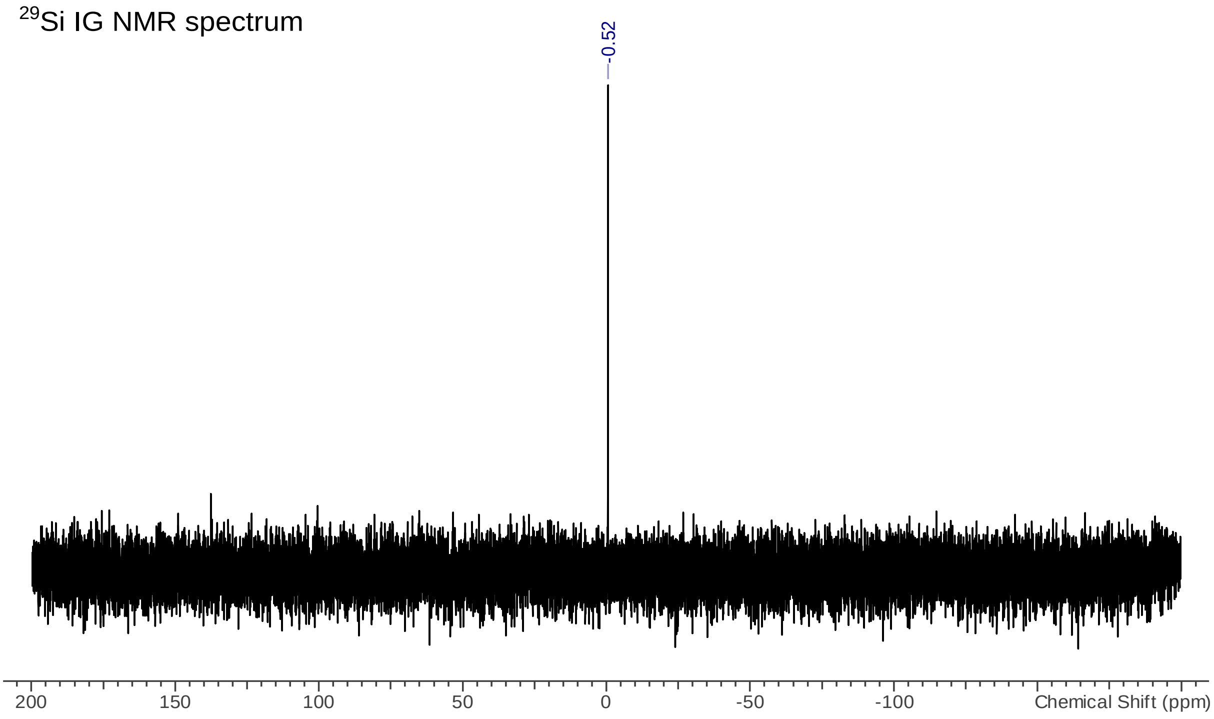

Experiment 5 (100 mg $[Ph_3C][B(C_6F_5)_4]$ + $(Me_3Si)_2O$ 0.05 mL + Me_3SiH 0.5 mL (CD_2Cl_2 in capillary)):

The staring materials are placed together in the NMR tube. NMR-tube reaction. An insoluble precipitate is formed. No formed products are removed.

^{29}Si INEPT NMR (−60 - 25 °C, 49.69 MHz): δ = −15.9 (ddec, $^{1}J(^{29}Si-^{1}H)$ = 183.1 Hz $^{2}J(^{29}Si-^{1}H)$ = 6.5 Hz; **Me_3SiH**), 0.64 (dec, $^{2}J(^{29}Si-^{1}H)$ = 6.5 Hz, **$SiMe_4$**), 7.6 (dec, $^{2}J(^{29}Si-^{1}H)$ = 5.7 Hz; **$(Me_3Si)_2O$**), 29.87 (dec, $^{2}J(^{29}Si-^{1}H)$ = 6.5 Hz).

Figure S7: *NMR-spectra of experiment 5.*

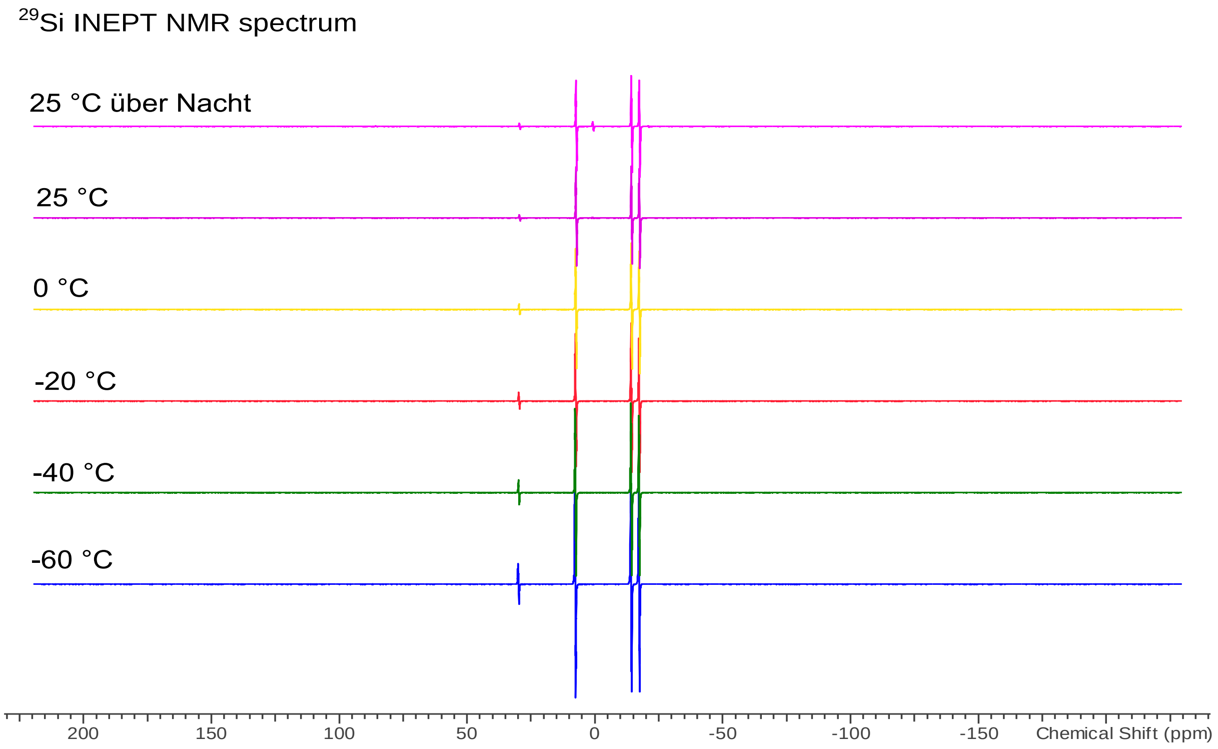

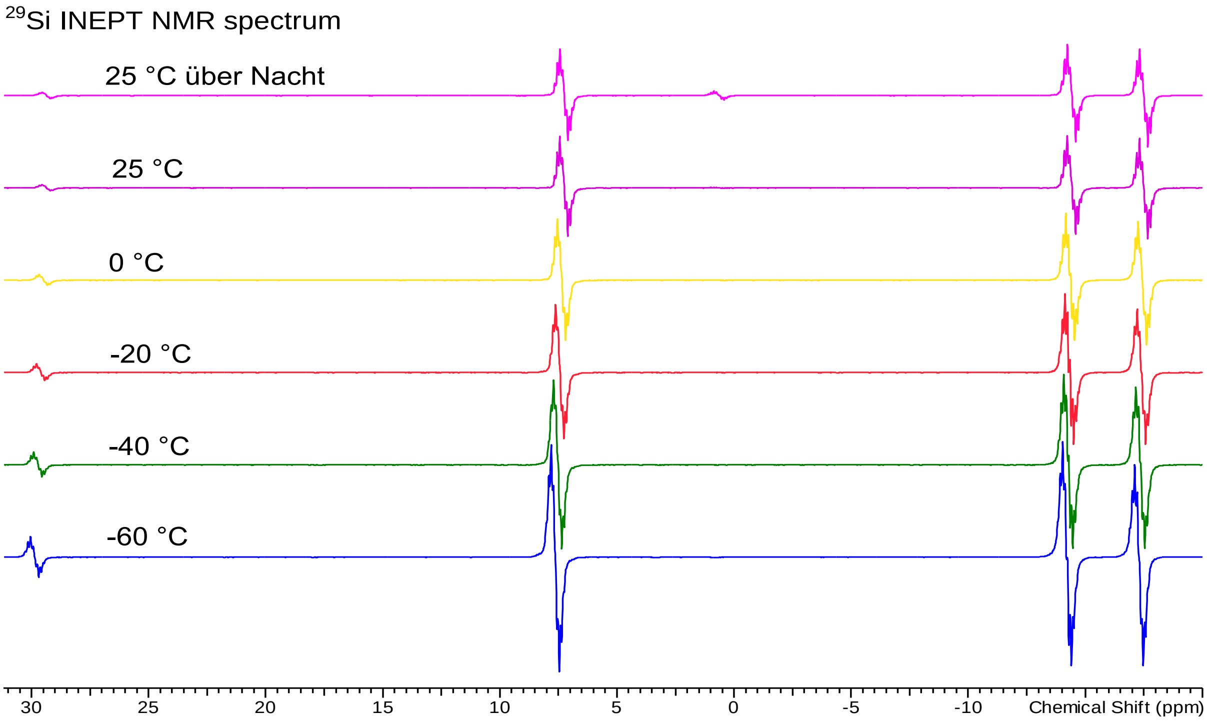

29Si INEPT NMR spectrum
25 °C über Nacht
25 °C
0 °C
-20 °C
-40 °C
-60 °C
30
25
20
15
10
5
0
-5
-10
Chemical Shift (ppm)

Experiment 6 (100 mg $[Ph_3C][B(C_6F_5)_4]$ + $(Me_3Si)_2O$ 0.05 mL + Me_3SiH 0.5 mL + CH_2Cl_2 0.1 mL (CD_2Cl_2 in capillary)): OLAH-reaction

The staring materials are placed together in the NMR tube. NMR-tube reaction. All the precipitation dissolves and decomposes. The concentration of $SiMe_4$ increases significantly. No formed products are removed.

^{29}Si INEPT NMR (−60 - 25 °C, 49.69 MHz): δ = −15.9 (ddec, $^1J(^{29}Si-^1H)$ = 183.1 Hz $^2J(^{29}Si-^1H)$ = 6.5 Hz; **Me_3SiH**), 0.64 (dec, $^2J(^{29}Si-^1H)$ = 6.5 Hz; **$SiMe_4$**), 7.6 (dec, $^2J(^{29}Si-^1H)$ = 5.7 Hz; **$(Me_3Si)_2O$**), 29.87 (dec, $^2J(^{29}Si-^1H)$ = 6.5 Hz).

Figure S8: *NMR-spectra of experiment 6.*

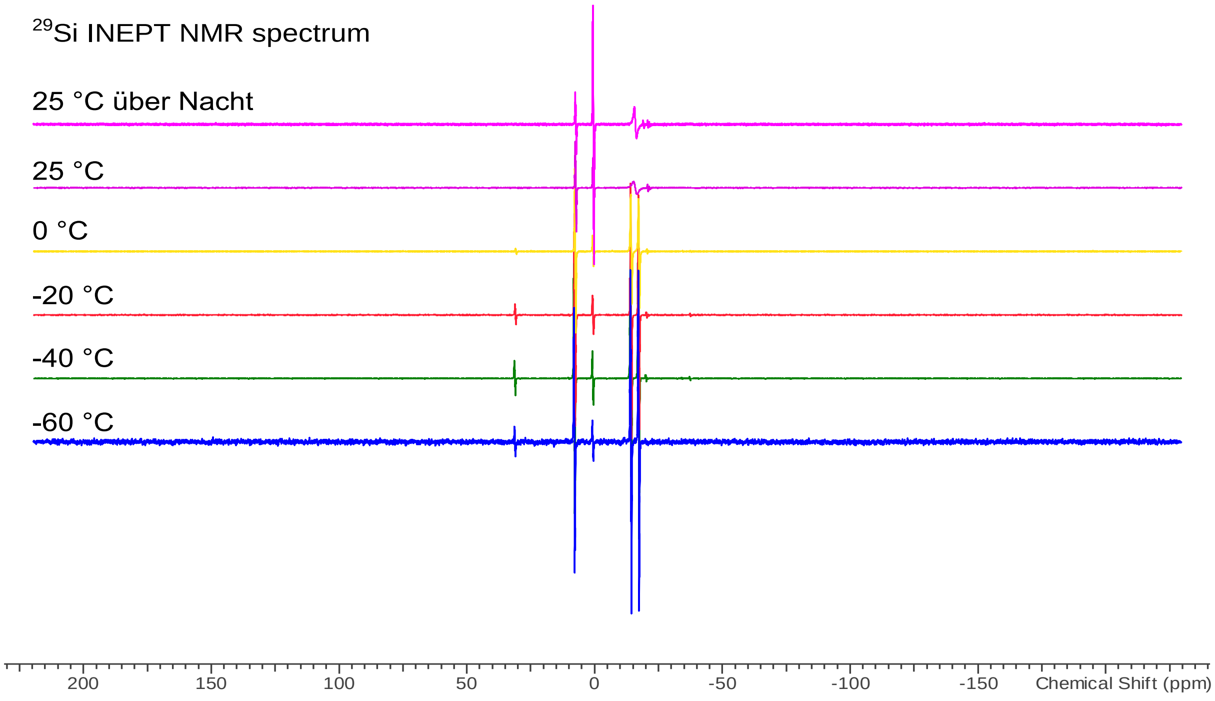

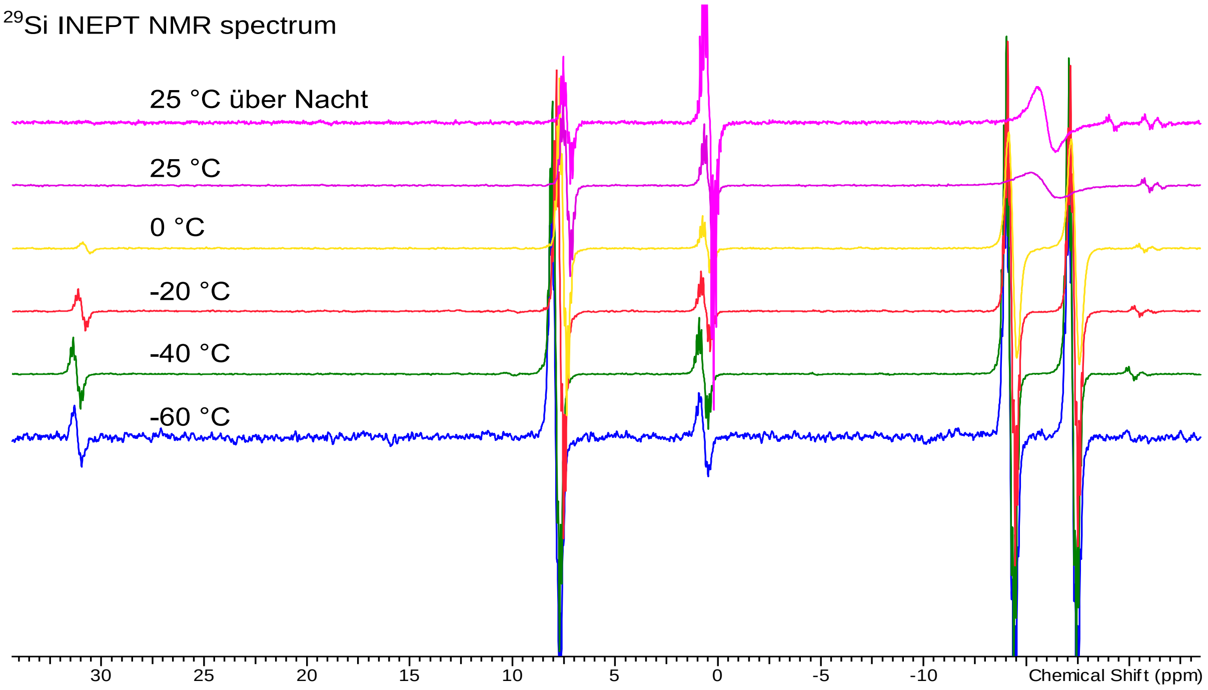

^{29}Si INEPT NMR spectrum
25 °C über Nacht
25 °C
0 °C
-20 °C
-40 °C
-60 °C
30
25
20
15
10
5
0
-5
-10
Chemical Shift (ppm)

Experiment 7 (100 mg [Ph_3C][$CHB_{11}Cl_{11}$] + ($Me_3Si)_2O$ 0.05 mL + Me_3SiH 0.5 mL (CD_2Cl_2 in capillary)):

The staring materials are placed together in the NMR tube. NMR-tube reaction. An insoluble precipitate is formed and no formation of $SiMe_4$ can be observed. No formed products are removed.

^{29}Si INEPT NMR (−60 - 25 °C, 49.69 MHz): δ = −15.9 (ddec, $^1J(^{29}Si\text{-}^1H)$ = 183.1 Hz $^2J(^{29}Si\text{-}^1H)$ = 6.5 Hz; **Me_3SiH**), 7.6 (dec, $^2J(^{29}Si\text{-}^1H)$ = 5.7 Hz; **($Me_3Si)_2O$**), 29.87 (dec, $^2J(^{29}Si\text{-}^1H)$ = 6.5 Hz).

Figure S9: *NMR-spectra of experiment 7.*

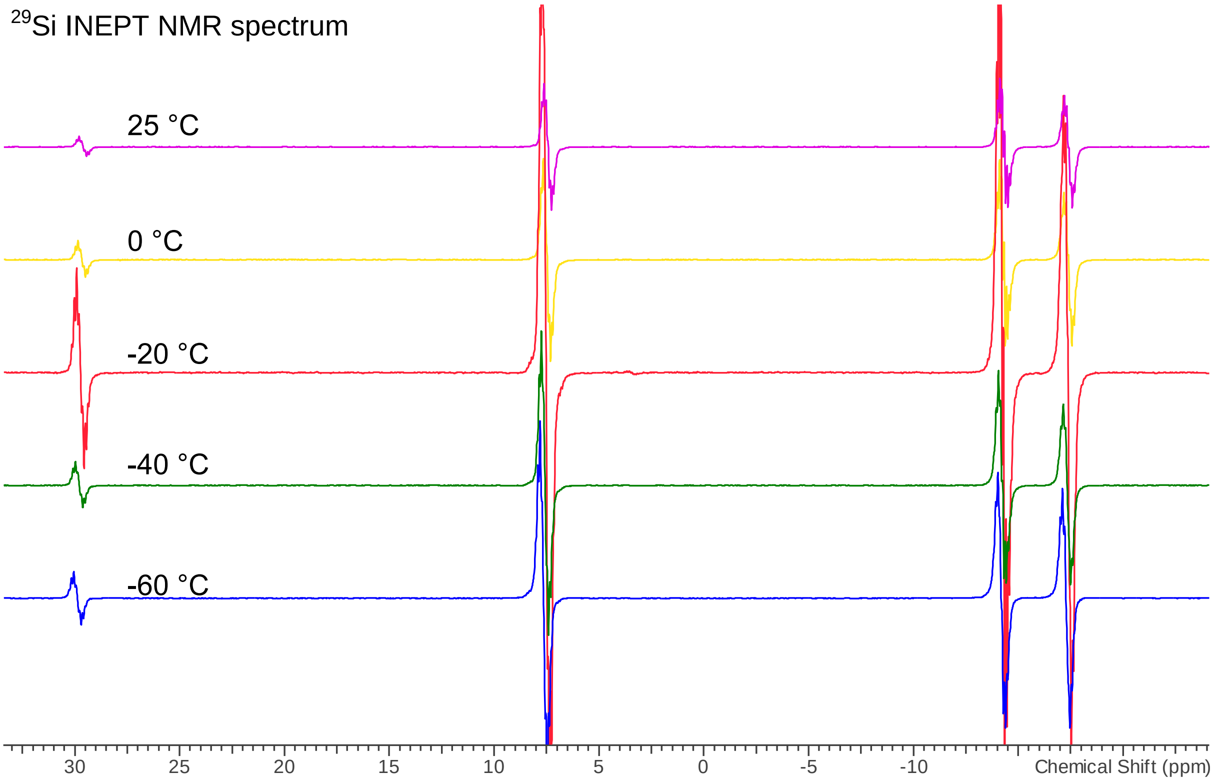

Experiment 8 (70 mg [Me_3Si][$CHB_{11}Cl_{11}$] + $(Me_3Si)_2O$ 0.1 mL + Toluene 0.5 mL):

The staring materials are placed together in the NMR tube. NMR-tube reaction. An insoluble precipitate is formed and no other Me_3Si-species can be detected out of $(Me_3Si)_2O$. No formed products are removed.

^{29}Si INEPT NMR (−60 - 25 °C, 49.69 MHz): δ = 7.6 (dec, $^{2}J(^{29}Si\text{-}^{1}H)$ = 5.7 Hz; (**$Me_3Si)_2O$**).

Figure S10: *NMR-spectra of experiment 8.*

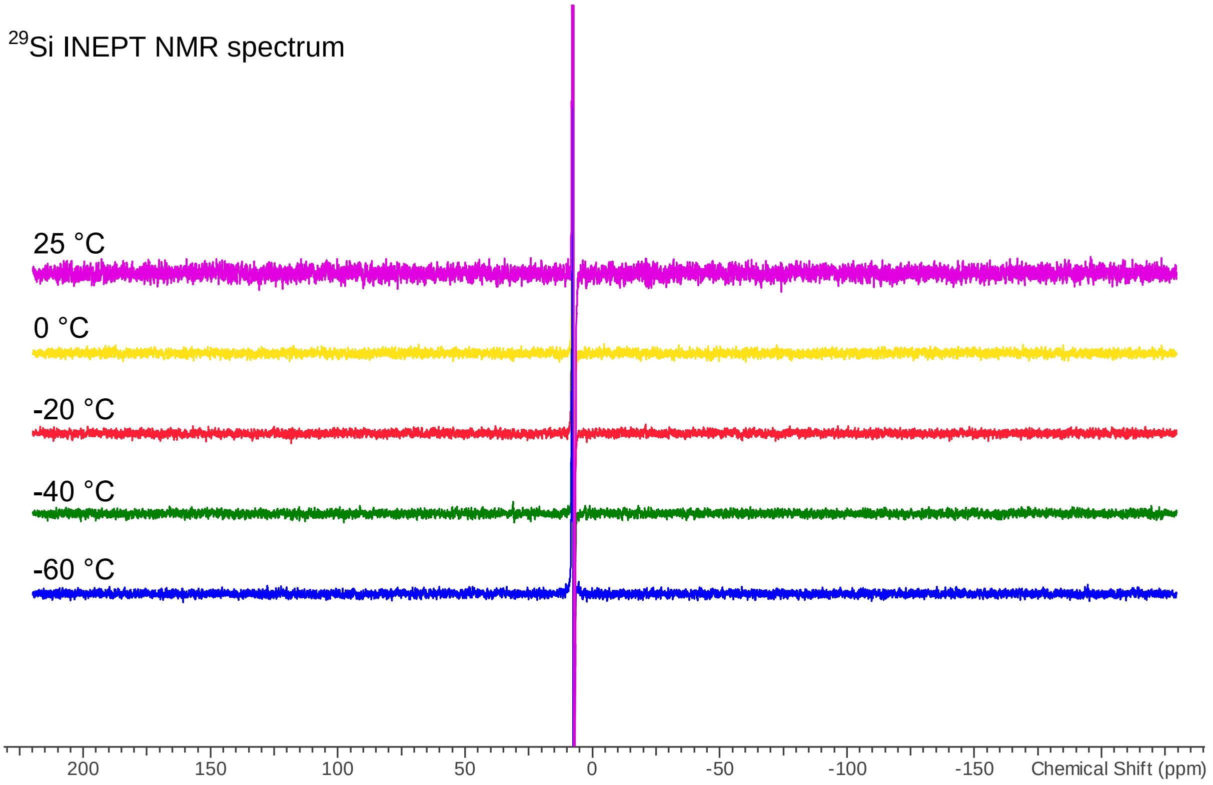

Experiment 9 (70 mg $[Me_3Si][CHB_{11}Cl_{11}]$ + $(Me_3Si)_2O$ 0.1 mL + Toluene 0.5 mL + CH_2Cl_2 0.1 mL):

The staring materials are placed together in the NMR tube. NMR-tube reaction. All the precipitation dissolves and decomposes. The concentration of $SiMe_4$ increases significantly. No formed products are removed.

^{29}Si INEPT NMR (−60 - 25 °C, 49.69 MHz): δ = −21.4, −19.0, 0.64 (dec, $^2J(^{29}Si\text{-}^1H)$ = 6.5 Hz; **$SiMe_4$**), 7.6 (dec, $^2J(^{29}Si\text{-}^1H)$ = 5.7 Hz; **$(Me_3Si)_2O$**).

Figure S11: *NMR-spectra of experiment 9.*

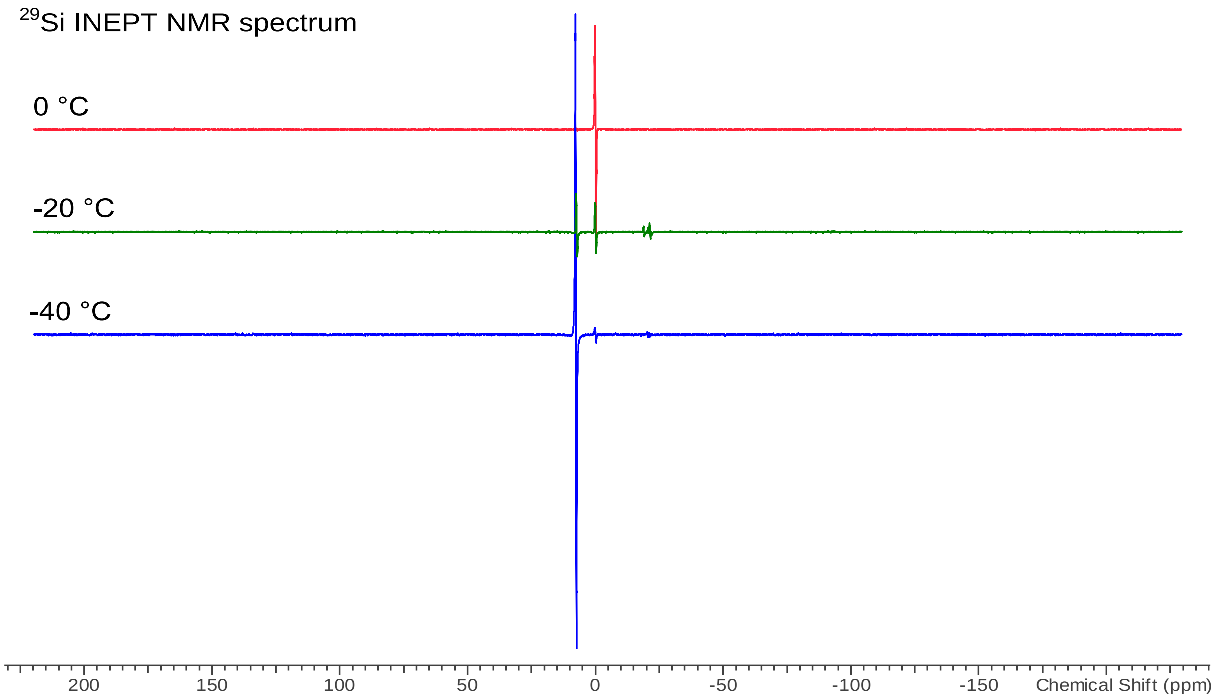

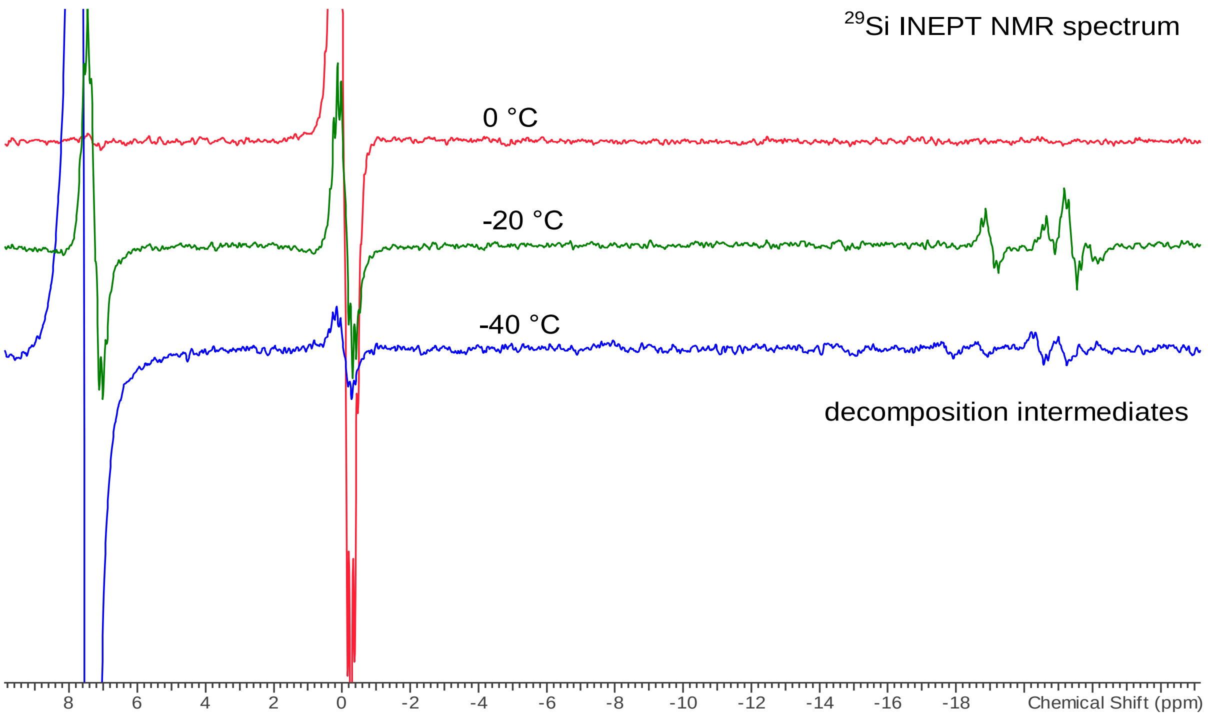

29Si INEPT NMR spectrum
0 °C
-20 °C
-40 °C
decomposition intermediates
8
6
4
2
0
-2
-4
-6
-8
-10
-12
-14
-16
-18
Chemical Shift (ppm)

4.7 Synthesis and characterization of bis-pentamethyldisilyloxonium undecachloroundecaborate – $[Me_3Si\text{-}\mu\text{-}OSiMe_2]_2[CHB_{11}Cl_{11}]_2$

$[Me_3Si][CHB_{11}Cl_{11}]$ (250 mg, 0.42 mmol) was suspended in 5 mL toluene. After the suspension has been stirred for 5 minutes and treated with an ultrasonic bath, hexamethyldisiloxane $(Me_3Si)_2O$ (70 mg, 0.43 mmol) is added via syringe. A two phase system is formed with a colourless toluene phase and an orange product phase. Warming to 70 °C and slow cooling to room temperature leads to crystals suitable for X-ray crystallographic analysis, yielding in 196 mg (0.23 mmol, 52%) pentamethyldisilyloxonium *closo*- 2, 3, 4, 5, 6, 7, 8, 9, 10, 11, 12- undecachloroundecaborate $[Me_3SiOSiMe_2][CHB_{11}Cl_{11}]$ as toluene solvate, it crystallized as a dimer.

$C_6H_{16}B_{11}Cl_{11}OSi_2$ (669.27 g/mol): **mp.** 92 °C (dec.). **EA** $[Me_3SiOSiMe_2][CHB_{11}Cl_{11}]$ · 0.75 toluene calc. (found), %: C, 18.30 (18.32); H, 3.00 (2.67); N, 0.00 (0.00). **^{1}H NMR** (25 °C, DMSO-[D_6], 300.13 MHz): δ = 0.11 (s, 9H, Si(C*H*$_3$)$_3$, $^3J(^1H\text{-}^{29}Si)$ = 7.4 Hz), 0.13 (s, 6H, Si(C*H*$_3$)$_2$, $^3J(^1H\text{-}^{29}Si)$ = 6.6 Hz), 2.30 (s, 3H, $C_6H_5CH_3$), 7.10 - 7.29 (m, 5H, $C_6H_5CH_3$). **^{11}B NMR** (25 °C, DMSO-[D_6], 96.29 MHz): δ = –13.0 (s, $B_{2\text{-}6}$Cl), –9.9 (s, $B_{7\text{-}11}$Cl), –2.8 (s, B_{12}Cl). **^{13}C{^{1}H} NMR** (25 °C, DMSO-[D_6], 75.47 MHz): δ = 0.0 (s, Si*C*H$_3$), 1.8 (s, Si*C*H$_3$), 21.0 (s, $C_6H_5CH_3$), 125.3 (s, *p*-C_6H_5), 128.2 (s, *m*-C_6H_5), 128.9 (s, *o*-C_6H_5), 137.3 (s, *ipso*-C_6H_5). **^{17}O NMR** (25 °C, DMSO-[D_6], 33.90 MHz): δ = not observed. **^{29}Si INEPT NMR** (25 °C, DMSO-[D_6], 59.62 MHz): δ = −17.4 (sep, *Si*(CH$_3$)$_2$, $^3J(^{29}Si\text{-}^1H)$ = 7.4 Hz), 42.6 (dec, *Si*(CH$_3$)$_3$, $^3J(^{29}Si\text{-}^1H)$ = 7.4 Hz). **IR** (ATR, 8 scans, 25 °C, cm^{-1})*: 3499 (w), 3349 (w), 3340 (w), 3023 (w), 3002 (w), 2965 (w), 2910 (w), 2075 (w), 2021 (w), 1663 (w), 1603 (w), 1494 (w), 1416 (w), 1265 (m), 1203 (w), 1117 (s), 1030 (s), 1008 (s), 954 (m), 898 (m), 859 (m), 845 (m), 814 (s), 734 (m), 713 (m), 696 (m), 670 (m), 643 (m), 626 (m), 581 (m), 530 (s), 488 (s), 466 (m). **MS** (CI+, m/z (%)): 563 $[(Me_3SiOSiMe_2)(CHB_{11}Cl_8)]^+$, 579 $[(Me_3SiOSiMe_3)(CHB_{11}Cl_8H)]^+$ /$[CHB_{11}Cl_{11}\cdot C_4H_9]^+$, 598 $[(Me_3SiOSiMe_2)(CHB_{11}Cl_9H)]^+$, 669 $[Me_3SiOSiMe_2][CHB_{11}Cl_{11}]^+$, 891 $[[Me_3SiOSiMe_2][CHB_{11}Cl_{11}]\cdot C_7H_8]^+$, 973 $[(CHB_{11}Cl_{10})_2]^+$.

In solution, we observed also the formation of the monomeric species: **^{1}H NMR** (25 °C, DMSO-[D_6]DMSO-[D_6], 300.13 MHz): δ = 0.17 (s, 6H, Si(C*H*$_3$)$_2$, $^3J(^1H\text{-}^{29}Si)$ = 6.2 Hz), 0.40 (s, 9H, Si(C*H*$_3$)$_3$, $^3J(^1H\text{-}^{29}Si)$ = 7.4 Hz). **^{13}C{^{1}H} NMR** (25 °C, DMSO-[D_6], 75.47 MHz): δ = –0.5 (s, Si*C*H$_3$), 0.9 (s, Si*C*H$_3$). **^{29}Si INEPT NMR** (25 °C, DMSO-[D_6], 59.62 MHz): δ = 1.5 (sep, *Si*(CH$_3$)$_2$, $^3J(^{29}Si\text{-}^1H)$ = 8.2 Hz), 9.0 (dec, *Si*(CH$_3$)$_3$, $^3J(^{29}Si\text{-}^1H)$ = 6.5 Hz).

* Hydrolysis during sample preparation.

5 NMR, IR and Raman spectra

Figure S12: ^{1}H NMR spectra (300.13 MHz) of **[K[18]crown-6][SSiMe$_3$]** in THF-[D$_8$].

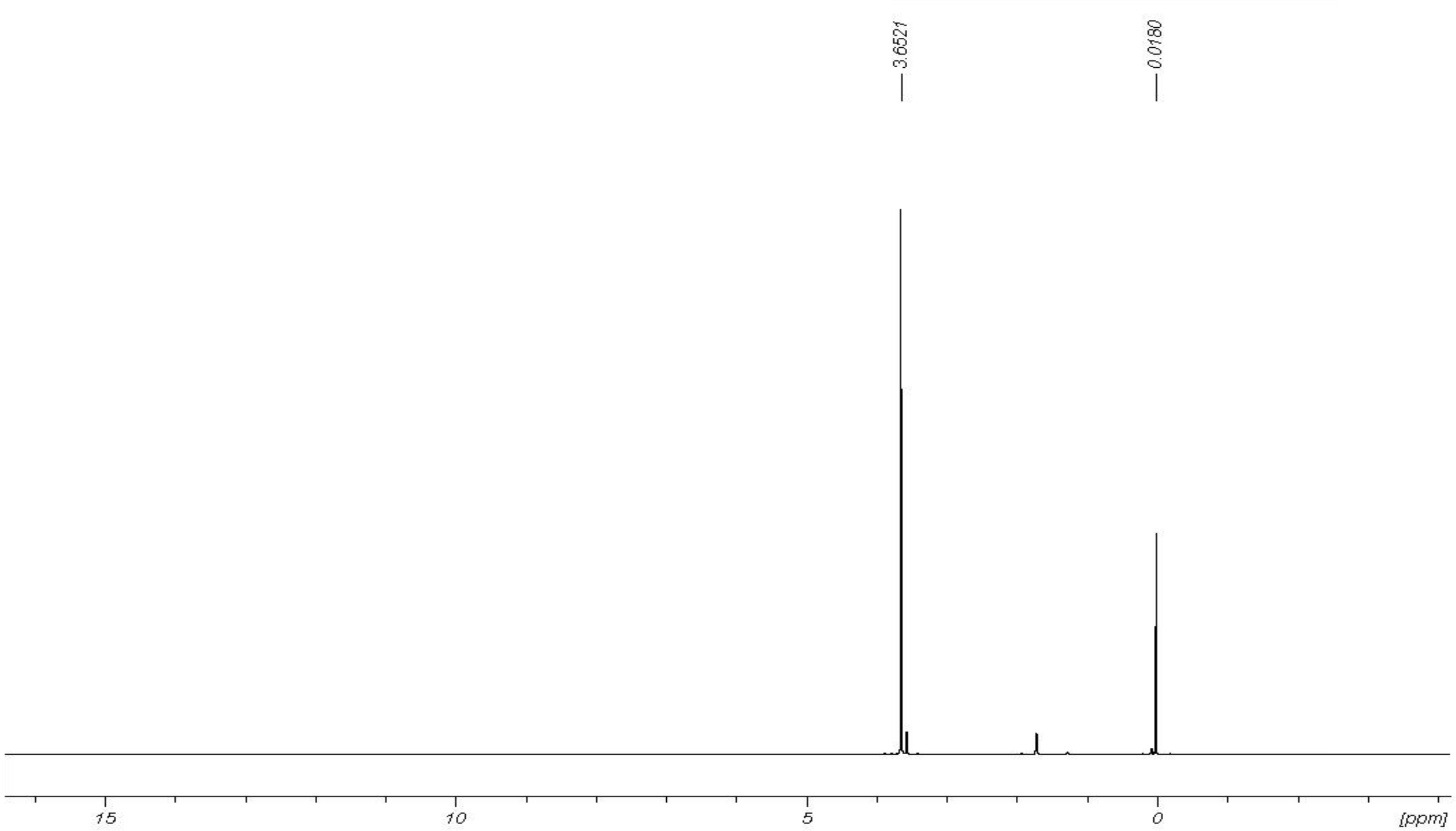

Figure S13: $^{13}C\{^{1}H\}$ NMR spectra (75.47 MHz) of **[K·[18]crown-6][SSiMe$_3$]** in THF-[D_8].

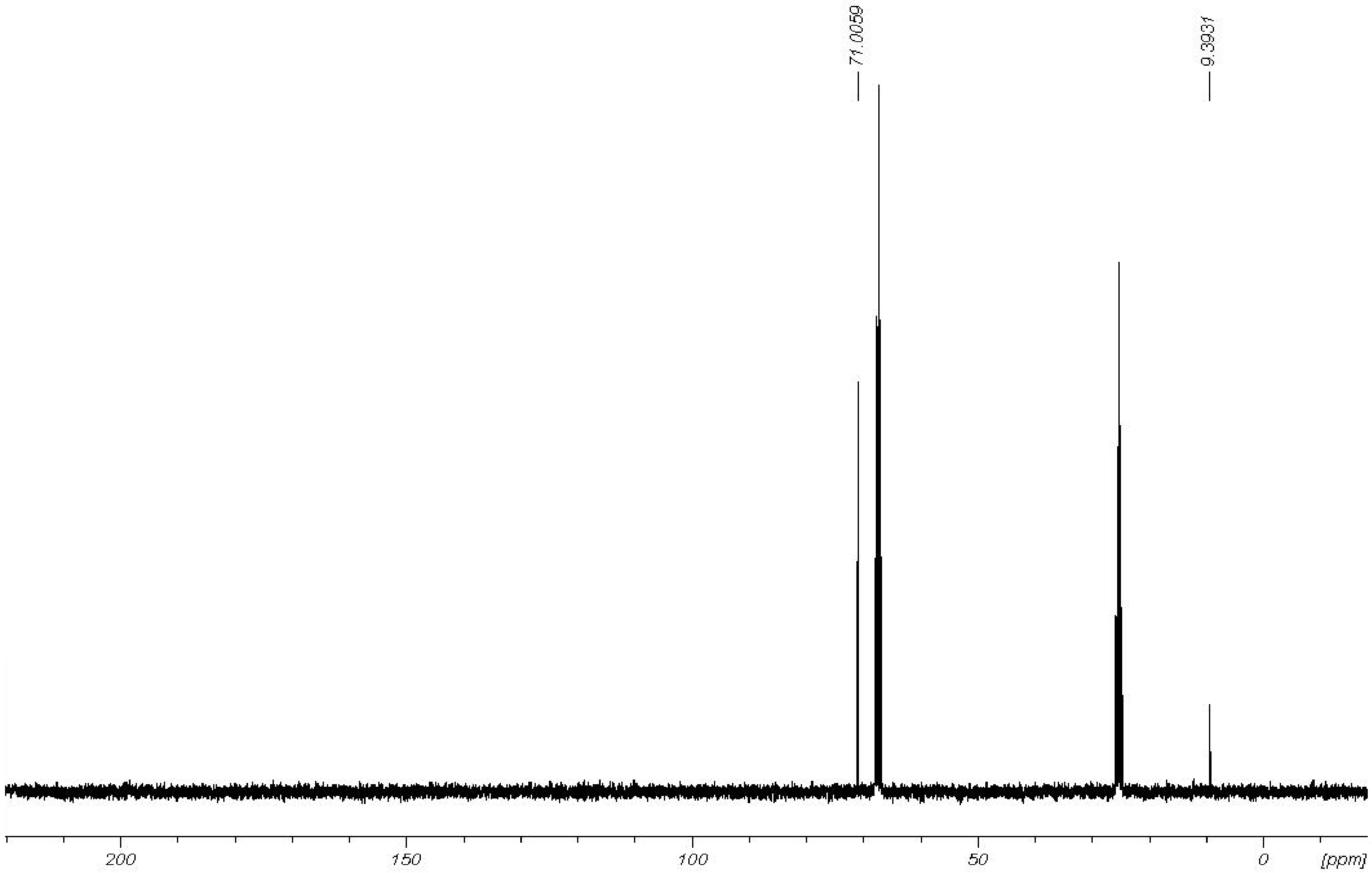

Figure S14: ^{29}Si INEPT NMR spectra (59.63 MHz) of **[K·[18]crown-6][SSiMe$_3$]** in THF-[D$_8$].

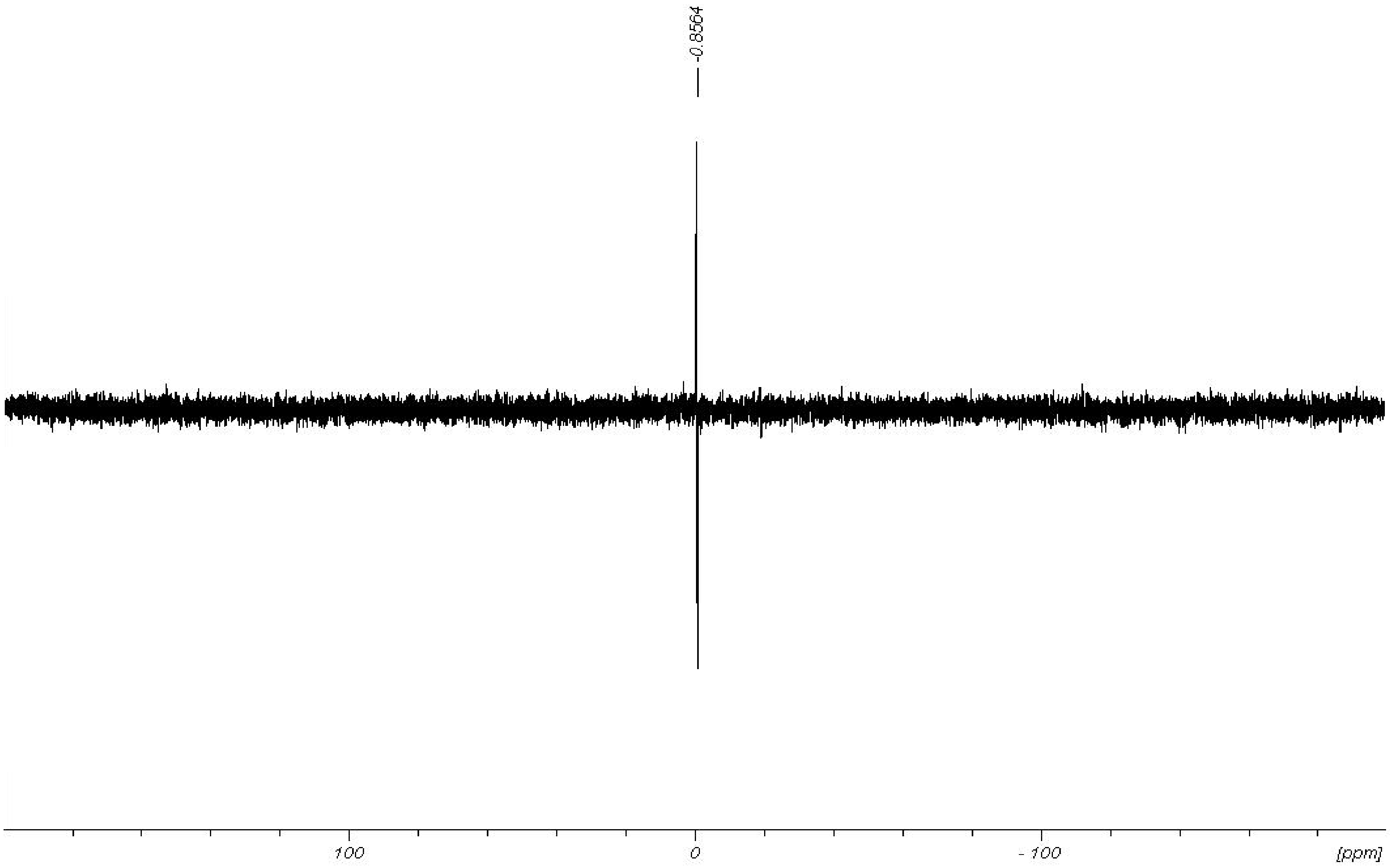

Figure S15: ^{1}H NMR spectra (300.13 MHz) of **$S(SiMe_3)_2$** in THF-[D_8].

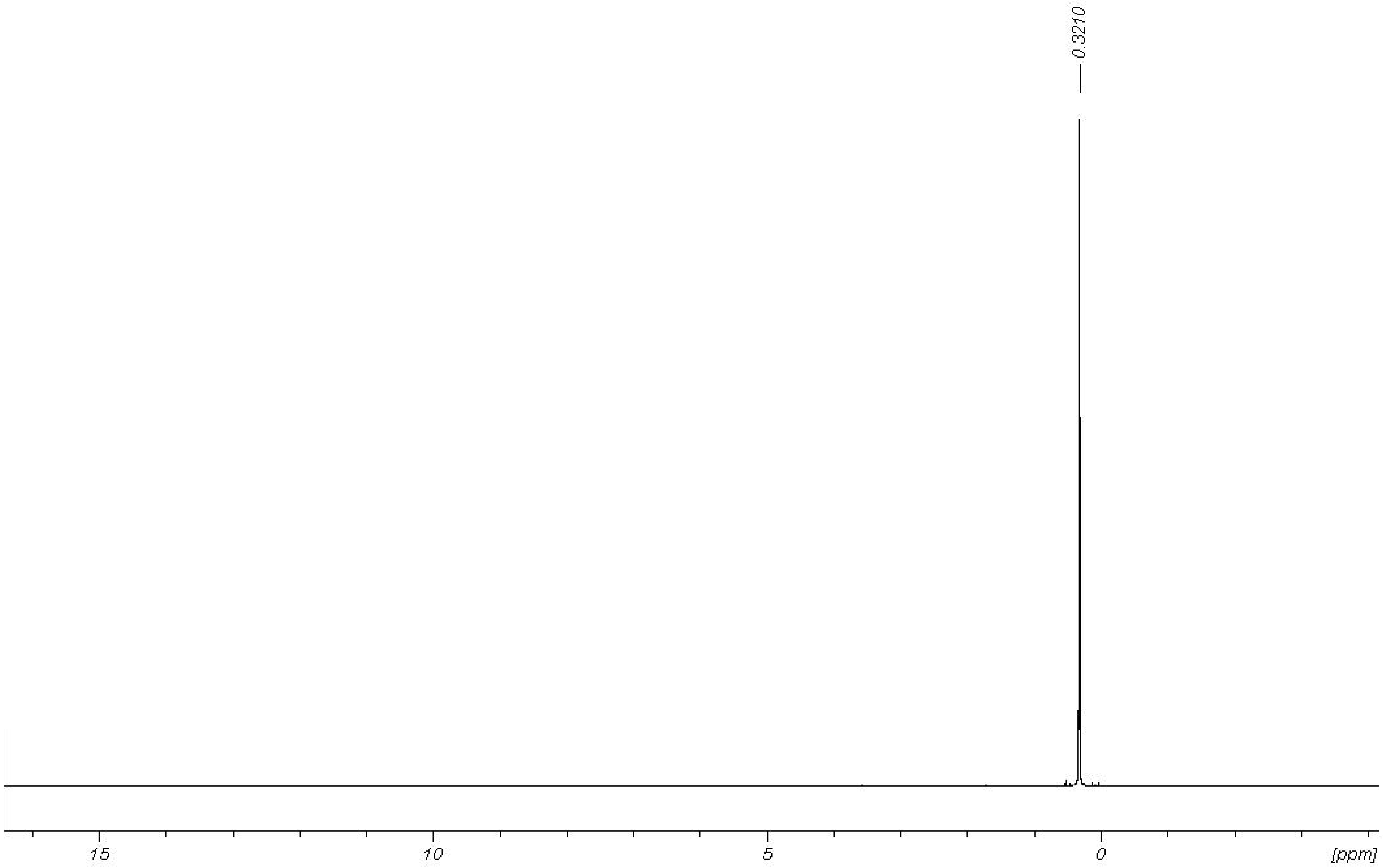

Figure S16: $^{13}C\{^{1}H\}$ NMR spectra (75.47 MHz) of **$S(SiMe_3)_2$** in THF-[D_8].

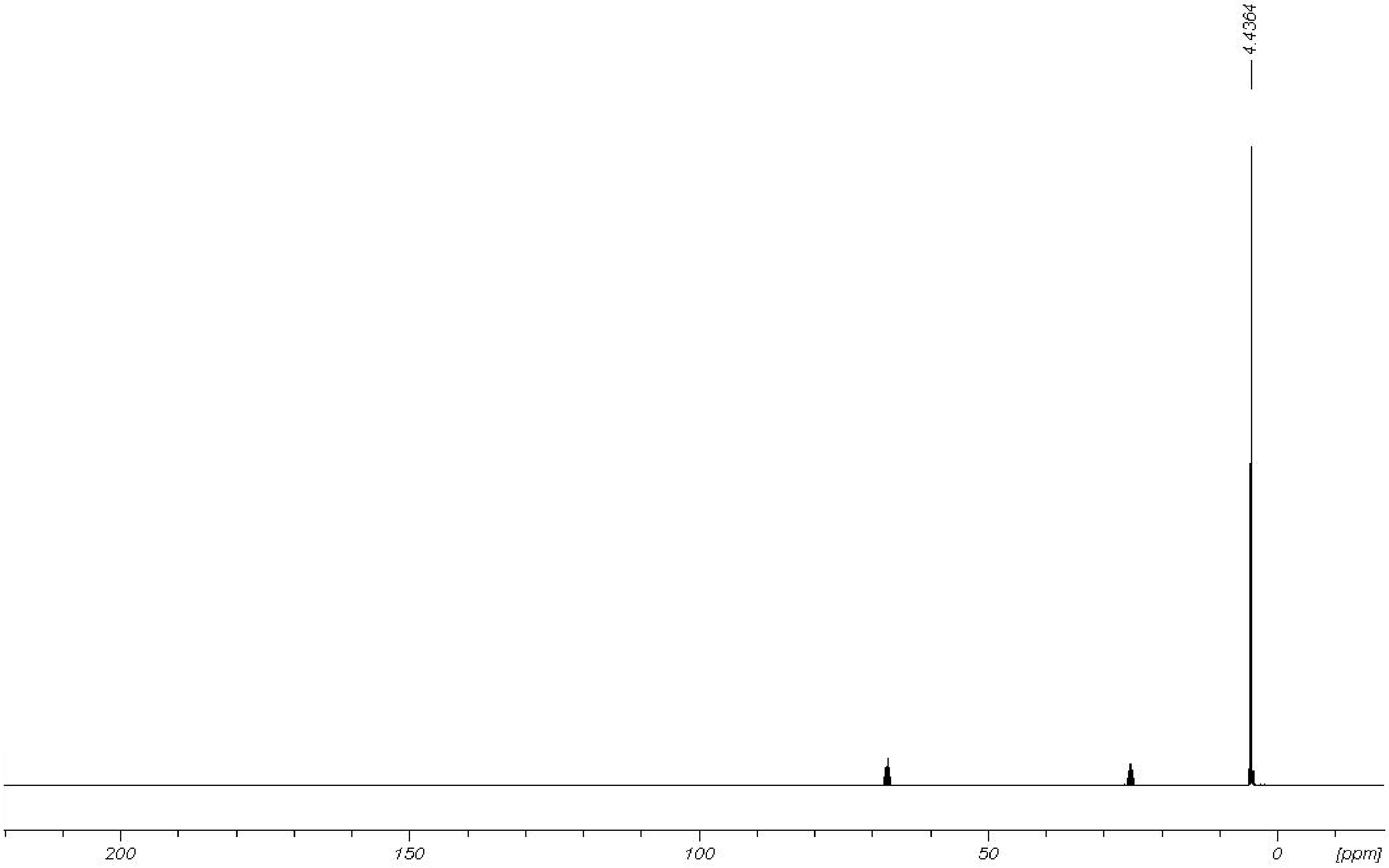

Figure S17: ^{29}Si INEPT NMR spectra (59.63 MHz) of **$S(SiMe_3)_2$** in THF-[D_8].

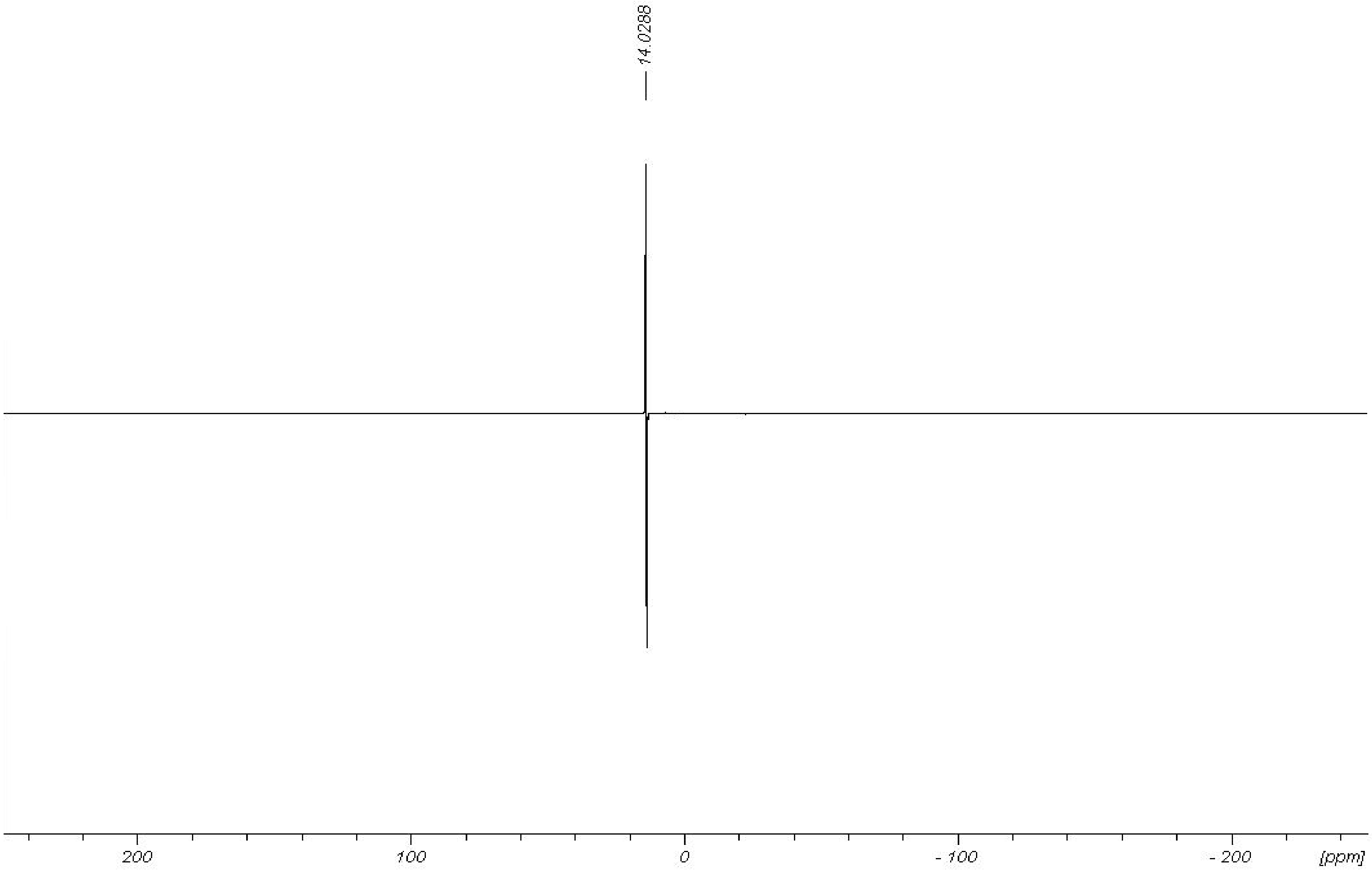

Figure S18: *NMR-spectra of* ***[K•[18]crown-6]O-SiMe₃*** *in THF-[D₈] (solvent signals indicated by asterisk, unknown impurities indicated plus).*

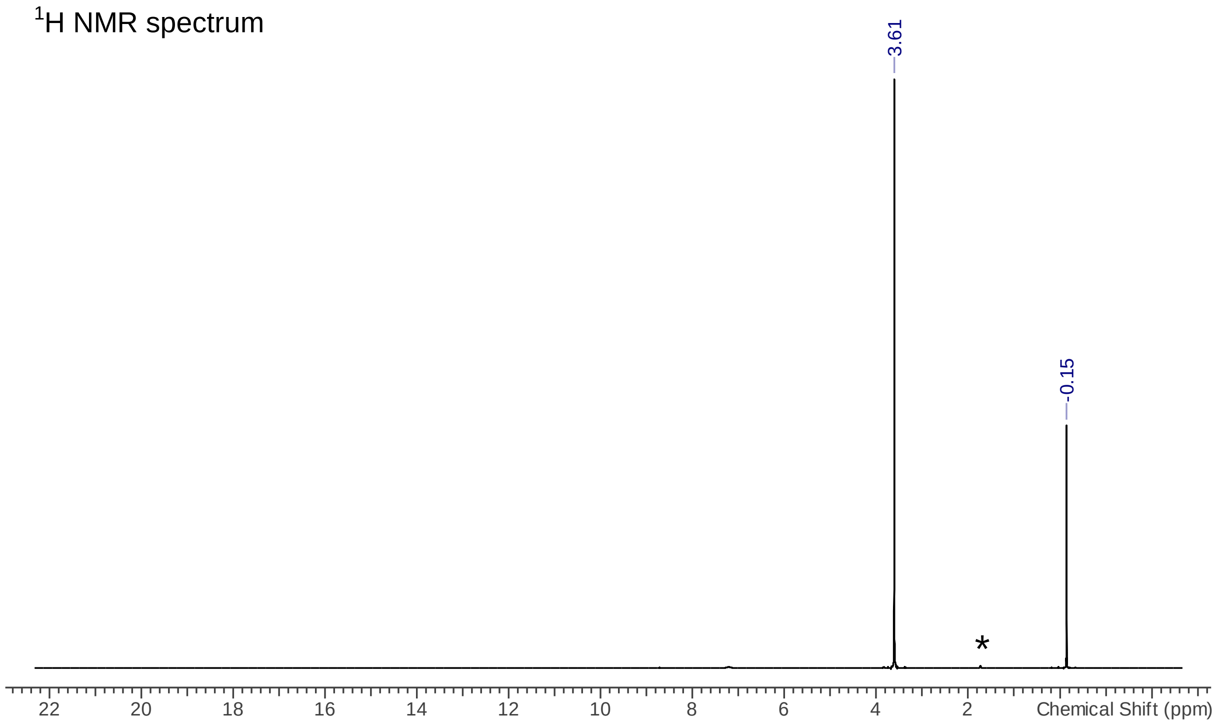

^{29}Si INEPT NMR spectrum

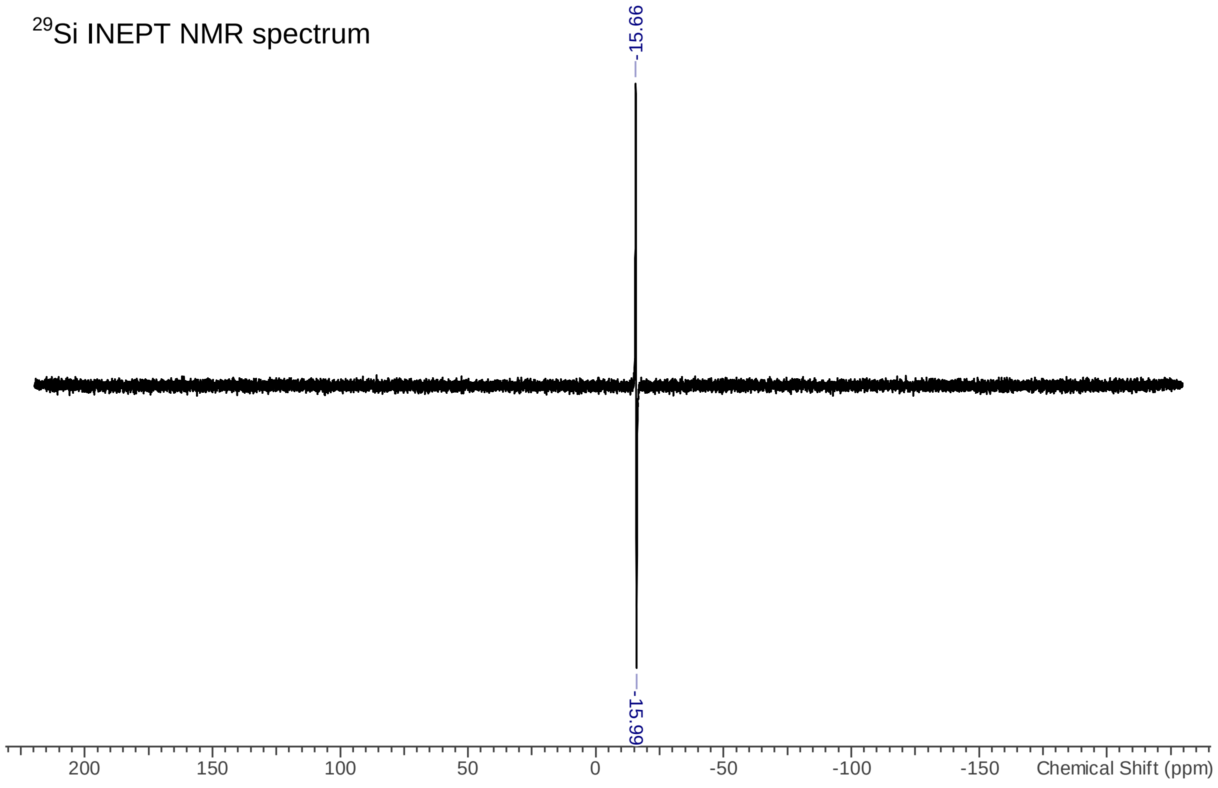

^{13}C {^{1}H} NMR spectrum

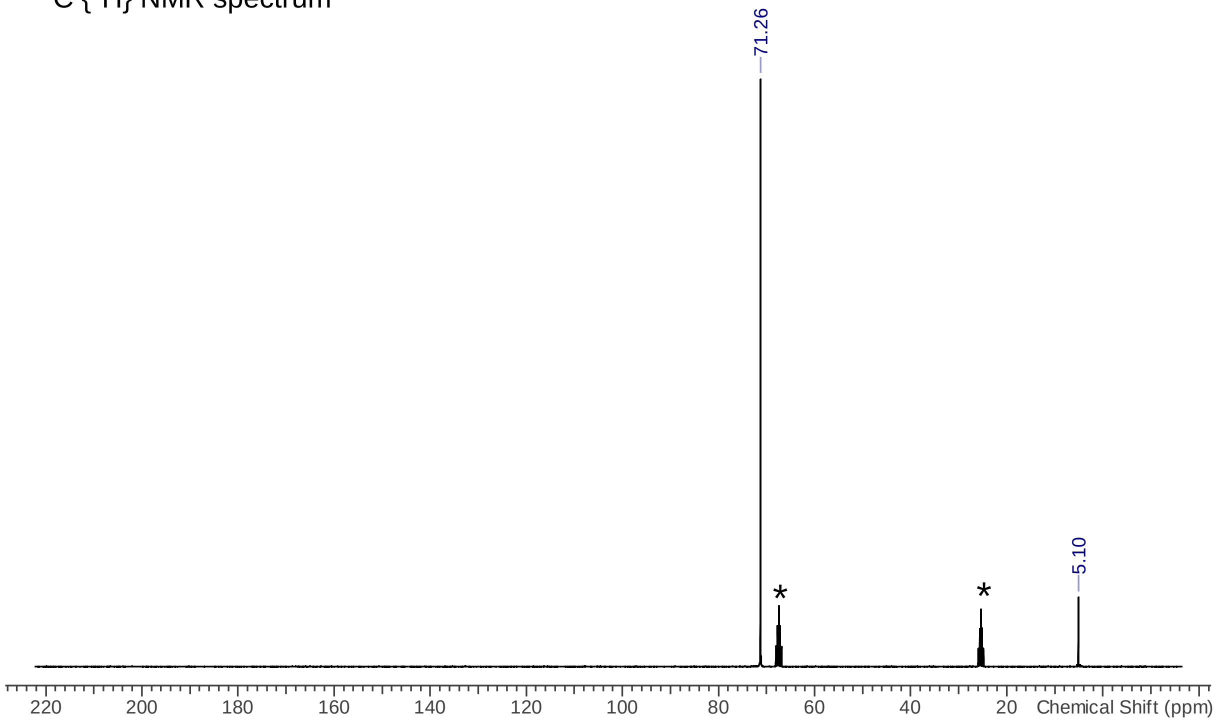

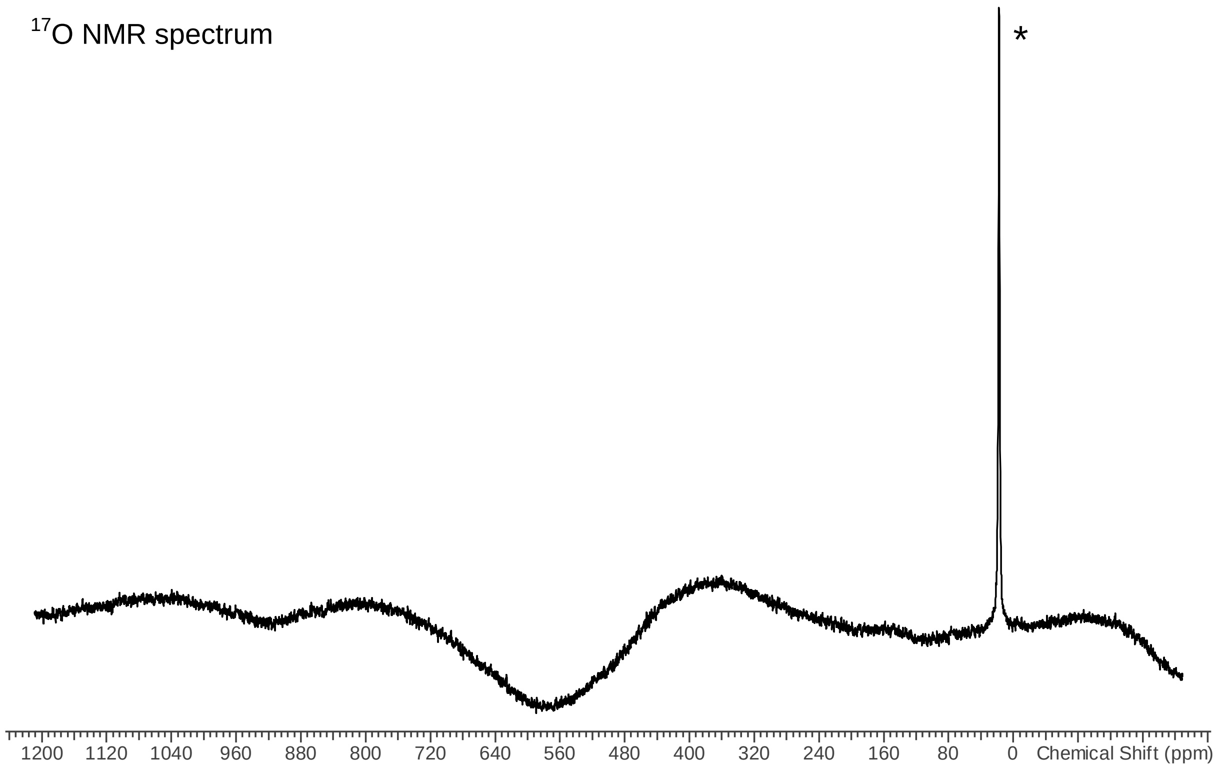

17O NMR spectrum
*
1200
1120
1040
960
880
800
720
640
560
480
400
320
240
160
80
0
Chemical Shift (ppm)

Figure S19: ^{1}H NMR spectra (300.13 MHz) of **$[(Me_3Si)_3S][CHB_{11}H_5Cl_6]$** in CD_2Cl_2.

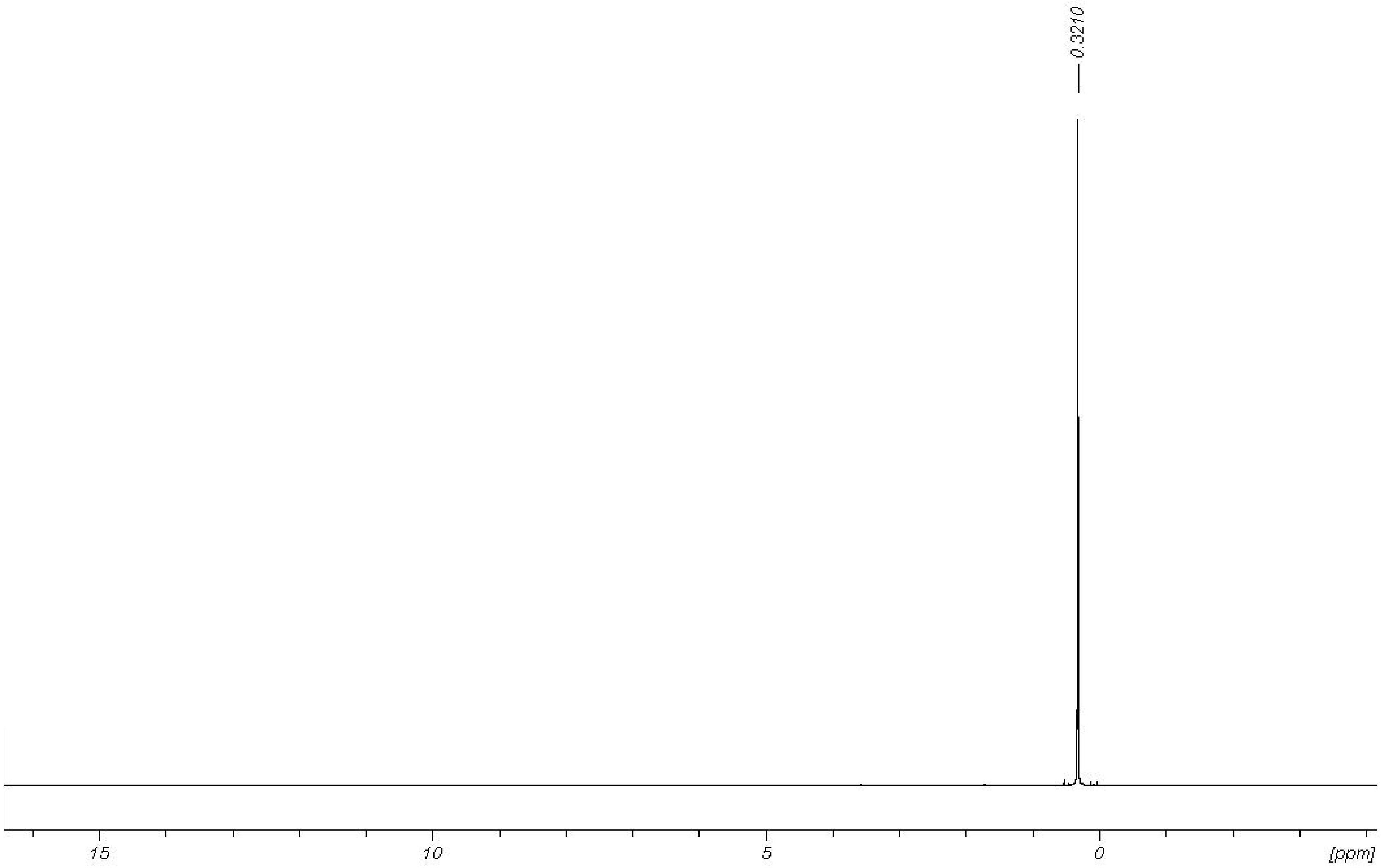

Figure S20: ^{1}H NMR spectra (300.13 MHz) of **[(Me$_3$Si)$_3$S][CHB$_{11}$H$_5$Cl$_6$]** in CD$_2$Cl$_2$. Area of BH/CH protons.

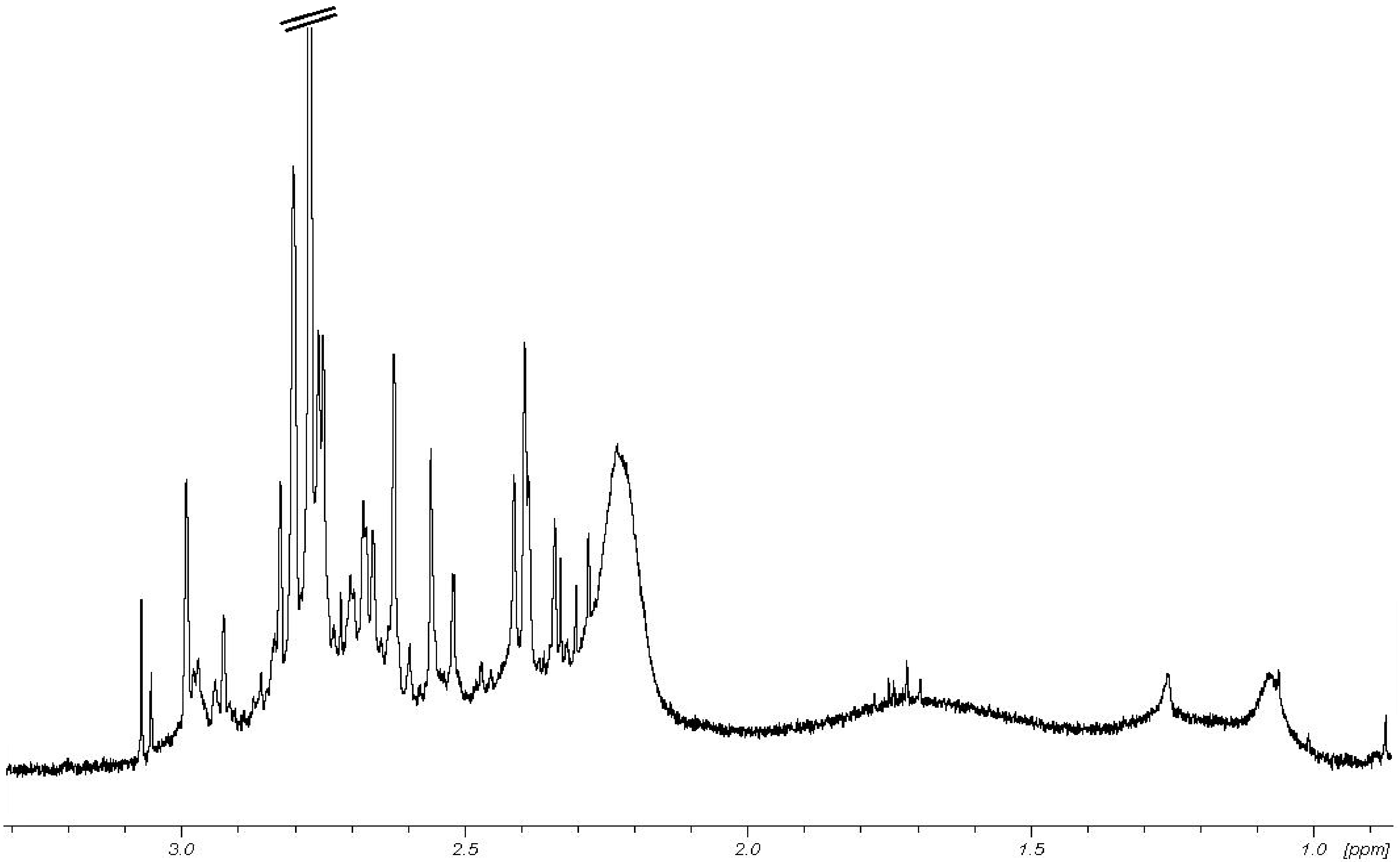

Figure S21: ^{11}B NMR spectra (96.29 MHz) of **[(Me$_3$Si)$_3$S][CHB$_{11}$H$_5$Cl$_6$]** in CD_2Cl_2. The asterisk marks a signal which arises from another species the partly over chlorinated [CHB$_{11}$H$_4$Cl$_7$]$^-$ anion (*vide infra* X-ray data *Table S1*).

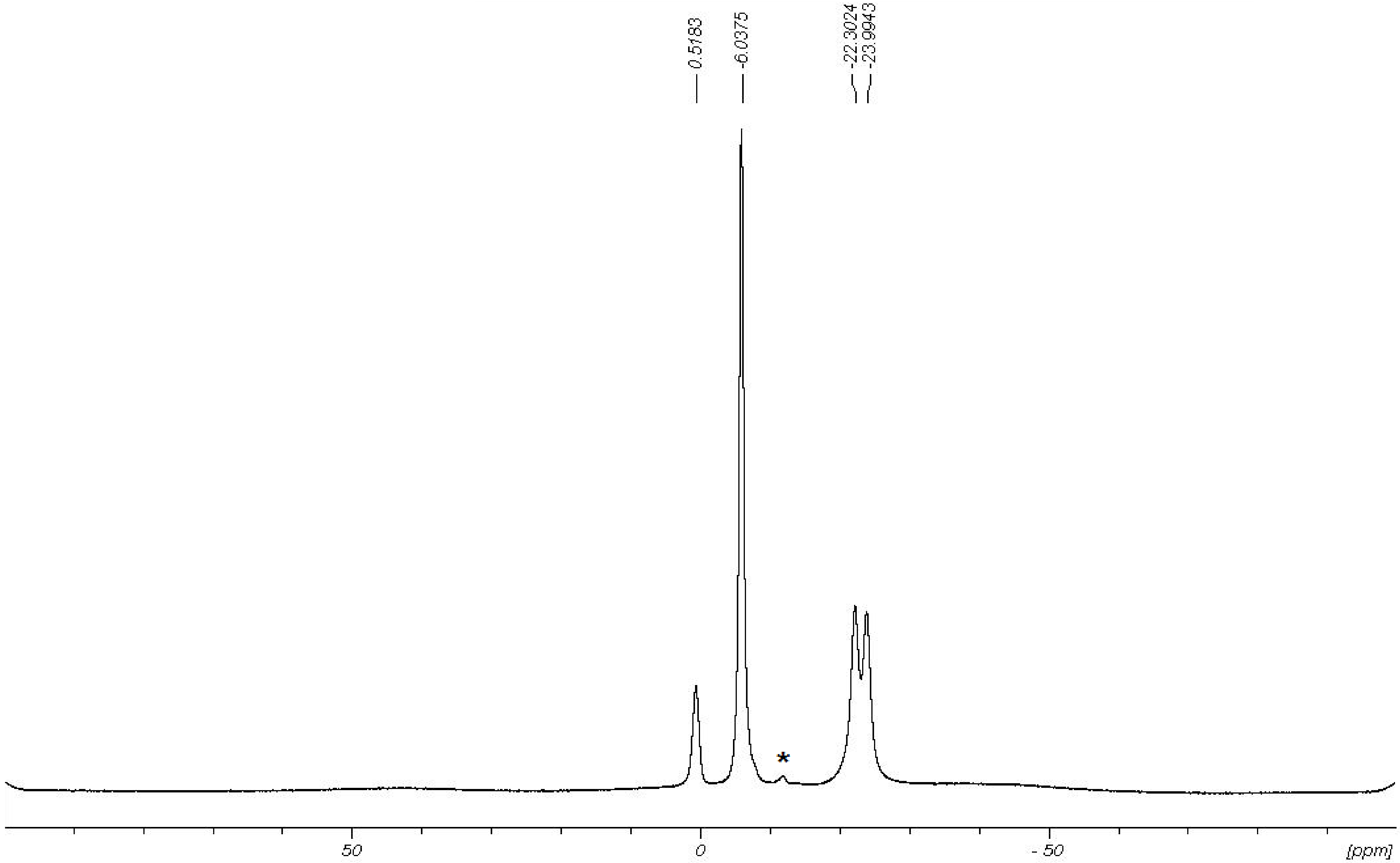

Figure S22: $^{13}C\{^1H\}$ NMR spectra (75.47 MHz) of **$[(Me_3Si)_3S][CHB_{11}H_5Cl_6]$** in CD_2Cl_2. The asterisk marks unreacted $S(SiMe_3)_2$.

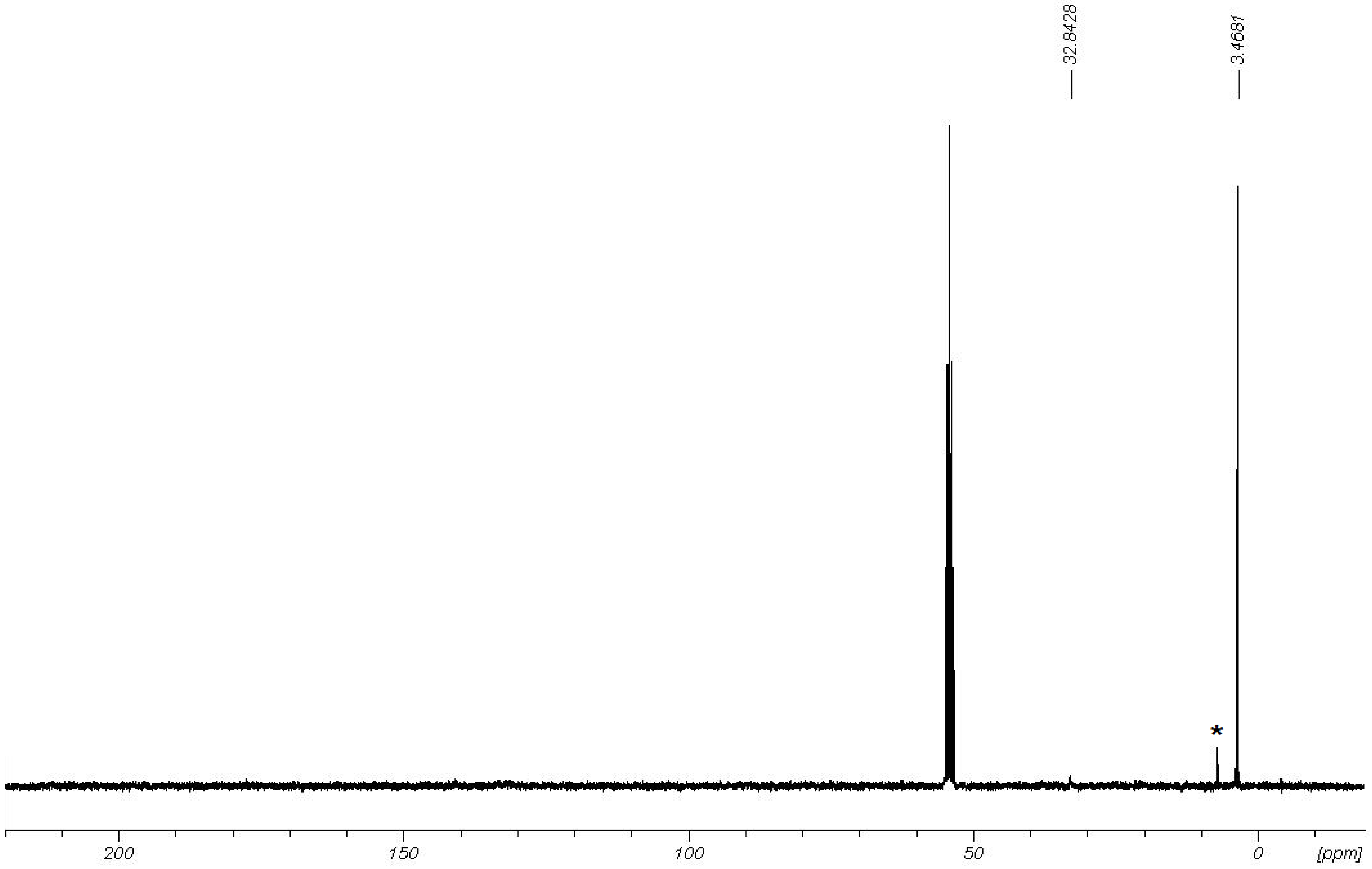

Figure S23: ^{29}Si INEPT NMR spectra (59.63 MHz) of **[(Me$_3$Si)$_3$S][CHB$_{11}$H$_5$Cl$_6$]** in CD$_2$Cl$_2$.

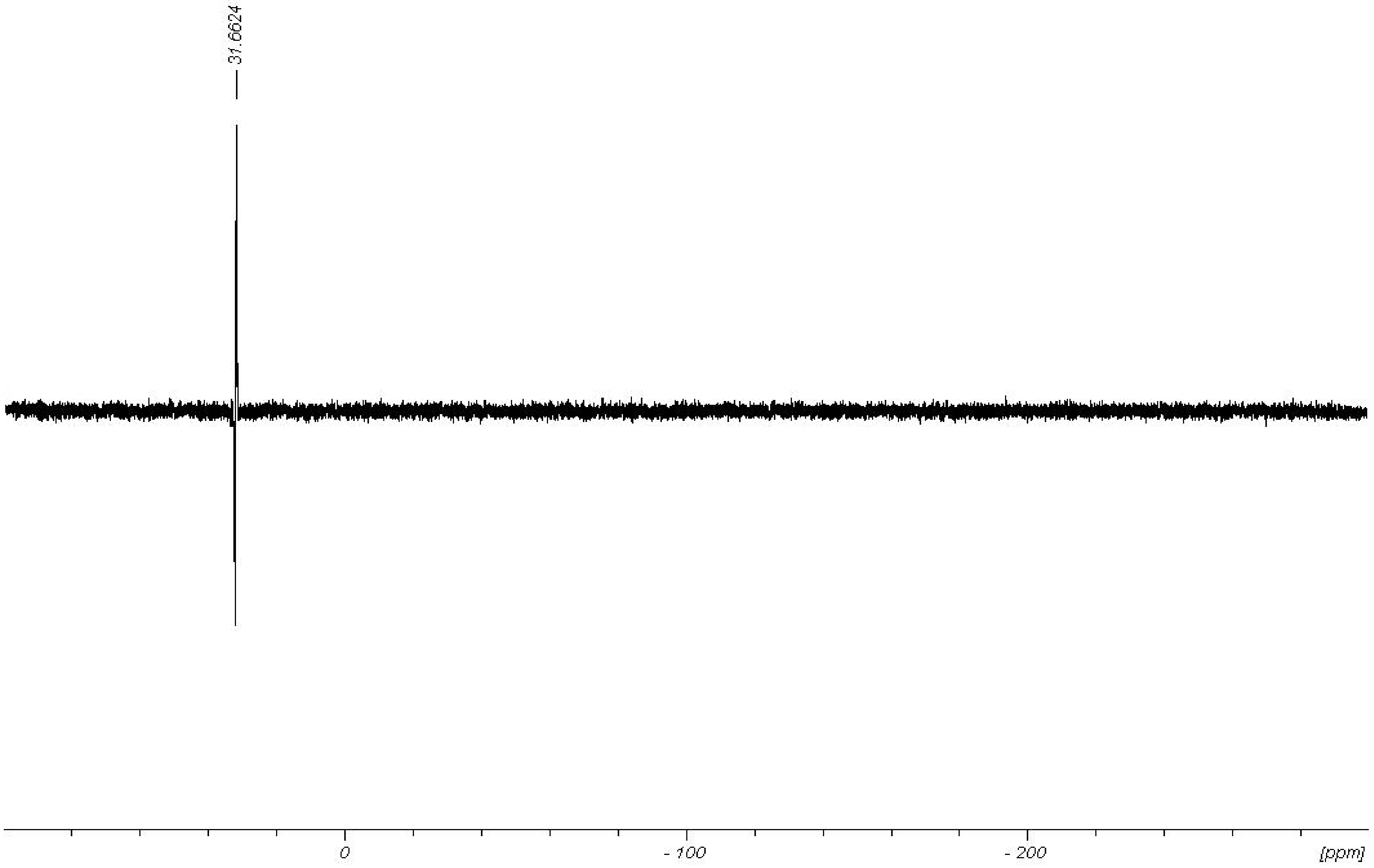

Figure S24: ^{1}H NMR spectra (300.13 MHz) of **[(Me$_3$Si)$_3$S][B(C$_6$F$_5$)$_4$]** in toluene referenced externally to CD_2Cl_2 which was added *via* a capillary.

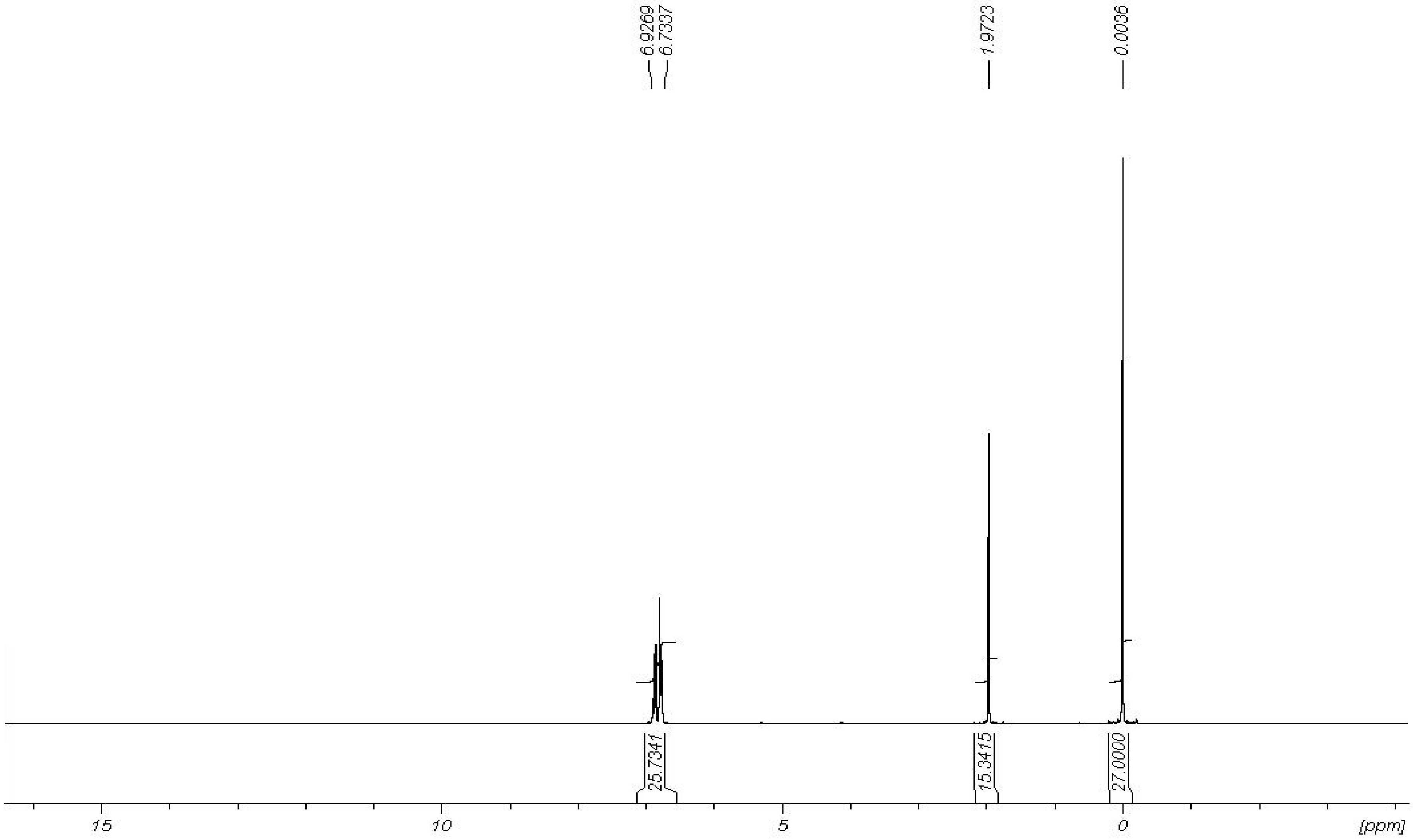

Figure S25: ^{11}B NMR spectra (96.29 MHz) of **$[(Me_3Si)_3S][B(C_6F_5)_4]$** in toluene.

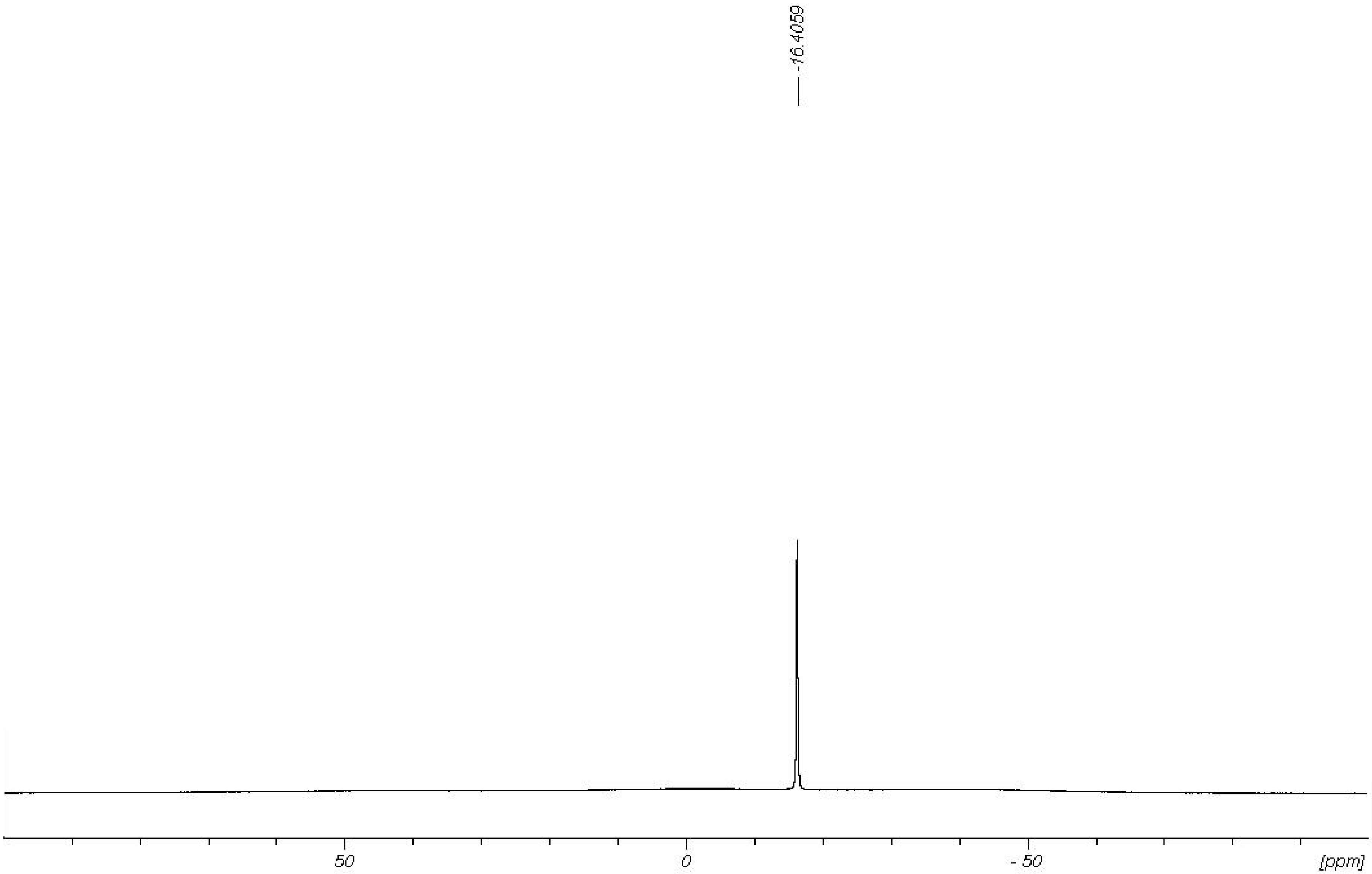

Figure S26: $^{13}C\{^{1}H\}$ NMR spectra (75.47 MHz) of **$[(Me_3Si)_3S][B(C_6F_5)_4]$** in toluene.

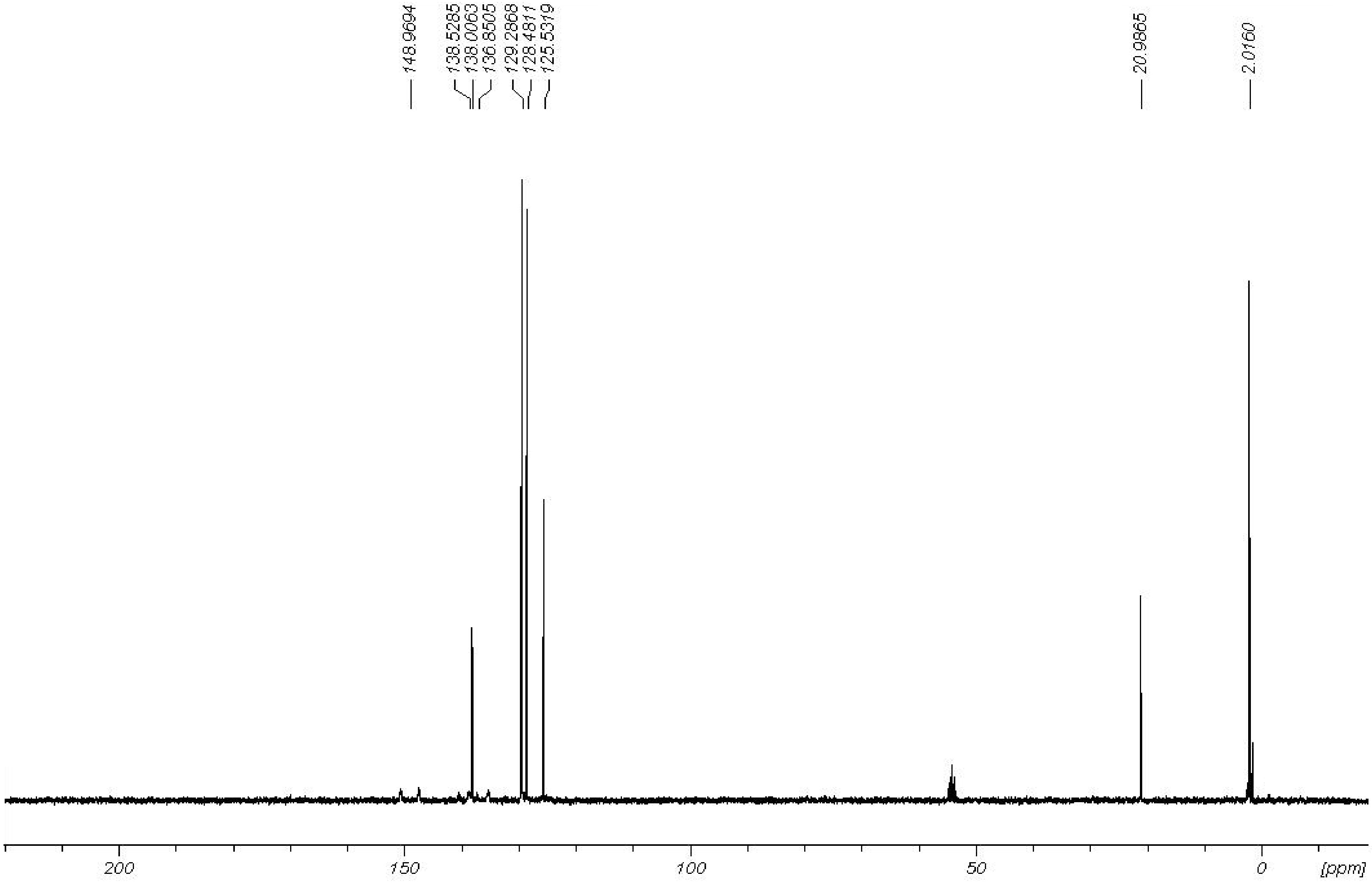

Figure S27: $^{19}F\{^1H\}$ NMR spectra (282.38 MHz) of **[(Me$_3$Si)$_3$S][B(C$_6$F$_5$)$_4$]** in toluene.

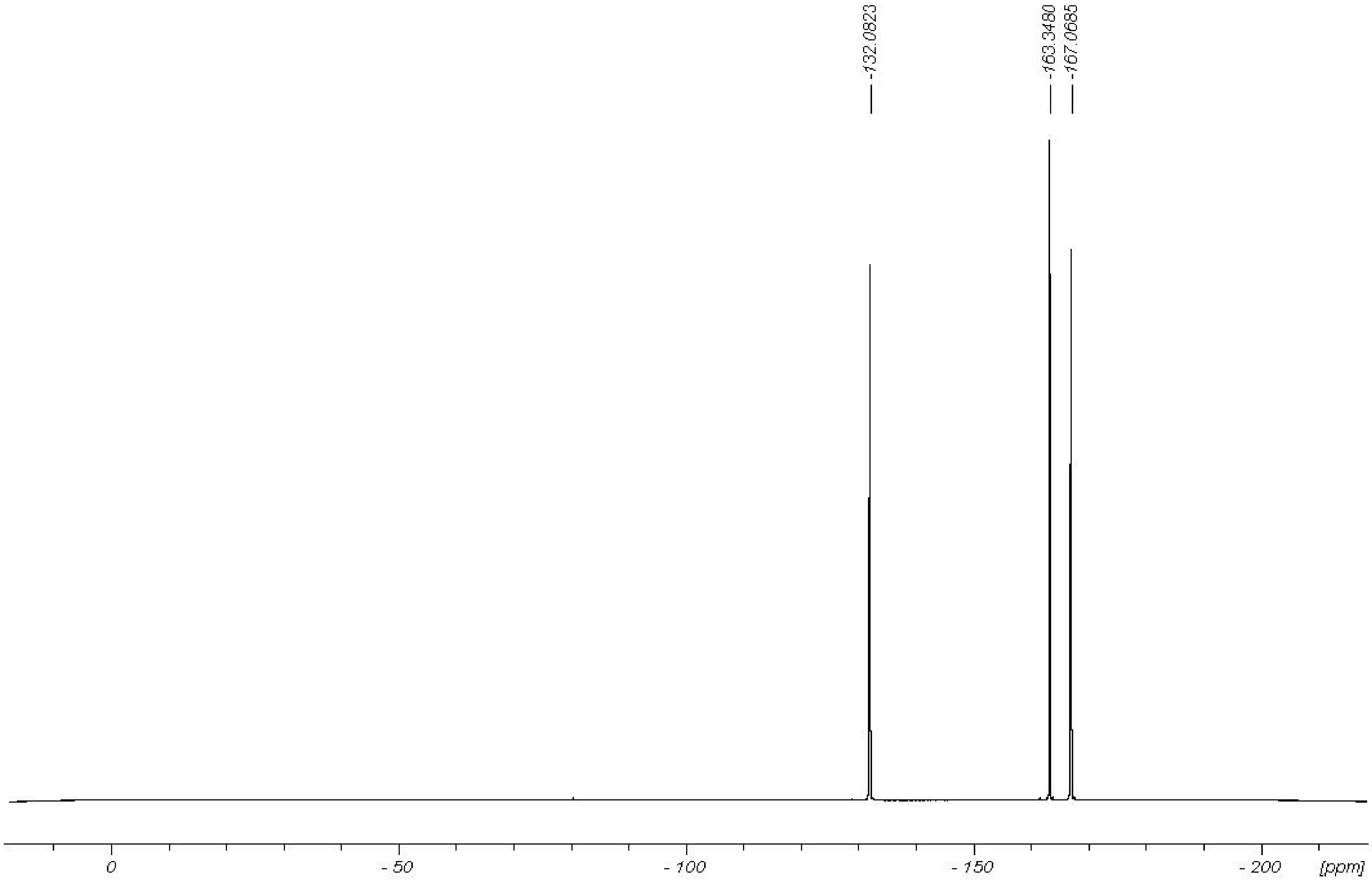

Figure S28: $^{29}Si\{^{1}H\}$ INEPT NMR spectra (59.63 MHz) of **[(Me$_3$Si)$_3$S][B(C$_6$F$_5$)$_4$]** in toluene.

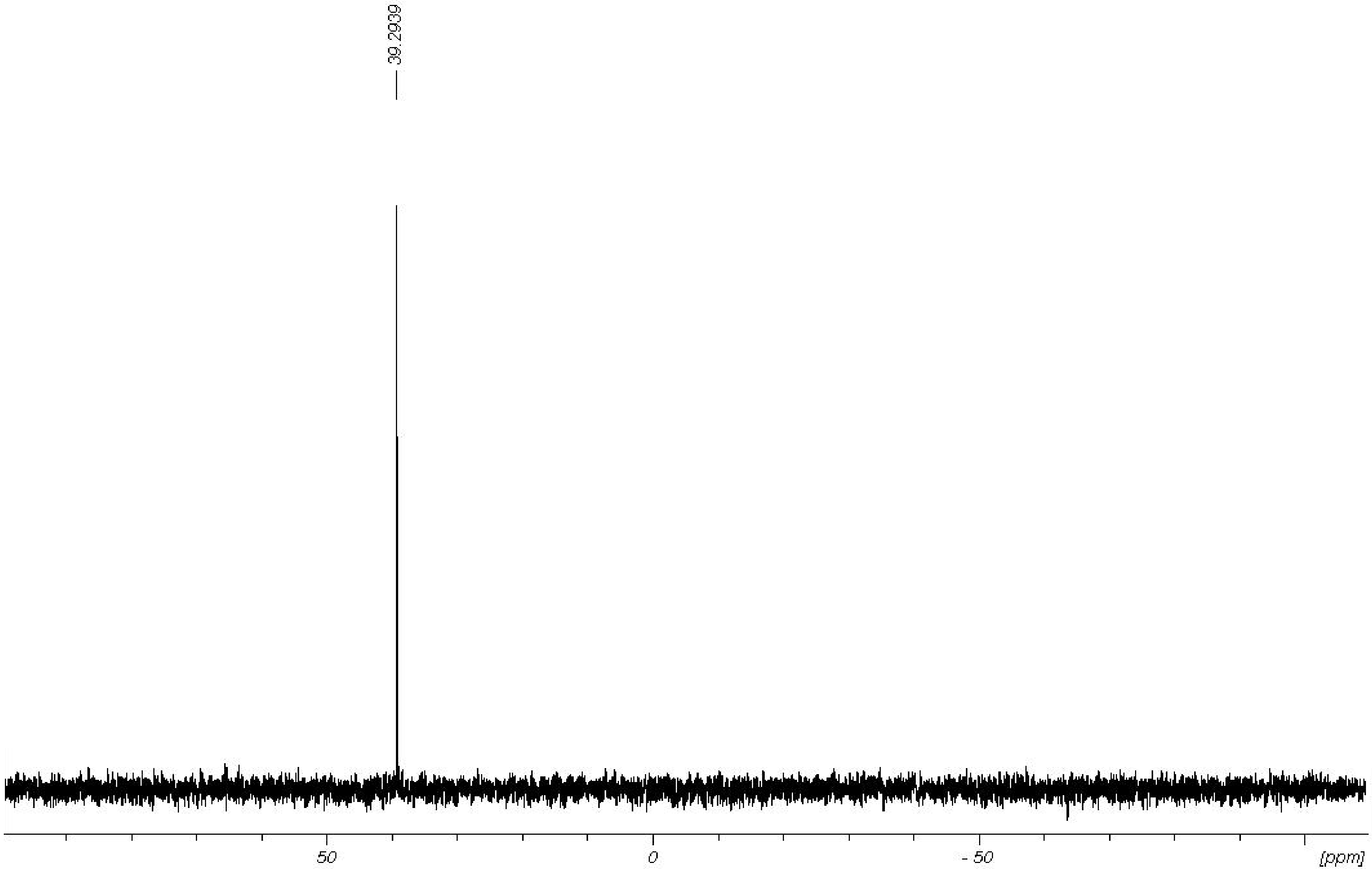

Figure S29: ^{1}H NMR spectra (300.13 MHz) of **[(Me$_3$Si)$_3$S][B(C$_6$F$_5$)$_4$]** in 1,2-DCB referenced externally to acetone-[D$_6$].

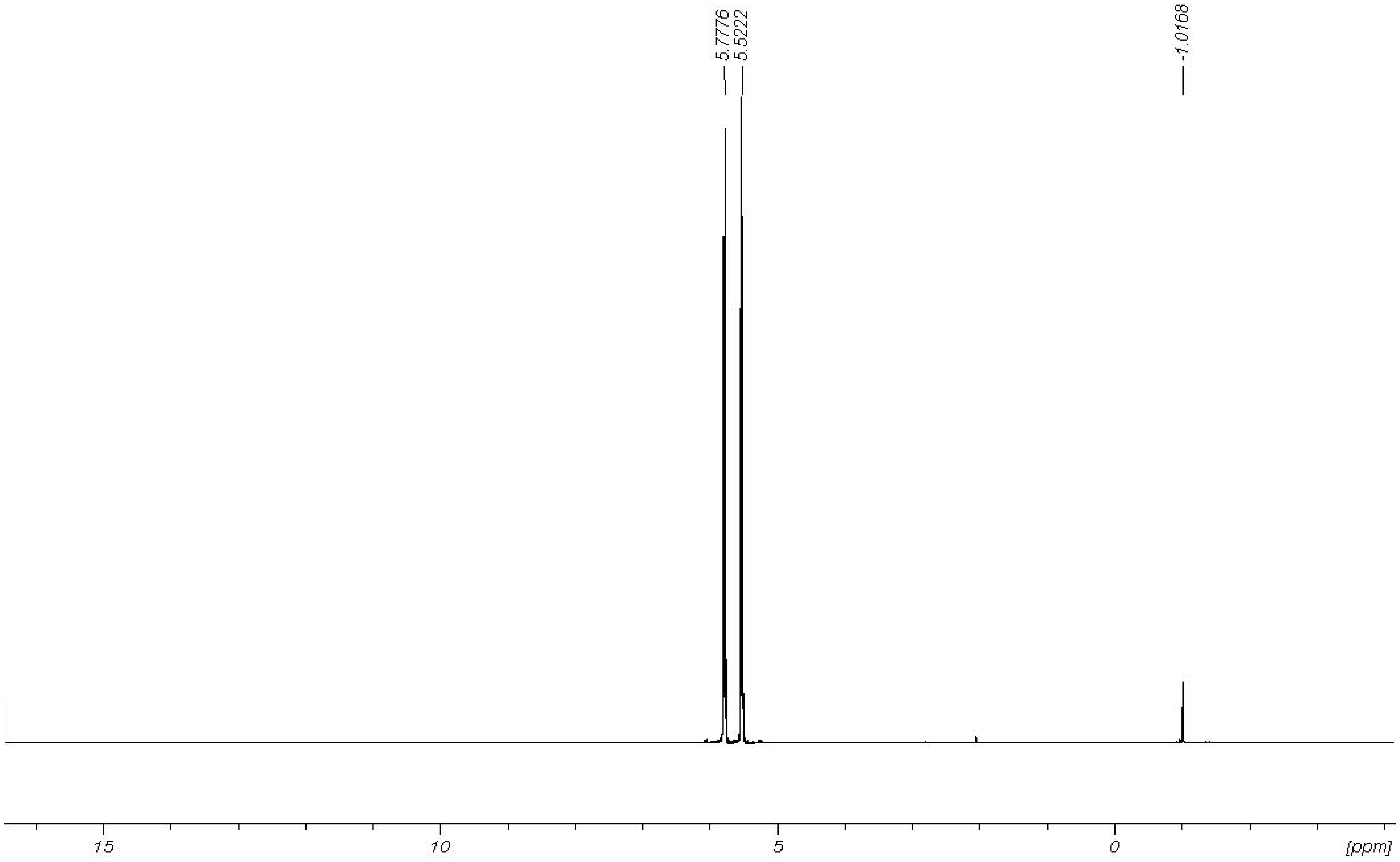

Figure S30: ^{11}B NMR spectra (96.29 MHz) of **$[(Me_3Si)_3S][B(C_6F_5)_4]$** in 1,2-DCB.

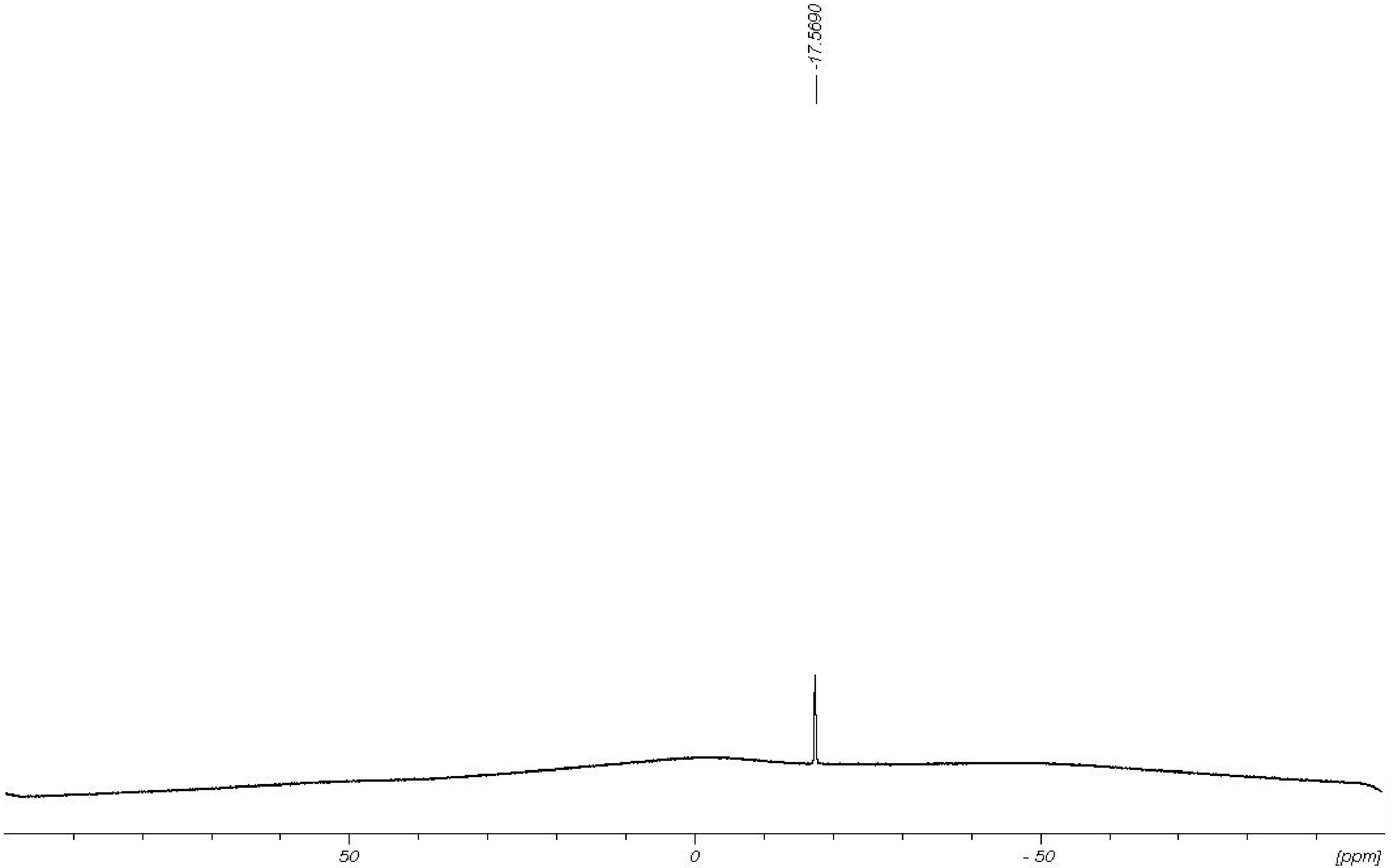

Figure S31: $^{13}C\{^{1}H\}$ NMR spectra (75.47 MHz) of **$[(Me_3Si)_3S][B(C_6F_5)_4]$** in 1,2-DCB referenced externally to acetone-$[D_6]$.

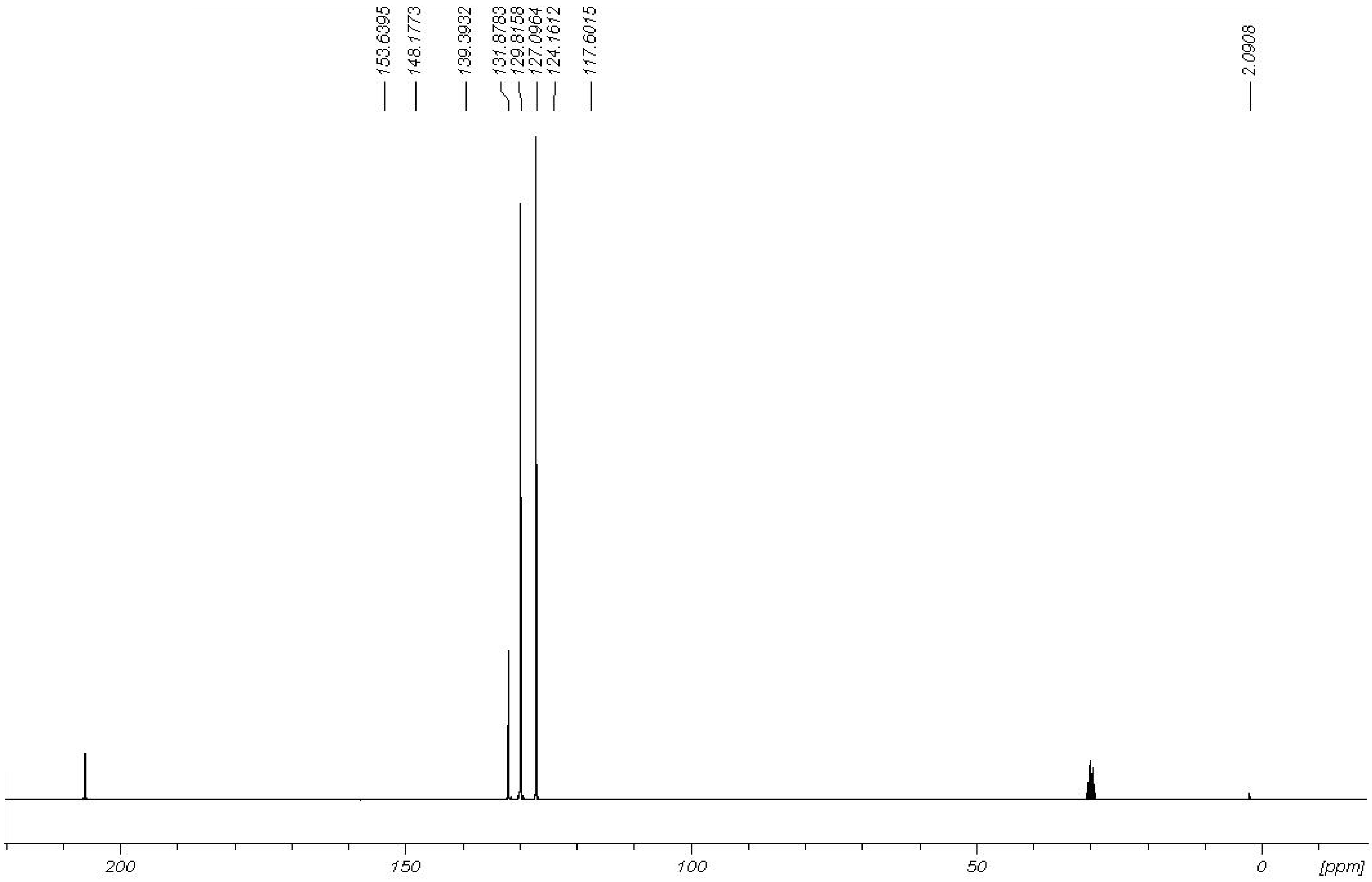

Figure S32: $^{19}F\{^{1}H\}$ NMR spectra (282.38 MHz) of **$[(Me_3Si)_3S][B(C_6F_5)_4]$** in 1,2-DCB.

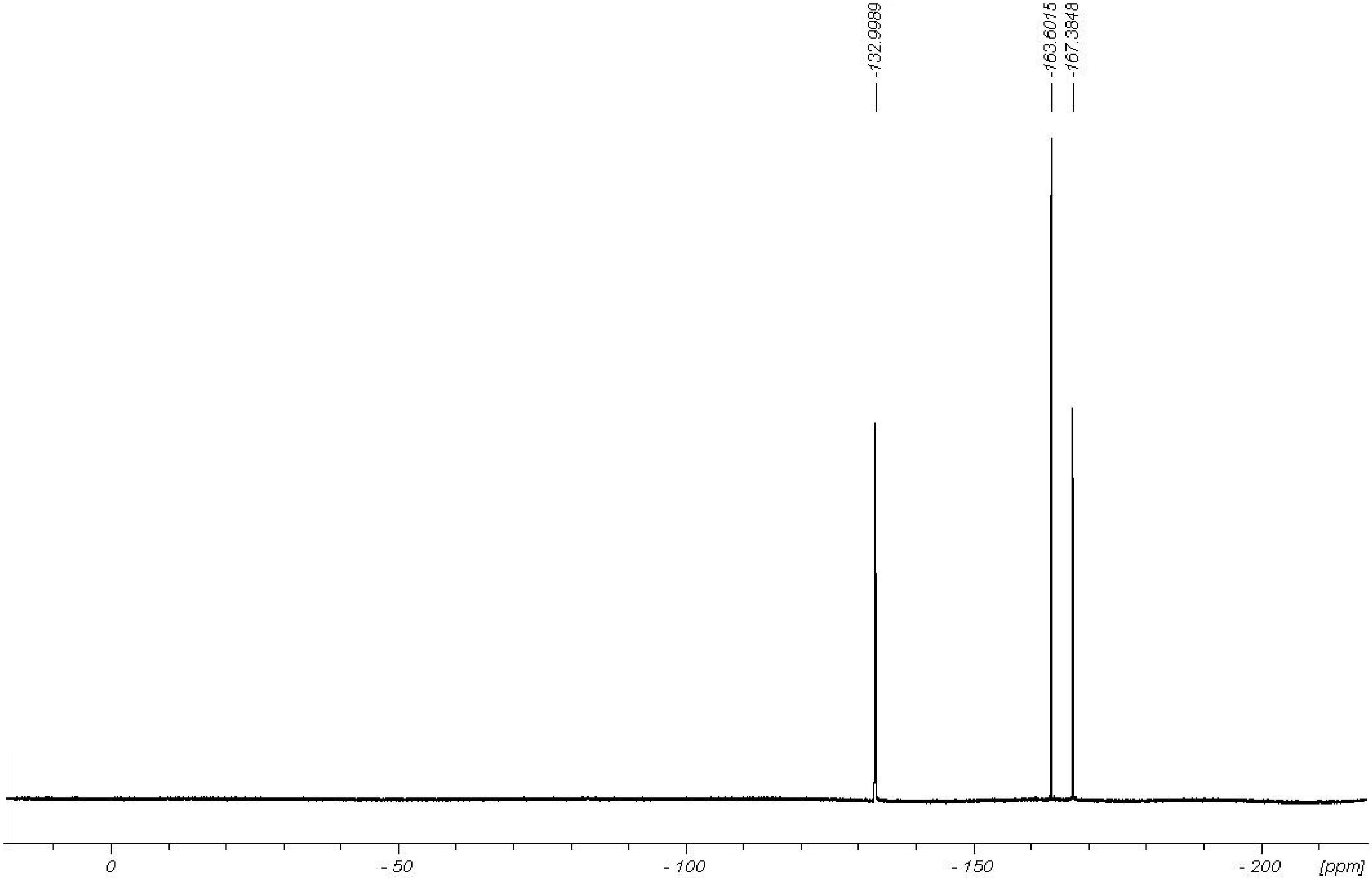

Figure S33: ^{29}Si INEPT NMR spectra (59.63 MHz) of **[(Me$_3$Si)$_3$S][B(C$_6$F$_5$)$_4$]** in 1,2-DCB.

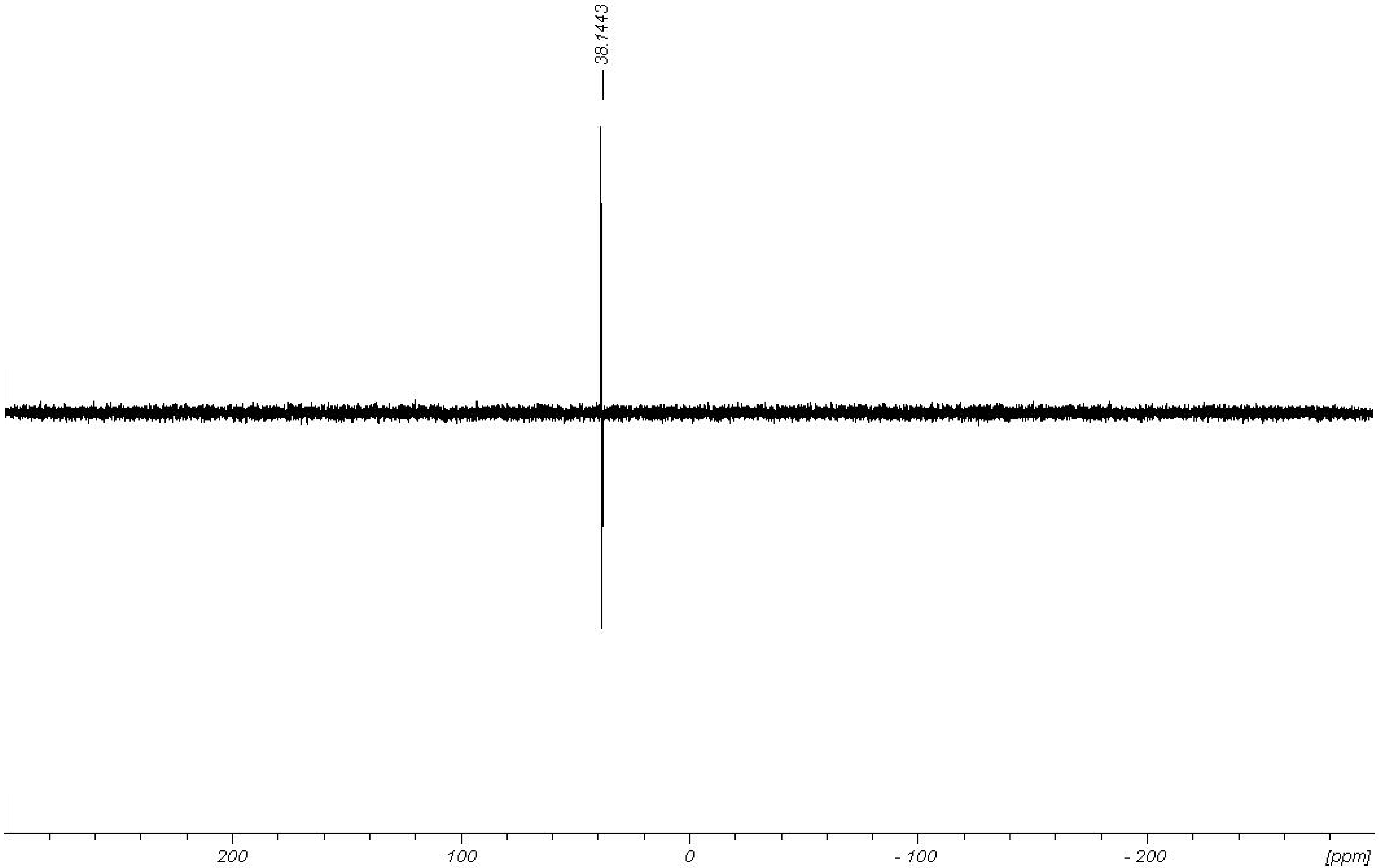

Figure S34: ^{1}H NMR spectra (300.13 MHz) of **$(Me_3Si)_2O$** in CD_2Cl_2 at 25 °C.

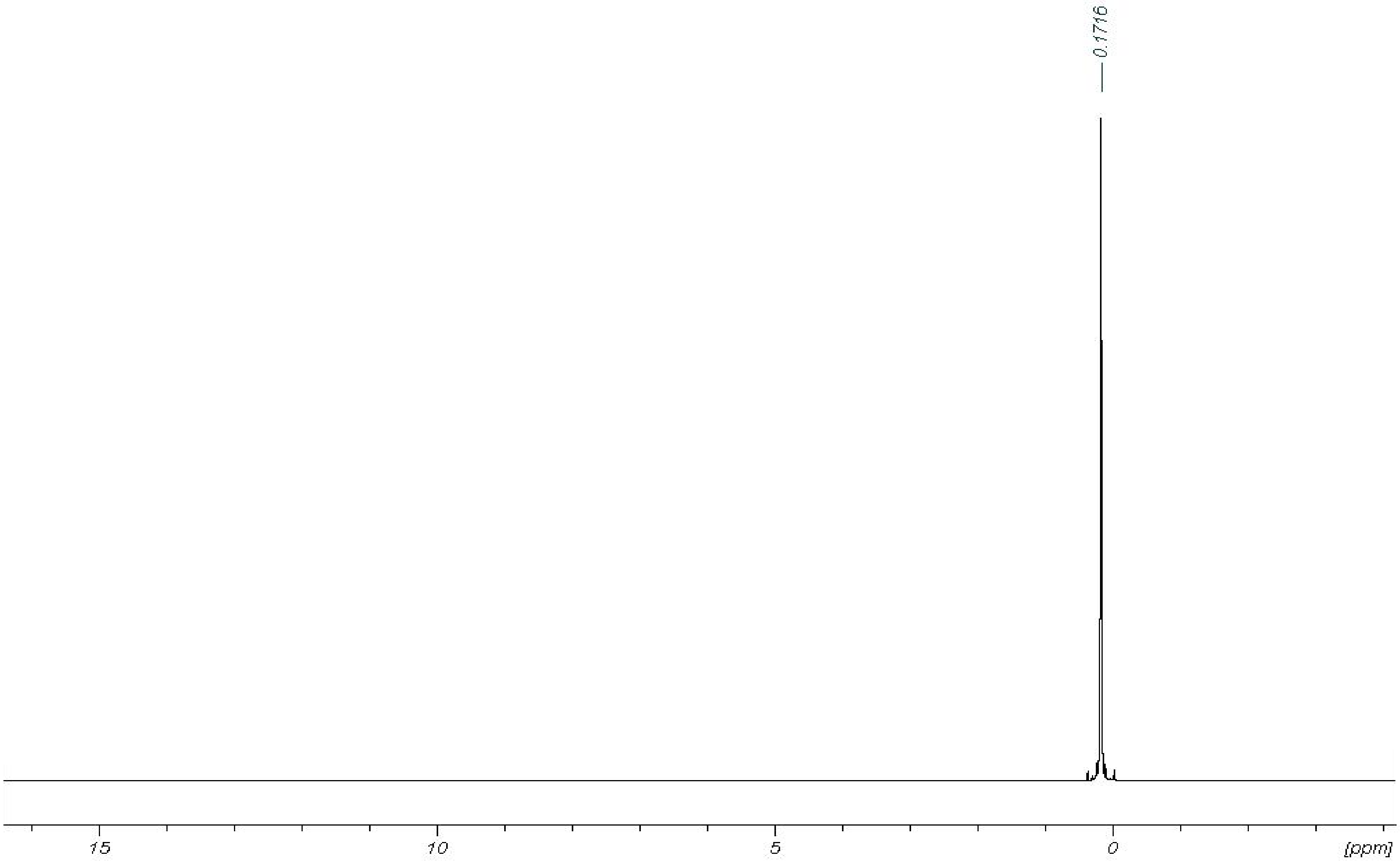

Figure S35: $^{13}C\{^{1}H\}$ NMR spectra (75.47 MHz) of **$(Me_3Si)_2O$** in CD_2Cl_2.

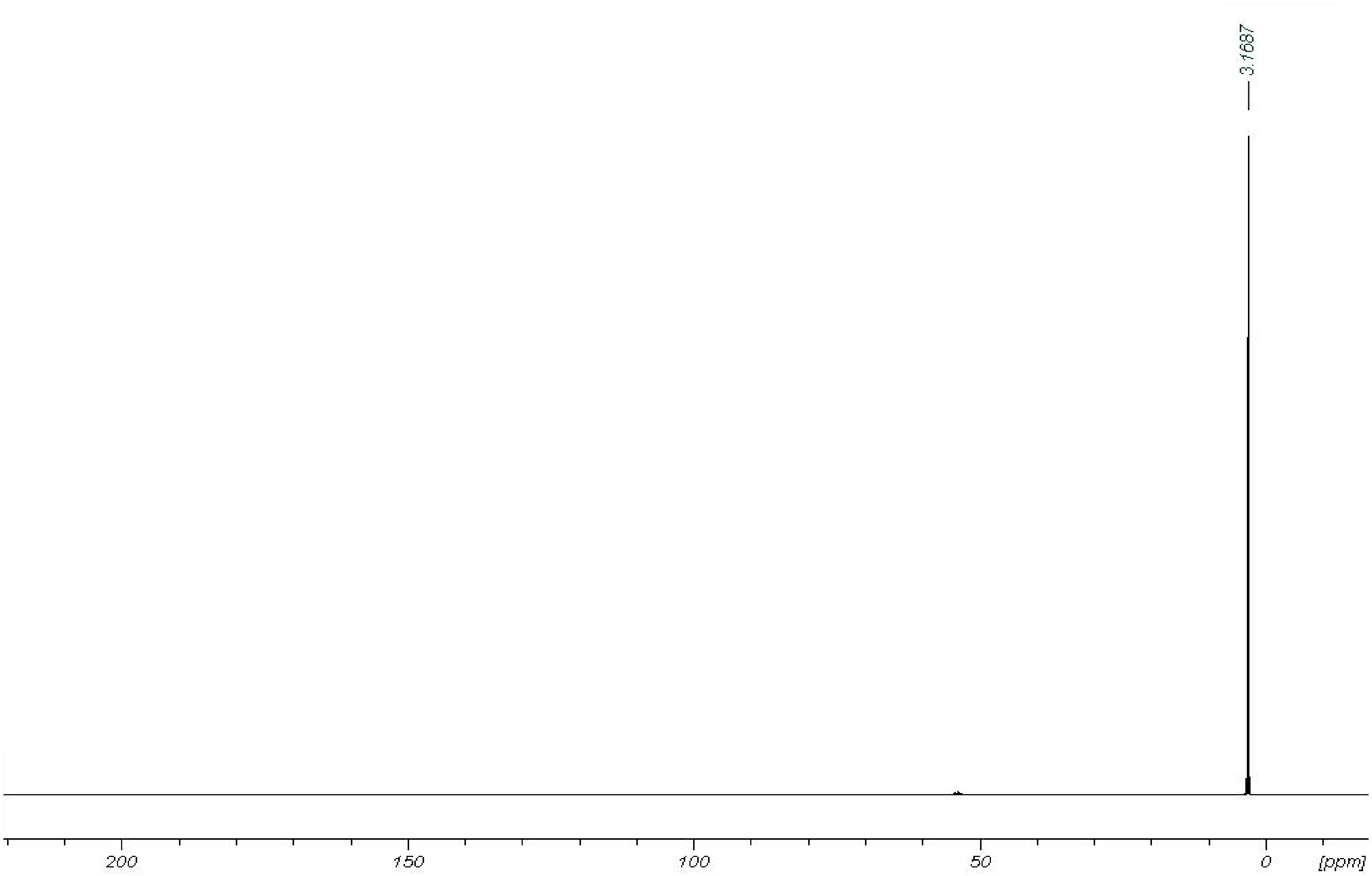

Figure S36: ^{29}Si INEPT NMR spectra (59.63 MHz) of **$(Me_3Si)_2O$** in CD_2Cl_2.

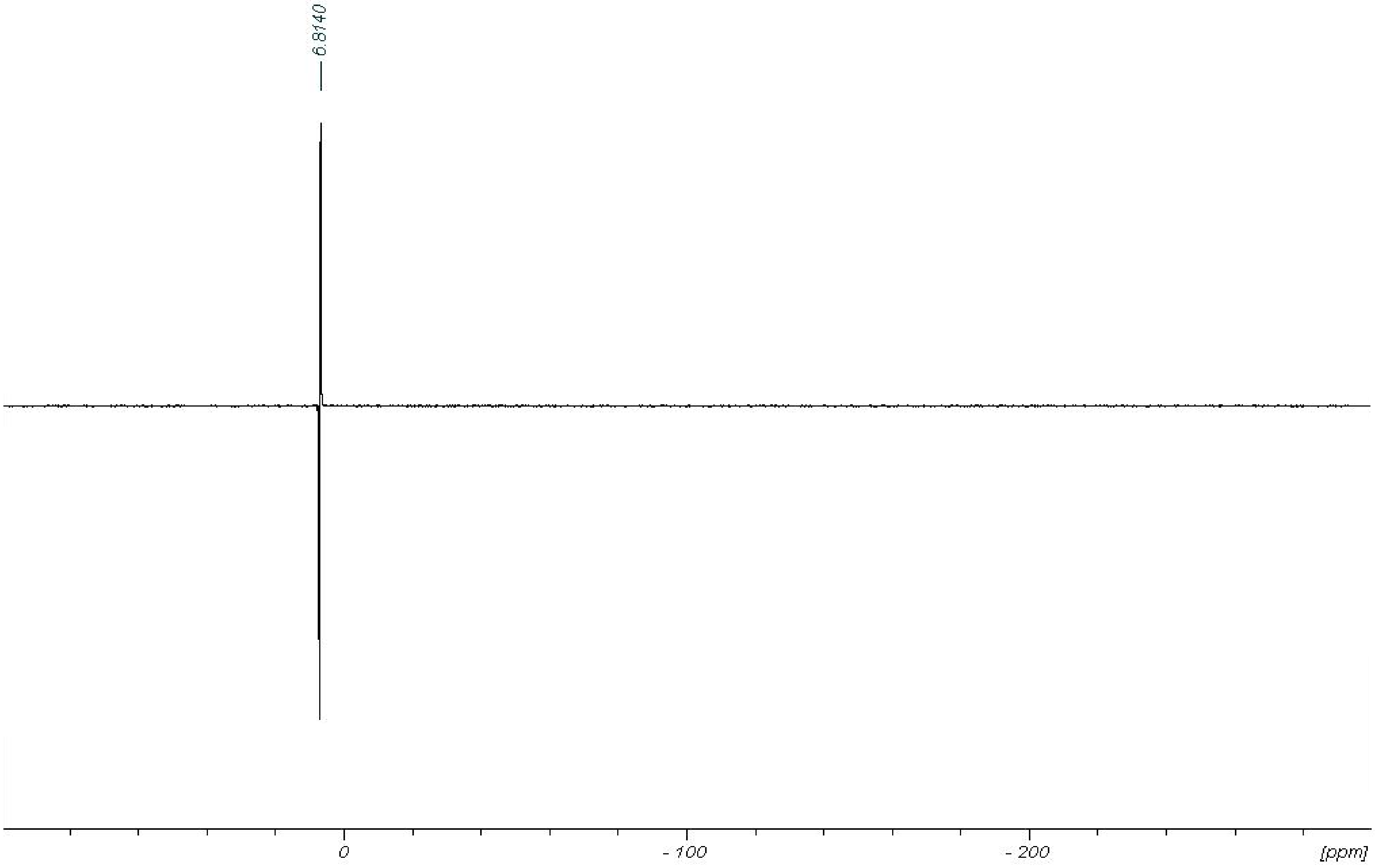

Figure S37: ^{17}O NMR spectra (67.82 MHz) of **$(Me_3Si)_2O$** in CD_2Cl_2.

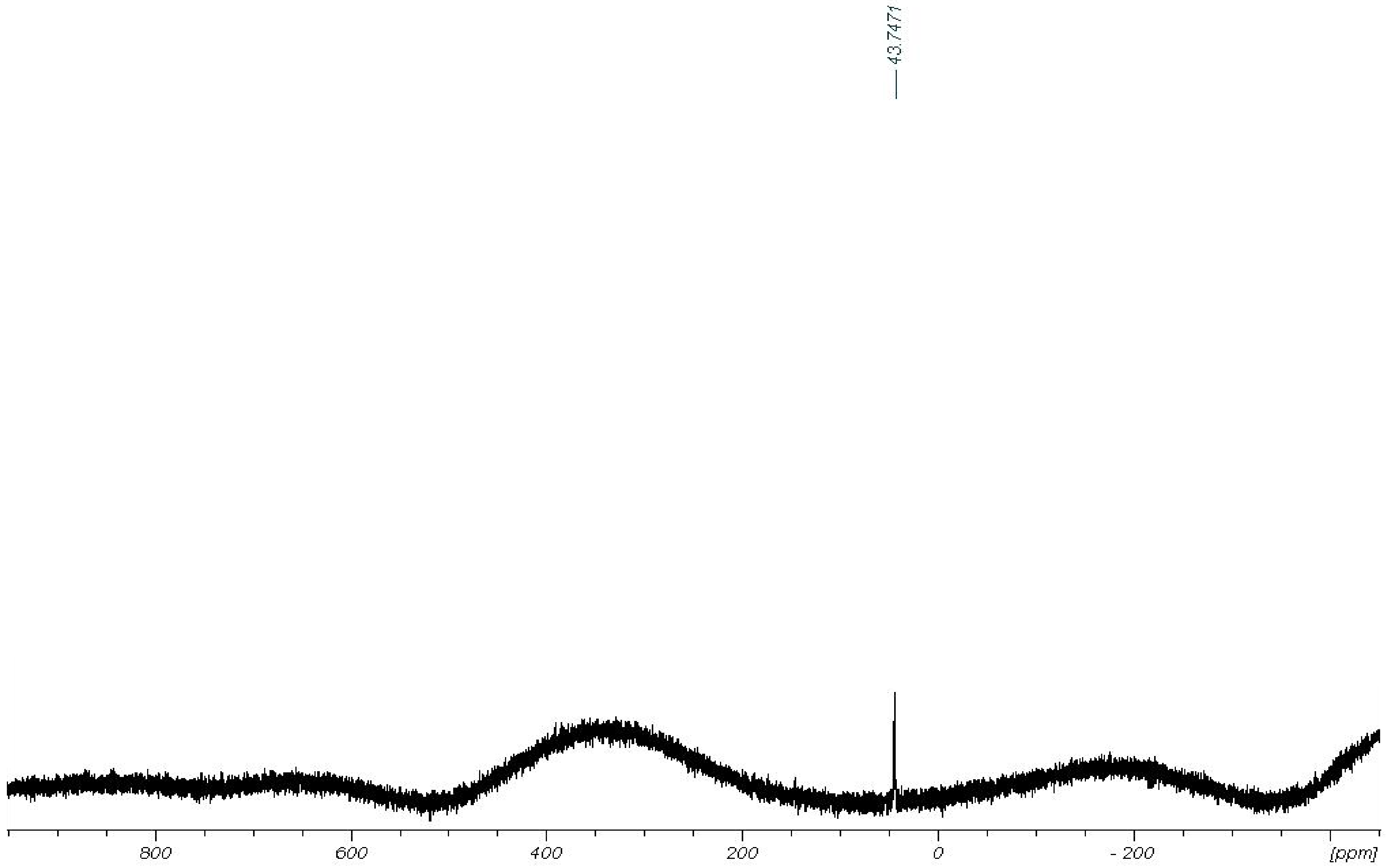

Figure S38: *NMR-spectra of [T_2(Me_2(H)Si)O][B(C_6F_5)$_4$] in CD_2Cl_2 (solvent signals indicated by asterisk, unknown impurities indicated plus).*

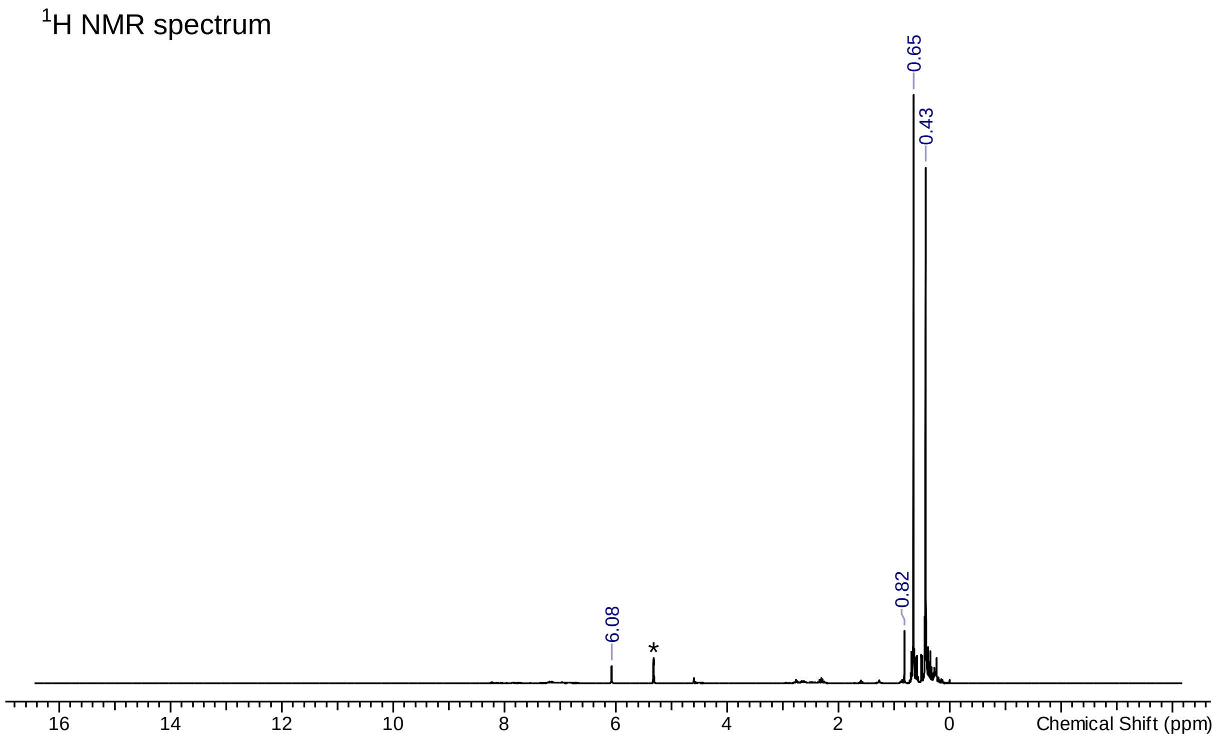

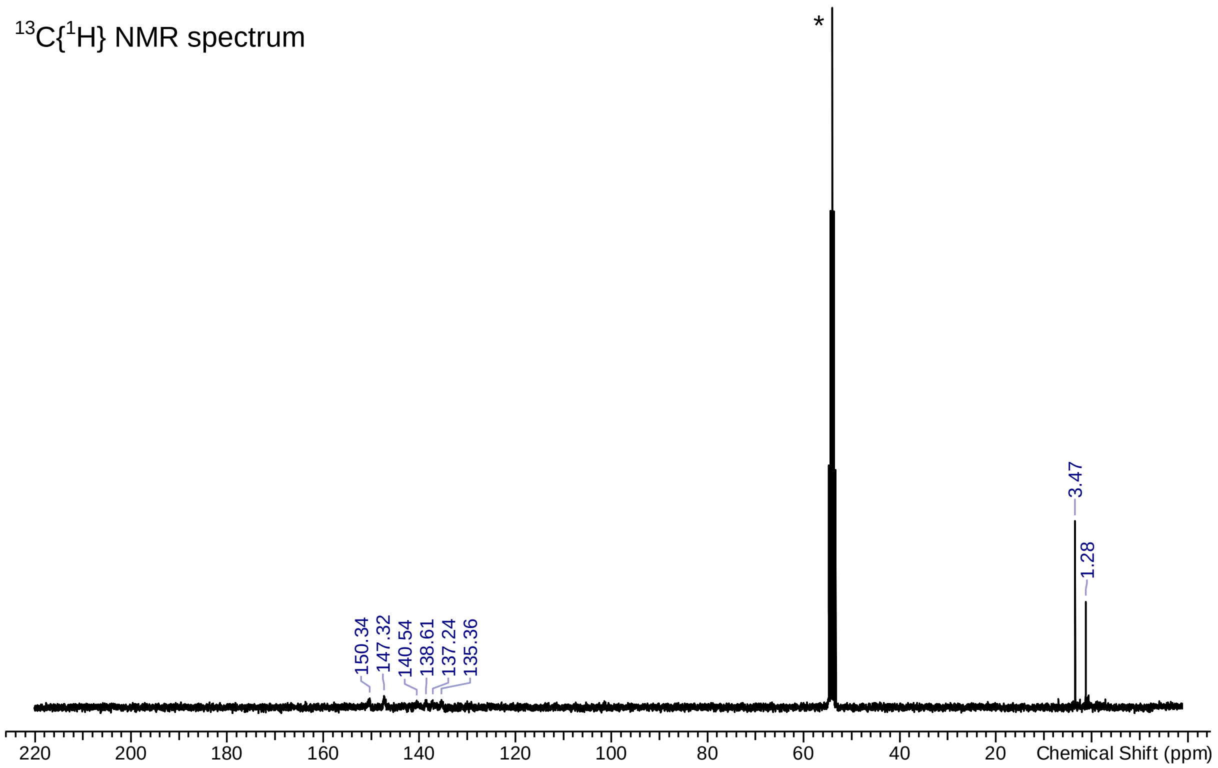

13C{1H} NMR spectrum
*
150.34
147.32
140.54
138.61
137.24
135.36
3.47
1.28
220
200
180
160
140
120
100
80
60
40
20
Chemical Shift (ppm)

^{11}B NMR spectrum

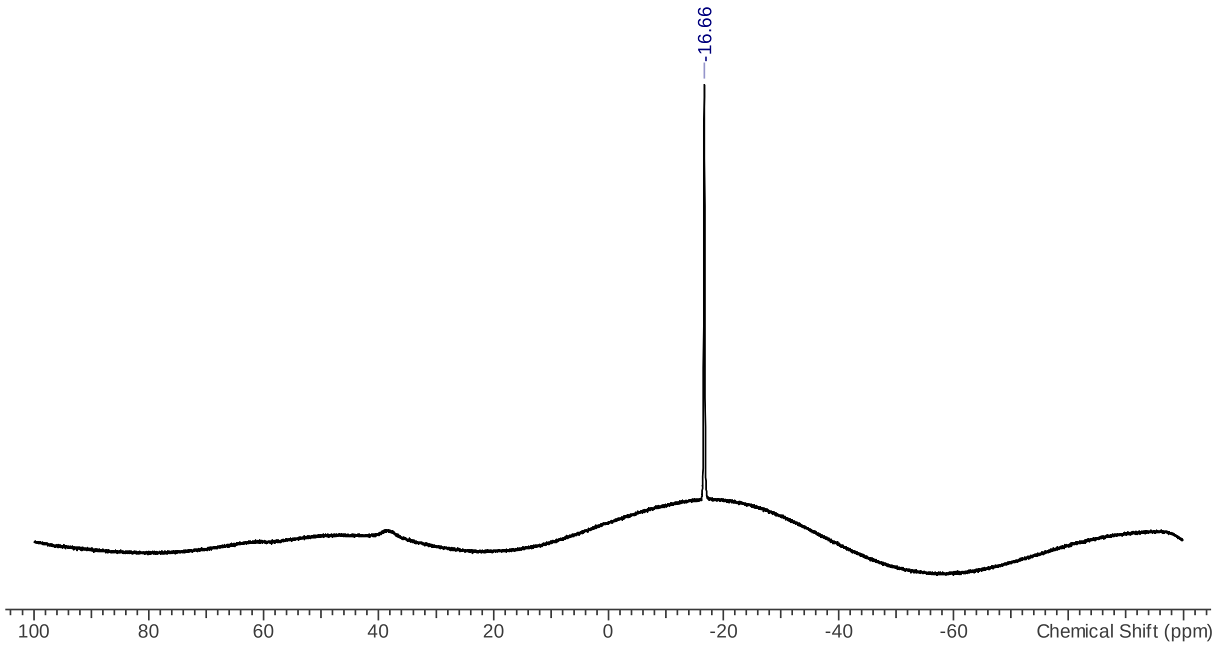

$^{19}F\{^1H\}$ NMR spectrum

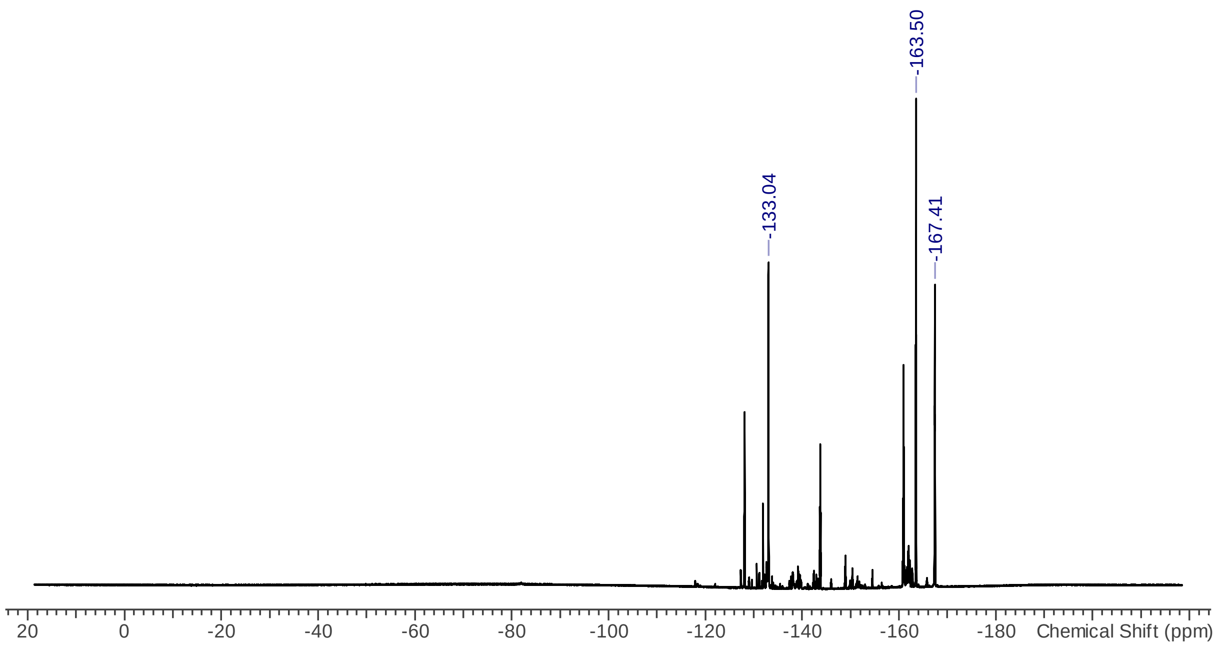

Figure S39: *NMR- and IR-spectra of $[Me_3Si\text{-}\mu\text{-}OSiMe_2]_2[CHB_{11}Cl_{11}]_2$ in DMSO-$[D_6]$ (solvent signals indicated by asterisk, unknown impurities indicated plus).*

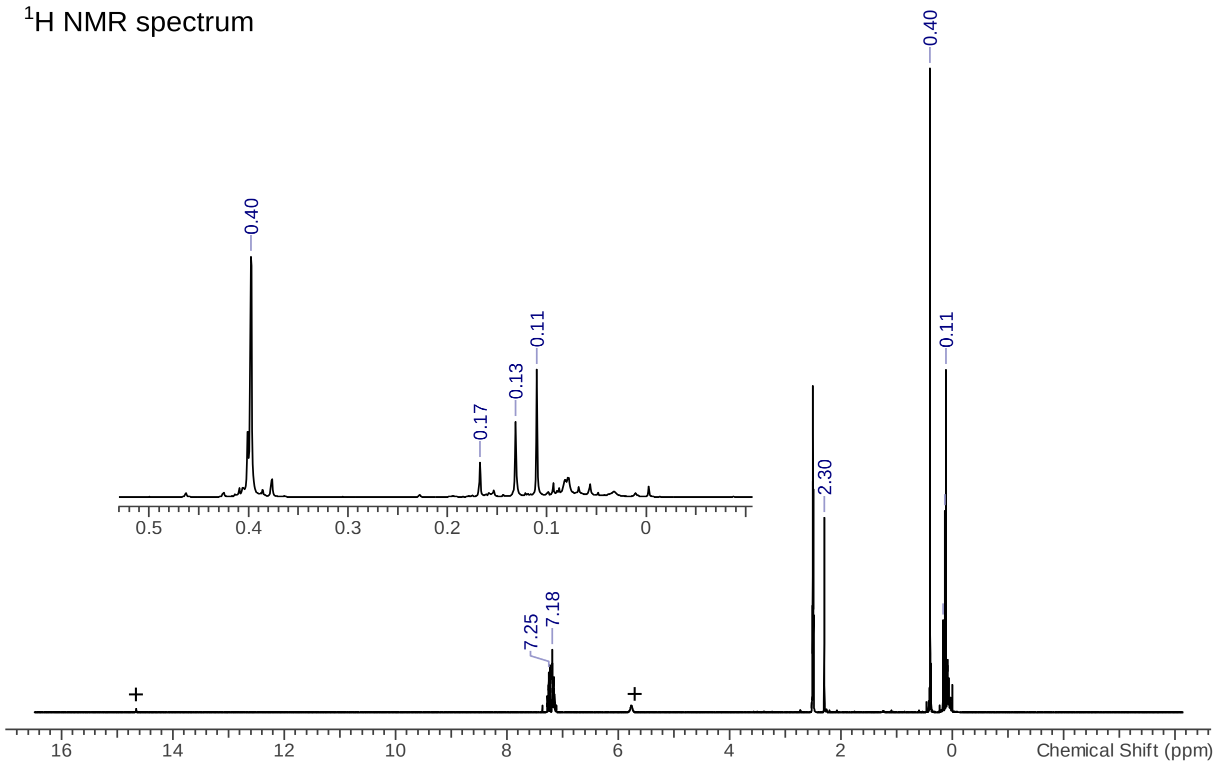

^{11}B NMR spectrum

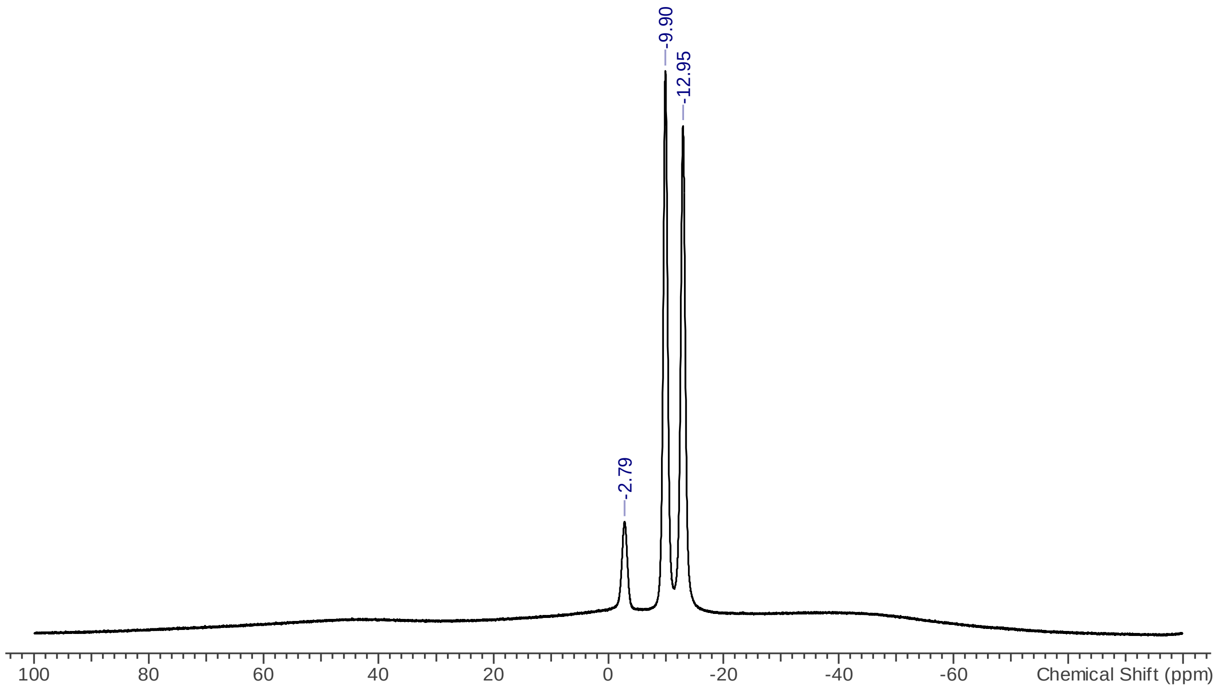

$^{13}C\{^{1}H\}$ NMR spectrum

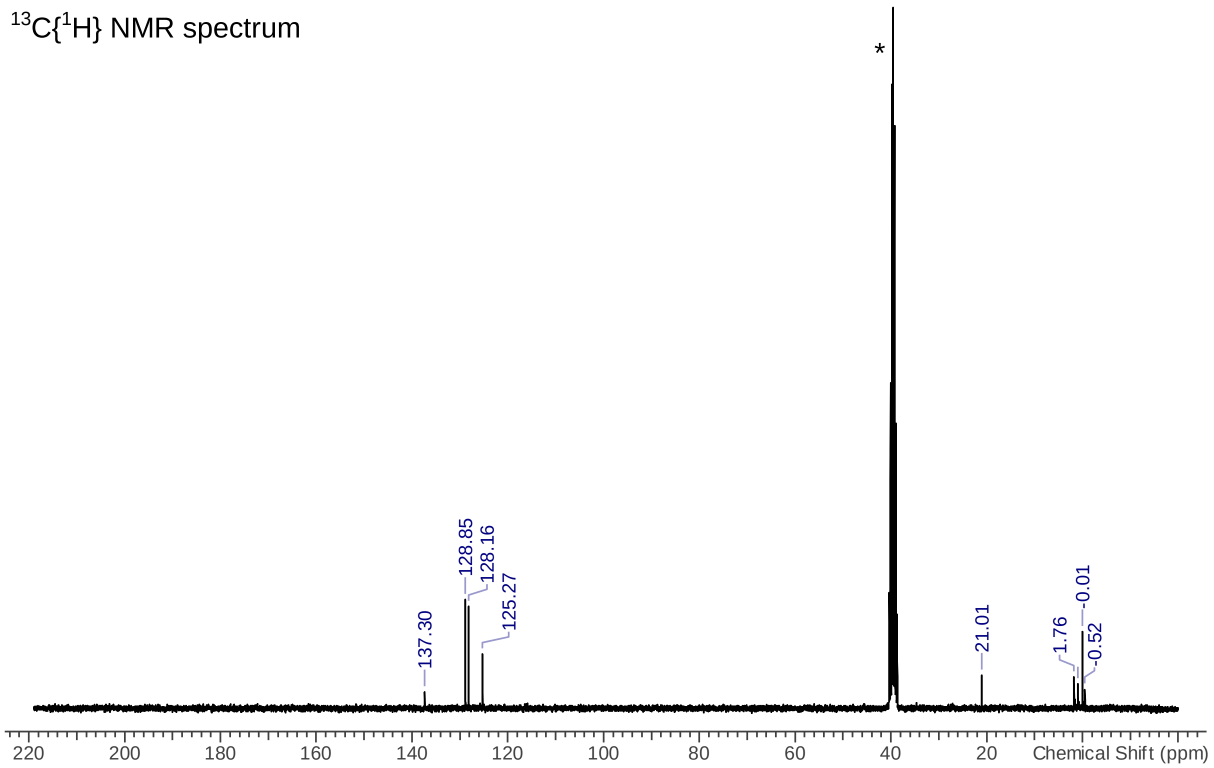

^{29}Si INEPT NMR spectrum

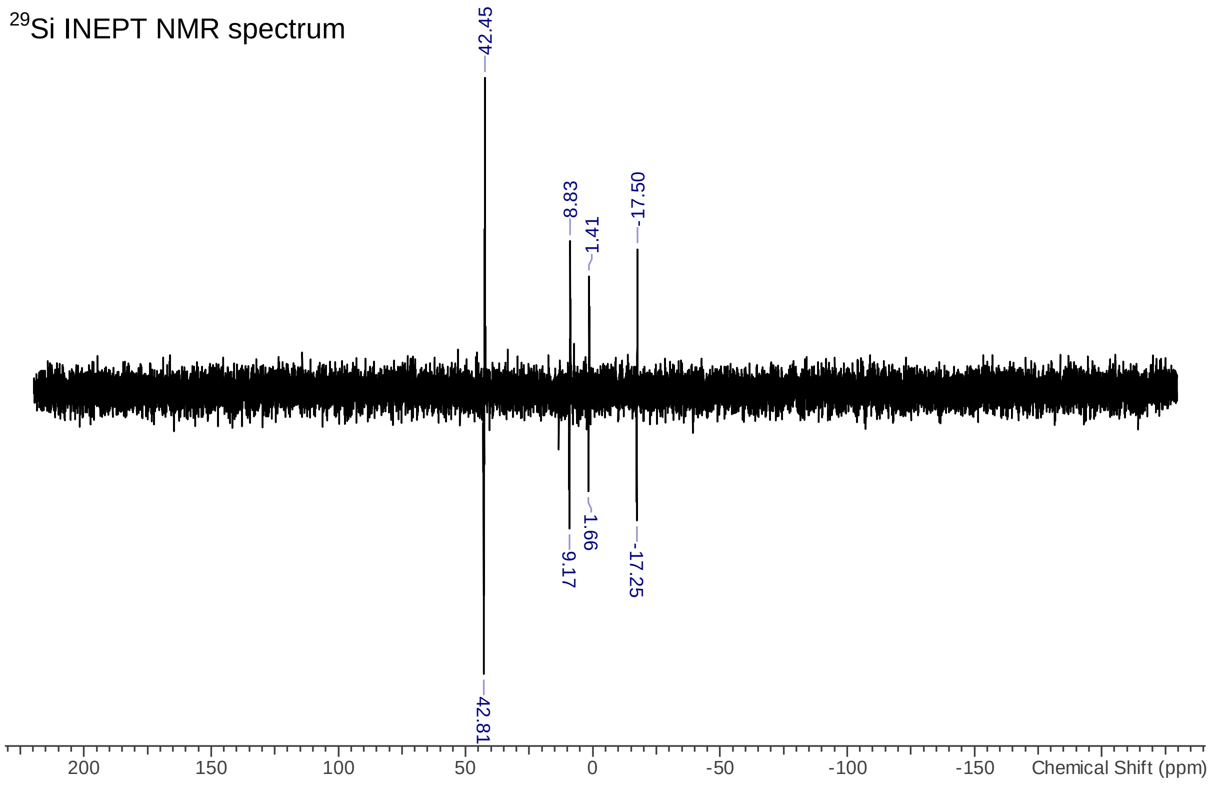

Figure S40: IR (red) and Raman (green) spectra of **$(Me_3Si)_2S$**.

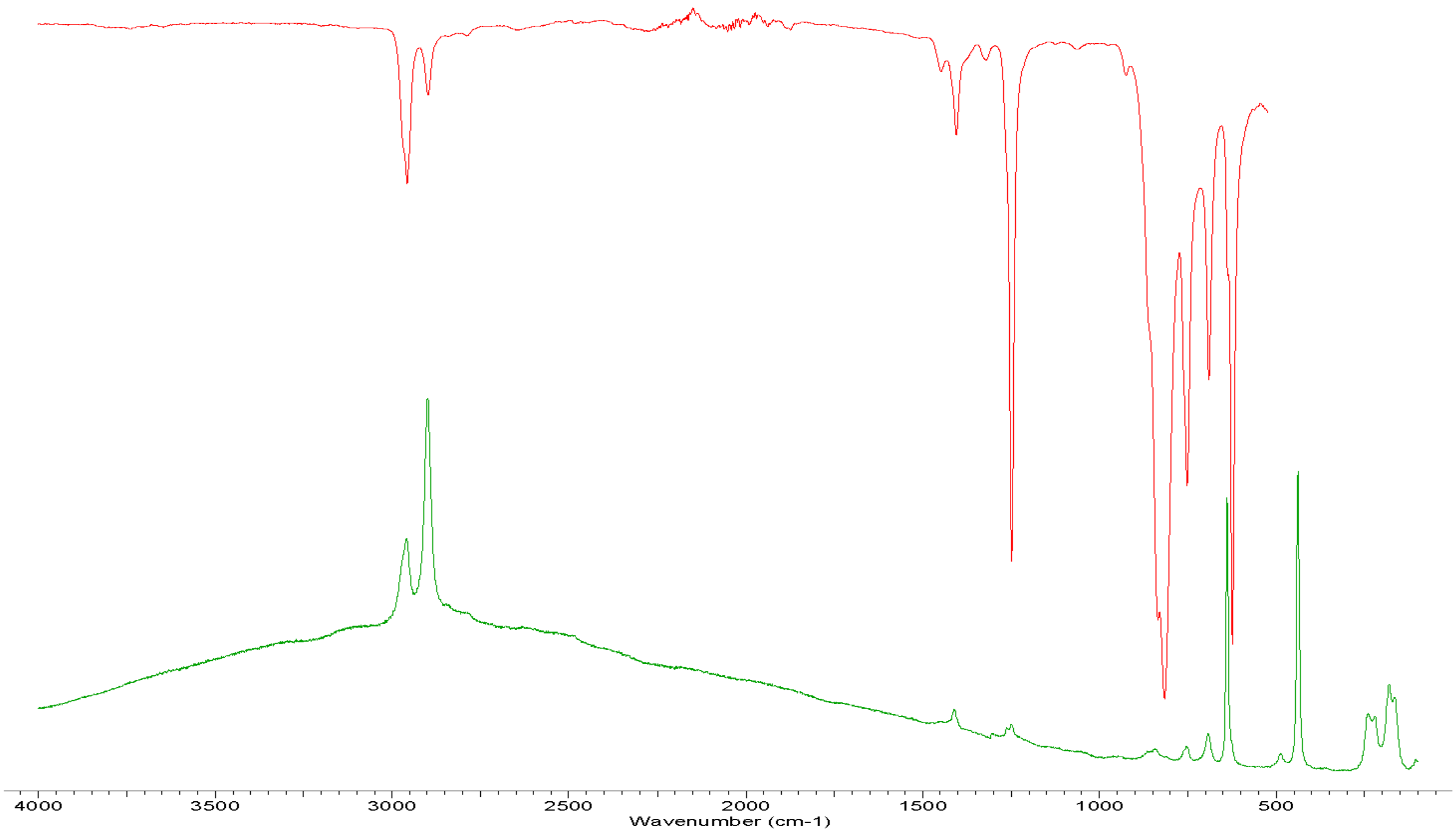

Figure S41: IR (red) and Raman (green) spectra of **[K·[18]crown-6][SSiMe$_3$]**.

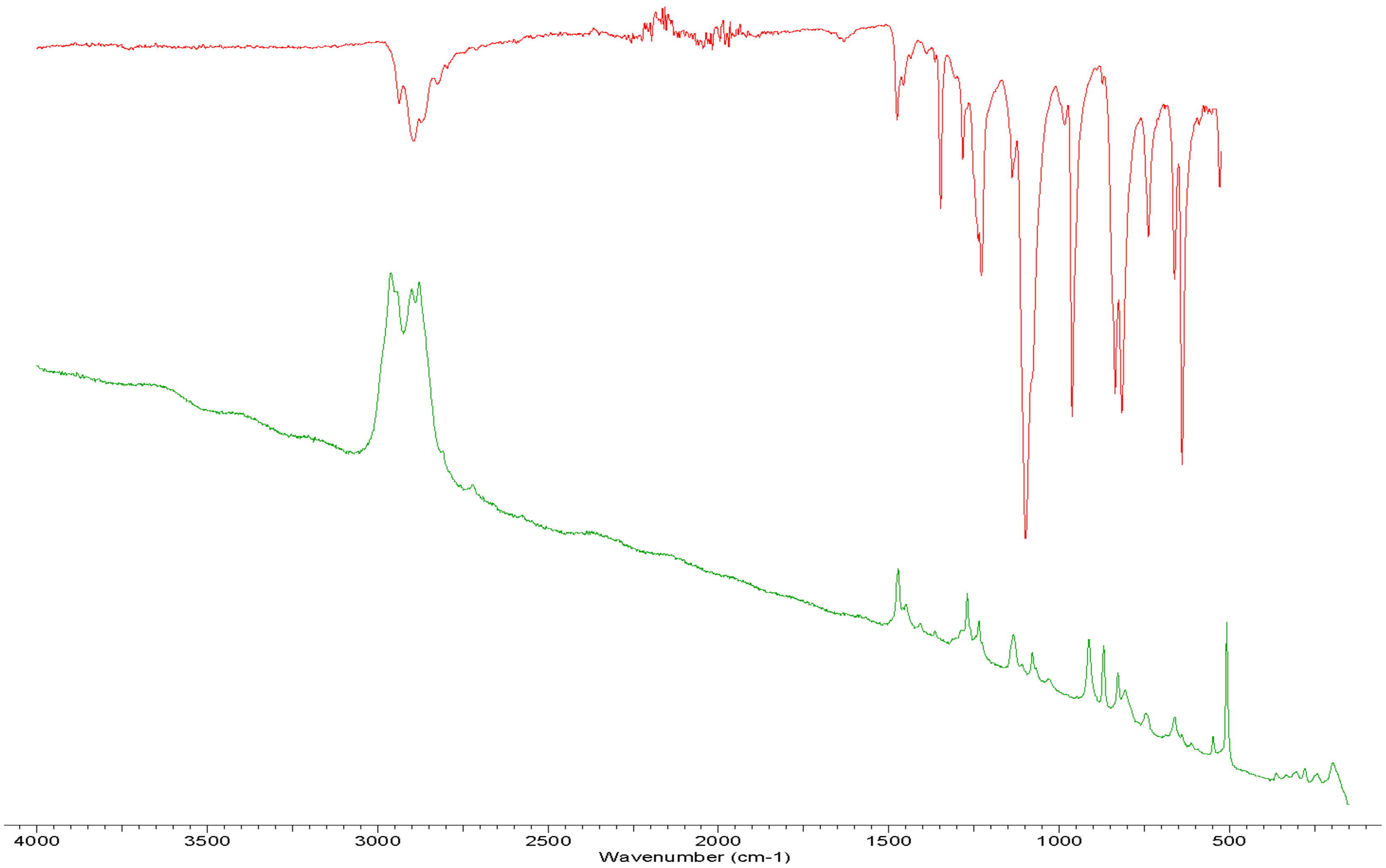

Figure S42: Raman and IR spectra of **[K·[18]crown-6][OSiMe$_3$]**.

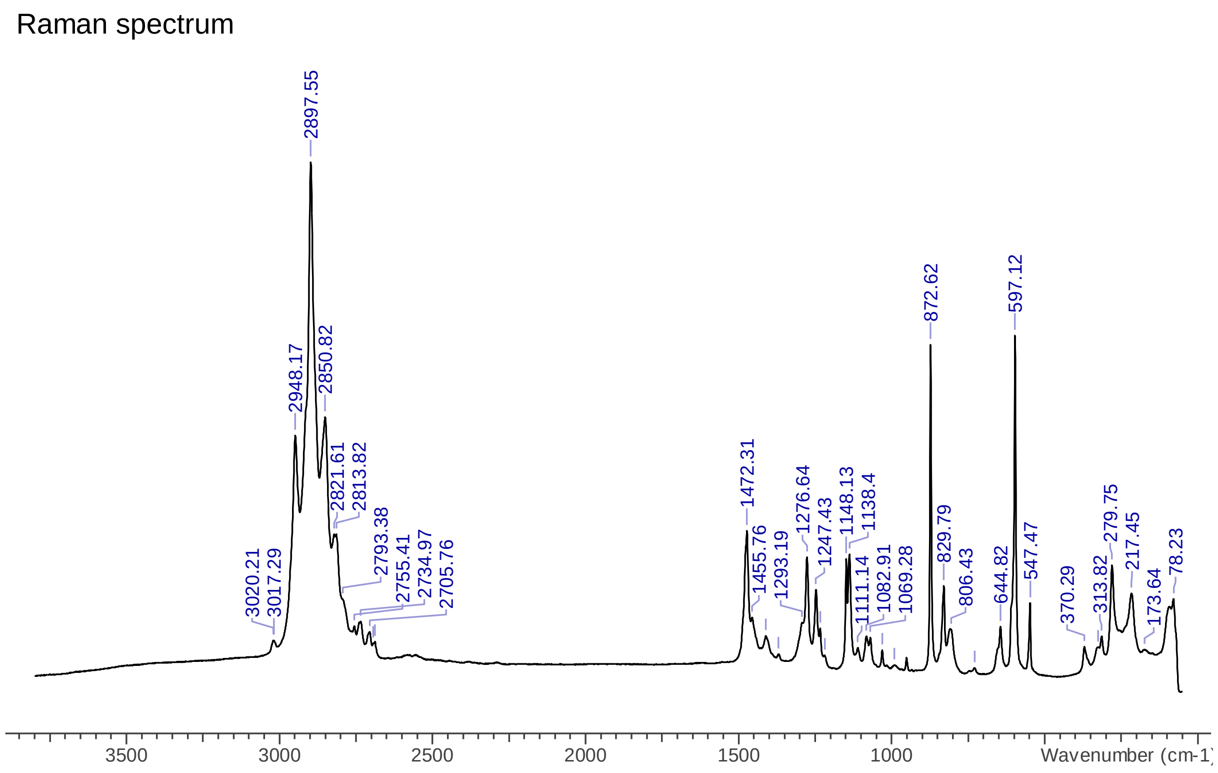

IR spectrum

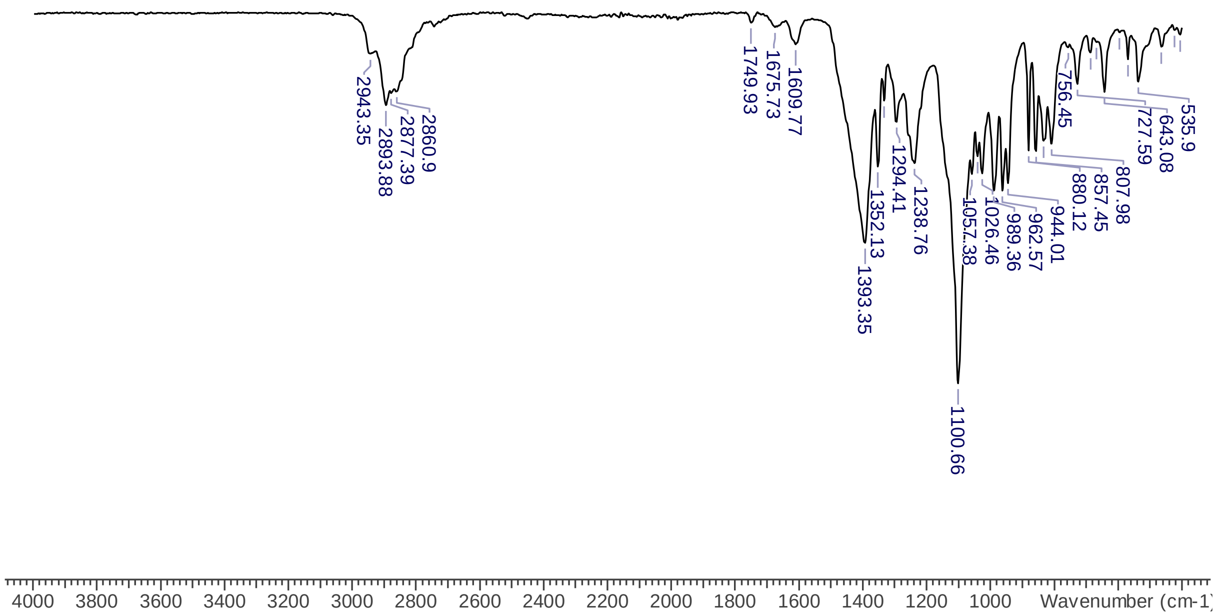
2943.35
2893.88
2877.39
2860.9
1749.93
1675.73
1609.77
1393.35
1352.13
1294.41
1238.76
1100.66
1057.38
1026.46
989.36
962.57
944.01
880.12
857.45
807.98
756.45
727.59
643.08
535.9
4000
3800
3600
3400
3200
3000
2800
2600
2400
2200
2000
1800
1600
1400
1200
1000
Wavenumber (cm-1)

Figure S43: IR spectra of **[$(Me_3Si)_3S$][$CHB_{11}H_5Cl_6$]**.

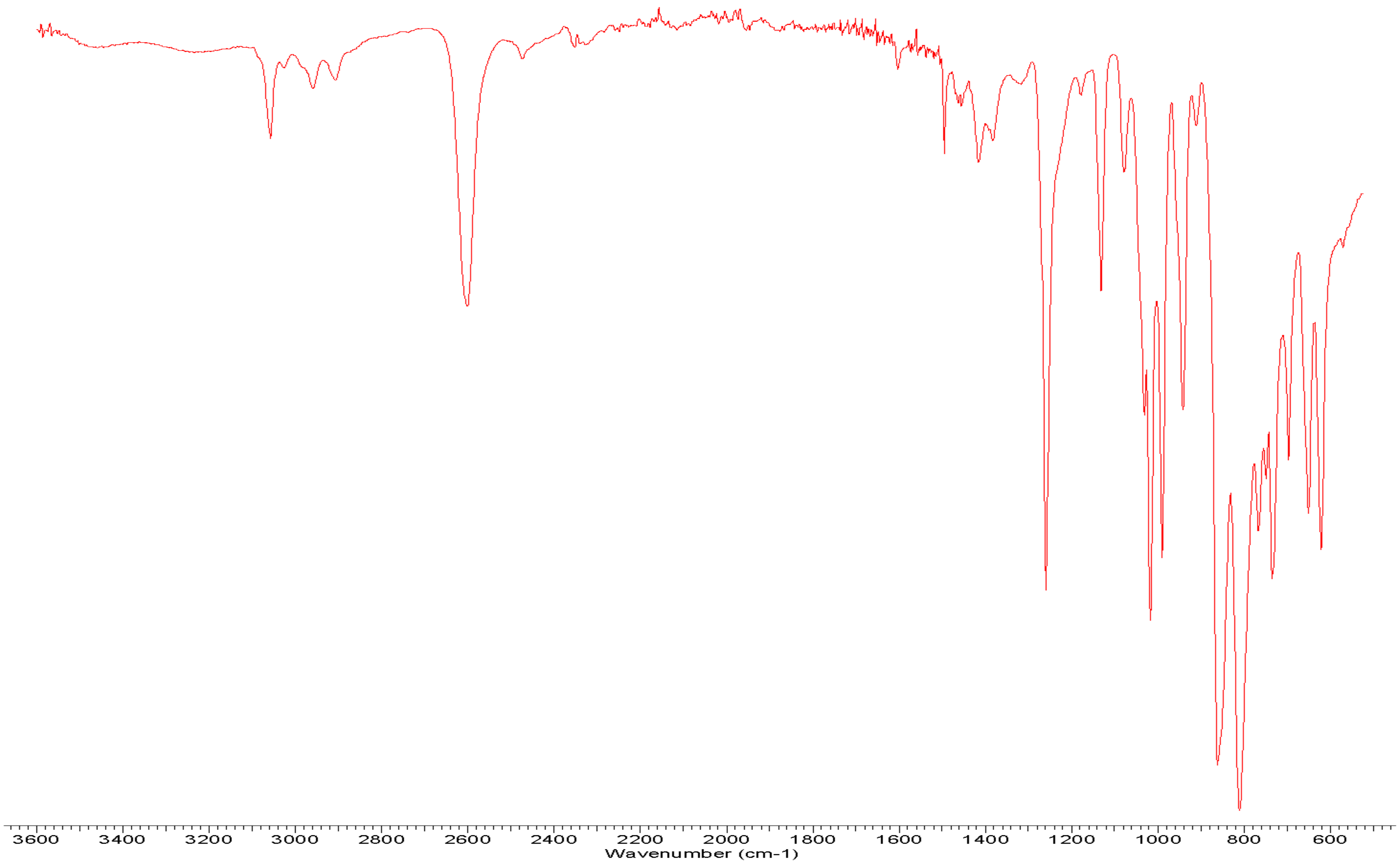

Figure S44: Raman spectra of **[$(Me_3Si)_3S$][$B(C_6F_5)_4$]**.

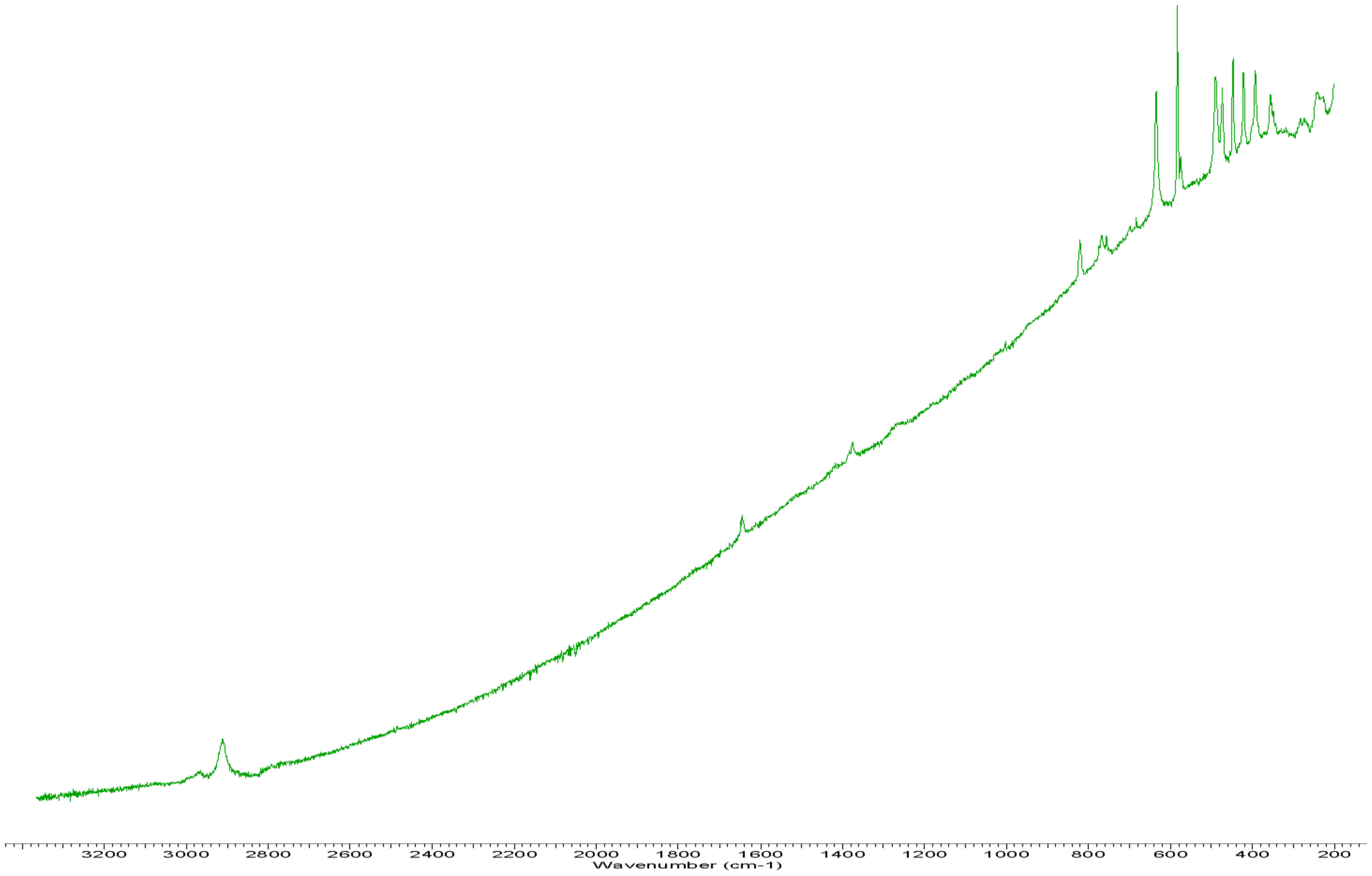

Figure S45: IR (red) and Raman (green) spectra of **$(Me_3Si)_2O$**.

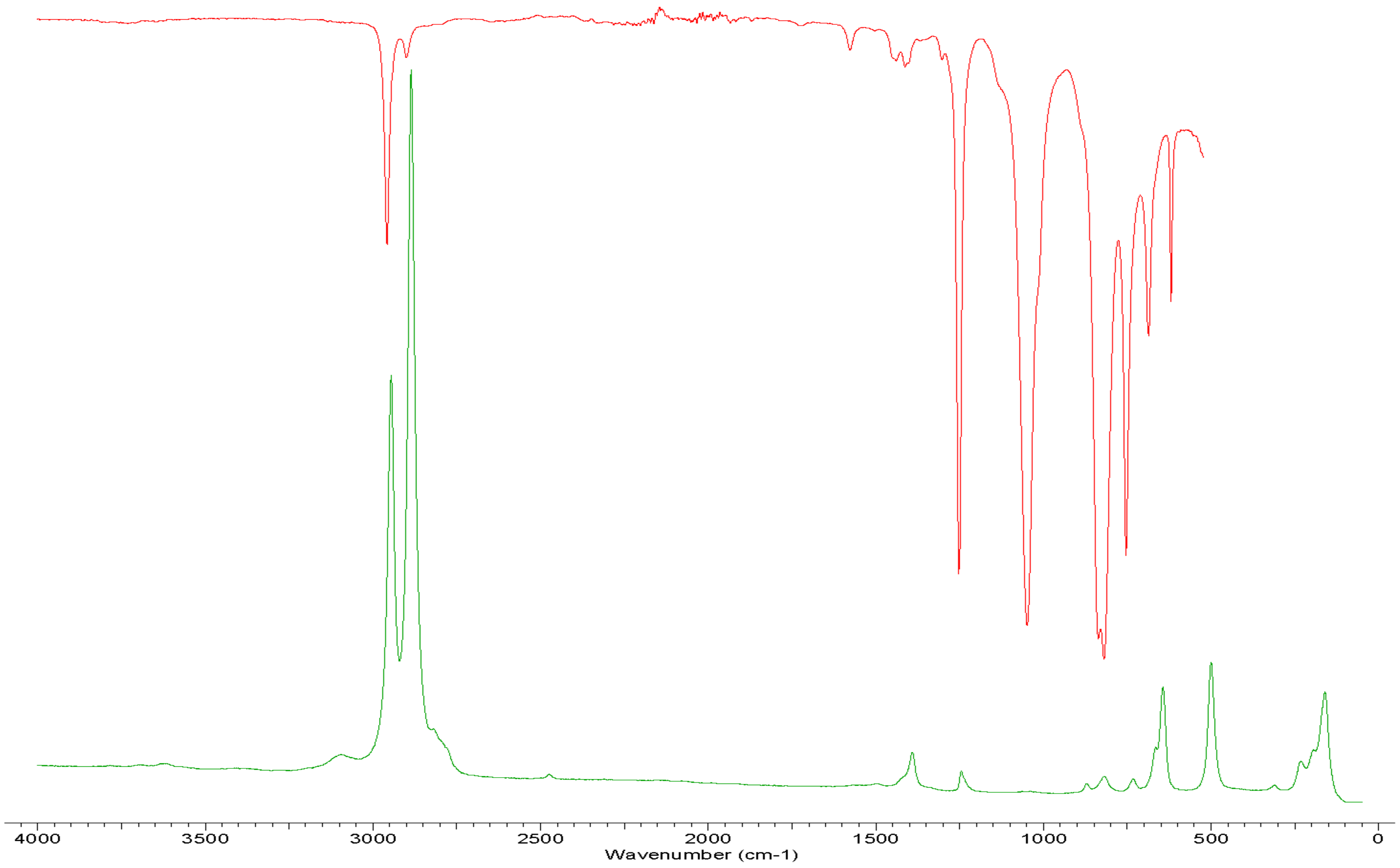

Figure S46: IR spectra of **[(Me$_3$Si)$_2$O(SiMe$_2$H)][B(C$_6$F$_5$)$_4$]**.Bands marked with an asterisk * appear due to hydrolysis.

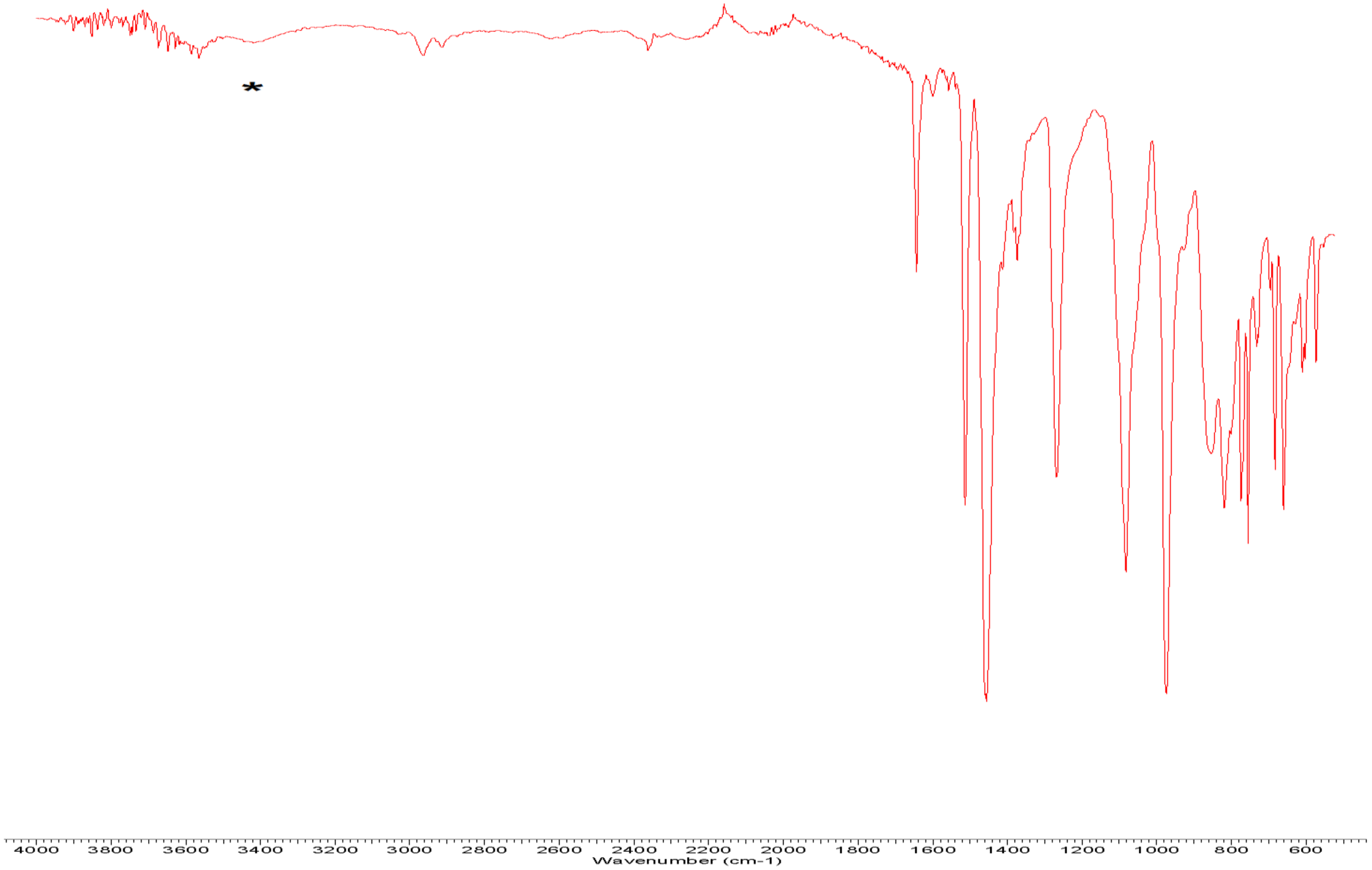

Figure S47: *IR-spectra of $[Me_3Si$-µ-$OSiMe_2]_2[CHB_{11}Cl_{11}]_2$ in DMSO-$[D_6]$ (solvent signals indicated by asterisk, unknown impurities indicated plus).*

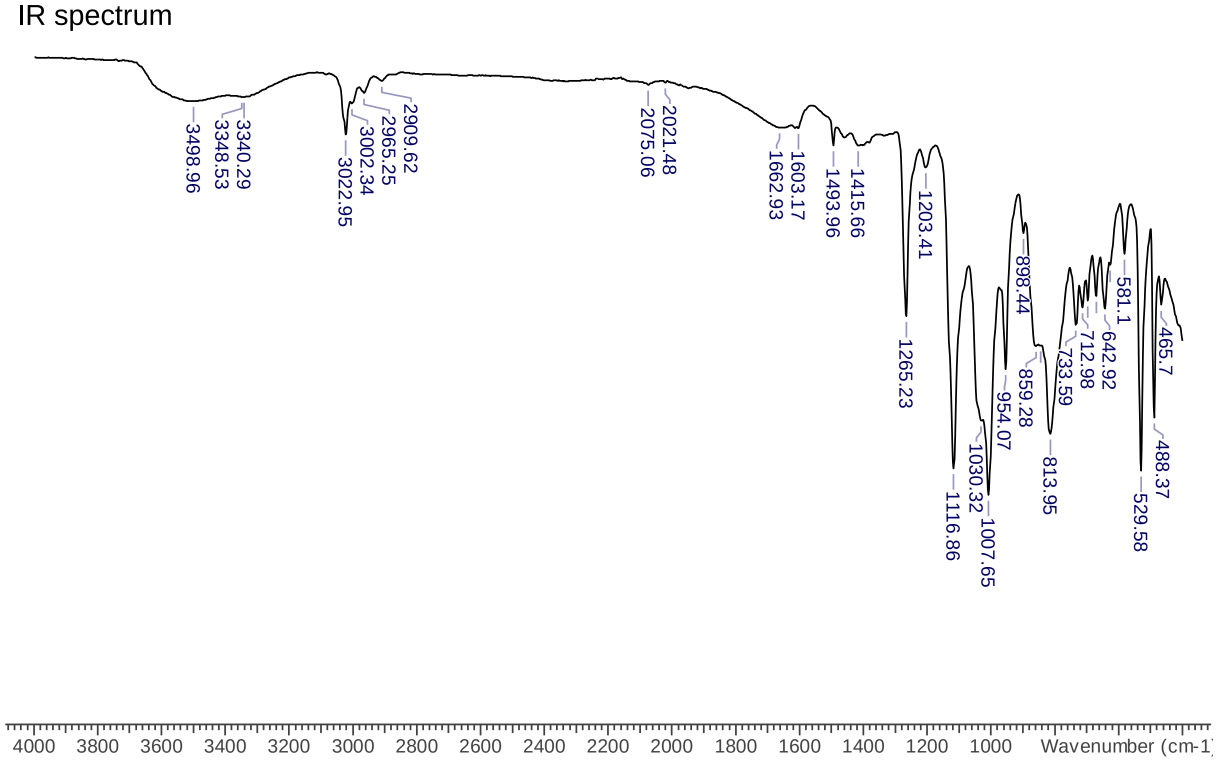

6 Computational Details

All computations were carried out using Gaussian09 [14] as well as the standalone version of NBO 6.0. [15-23]

Methods. DFT calculations were carried out using the hybrid DFT functional PBE0 including Grimme's dispersion model D3BJ [24-25] in conjunction with the aug-cc-pVDZ or def2svp basis set. All structures were fully optimized and confirmed as minima by frequency analyses (NIMAG = 0). To obtain reliable estimates of the structures, activation barriers (NIMAG = 1), potential energy surfaces and Gibbs free energies of the reactions involving the carborate anion $[CHB_{11}Cl_{11}]^{-}$, the corresponding species were optimized at the PBE0−D3BJ/def2svp level of theory, while the larger basis set aug-cc-pVDZ was only applied for the ligand scrambling process. To localize the transition states as well as other stationary points ***many*** scans have been carried out, these data (energies and associated structures) are not listed in the ESI – these data can be obtained by the authors.

Population analysis – Steric effects. Partial charges, Lewis representations, delocalization and polarization effects etc. were computed using the NBO 6.0 program (NBO, NRT, NBO deletion) at the PBE0−D3BJ/ def2svp level of theory. [15-17, 19,21-23] To study the wave function as well as the Shubin Liu energy decomposition analysis,[13] 26 we used the MULTIWFN by Tian Lu.[27]

Energies

Ligand Scrambling

Lewel of theory: the PBE1PBE/AUG-cc-pVDZ empiricaldispersion = gd3bj

Table S4. Absolute energies in a.u. for all species involved in the ligand scrambling process.

	E	H	G	NImag
[SiMe3](+)	-408.719388	-408.601961	-408.643111	0
[HSiMe2](+)	-369.415610	-369.328876	-369.364718	0
[H2SiMe](+)	-330.108531	-330.052357	-330.083212	0
[Me3Si-H-SiMe3](+)	-818.378326	-818.130662	-818.190961	0
[Me3Si-Me-Si(H)Me2](+)	-818.357819	-818.110452	-818.170538	0
TS_H -> Me-bridge	-818.341356	-818.095078	-818.153062	1
**TS_Me->HSiMe2(+) + Me4Si**	**no barrier**			
**TS_Me->SiMe3(+) + Me3SiH**	**no barrier**			
**TS_H -> SiMe3(+) + HSiMe3**	**no barrier**			
[Me2(H)Si-H-SiMe3](+)	-779.085350	-778.867958	-778.924290	0
Me2H2Si	-370.312325	-370.216031	-370.250486	0
HSiMe3	-409.597991	-409.471228	-409.509911	0
SiMe4	-448.884220	-448.727085	-448.769812	0
Me3Si-O-SiMe3	-893.273073	-893.029442	-893.088281	0
[(Me3Si)3O](+)	-1302.081973	-1301.715675	-1301.788811	0
[(Me3Si)2(Me2SiH)O](+)	-1262.796551	-1262.461067	-1262.532580	0
Me3Si-S-SiMe3	-1216.159869	-1215.917588	-1215.977527	0
[(Me3Si)3S](+)	-1624.978312	-1624.613982	-1624.689431	0
[(Me3Si)2(Me2SiHS](+)	-1585.688117	-1585.354588	-1585.428385	0

Table S5. Reaction energies in kcal/mol

**scrambling reactions first step**	DE	DH	DG	
[Me3Si-H-SiMe3](+) = [SiMe3](+) + HSiMe3	38.24	36.06	23.81	**delDG**
[Me3Si-H-SiMe3](+) = [HSiMe2](+) + SiMe4	49.26	46.88	35.41	11.60
[Me3Si-Me-SiMe3](+) = [SiMe3](+) + HSiMe3	25.38	23.38	10.99	**delDG**
[Me3Si-Me-SiMe3](+) = [HSiMe2](+) + SiMe4	36.39	34.19	22.60	11.60

Table S6. Reaction energies in kcal/mol

silylation reactions first step	DE	DH	DG	
[Me3Si](+) + Me3Si-O-SiMe3 = [(Me3Si)3O](+)	-56.17	-52.88	-36.03	**delDG**
[Me2(H)Si](+) + Me3Si-O-SiMe3 = [(Me3Si)2(Me2SiH)O](+)	-67.69	-64.48	-49.94	-13.91
[Me3Si](+) + Me3Si-S-SiMe3 = [(Me3Si)3S](+)	-62.16	-59.26	-43.17	
[Me2(H)Si](+) + Me3Si-S-SiMe3 = [(Me3Si)2(Me2SiH)S](+)	-70.68	-67.85	-54.05	-10.89
[Me3Si-H-SiMe3](+) + Me3Si-O-SiMe3 = [(Me3Si)3O](+) + HSiMe3	-17.93	-16.82	**-12.22**	**delDG**
[Me3Si-H-SiMe3](+) + Me3Si-O-SiMe3 = [(Me3Si)2(HSiMe2)O](+) + SiMe4	-18.43	-17.60	**-14.53**	-2.30
[Me3Si-H-SiMe3](+) + Me3Si-S-SiMe3 = [(Me3Si)3S](+) + HSiMe3	-23.91	-23.19	**-19.36**	
[Me3Si-H-SiMe3](+) + Me3Si-S-SiMe3 = [(Me3Si)2(HSiMe2)S](+) + SiMe4	-21.42	-20.97	**-18.64**	0.72

Table S7. Reaction energies in kcal/mol

scrambling reactions second step	DE	DH	DG	
[Me2(H)Si-H-SiMe3](+) = [SiMe3](+) + H2SiMe2	33.66	31.35	19.26	**delDG**
[Me2(H)Si-H-SiMe3](+) = [HSiMe2](+) + HSiMe3	45.02	42.58	31.16	11.90
[Me2(H)Si-H-SiMe3](+) = [H2SiMe](+) + SiMe4	58.11	55.54	44.72	25.46

Table S8. Reaction energies in kcal/mol (see Scheme 3 in the manuscript)

gas phase	DE	DH	DG
A->7: [Me3Si](+) + Me3SiH = [Me3Si-H-SiMe3](+)	-38.24	-36.06	-23.81
A'->7: [Me3Si](+) + Me3SiH = [Me3Si-H-SiMe3](+)	-38.24	-36.06	-23.81
B 7->8: [Me3Si-H-SiMe3](+) = [Me3Si-Me-Si(H)Me2](+)	12.87	12.68	12.82
C : [Me3Si-Me-Si(H)Me2](+) = [Me3Si](+) + Me3SiH	25.38	23.38	10.99
D: [Me3Si-Me-Si(H)Me2](+) = [Me2(H)Si](+) + Me4Si	36.39	34.19	22.60
E-> 9: [Me2(H)Si](+) + Me3SiH = [Me3Si-H-Si(H)Me2](+)	-45.02	-42.58	-31.16
F->9: [Me3Si](+) + Me2SiH2 = [Me3Si-H-Si(H)Me2](+)	-33.66	-31.35	-19.26

Table S9. Reaction energies in kcal/mol (see Scheme 3 in the manuscript)

toluene adducts	DE	DH	DG
A->7: [Me3Si_tol](+) + Me3SiH = [Me3Si-H-SiMe3](+) + tol	0.93	0.83	-0.35
A'->7: [Me3Si_tol](+) + Me3SiH = [Me3Si-H-SiMe3](+) + tol	0.93	0.83	-0.35
B 7->8: [Me3Si-H-SiMe3](+) + tol = [Me3Si-Me-Si(H)Me2](+) +tol	12.87	12.68	12.82
C: [Me3Si-Me-Si(H)Me2](+) + tol = [Me3Si_tol](+) + Me3SiH	-13.79	-13.51	-12.46
D: [Me3Si-Me-Si(H)Me2](+) + tol = [Me2(H)Si_tol](+) + Me4Si	-10.34	-10.06	-8.56
E-> 9: [Me2(H)Si_tol](+) + Me3SiH = [Me3Si-H-Si(H)Me2](+) + tol	1.70	1.68	0.00
F->9: [Me3Si_tol](+) + Me2SiH2 = [Me3Si-H-Si(H)Me2](+) + tol	5.51	5.54	4.20
[Me3Si_tol](+) + Me3Si-O-SiMe3 = [(Me3Si)3O](+) + tol	-17.00	-15.98	-12.58
[Me2(H)Si_tol](+) + Me3Si-O-SiMe3 = [(Me3Si)2(Me2SiH)O](+) + tol	-20.96	-20.22	-18.78
[Me3Si_tol](+) + Me3Si-S-SiMe3 = [(Me3Si)3S](+) + tol	-22.99	-22.36	-19.71
[Me2(H)Si_tol](+) + Me3Si-S-SiMe3 = [(Me3Si)2(Me2SiH)S](+) + tol	-23.96	-23.59	-22.89

Carboratereaction – Dication formation **E = Oxygen**

Level of theory: PBE1PBE/def2svp empiricaldispersion=gd3bj

Table S10. Absolute energies in a. u. for all species involved in the dication formation process.

	E	H	G	NImag
**neutral**				
**SiMe4**	-448.668831	-448.511336	-448.554064	0
**Toluene**	-271.055510	-270.919804	-270.957931	0
**Me3Si-O-SiMe3**	-892.842042	-892.597908	-892.658497	0
**cations**				
**[SiMe3](+)**	-408.534127	-408.416502	-408.458823	0
**[(Me3Si)3O](+)**	-1301.466228	-1301.099372	-1301.173178	0
**[Me3Si-O=SiMe2](+) = monocat, u-shaped methyl**	-852.723974	-852.518700	-852.577884	0
**[(Me3Si-O=SiMe2)*SiMe4](+)**	-1301.429163	-1301.063828	-1301.144317	0
iso1	-1301.428760	-1301.063558	-1301.145709	0
**[Me3Si-O=SiMe2(+)]2 = dicat**	-1705.465723	-1705.052741	-1705.139770	0
**anions**				
**HCB11Cl11(-) = cb(-)**	-5371.352714	-5371.240934	-5371.317595	0
**B(C6F5)4(-) = bf(-)**	-2931.140941	-2930.893752	-2931.003317	0

Table S11. Absolute energies in a. u. for all species involved in the dication formation process, relative energies in kcal/mol

toluene adducts	E	H	G	Nimag			
					DE	DH	DG
**[SiMe3*tol](+) para**	**-679.6517328**	**-679.395027**	**-679.45144**	0	**0.00**	**0.00**	**0.00**
[SiMe3*tol](+) ortho	-679.6505945	-679.393594	-679.448183	0	0.71	0.90	2.04
[SiMe3*tol](+) meta	-679.6488138	-679.391922	-679.447971	0	1.83	1.95	2.18
**[(Me3Si)3O*tol](+)**	**-1572.535702**	**-1572.031817**	**-1572.124527**	0			
					DE	DH	DG
**[Me3Si-O=SiMe2*tol](+) para**	**-1123.830445**	**-1123.487054**	**-1123.561523**	0	**0.00**	**0.00**	**0.00**
[Me3Si-O=SiMe2*tol](+) adduct2 metha	-1123.827849	-1123.484308	-1123.55802	0	1.63	1.72	2.20
[Me3Si-O=SiMe2*tol](+) adduct3 ortho	-1123.829479	-1123.485836	-1123.558	0	0.61	0.76	2.21
[Me3Si-O=SiMe2*tol](+) adduct4 para	-1123.830216	-1123.486769	-1123.561311	0	0.14	0.18	0.13
[Me3Si-O=SiMe2*tol](+) adduct5	-1123.830446	-1123.487054	-1123.561538	0	0.00	0.00	-0.01
[Me3Si-O=SiMe2*tol](+) adduct6	-1123.831379	-1123.487917	-1123.560738	0	-0.59	-0.54	0.49
**[dicat*2tol](2+)**	**-2247.625316**	**-2246.936313**	**-2247.062864**	0			
**[toluene_cb](-)**	**-5642.429326**	**-5642.180831**	**-5642.274031**	0			

Table S12. Absolute energies in a. u. for all species involved in the dication formation process, relative energies in kcal/mol

cb - salts	E	H	G	Nimag			
					**DE**	**DH**	**DG**
**[SiMe3]cb**	**-5780.052541**	**-5779.819745**	**-5779.914491**	0	**0.00**	**0.00**	**0.00**
iso1	-5780.050608	-5779.817689	-5779.912212	0	1.21	1.29	1.43
iso2	-5780.040905	-5779.808298	-5779.902935	0	7.30	7.18	7.25
					**DE**	**DH**	**DG**
**[Me3Si-O=SiMe2]cb**	**-6224.231202**	**-6223.911451**	**-6224.021947**	0	**0.00**	**0.00**	**0.00**
iso1	-6224.220025	-6223.900398	-6224.012086	0	7.01	6.94	6.19
iso2	-6224.228314	-6223.908618	-6224.019314	0	1.81	1.78	1.65
iso3	-6224.215816	-6223.896115	-6224.00718	0	9.65	9.62	9.27
iso4	-6224.219401	-6223.899796	-6224.011445	0	7.40	7.31	6.59
iso5	-6224.228314	-6223.908618	-6224.019338	0	1.81	1.78	1.64
iso6	-6224.228314	-6223.908619	-6224.019339	0	1.81	1.78	1.64

Table S13. Absolute energies in a. u. for all species involved in the dication formation process, relative energies in kcal/mol

System: T-O-T/Me3Si/cb vs. monocat/cb/Me4Si	E	H	G	NImag	DE	DH	DG
**iso0_monocat_cb_SiMe4**	**-6672.911304**	**-6672.432024**	**-6672.570739**	0	**0.00**	**0.00**	**0.00**
iso1	-6672.894963	-6672.415767	-6672.552702	0	10.25	10.20	11.32
iso2	-6672.915818	-6672.436343	-6672.570632	0	-2.83	-2.71	0.07
**iso3**	**-6672.911606**	**-6672.432200**	**-6672.568859**	0	-0.19	-0.11	1.18
iso4	-6672.911168	-6672.431778	-6672.570455	0	0.09	0.15	0.18
iso5	-6672.906490	-6672.427236	-6672.566275	0	3.02	3.00	2.80
iso6	-6672.894963	-6672.415767	-6672.552703	0	10.25	10.20	11.32
iso7	-6672.900124	-6672.421100	-6672.560919	0	7.02	6.85	6.16
**iso8**	**-6672.920321**	**-6672.440893**	**-6672.574957**	**0**	**-5.66**	**-5.57**	**-2.65**
**iso9**	**-6672.905388**	**-6672.426158**	**-6672.560738**	**0**	**3.71**	**3.68**	**6.28**
iso10	-6672.916484	-6672.436831	-6672.569720	0	**-3.25**	**-3.02**	**0.64**
iso11	-6672.914201	-6672.434748	-6672.572140	0	**-1.82**	**-1.71**	**-0.88**
iso12	-6672.906134	-6672.426930	-6672.561339	0	**3.24**	**3.20**	**5.90**
iso13	-6672.915033	-6672.435577	-6672.571690	0	**-2.34**	**-2.23**	**-0.60**

Figure S48. Molecular structure of computed isomers of the system O-T/Me3Si/cb vs. monocat/cb/Me4Si

Iso0

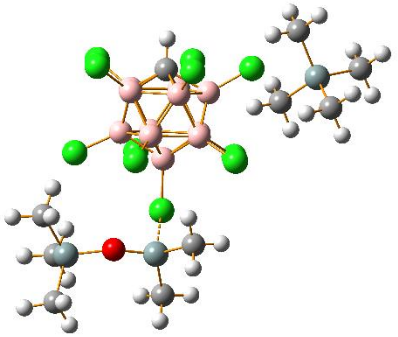

iso1

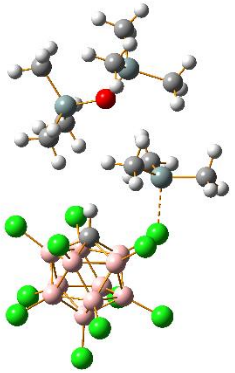

iso2

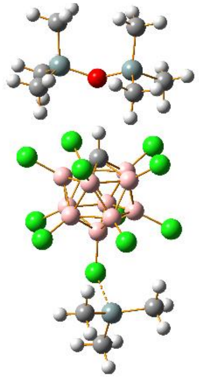

Iso3

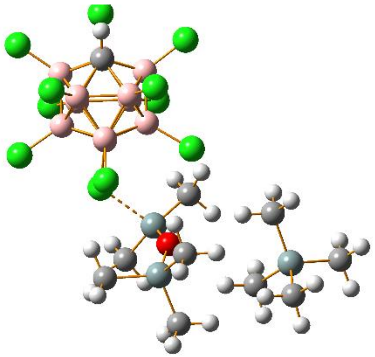

iso4

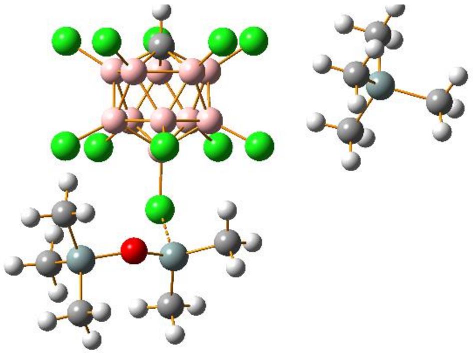

iso5

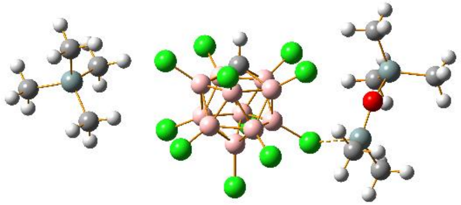

Iso6

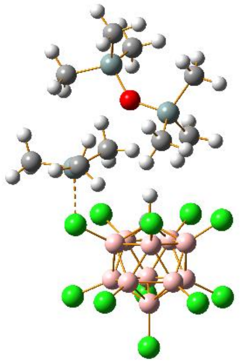

iso7

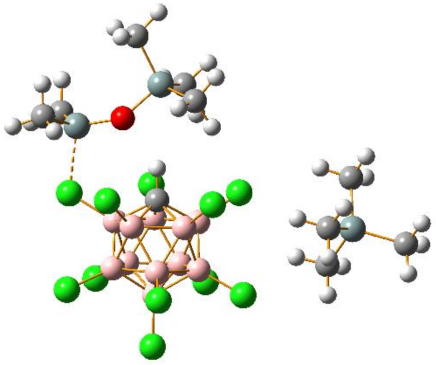

iso8

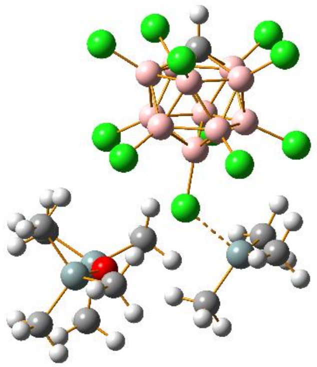

Iso9

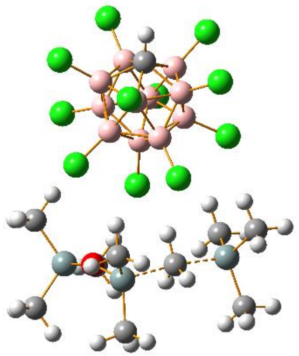

iso10

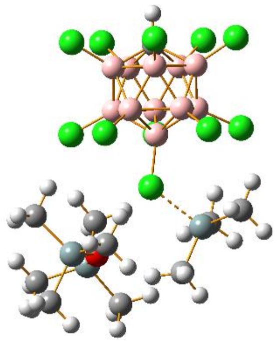

iso11

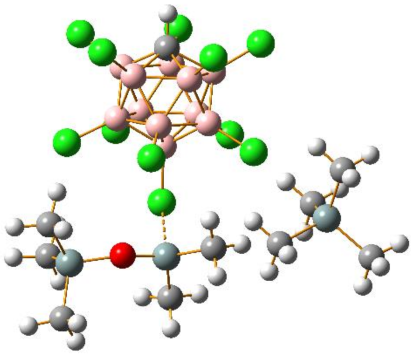

Iso12

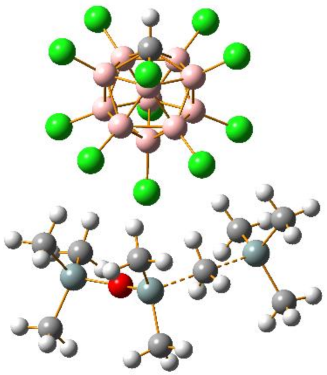

iso13

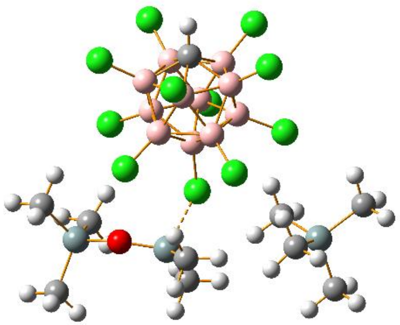

Table S14. Absolute energies in a. u. for all species involved in the dication formation process, relative energies in kcal/mol

	E	H	G	NImag	DE	DH	DG
**[(Me3Si)3O]cb**	**-6672.930513**	**-6672.449458**	**-6672.579315**	0	**0.00**	**0.00**	**0.00**
iso1	-6672.92736	-6672.44641	-6672.577479	0	1.98	1.91	1.15
iso2	-6672.914187	-6672.433113	-6672.563054	0	10.24	10.26	10.20
					DE	DH	DG
**[dicat_cb](+)**	**-7077.028298**	**-7076.500906**	**-7076.640634**	0	**0.00**	**0.00**	**0.00**
iso1	-7077.023601	-7076.495996	-7076.634736	0	2.95	3.08	3.70
iso2	-7077.024449	-7076.496891	-7076.636124	0	2.42	2.52	2.83
					DE	DH	DG
**cb_dicat_cb**	**-12448.512480**	**-12447.870579**	**-12448.068069**	0	**0.00**	**0.00**	**0.00**
iso1	-12448.512517	-12447.870589	-12448.066277	0	-0.02	-0.01	1.12
iso2	-12448.496817	-12447.855386	-12448.053560	0	9.83	9.53	9.10
iso3	-12448.512480	-12447.870579	-12448.068069	0	0.00	0.00	0.00
iso4	-12448.477281	-12447.836675	-12448.032750	0	22.09	21.28	22.16
iso5	-12448.508257	-12447.866608	-12448.063103	0	2.65	2.49	3.12
iso6	-12448.506593	-12447.864848	-12448.061445	0	3.69	3.60	4.16

**bf - salts**				
**[SiMe3]bf**	**-3339.798888**	**-3339.430787**	**-3339.559169**	0
**[Me3Si-O=SiMe2]bf**	**-3783.986255**	**-3783.53085**	**-3783.674441**	0

Table S15. Reaction energies in kcal/mol

[SiMe3](+) addition	DE	DH	DG
[SiMe3](+) + toluene = [SiMe3*tol](+)	-38.97	-36.85	-21.77
[SiMe3](+) + Me3Si-O-SiMe3 = [(Me3Si)3O](+)	-56.51	-53.31	-35.05
[SiMe3](+) + cb(-) = [SiMe3]cb	-103.98	-101.85	-86.64
[SiMe3](+) + bf(-) = [SiMe3]bf	-77.70	-75.64	-60.89

toluene addition	DE	DH	DG
[SiMe3](+) + toluene = [SiMe3*tol](+)	-38.97	-36.85	-21.77
[Me3Si-O=SiMe2](+) + toluene = [Me3Si-O=SiMe2*tol](+)	-31.98	-30.47	-16.13
[(Me3Si)3O](+) + toluene = [(Me3Si)3O*tol](+)	-8.76	-7.93	4.13
[dicat](2+) + 2 toluene = [dicat*2tol](2+)	-30.48	-27.59	-4.54
cb(-) + toluene = toluene*cb(-)	-13.24	-12.61	0.94

cb(-) addition / ion paring	DE	DH	DG
[SiMe3](+) +cb(-) = Me3Si_cb	-103.98	-101.85	-86.64
[Me3Si-O=SiMe2](+) + cb(-) = [Me3Si-O=SiMe2]cb	-96.96	-95.27	-79.36
[Me3Si-O=SiMe2*SiMe4](+) + cb(-) = [Me3Si-O=SiMe2*SiMe4]cb	-81.22	-79.86	-68.29
[(Me3Si)3O](+) + cb(-) = [(Me3Si)3O]cb	-70.01	-68.49	-55.56
dicat + 2cb(-) = [cb_dicat_cb]	-214.19	-210.82	-183.93
1/2[dicat + 2cb(-) = [cb_dicat_cb]	-107.09	-105.41	-91.96

Table S16. Reaction energies in kcal/mol

[Me3Si-O=SiMe2](+) formation (monocat)	DE	DH	DG
**naked**			
**[Me3Si](+) + T-O-T = [Me3Si-O=SiMe2](+) + SiMe4**	-10.44	-9.81	**-9.18**
**[Me3Si](+) + T-O-T = [Me3Si-O=SiMe2*SiMe4](+)**	-33.25	-31.01	**-16.94**
**[(Me3Si)3O](+) = [Me3Si-O=SiMe2](+) + SiMe4**	46.07	43.51	**25.87**
**[(Me3Si)3O](+) = [(Me3Si-O=SiMe2)*SiMe4](+)**	23.26	22.30	**18.11**
**toluene adducts**			
**[Me3Si*tol](+) + T-O-T = [(Me3Si-O=SiMe2)*tol](+) +SiM4**	-3.45	-3.42	**-3.55**
**[(Me3Si)3O](+) + tol = [(Me3Si-O=SiMe2)*tol](+) + SiMe4**	14.10	13.04	**9.74**
[(Me3Si)3O*tol](+) = [(Me3Si-O=SiMe2)*tol](+) + SiMe4	22.86	20.98	5.61
**ion pairs**			
**[SiMe3]cb + Me3Si-O-SiMe3 = [Me3Si-O=SiMe2]cb + SiMe4**	-3.42	-3.22	**-1.90**
**[SiMe3]cb + Me3Si-O-SiMe3 = [Me3Si-O=SiMe2*SiMe4]cb**	-10.49	-9.02	**1.41**
**[(Me3Si)3O]cb = [Me3Si-O=SiMe2]cb + SiMe4**	19.13	16.74	**2.07**
**[(Me3Si)3O]cb = [Me3Si-O=SiMe2*SiMe4]cb**	12.05	10.94	**5.38**

Table S17. Reaction energies in kcal/mol

[(Me3Si)3O](+) formation	DE	DH	DG
**naked**			
[Me3Si](+) + T-O-T = [(Me3Si)3O](+)	-56.51	-53.31	**-35.05**
**toluene adducts**			
[Me3Si*toluene](+) + Me3Si-O-SiMe3 = [(Me3Si)3O](+) + toluene	-17.55	-16.47	**-13.29**
**ion pairs**			
[SiMe3]cb + Me3Si-O-SiMe3 = [(Me3Si)3O]cb	-22.55	-19.96	**-3.97**

Table S18. Reaction energies in kcal/mol

dimerization	DE	DH	DG
**naked**			
2[Me3Si-O=SiMe2](+) = [Me3Si-O=SiMe2(+)]2 (dicat)	-11.15	-9.63	**10.04**
**toluene adducts**			
2[Me3Si-O=SiMe2*toluene](+) = [dicat*toluene](2+)	22.32	23.72	**37.76**
**ion pairs**			
2[Me3Si-O=SiMe2]cb= cb_dicat_cb	-31.42	-29.92	**-15.17**
**starting from [(Me3Si)3O](+)**			
2[(Me3Si)3O](+) = [ring](2+) + 2 SiMe4	80.99	77.39	**61.78**
2[(Me3Si)3O](+) + 2 toluene = [ring*2tol](2+) + 2 SiMe4	50.51	49.80	**57.25**
2[(Me3Si)3O]cb = cb_dicat_cb + 2 SiMe4	6.83	3.55	**-11.02**

Table S19. Reaction energies in kcal/mol

relative energies	DE	DH	DG
naked			
Me3Si-O-SiMe3 + [Me3Si](+)	56.51	53.31	35.05
[Me3Si-O=SiMe2](+) + SiMe4	46.07	43.51	25.87
[(Me3Si-O=SiMe2)*SiMe4](+)	23.26	22.30	18.11
[(Me3Si)3O](+)	0.00	0.00	0.00
1/2[dicat](2+) + SiMe4	40.50	38.70	30.89
toluene stabilized			
Me3Si-O-SiMe3 + [Me3Si*tol](+)	17.55	16.47	13.29
[Me3Si-O=SiMe2*tol](+) + SiMe4	14.10	13.04	9.74
[(Me3Si-O=SiMe2)*SiMe4](+) + toluene	23.26	22.30	18.11
[(Me3Si)3O](+) + toluene	0.00	0.00	0.00
[(Me3Si)3O*toluene](+)	-8.76	-7.93	4.13
1/2[dicat*2tol](2+) + SiMe4	25.26	24.90	28.62
cb salts			
Me3Si-O-SiMe3 + [Me3Si](+) + cb(-)	126.53	121.81	90.61
[Me3Si-O=SiMe2](+) + cb(-) + SiMe4	116.09	112.00	81.43
[(Me3Si-O=SiMe2)*SiMe4](+) + cb(-)	93.27	90.80	73.67
[(Me3Si)3O](+) + cb(-)	70.01	68.49	55.56
Me3Si-O-SiMe3 + [Me3Si]cb	22.55	19.96	3.97
[Me3Si-O-SiMe3 + Me3Si]cb	6.40	5.37	2.73
[Me3Si-O=SiMe2]cb + SiMe4	19.13	16.74	2.07
[(Me3Si-O=SiMe2)....SiMe4]cb	10.24	9.23	4.50
[(Me3Si-O=SiMe2)*SiMe4_adduct]cb	15.30	14.14	11.28
[(Me3Si)3O]cb	0.00	0.00	0.00
1/2 cb_dicat_cb + SiMe4	3.42	1.78	-5.51

continued next page

all together	DE	DH	DG
Me3Si-O-SiMe3 + [Me3Si](+) + toluene + cb(-)	126.53	121.81	90.61
[Me3Si-O=SiMe2](+) + SiMe4 + toluene + cb(-)	116.09	112.00	81.43
[(Me3Si-O=SiMe2)*SiMe4](+) + toluene + cb(-)	93.27	90.80	73.67
[(Me3Si)3O](+) + toluene + cb(-)	70.01	68.49	55.56
1/2 [dicat](2+) + SiMe4 + toluene + cb(-)	110.51	107.19	86.45
Me3Si-O-SiMe3 + [Me3Si*tol](+) + cb(-)	87.56	84.96	68.85
[Me3Si-O=SiMe2*tol](+) + SiMe4 + cb(-)	84.11	81.54	65.30
1/2 [dicat*2tol](2+) + SiMe4 + cb(-)	95.27	93.40	84.18
[(Me3Si-O=SiMe2)*SiMe4](+) + toluene + cb(-)	93.27	90.80	73.67
Me3Si-O-SiMe3 + [Me3Si]cb + tol	22.55	19.96	3.97
[Me3Si-O=SiMe2]cb + SiMe4 + tol	19.13	16.74	2.07
[(Me3Si-O=SiMe2)*SiMe4]cb + tol	12.05	10.94	5.38
[(Me3Si)3O]cb + tol	0.00	0.00	0.00
1/2[cb_dicat_cb] + toluene + SiMe4	3.42	1.78	-5.51
Me3Si-O-SiMe3_cb_Me3Si + toluene	9.22	8.23	5.45

Table S20. Reaction energies*

scrf=(solvent=toluene)

absolute energies, a.u.	E	H	G
**SiMe4**	-448.6691452	-448.511898	-448.554523
**toluene**	-271.0567513	-270.921111	-270.959157
**Me3Si-O-SiMe3**	-892.8430878	-892.599325	-892.659523
**[Me3Si]cb**	-5780.06061	-5779.827967	-5779.923103
**[Me3Si-O=SiMe2]cb**	-6224.239053	-6223.919619	-6224.030676
**[(Me3Si-O=SiMe2)*SiMe4]cb**	-6672.919248	-6672.440483	-6672.579452
**[(Me3Si)3O]cb**	-6672.944061	-6672.464029	-6672.590769
**[cb_dicat_cb]**	-12448.5368	-12447.8945	-12448.09086

relative energies, kacl/mol	DE	DH	DG
**Me3Si-O-SiMe3 + [Me3Si]cb + tol**	25.33	23.05	5.11
**[Me3Si-O=SiMe2]cb + SiMe4 + tol**	22.50	20.40	3.50
**[(Me3Si-O=SiMe2)*SiMe4]cb + tol**	15.57	14.78	7.10
**[(Me3Si)3O]cb + tol**	0.00	0.00	0.00
**1/2[cb_dicat_cb] + toluene + SiMe4**	4.09	3.06	-5.76

*calculation performed in the presence of a solvent by placing the solute in a cavity within the solvent reaction field

Table S21. Absolute energies (a.u.) and reaction energies in (kcal/mol) of transition states involved in the dication formation process

transition states						DE	DH	DG
naked								
T-O-T + Me3Si(+)	-1301.376169	-1301.014410	-1301.117320			56.51	53.31	35.05
[(Me3Si)3O](+)	-1301.466228	-1301.099372	-1301.173178			0.00	0.00	0.00
[Me3Si-O=SiMe2](+) + SiMe4	-1301.392805	-1301.030036	-1301.131948			46.07	43.51	25.87
[(Me3Si-O=SiMe2)*SiMe4](+)	-1301.429163	-1301.063828	-1301.144317			23.26	22.30	18.11
Ea_ts1->forward								
TS1 formation of sime4_adduct from T-O-T and Me3Si(+)	0	scan exergonic						
TS2 formation T3O+ from T-O-T and Me3Si(+)	0	scan exergonic						
				NIMAG				
TS3_formation of sime4_adduct_from_t3o	-1301.413589	-1301.049819	-1301.125969	1	-120.7	33.03	31.09	29.62
reverse reaction						9.77	8.79	11.51
TS4 for Me4Si release from SiMe4 adduct	0	scan endergonic without TS						

Table S22. Absolute energies (a.u.) and reaction energies in (kcal/mol) of transition states involved in the dication formation process

cb- salts	E	H	G					
Me3Si-O-SiMe3 /CB / [Me3Si]	-6672.916484	-6672.436831	-6672.569720					
[Me3Si-O=SiMe2]/CB/SiMe4	-6672.914201	-6672.434748	-6672.572140					
[(Me3Si-O=SiMe2)*SiMe4]cb	-6672.906134	-6672.426930	-6672.561339					
[(Me3Si)3O]cb	-6672.930513	-6672.449458	-6672.579315					
				NIMAG	freq	DE	DH	DG
**TS1 formation of sime4_adduct from T-O-T/CB/Me3Si(+)**	**-6672.883654**	**-6672.40608**	**-6672.538975**	**1**	-114.1	**20.60**	**19.30**	**19.29**
**reverse reaction**						**14.11**	**13.08**	**14.03**
				NIMAG				
**TS2 formation T3O+ from T-O-T /CB/ Me3Si(+) double stab.**	**-6672.900503**	**-6672.421401**	**-6672.549348**	**1**	-42.5	**10.03**	**9.68**	**12.78**
**reverse reaction**						**18.83**	**17.61**	**18.80**
double_stabilized – formal TS2 „isomer“	-6672.896634	-6672.417882	-6672.546538	1	-120.3	12.46	11.89	14.55
double_stabilized – formal TS2 „isomer“	-6672.895738	-6672.416513	-6672.544941	1	-83	13.02	12.75	15.55
mono_stabilized – formal TS2 „isomer“	-6672.882345	-6672.404293	-6672.537535	1	-120.8	21.42	20.42	20.20
				NIMAG				
**TS3_formation of sime4_adduct_from_t3o/CB**	**-6672.884144**	**-6672.406876**	**-6672.539712**	**1**	-78.8	**29.10**	**26.72**	**24.85**
**reverse reaction**						**13.80**	**12.58**	**13.57**
				NIMAG				
**TS4 for Me4Si release from SiMe4 adduct/CB**	**-6672.886623**	**-6672.40879**	**-6672.543005**	**1**	-69.6	**12.24**	**11.38**	**11.50**
**reverse reaction**						**17.31**	**16.29**	**18.28**

E = Sulfur

Table S23. Absolute energies (a.u.)

	E	H	G	Nimag
**neutral**				
**SiMe4**	-448.668831	-448.511336	-448.554064	0
**Toluene**	-271.055510	-270.919804	-270.957931	0
**Me3Si-S-SiMe3**	-1215.661188	-1215.418356	-1215.478186	0
**cations**				
**[SiMe3](+)**	-408.534127	-408.416502	-408.458823	0
**[(Me3Si)3S](+)**	-1624.296283	-1623.931468	-1624.007983	0
**[Me3Si-S=SiMe2](+) = monocat, w-shaped methyl**	-1175.545688	-1175.341957	-1175.40031	0
**[(Me3Si-S=SiMe2)*SiMe4](+)**	-1624.246108	-1623.882128	-1623.961334	0
iso1				0
**[Me3Si-S=SiMe2(+)]2 = dicat**	-2351.093622	-2350.683800	-2350.778072	0
**anions**				
**HCB11Cl11(-) = cb(-)**	-5371.352714	-5371.240934	-5371.317595	0
**B(C6F5)4(-)=bf(-)**	-2931.140941	-2930.893752	-2931.003317	0

Table S24. Absolute energies (a.u.), relative energies in kcal/mol.

toluene adducts	E	H	G				
					DE	DH	DG
**[SiMe3*tol](+) para**	**-679.6517328**	**-679.395027**	**-679.45144**	0	**0.00**	**0.00**	**0.00**
[SiMe3*tol](+) ortho	-679.6505945	-679.393594	-679.448183	0	0.71	0.90	2.04
[SiMe3*tol](+) meta	-679.6488138	-679.391922	-679.447971	0	1.83	1.95	2.18
**[(Me3Si)3S*tol](+)**	**-1895.365404**	**-1894.863670**	**-1894.959306**	0			
**[Me3Si-S=SiMe2*tol](+) para**	**-1446.646186**	**-1446.304173**	**-1446.378017**	0			
**[dicat*2tol](2+)**	**-2893.244857**	**-2892.559035**	**-2892.691012**	0			
**[toluene_cb](-)**	**-5642.429326**	**-5642.180831**	**-5642.274031**	0			

Table S25. Absolute energies (a.u.), relative energies in kcal/mol.

cb - salts	E	H	G	NImag	DE	DH	DG
					DE	DH	DG
**[SiMe3]cb**	**-5780.052541**	**-5779.819745**	**-5779.914491**	0	**0.00**	**0.00**	**0.00**
iso1	-5780.050608	-5779.817689	-5779.912212	0	1.21	1.29	1.43
iso2	-5780.040905	-5779.808298	-5779.902935	0	7.30	7.18	7.25
					DE	DH	DG
**[Me3Si-S=SiMe2]cb**	**-6547.046772**	**-6546.728468**	**-6546.839078**	0	**0.00**	**0.00**	**0.00**
iso1	-6547.031122	-6546.712964	-6546.824881	0	9.82	9.73	8.91
iso2	-6547.043776	-6546.725515	-6546.836499	0	1.88	1.85	1.62
iso3	-6547.031417	-6546.713149	-6546.824507	0	9.64	9.61	9.14
iso4	-6547.03469	-6546.716504	-6546.828096	0	7.58	7.51	6.89
iso5	-6547.043776	-6546.725515	-6546.836499	0	1.88	1.85	1.62
iso6	-6547.043776	-6546.725517	-6546.83651	0	1.88	1.85	1.61

Table S26. Absolute energies (a.u.), relative energies in kcal/mol.

System: T-S-T/Me3Si/cb vs. monocat/cb/Me4Si	E	H	G		DE	DH	DG
**monocat_cb_SiMe4**	**-6995.727683**	**-6995.249851**	**-6995.388244**	0	**0.00**	**0.00**	**0.00**
iso1	-6995.717652	-6995.239799	-6995.376896	0	6.29	6.31	7.12
**iso2**	**-6995.739245**	**-6995.262245**	**-6995.395533**	**0**	**-7.26**	**-7.78**	**-4.57**
iso3	-6995.727741	-6995.249784	-6995.386285	0	-0.04	0.04	1.23
iso4	-6995.72668	-6995.24894	-6995.387905	0	0.63	0.57	0.21
iso5	-6995.723091	-6995.245338	-6995.384104	0	2.88	2.83	2.60
iso6	-6995.717652	-6995.239799	-6995.376909	0	6.29	6.31	7.11
iso7	-6995.711473	-6995.233747	-6995.373420	0	10.17	10.11	9.30
iso8	-6995.737828	-6995.25981	-6995.396288		-6.37	-6.25	-5.05
iso9	-6995.716441	-6995.238891	-6995.373752		7.05	6.88	9.09
					**DE**	**DH**	**DG**
**[(Me3Si)3S]cb**	**-6995.758863**	**-6995.280193**	**-6995.413355**	0	**0.00**	**0.00**	**0.00**
iso1	-6995.755729	-6995.277014	-6995.41156	0	1.97	1.99	1.13
iso2	-6995.753286	-6995.274657	-6995.407611	0	3.50	3.47	3.60
					**DE**	**DH**	**DG**
**[dicat_cb](+)**	**-7722.65197**	**-7722.127798**	**-7722.273389**	0	**0.00**	**0.00**	**0.00**
					**DE**	**DH**	**DG**
**cb_dicat_cb iso1**	**-13094.126482**	**-13093.488128**	**-13093.691408**	0	**0.00**	**0.00**	**0.00**
Iso2 – see oxygen	-13094.125889	-13093.488559	-13093.688325	0	0.37	-0.27	1.93
iso2	-13094.111165	-13093.473143	-13093.672476	0	9.61	9.40	11.88
iso3	-13094.127773	-13093.489244	-13093.689945	0	-0.81	-0.70	0.92
iso4	-13094.109695	-13093.473765	-13093.671321	0	10.53	9.01	12.60
iso5	-13094.124646	-13093.486284	-13093.687619	0	1.15	1.16	2.38

Table S27. Reaction energies in kcal/mol.

[SiMe3](+) addition	DE	DH	DG
[SiMe3](+) + toluene = [SiMe3*tol](+)	-38.97	-36.85	-21.77
[SiMe3](+) + Me3Si-S-SiMe3 = [(Me3Si)3S](+)	-63.36	-60.62	-44.54
[SiMe3](+) + cb(-) = [SiMe3]cb	-103.98	-101.85	-86.64
[SiMe3](+) + bf(-) = [SiMe3]bf	-77.70	-75.64	-60.89
**toluene addition**	**DE**	**DH**	**DG**
[SiMe3](+) + toluene = [SiMe3*tol](+)	-38.97	-36.85	-21.77
[Me3Si-S=SiMe2](+) + toluene = [Me3Si-S=SiMe2*tol](+)	-28.23	-26.61	-12.41
[(Me3Si)3S](+) + toluene = [(Me3Si)3S*tol](+)	-8.54	-7.78	4.15
[dicat](2+) + 2 toluene = [dicat*2tol](2+)	-25.24	-22.36	1.83
cb(-) + toluene = toluene*cb(-)	-13.24	-12.61	0.94
**cb(-) addition / ion paring**	**DE**	**DH**	**DG**
[SiMe3](+) +cb(-) = Me3Si_cb	-103.98	-101.85	-86.64
[Me3Si-S=SiMe2](+) + cb(-) = [Me3Si-S=SiMe2]cb	-93.10	-91.35	-76.04
[Me3Si-S=SiMe2*SiMe4](+) + cb(-) = [Me3Si-S=SiMe2*SiMe4]cb	-80.86	-79.56	-68.60
[(Me3Si)3S](+) + cb(-) = [(Me3Si)3S]cb	-68.94	-67.64	-55.08
dicat + 2cb(-) = cb_dicat_cb	-205.47	-202.35	-174.54
1/2[dicat + 2cb(-) = cb_dicat_cb]	-102.73	-101.17	-87.27

Table S28. Reaction energies in kcal/mol.

[Me3Si-S=SiMe2](+) formation (monocat)	DE	DH	DG
naked			
[Me3Si](+) + T-O-T = [Me3Si-O=SiMe2](+) + SiMe4	-12.05	-11.57	-10.90
[Me3Si](+) + T-O-T = [Me3Si-O=SiMe2*SiMe4](+)	-31.87	-29.66	-15.26
[(Me3Si)3S](+) = [Me3Si-S=SiMe2](+) + SiMe4	51.31	49.06	33.64
[(Me3Si)3S](+) = [(Me3Si-S=SiMe2)*SiMe4](+)	31.49	30.96	29.27
toluene adducts			
[Me3Si*tol](+) + T-S-T = [(Me3Si-S=SiMe2)*tol](+) +SiM4	-1.32	-1.33	-1.54
[(Me3Si)3S](+) + tol = [(Me3Si-S=SiMe2)*tol](+) + SiMe4	23.08	22.44	21.23
[(Me3Si)3S*tol](+) = [(Me3Si-S=SiMe2)*tol](+) + SiMe4	31.62	30.22	17.08
ion pairs			
[SiMe3]cb + Me3Si-S-SiMe3 = [Me3Si-S=SiMe2]cb + SiMe4	-1.18	-1.07	-0.29
[SiMe3]cb + Me3Si-S-SiMe3 = [Me3Si-S=SiMe2*SiMe4]cb	-8.76	-7.37	2.78
[(Me3Si)3S]cb = [Me3Si-S=SiMe2]cb + SiMe4	27.15	25.34	12.68
[(Me3Si)3S]cb = [Me3Si-S=SiMe2*SiMe4]cb	19.57	19.04	15.76
**[(Me3Si)3O](+) formation**	**DE**	**DH**	**DG**
naked			
[Me3Si](+) + T-S-T = [(Me3Si)3S](+)	-63.36	-60.62	-44.54
toluene adducts			
[Me3Si*toluene](+) + Me3Si-S-SiMe3 = [(Me3Si)3S](+) + toluene	-24.39	-23.78	-22.77
ion pairs			
[SiMe3]cb + Me3Si-S-SiMe3 = [(Me3Si)3S]cb	-28.32	-26.41	-12.98

Table S29. Reaction energies in kcal/mol.

dimerization	DE	DH	DG
**naked**			
**2[Me3Si-S=SiMe2](+) = [Me3Si-S=SiMe2(+)]2 (dicat)**	-1.41	0.07	**14.15**
**toluene adducts**			
**2[Me3Si-S=SiMe2*toluene](+) = [dicat*toluene](2+)**	29.82	30.94	**40.80**
**ion pairs**			
**2[Me3Si-S=SiMe2]cb= cb_dicat_cb**	-20.67	-19.57	**-8.32**
**starting from [(Me3Si)3S](+)**			
**2[(Me3Si)3S](+) = [ring](2+) + 2 SiMe4**	101.21	98.18	**81.43**
**2[(Me3Si)3S](+) + 2 toluene = [ring*2tol](2+) + 2 SiMe4**	75.97	75.83	**83.26**
**2[(Me3Si)3S]cb = cb_dicat_cb + 2 SiMe4**	33.62	31.12	**17.05**

Table S30. Relative energies in kcal/mol.

relative energies	DE	DH	DG
naked			
Me3Si-S-SiMe3 + [Me3Si](+)	63.36	60.62	44.54
[Me3Si-S=SiMe2](+) + SiMe4	51.31	49.06	33.64
[(Me3Si-S=SiMe2)*SiMe4](+)	31.49	30.96	29.27
[(Me3Si)3S](+)	0.00	0.00	0.00
1/2[dicat](2+) + SiMe4	50.60	49.09	40.71
toluene stabilized			
Me3Si-S-SiMe3 + [Me3Si*tol](+)	24.39	23.78	22.77
[Me3Si-S=SiMe2*tol](+) + SiMe4	23.08	22.44	21.23
[(Me3Si-S=SiMe2)*SiMe4](+) + toluene	31.49	30.96	29.27
[(Me3Si)3S](+) + toluene	0.00	0.00	0.00
[(Me3Si)3S*toluene](+)	-8.54	-7.78	4.15
1/2[dicat*2tol](2+) + SiMe4	37.99	37.91	41.63
cb salts			
Me3Si-S-SiMe3 + [Me3Si](+) + cb(-)	132.30	128.26	99.62
[Me3Si-S=SiMe2](+) + cb(-) + SiMe4	120.25	116.70	88.72
[(Me3Si-S=SiMe2)*SiMe4](+) + cb(-)	100.43	98.60	84.35
[(Me3Si)3S](+) + cb(-)	68.94	67.64	55.08
Me3Si-S-SiMe3 + [Me3Si]cb	28.32	26.41	12.98
[Me3Si-S-SiMe3 + Me3Si]cb	13.20	12.79	10.71
[Me3Si-S=SiMe2]cb + SiMe4	27.15	25.34	12.68
[(Me3Si-S=SiMe2)*SiMe4]cb	19.57	19.04	15.76
[(Me3Si)3S]cb	0.00	0.00	0.00
1/2 cb_dicat_cb + SiMe4	16.81	15.56	8.53

continued next page

all together			
Me3Si-S-SiMe3 + [Me3Si](+) + toluene + cb(-)	132.30	128.26	99.62
[Me3Si-S=SiMe2](+) + SiMe4 + toluene + cb(-)	120.25	116.70	88.72
[(Me3Si-S=SiMe2)*SiMe4](+) + toluene + cb(-)	100.43	98.60	84.35
[(Me3Si)3S](+) + toluene + cb(-)	68.94	67.64	55.08
1/2 [dicat](2+) + SiMe4 + toluene + cb(-)	119.54	116.73	95.80
Me3Si-S-SiMe3 + [Me3Si*tol](+) + cb(-)	93.33	91.42	77.85
[Me3Si-S=SiMe2*tol](+) + SiMe4 + cb(-)	92.02	90.08	76.31
1/2 [dicat*2tol](2+) + SiMe4 + cb(-)	106.93	105.55	96.71
[(Me3Si-S=SiMe2)*SiMe4](+) + toluene + cb(-)	100.43	98.60	84.35
Me3Si-S-SiMe3 + [Me3Si]cb + tol	28.32	26.41	12.98
[Me3Si-S=SiMe2]cb + SiMe4 + tol	27.15	25.34	12.68
[(Me3Si-S=SiMe2)*SiMe4]cb + tol	19.57	19.04	15.76
[(Me3Si)3S]cb + tol	0.00	0.00	0.00
1/2[cb_dicat_cb] + toluene + SiMe4	16.81	15.56	8.53
Me3Si-O-SiMe3_cb_Me3Si + toluene	12.31	11.26	11.18

Ligand Scrambling

Table S31. NBO charges in e.

**NBO**							
charge in e	Si1	Si2	TMS1	TMS2	Me2SiH	H_bridge	Me_bridge
Me3Si+	2.017	-	**1.000**				
[Me3Si-H-SiMe3](+)	1.718	1.718	0.652	0.652	-	**-0.305**	-
[Me3Si-Me-Si(H)Me2](+)	1.838	1.511	0.724	-	**0.643**	-	**-0.367**

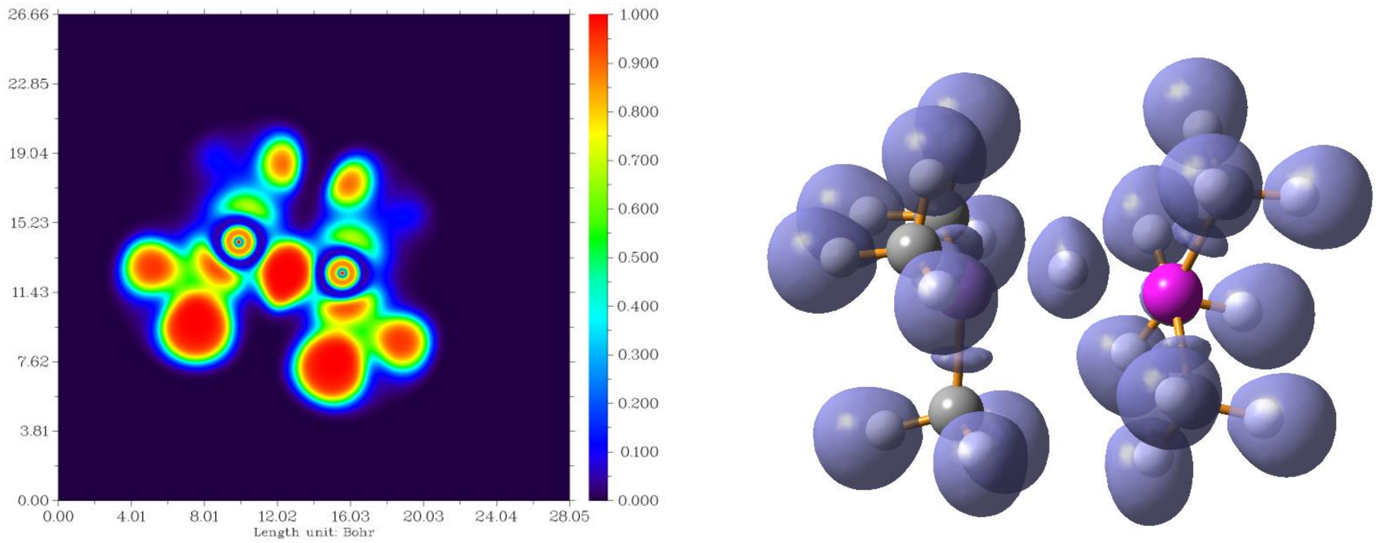

Figure S49. ELF representations of [T-H-T]+, left: 2-d T-H-T plane, right: 3D-ELF at 0.9.

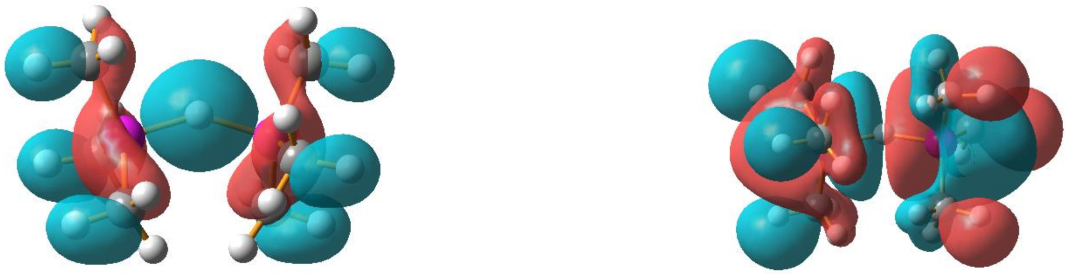

Figure S50. HOMO (left) and LUMO (right) of [T-H-T]+.

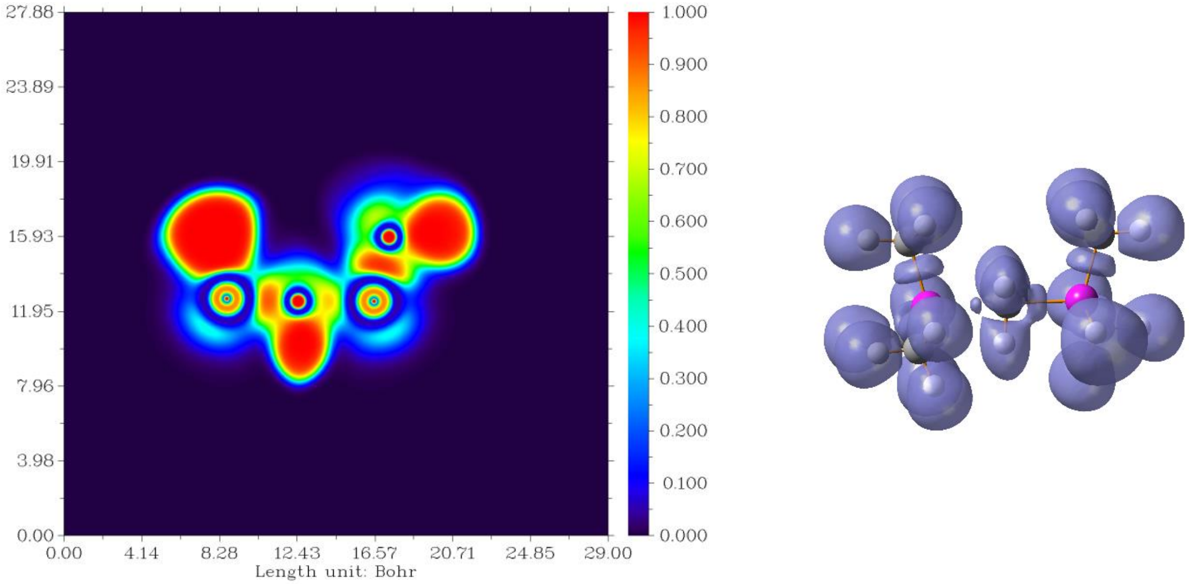

Figure S51. ELF representations of [T-Me-Si(H)Me2]+, left: 2-d T-H-T plane, right: 3D-ELF at 0.9.

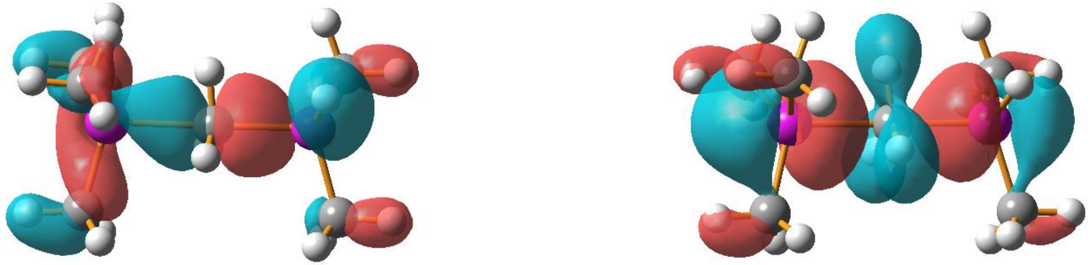

Figure S52. HOMO-2 (left) and LUMO (right) of [T-Me-Si(H)Me2]+.

Table S32. NBO data of oxygen species, charges in e.

	Si1	Si2	TMS1	TMS2 or DMS	H	O	X = O,S LP (X) X p character %	occ	QCT
Me3Si-O-SiMe3	1.962	1.962	0.633	0.633		-1.266	99.80	1.908	-
[(Me3Si)3O](+)	1.940	1.940	0.748	0.748	-	-1.244	98.30	1.967	**0.252**
[(Me3Si)2(Me2SiH)O](+)	1.944	1.670	0.752	0.738	**-0.179**	-1.242	98.88	1.938	
[Me3Si-O=SiMe2](+) u-shaped methyl	1.939	2.185	0.988	**1.241**	-	-1.229	83.79	1.937	
[Me3Si-O=SiMe2]*tol(+) para	1.948	2.083	0.699	**1.283**		-1.274	79.84/97.88	1.923/1.903	**0.291**
[Me3Si-O=SiMe2(+)]2	1.936	2.203	0.806	1.468		-1.274	99.38	1.933	

Table S33. NBO data of sulfur species, charges in e.

	Si1	Si2	TMS1	TMS2 or DMS	H	S	X = O,S LP (X) X p character	occ
Me3Si-S-SiMe3	1.566	1.566	0.324	0.324	-	-0.648	99.65	1.879
[(Me3Si)3S](+)	1.635	1.635	0.882	0.882	-	-0.518	63.88	1.937
[(Me3Si)2(Me2SiHS](+)	1.640	1.343	0.514	0.484	**-0.147**	-0.512	62.11	1.939
[Me3Si-S=SiMe2](+) u-shaped methyl	1.624	1.651	0.506	0.986	-	-0.492	39.52	1.950
[Me3Si-S=SiMe2(+)]2	1.687	1.542	0.621	0.879	-	-0.500	58.97	1.932

Table S34. NBO/NRT data of oxygen/sulfur species, charges in e.

	NBO									LP	NRT Si-E bond		STERIC tot
	Si1	TMS1	H	E	QCT_TMS*	LP p%	occ	loc E-Si	loc E-H	**DA energy**	cov. BO	ionic BO	exchange
H3O+	-	-	0.590	-0.769	-	84.100	1.999	-	79.570	4.4	0.409	0.591	-14.5
TH2O+	1.935	0.802	0.559	-0.920	0.198	96.540	1.983	91.04	78.110	8.2	0.170	0.785	171.8
T2HO+	1.945	0.763	0.553	-1.079	0.237	99.990	1.955	89.84	77.870	16.3	0.193	0.767	384.9
T3O+	1.931	0.740	-	-1.219	0.260	99.370	1.927	89.21	-	**20.7**	0.202	0.749	642.1
T2_Me2SiH_O+	1.940	0.570	1.669	-1.222	0.430	99.630	1.926	89.47	88.710	21.5			555.9
H3S+	-	-	0.275	0.173	-	46.470	1.999	-	63.840	0.0	0.723	0.277	-46.5
TH2S+	1.685	0.612	0.248	-0.108	0.389	50.940	1.989	81.16	62.510	4.2	0.354	0.591	137.6
T2HS+	1.666	0.551	0.242	-0.344	0.449	59.050	1.966	78.78	62.190	10.2	0.402	0.551	334.9
T3S+	1.661	0.520	-	-0.559	**0.480**	68.530	1.932	77.67	-	18.2	0.421	0.531	552.8
T3S+ (planar)	1.687	0.543	-	-0.630	0.457	100.000	1.905	79.1	-	**27.1**	0.421	0.531	512.1

*per tms group

Carborate reaction – Dication formation

Table S35. NBO/NRT data of oxygen/sulfur species, charges in e.

dications	Q (E)	Q(TMS term)	QCT	LP p %	deloc LP in Si_ring-C	deloc LP in Si_term-C	total LP deloc
O	-1.249	0.796	0.204	99.7	35.00	10.30	65.00
S	-0.541	0.638	0.362	64.0	17.80	7.16	47.57

Table S36. NBO/NRT data of oxygen/sulfur species, charges in e.

ion pairs	q(cat)	q(CB)	charge transfer	q(E)
T_CB	0.599	-0.599	0.401	-
T3O_CB	0.885	-0.885	0.115	-1.212
T3S_CB	0.881	-0.881	0.119	-0.585
O_monocat_CB	0.621	-0.621	0.379	-1.243
S_monocat_CB	0.607	-0.607	0.393	-0.574
O-dicat_CB	0.887	-0.887	0.114	-1.248
S-dicat_CB	0.880	-0.880	0.120	-0.530

Table S37. NBO/STERIC/DELETION data of $[T_3E]^+$ in C_3 symmetry and fixed Si-E-Si angles (α).

	E(full)	E(L)	E(NL) = E(full)-E(L)			Delta	values				
**oxygen**	$E^{tot,scf}$			E(NL)	**steric**	DE(full)	DE(L)	DE(NL)	DE,steric	LP, deloc	delta, LP
α in °	a.u.	a.u.	a.u.	kcal/mol	**kcal/mol**	kcal/mol	kcal/mol	kcal/mol	kcal/mol	kcal/mol	kcal/mol
120	-1301.465242	-1300.621101	-0.844141	-529.71	641.20	0.00	0.00	0.00	0.00	30.263	0.00
119	-1301.466168	-1300.619392	-0.846776	-531.36	641.97	-0.58	1.07	-1.65	0.77	30.158	-0.11
109	-1301.447564	-1300.534663	-0.912901	-572.85	668.57	11.09	54.24	-43.15	27.37	29.983	-0.28
90	-1301.351577	-1300.197612	-1.153964	-724.12	730.63	71.33	265.74	-194.42	89.43	35.678	5.42
	E(full)	E(L)	E(NL) = E(full)-E(L)			Delta	values				
**sulfur**	$E^{tot,scf}$			E(NL)	**steric**	DE(full)	DE(L)	DE(NL)	DE,steric	LP, deloc	delta, LP
α in °	a.u.	a.u.	a.u.	kcal/mol	**kcal/mol**	kcal/mol	kcal/mol	kcal/mol	kcal/mol	kcal/mol	kcal/mol
120	-1624.287698	-1623.518538	-0.769161	-482.66	512.07	0.00	0.00	0.00	0.00	30.504	0.00
119	-1624.288863	-1623.520673	-0.768190	-482.05	514.50	-0.73	-1.34	0.61	2.43	30.122	-0.38
109	-1624.296192	-1623.522730	-0.773462	-485.35	551.50	-5.33	-2.63	-2.70	37.00	23.959	-6.55
90	-1624.262873	-1623.398991	-0.863882	-542.09	644.37	15.58	75.02	-59.44	92.87	22.407	-8.10

Table S38. Shubin Liu analysis (SLA) of $T_3O(+)$ in C_3 symmetry and fixed Si-O-Si angles.

		min Etot		SLA					SLA delta			delta	delta
		**-1301.465210**		E_steric	E_electrostatic	E_quantum	Ex	Epauli	E_steric	E_electrostat	E_quant	Ex	Epauli
		Etot	DErel						DE				
Si-O-Si	X-O-Si	a.u.	kcal/mol	a.u.	a.u.	a.u.	a.u.	a.u.	kcal/mol	kcal/mol	kcal/mol	kcal/mol	
120.00	90	-1301.465209	0.00	887.370998	-2465.742614	276.906375	-124.763978	406.119896	0	0	0	0	0
119.97	91	-1301.465608	-0.25										
119.88	92	-1301.465911	-0.44										
119.73	93	-1301.466113	-0.57										
119.52	94	-1301.466209	-0.63	887.358074	-2465.743633	276.919391	-124.763900	406.133385	-8.11	-0.64	8.17	0.05	8.46
119.25	95	-1301.466183	-0.61										
118.92	96	-1301.466052	-0.53										
118.54	97	-1301.465777	-0.36										
118.10	98	-1301.465347	-0.09										
117.60	99	-1301.464752	0.29										
117.05	100	-1301.463982	0.77	887.339625	-2465.742989	276.939383	-124.760307	406.151633	-19.69	-0.24	20.71	2.30	19.92
116.45	101	-1301.463027	1.37										
115.80	102	-1301.461875	2.09										
115.09	103	-1301.460518	2.94	887.334922	-2465.741156	276.945652	-124.756316	406.155235	-22.64	0.91	24.65	4.81	22.18
114.34	104	-1301.458942	3.93										
113.55	105	-1301.457136	5.07										
112.71	106	-1301.455092	6.35	887.333404	-2465.738333	276.949802	-124.750892	406.155570	-23.59	2.69	27.25	8.21	22.39
111.83	107	-1301.4528	7.79										
110.90	108	-1301.450377	9.31										
109.94	109	-1301.447564	11.07	887.333134	-2465.732887	276.952189	-124.743922	406.152968	-23.76	6.10	28.75	12.59	20.75
108.94	110	-1301.444445	13.03										
107.90	111	-1301.441005	15.19	887.336400	-2465.727913	276.950507	-124.738187	406.146975	-21.71	9.23	27.69	16.18	16.99
106.83	112	-1301.437226	17.56										
105.72	113	-1301.433094	20.15										

104.59	114	-1301.428598	22.97	887.344690	-2465.717267	276.943980	-124.727649	406.132214	-16.51	15.91	23.60	22.80	7.73
103.42	115	-1301.423728	26.03										
102.22	116	-1301.418478	29.32										
101.00	117	-1301.412836	32.86	887.357462	-2465.701940	276.931641	-124.714577	406.109318	-8.49	25.52	15.85	31.00	-6.64
99.75	118	-1301.406792	36.66										
98.48	119	-1301.400325	40.72										
97.18	120	-1301.393414	45.05	887.375281	-2465.682834	276.914139	-124.699082	406.079039	2.69	37.51	4.87	40.72	-25.64
95.86	121	-1301.386039	49.68										
94.52	122	-1301.37818	54.61	887.391178	-2465.668257	276.898898	-124.687185	406.053744	12.66	46.66	-4.69	48.19	-41.51
93.16	123	-1301.369824	59.86										
91.77	124	-1301.36096	65.42										
90.37	125	-1301.351577	71.31	887.422109	-2465.644875	276.871190	-124.667085	406.008669	32.07	61.33	-22.08	60.80	-69.80

Table S39. Shubin Liu analysis (SLA) of **$T_3S(+)$** in C_3 symmetry and fixed Si-S-Si angles.

		min Etot		SLA					SLA delta			delta	delta
		-1624.287652		E_steric	E_electrostatic	E_quantum	Ex	Epauli	E_steric	E_electrostat	E_quant	Ex	Epauli
		Etot	DErel						DE				
Si-S-Si	X-S-Si	a.u.	kcal/mol	a.u.	a.u.	a.u.	a.u.	a.u.	kcal/mol	kcal/mol	kcal/mol	kcal/mol	
120.00	90	-1624.287652	0.00	1072.240185	-3094.238265	397.710383	-141.418908	543.922311	0.00	0.00	0.00	0.00	0.00
119.97	91	-1624.287859	-0.13										
119.88	92	-1624.288132	-0.30										
119.73	93	-1624.288470	-0.51										
119.52	94	-1624.288863	-0.76	1072.237102	-3094.243180	397.717215	-141.418791	543.929238	-1.93	-3.08	4.29	0.07	4.35
119.25	95	-1624.289308	-1.04										
118.92	96	-1624.289801	-1.35										
118.54	97	-1624.290341	-1.69										
118.10	98	-1624.290928	-2.06										
117.60	99	-1624.291549	-2.45										
117.05	100	-1624.292189	-2.85	1072.219866	-3094.251890	397.739907	-141.418326	543.952197	-12.75	-8.55	18.53	0.37	18.75
116.45	101	-1624.292835	-3.25										
115.80	102	-1624.293466	-3.65										
115.09	103	-1624.294063	-4.02	1072.204629	-3094.254184	397.755576	-141.417952	543.968156	-22.31	-9.99	28.36	0.60	28.77
114.34	104	-1624.294611	-4.37										
113.55	105	-1624.295098	-4.67										
112.71	106	-1624.295515	-4.93	1072.183452	-3094.253169	397.774200	-141.417042	543.986789	-35.60	-9.35	40.05	1.17	40.46
111.83	107	-1624.295846	-5.14										
110.90	108	-1624.296078	-5.29										
109.94	109	-1624.296192	-5.36	1072.158372	-3094.245964	397.791401	-141.415223	544.003324	-51.34	-4.83	50.84	2.31	50.84
108.94	110	-1624.296164	-5.34										
107.90	111	-1624.295964	-5.22	1072.141977	-3094.238153	397.800212	-141.413700	544.011508	-61.63	0.07	56.37	3.27	55.97
106.83	112	-1624.295565	-4.97										
105.72	113	-1624.294941	-4.57										

104.59	114	-1624.294070	-4.03	1072.116068	-3094.222226	397.812089	-141.410511	544.021796	-77.88	10.06	63.82	5.27	62.43
103.42	115	-1624.292927	-3.31										
102.22	116	-1624.291497	-2.41										
101.00	117	-1624.289761	-1.32	1072.088399	-3094.201054	397.822894	-141.406037	544.030115	-95.25	23.35	70.60	8.08	67.65
99.75	118	-1624.287705	-0.03										
98.48	119	-1624.285311	1.47										
97.18	120	-1624.282560	3.19	1072.061683	-3094.173362	397.829118			-112.01	40.73	74.51		
95.86	121	-1624.279436	5.16										
94.52	122	-1624.275920	7.36	1072.045268	-3094.148895	397.827505	-141.394273	544.026958	-122.31	56.08	73.50	15.46	65.67
93.16	123	-1624.271996	9.82										
91.77	124	-1624.267651	12.55										
90.37	125	-1624.262873	15.55	1072.020194	-3094.109570	397.826503	-141.384460	544.018956	-138.05	80.76	72.87	21.62	60.65

Figure S53. Energy potentials as function of the Si-E-Si angle in C3 symmetry, rel. energies in kcal/mol

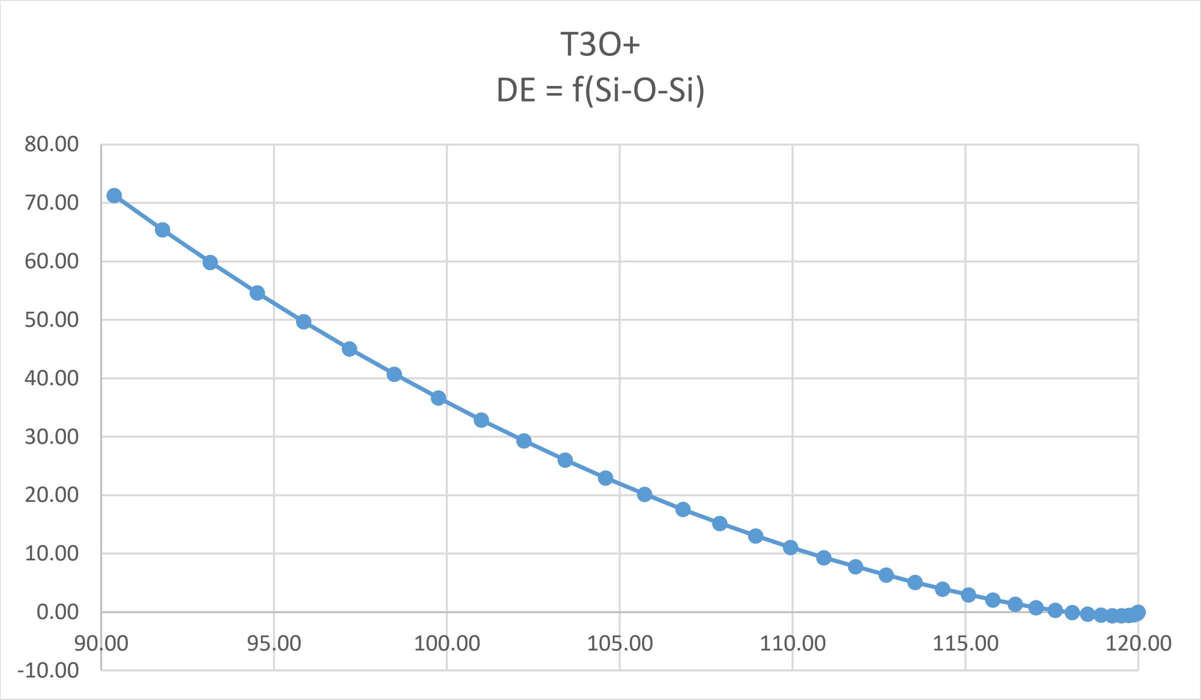

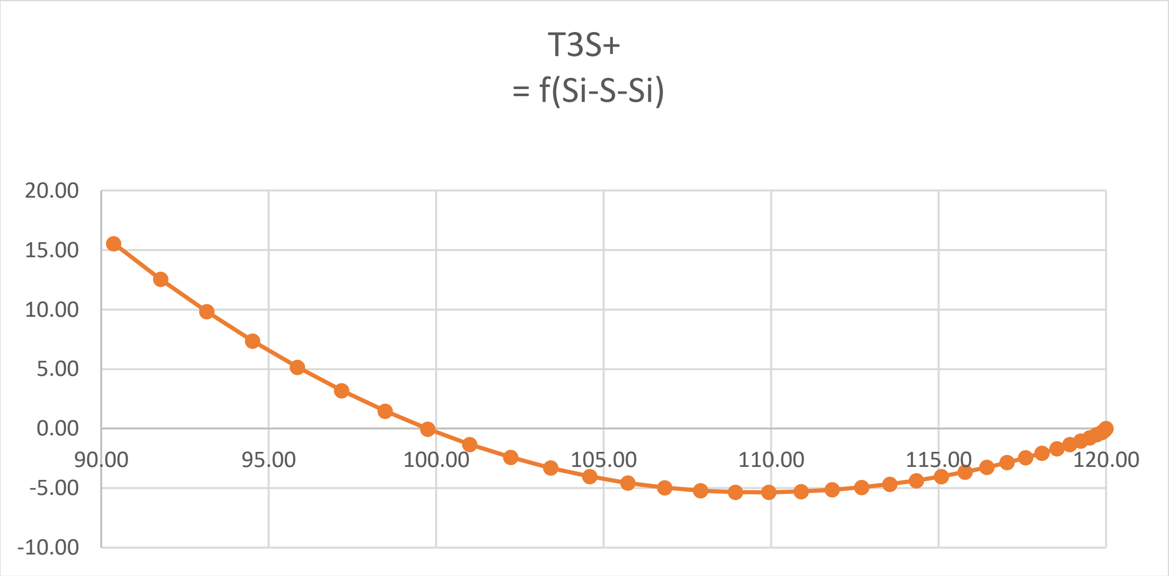

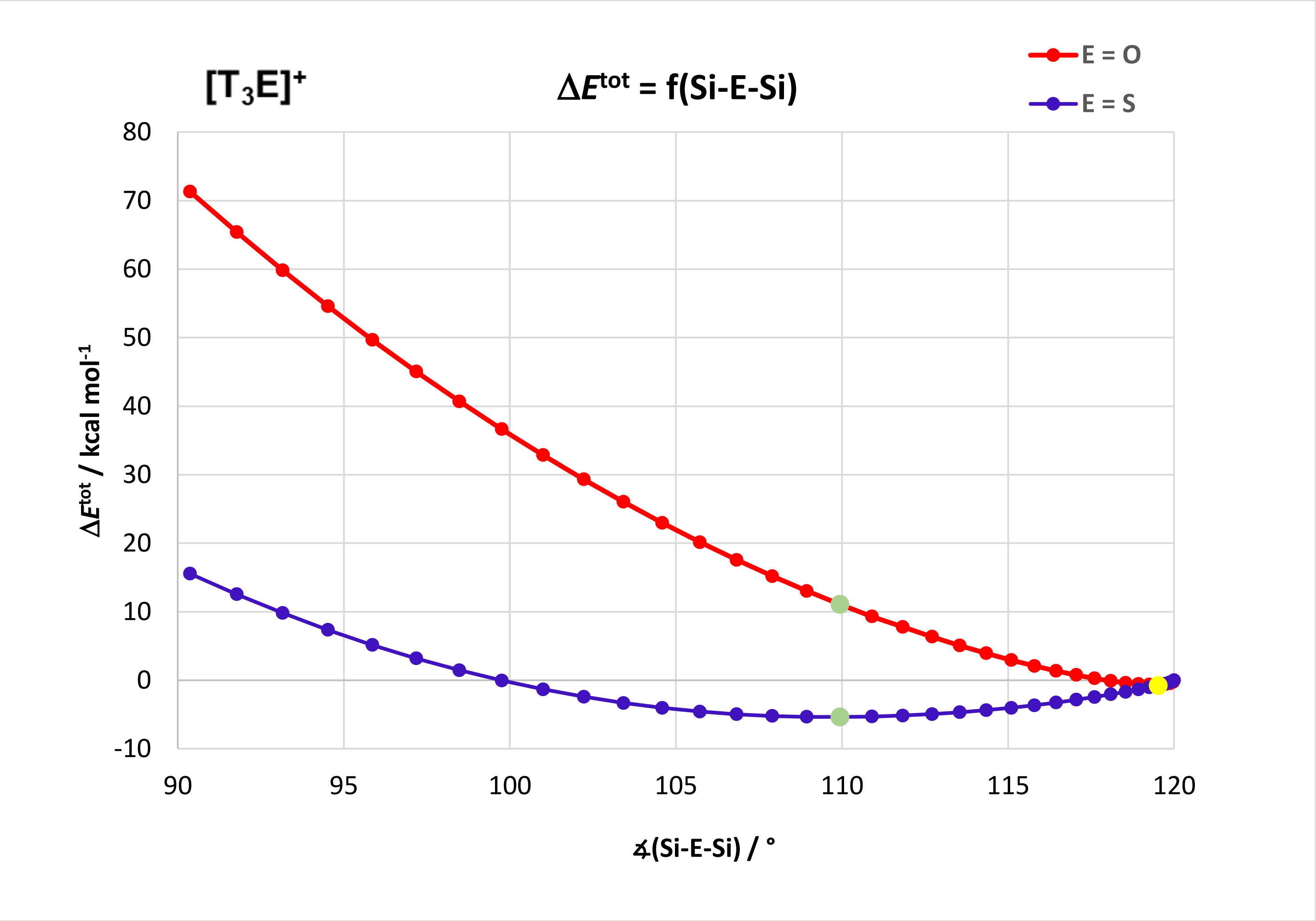

[T3E]+
ΔEtot = f(Si-E-Si)
E = O
E = S
80
70
60
50
40
30
20
10
0
-10
ΔEtot / kcal mol-1
90
95
100
105
110
115
120
∡(Si-E-Si) / °

Selected structural data

Ligand Scrambling

Table S40. Selected structural data (bond lengths in Å, angles in °) – see Figure S53

			averaged			
	**Si-H**	**Si-H-Si**	**Si-C-Si**	**sum_angle_Si**		
**[Me3Si-H-SiMe3](+), (7)**	1.636	147.4	116.050	348.200		
	**Si(H)-C_bridge**	**Si-C_bridge**		**sum1_SiMe3**	**sum2_Me**	**sum_SiHMe2**
**[Me3Si-Me-Si(H)Me2](+), (8)**	2.013	2.140	177.857	345.000	356.800	341.400
	**Si-H**	**Si-H-Si**		**sum_angle_Si**		
**[Me3Si-H-Si(H)Me2]**	1.61809 (bridge)	135.48188	116.5	349.4 (T)		
	1.66499 (bridge)			348.1 (SiMe2H)		
	1.49038 (term)					
	**Si-H**	**Si-Me bridge**		**Si-H-Si**	**Si-C-Si**	**sum_angle_Si**
**TS1**	1.51045	1.92016	117.5	102.96158	84.82825	352.4 (T+)
	2.46881	2.70138				

Figure S54. Computed structures that play an essential role in the silylium ion catalysed scrambling process.

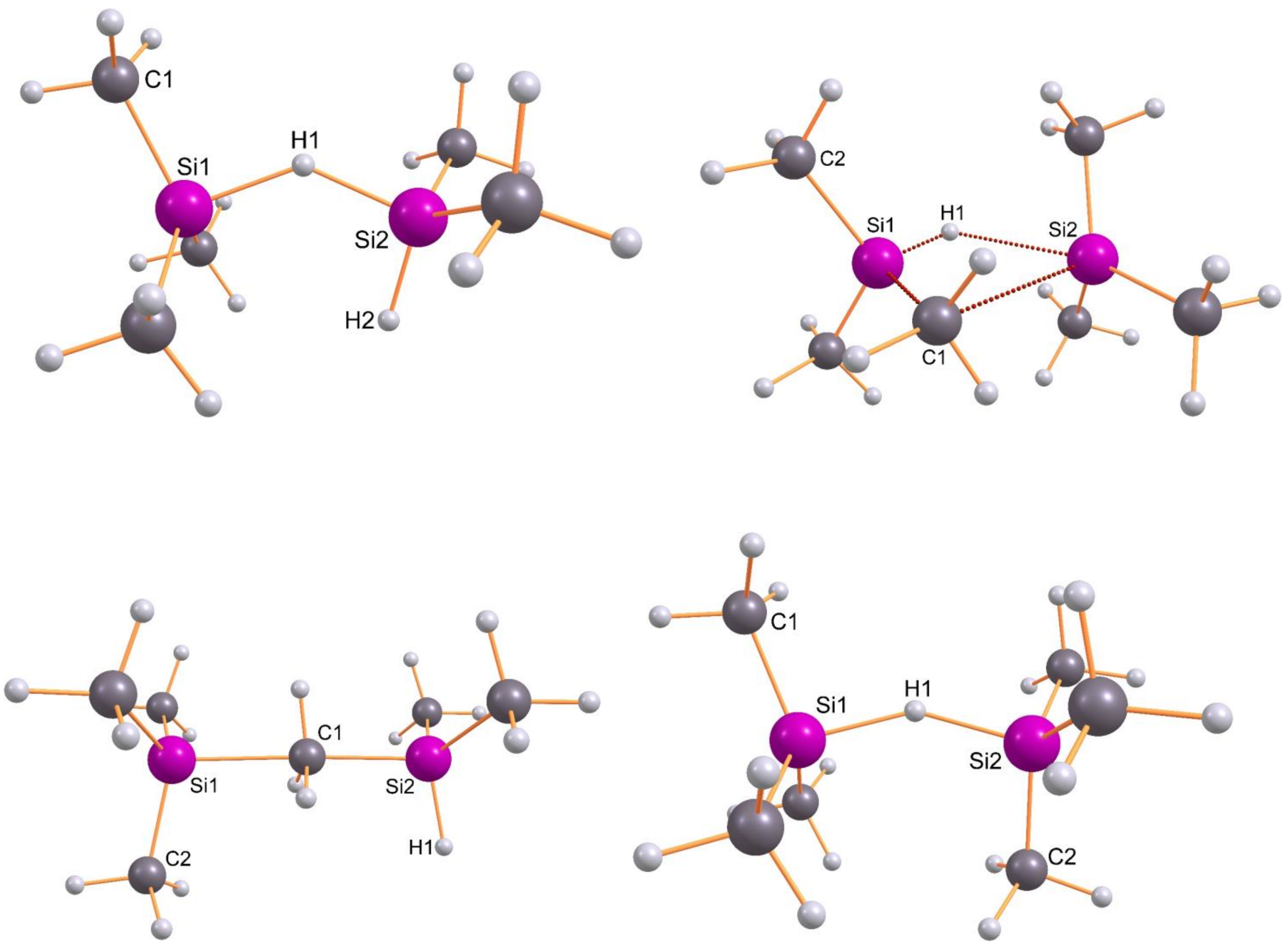

Table S41. Selected structural data (bond lengths in Å, angles in °)

	E-H	E-Si	H-E-H	H-E-T	T-E-T	angle sum E
**H3O+**	0.982	-	112.6	-	-	337.7
**TH2O+**	0.971	1.908	109.8	122.8	-	355.4
**T2HO+**	0.970	1.831	-	112.9	134.3	360.0
**T3O+**	-	1.823	-	-	119.5	358.5
**[T2(Me2(H)Si)O]+**	1.80162	1.815	114.3	121.3	123.5	359.1
**H3S+**	1.364	-	94.8	-	-	284.4
**TH2S+**	1.356	2.378	94.1	99.6	-	293.3
**T2HS+**	1.354	2.299	-	99.4	112.5	311.3
**T3S+**	-	2.265	-	-	109.7	329.1
**T3S+ (planar)**		**2.255**	**-**	**-**	120.0	360.0

Carborate reaction – Dication formation

Table S42. Selected structural data (bond lengths in Å, angles in °. *See Figure 54*

	$[T_3S]^+$ naked	$[T_3S]$cb **4S**	$[T_3O]^+$ naked	$[T_3O]$cb **4O**
Si-E	2.266	2.282	1.823	1.834
Si-E	2.265	2.242	1.822	1.830
Si-E	2.265	2.286	1.823	1.817
∅(Si-E)	2.265	2.270	1.823	1.827
Si1-E-Si2	109.8	111.1	119.5	124.6
Si2-E-Si3	109.7	124.1	119.5	117.5
Σ∢E	329.3	345.7	358.4	360.0
	**monocat, naked** **$5O^+$**	**[monocat]cb** **5O**	**dicat, naked** **$6O^{2+}$**	**$[dicat]cb_2$** **6O**
Si-O	1.756	1.707	1.859(term)	1.840
Si=O	1.571	1.604	1.770(ring)	1.779, 1.774 (ring)
Si-O-Si	180.0	152.3	131.3	130.7
Si-O-Si	-	-	97.4	97.6
O-Si-O		-	82.6 (ring)	81.9
Σ∢Si	360.0	-	345.9	342.7
Σ∢O		-	360.0	360.0
	**monocat_me_bridge** **$10O^+$**	**[monocat_me]cb** **10O**	**$[Me_3Si]$cb** **11**	**TOT[CB]T** **12O**
Si1-O	1.616	1.622	-	1.680
Si2-E	1.713	1.705	-	1.680
Si-C_{bridge}	2.118	2.051	-	-
Si-C_{bridge}	2.013	2.021	-	-
Si-O-Si	146.3	150.6	-	139.3
Si-C_{bridge}-Si	177.6	172.4	-	-
Σ∢Si	340.5	335.0	346.5	346.3 (T+)
Si-Cl	-	-	2.273	2.298

Figure S55. Computed structures that play an essential role in the dication formation.

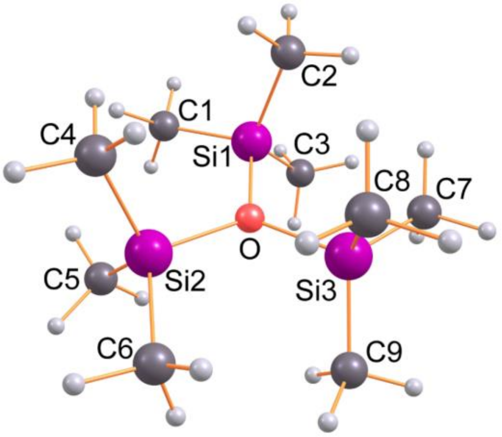

$[T_3O]^+$

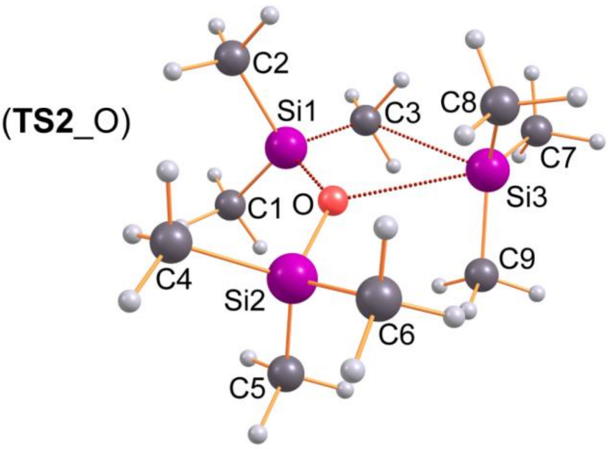

(**TS2**_O)

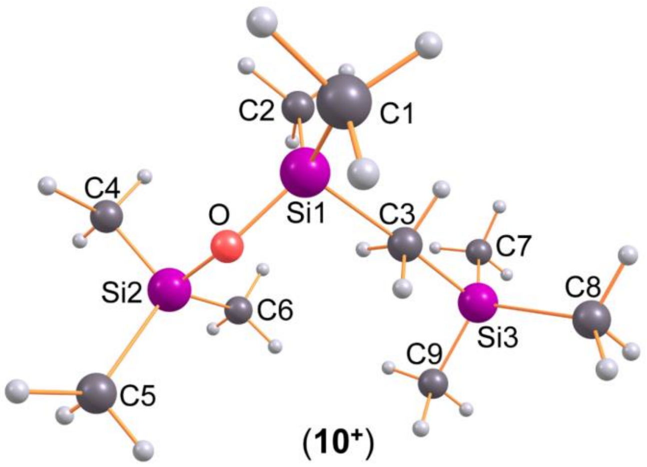

(**10**$^+$)

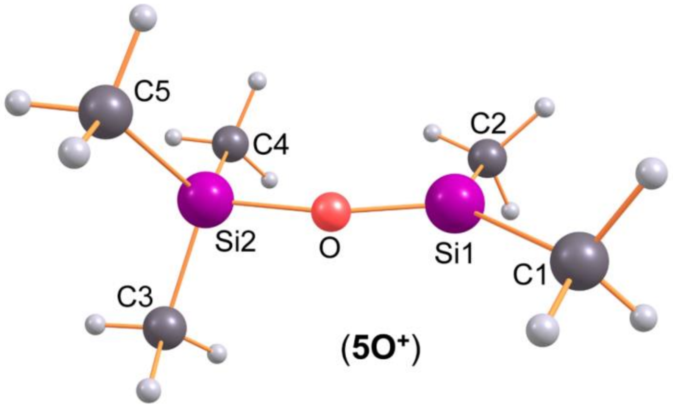

(**5O**$^+$)

(**4**O)

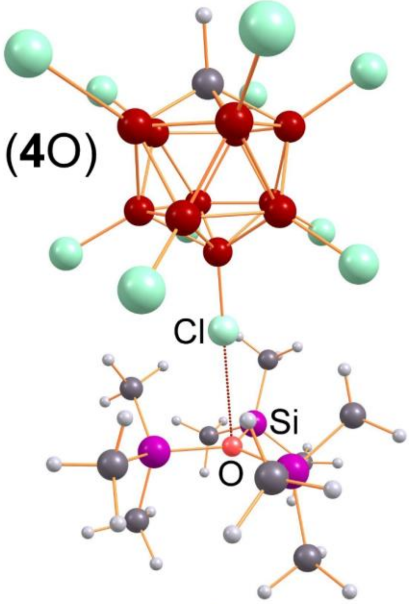

(**5**O)

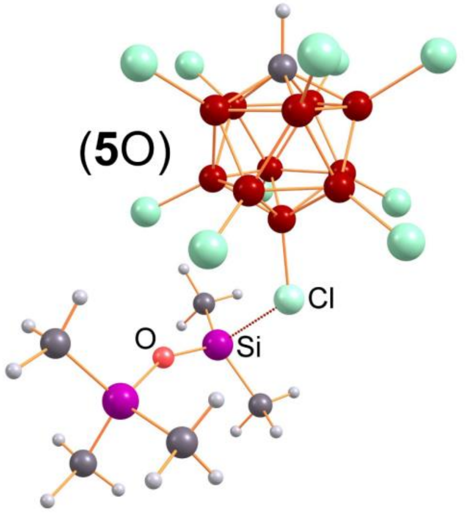

(**10**O)

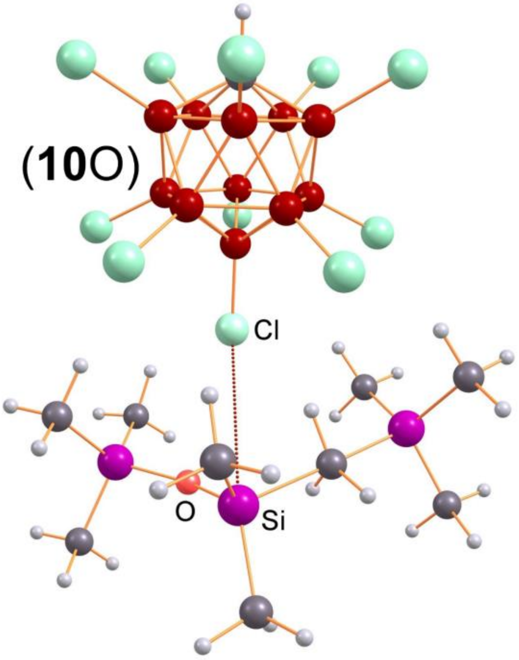

(**11**)

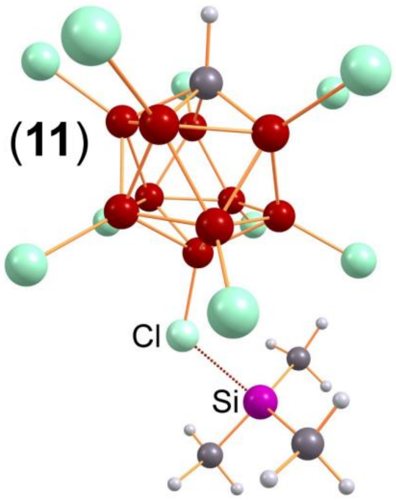

(**12**O)

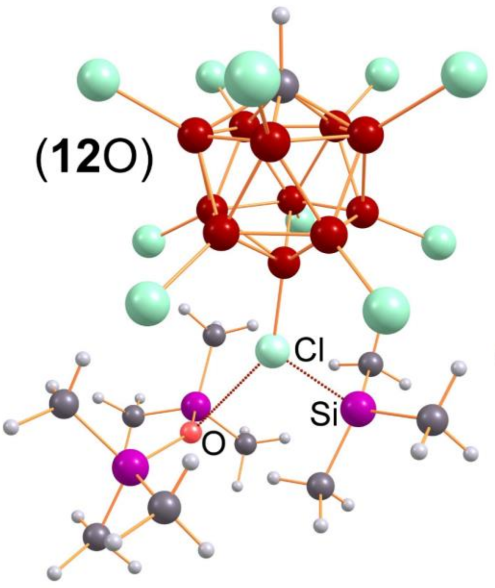

(**13**O)

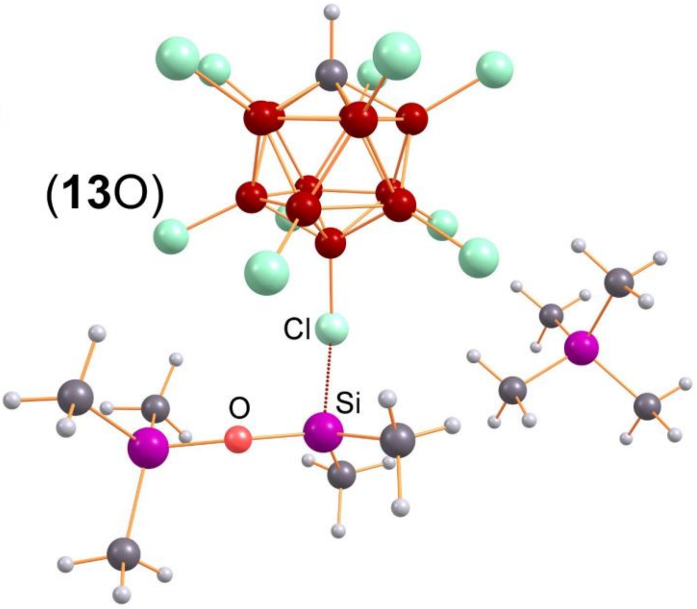

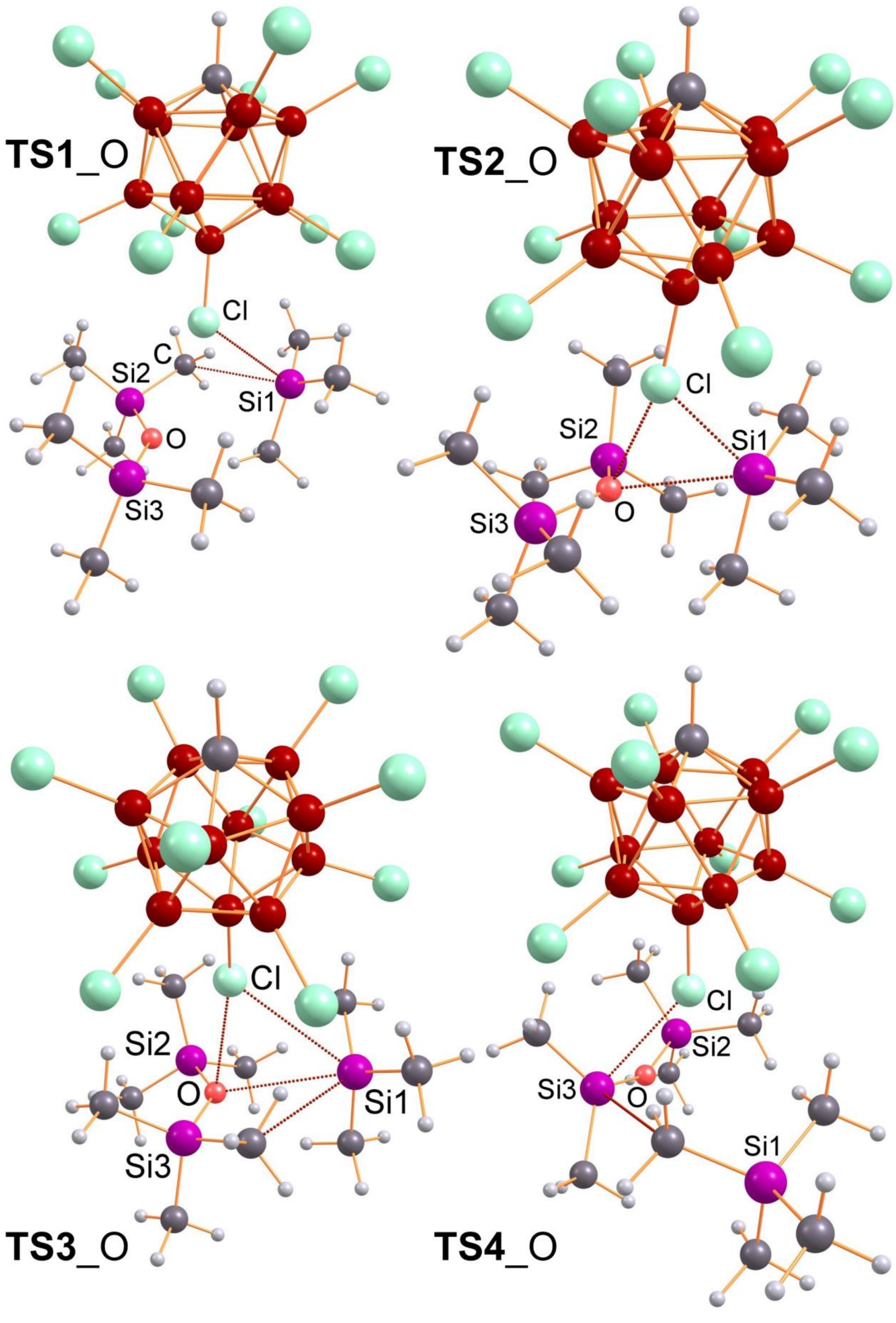
TS1_O
Cl
C
Si2
Si1
O
Si3
TS2_O
Cl
Si2
Si1
Si3
O
Cl
Si2
O
Si1
Si3
TS3_O
Cl
Si2
Si3
O
Si1
TS4_O

Table S43. Selected structural data of **6**E^{2+}(bond lengths in Å, angles in °. *See Figure 55*

**dications**	**Si$_{ring}$-E**	**Si$_{term}$-E**	**Si-E-Si ring**	**E-Si-E ring**	**Si-E-Si term**	**Si-E-Si-E ring**	**Si_term_dieder**	**sum angle E**
**O**	1.770	1.859	97.390	82.610	131.274	0	171.58482	359.900
**S**	2.230	2.355	88.731	91.269	117.743	0	121.39459	325

Table S44. Selected structural data (bond lengths in Å, angles in °. *See Figures S54-55*)

ion pairs	shortest E-Cl	shortest Cl-Si
[T]CB	-	2.273
[T_3O]CB (**4**O)	2.942	3.299
[T_3S]CB (**4**S)	3.300	3.332
[O_monocat]CB (**5**O)	3.160	2.309
[S_monocat]CB (**5**S)	3.539	2.303
[O-dicat]CB (**6O**)	2.865	3.438
[S-dicat]CB (**6S**)	3.451	3.507

Optimized structures

PBE1PBE/AUG-cc-pVDZ level of theory

Ligand Scrambling

Me_4Si

14	0.000038000	0.000233000	-0.000356000
6	0.421672000	-1.838849000	-0.065989000
1	0.714890000	-2.143996000	-1.080746000
1	1.255278000	-2.079954000	0.609159000
1	-0.438730000	-2.454716000	0.232631000
6	-1.431757000	0.360023000	-1.176680000
1	-2.326343000	-0.216760000	-0.901338000
1	-1.702805000	1.425391000	-1.159188000
1	-1.171341000	0.098555000	-2.212341000
6	1.509062000	1.010301000	-0.516298000
1	1.294750000	2.088299000	-0.488036000
1	2.360890000	0.821434000	0.152610000
1	1.825118000	0.759108000	-1.538943000
6	-0.498962000	0.468323000	1.759230000
1	0.316997000	0.272235000	2.469549000
1	-0.753935000	1.535480000	1.829823000
1	-1.375395000	-0.107123000	2.090230000

toluene

6	0.191727000	1.202500000	-0.000049000
6	0.914326000	0.006392000	-0.000058000
6	0.199949000	-1.198227000	-0.000048000
6	-1.192376000	-1.206576000	0.000009000
6	-1.901368000	-0.004581000	0.000045000
6	-1.203533000	1.200775000	0.000011000
1	0.729733000	2.152366000	-0.000096000
1	-1.728665000	-2.156508000	0.000007000
1	-2.991766000	-0.009497000	0.000077000
1	-1.746210000	2.147028000	0.000012000
6	2.416638000	0.003014000	0.000048000
1	2.811221000	-0.514821000	0.885928000
1	2.811385000	-0.517748000	-0.884026000
1	2.817198000	1.023942000	-0.001566000
1	0.744926000	-2.144546000	-0.000089000

Me_3SiH

14	-0.000065000	-0.000034000	-0.378019000
1	-0.000001000	-0.000033000	-1.883586000
6	0.752341000	1.621725000	0.223196000
1	0.768993000	1.663090000	1.322049000
1	1.785583000	1.736008000	-0.132978000
1	0.174974000	2.484743000	-0.136565000
6	-1.780670000	-0.159362000	0.223241000
1	-2.397823000	0.675250000	-0.137433000
1	-2.237324000	-1.093601000	-0.132021000
1	-1.825302000	-0.160018000	1.322093000
6	1.028403000	-1.462306000	0.223213000
1	0.613451000	-2.414241000	-0.136183000
1	2.065528000	-1.391454000	-0.133055000
1	1.052385000	-1.499617000	1.322045000

Me_2SiH_2

6	1.558202000	-0.512905000	0.000015000
14	0.000000000	0.549198000	-0.000050000
1	1.591848000	-1.158884000	-0.888650000
1	1.592311000	-1.158084000	0.889243000
1	2.461808000	0.111514000	-0.000500000
6	-1.558201000	-0.512906000	0.000075000
1	-0.000026000	1.438404000	-1.211180000
1	0.000026000	1.438597000	1.210939000
1	-1.592052000	-1.158625000	-0.888770000
1	-2.461805000	0.111517000	-0.000055000
1	-1.592112000	-1.158339000	0.889126000

Me_3Si+

6	-1.509621000	-1.044270000	-0.003915000
14	0.000136000	-0.000463000	-0.017337000
1	-1.740830000	-1.268808000	1.054563000
1	-1.355783000	-2.004779000	-0.512207000
1	-2.378261000	-0.518011000	-0.420606000
6	1.660635000	-0.782585000	-0.003974000
6	-0.151215000	1.828125000	0.016366000
1	1.920580000	-0.976799000	1.053838000
1	2.433390000	-0.117956000	-0.411500000
1	1.667934000	-1.750818000	-0.520963000
1	0.714131000	2.304503000	0.495380000
1	-1.087279000	2.155757000	0.487036000
1	-0.174575000	2.175770000	-1.033689000

H_2SiMe+

14	0.639498000	-0.000111000	-0.006541000
6	-1.183818000	-0.000888000	-0.010921000
1	-1.494270000	0.025754000	1.054789000
1	-1.592468000	0.899516000	-0.490066000
1	-1.594313000	-0.920287000	-0.449765000
1	1.413800000	1.267874000	0.020928000
1	1.417187000	-1.265975000	0.021215000

T-H-Si(H)Me_2+

14	-1.625288000	-0.028100000	-0.275296000
14	1.399472000	-0.012491000	0.012690000
6	-2.267393000	1.620956000	0.289352000
1	-1.636887000	2.444463000	-0.068011000
1	-3.275884000	1.764474000	-0.126997000
1	-2.344515000	1.669908000	1.383047000
6	-2.452502000	-1.570804000	0.346390000
1	-2.549281000	-1.563148000	1.439552000
1	-3.464540000	-1.620727000	-0.083069000
1	-1.910299000	-2.472709000	0.036079000
6	1.639171000	-1.535285000	-1.024025000
1	1.100989000	-1.481396000	-1.978280000
1	2.712223000	-1.628581000	-1.252571000
1	1.339557000	-2.444224000	-0.486612000
6	1.497794000	1.626742000	-0.857484000
1	0.940373000	1.637459000	-1.802424000
1	1.152621000	2.449279000	-0.218139000
1	2.554849000	1.820713000	-1.097153000
6	2.103973000	-0.069795000	1.732232000
1	1.853665000	-1.006605000	2.245353000
1	3.201047000	-0.013338000	1.654216000
1	1.768761000	0.778596000	2.341764000
1	-0.195785000	-0.119812000	0.477255000

1	-1.211736000	-0.076965000	-1.706316000

T-Me-Si(H)Me_2+

14	-1.865853000	0.000112000	-0.036804000
14	2.258829000	-0.000169000	0.441370000
6	-2.461905000	-0.008736000	1.725250000
1	-2.133115000	0.880032000	2.279602000
1	-3.562483000	-0.005463000	1.720080000
1	-2.138355000	-0.906438000	2.268183000
6	-2.199744000	1.573949000	-0.970950000
1	-1.722197000	1.581964000	-1.959496000
1	-3.285651000	1.658686000	-1.129138000
1	-1.878982000	2.463356000	-0.412885000
6	-2.199487000	-1.564924000	-0.985753000
1	-1.878205000	-2.459427000	-0.436193000
1	-3.285441000	-1.648552000	-1.144261000
1	-1.722383000	-1.563549000	-1.974538000
6	0.264167000	-0.000577000	0.171078000
1	0.089362000	0.942097000	0.701995000
1	0.274605000	0.001649000	-0.924365000
1	0.089697000	-0.945476000	0.698123000
6	2.887051000	1.575718000	-0.331038000
1	2.447753000	2.466303000	0.137172000
1	3.976699000	1.633628000	-0.196659000
1	2.684822000	1.607643000	-1.410421000
6	2.888018000	-1.575310000	-0.331746000
1	2.449903000	-2.466336000	0.136735000
1	2.685059000	-1.607355000	-1.410983000
1	3.977816000	-1.632179000	-0.198121000
1	2.360833000	-0.000516000	1.930191000

T_2HSi+

14	0.000610000	0.457463000	-0.001419000
6	1.620808000	-0.393100000	-0.000534000
6	-1.606428000	-0.416568000	-0.000440000
1	1.687883000	-1.031026000	0.898349000
1	-2.172449000	-0.110070000	0.897032000
1	1.672635000	-1.078577000	-0.863751000
1	-2.202534000	-0.069819000	-0.862311000
1	2.464280000	0.306056000	-0.023706000
1	-1.507563000	-1.507801000	-0.024860000
1	-0.037069000	1.944762000	0.004950000

TS1

14	-1.595784000	0.000008000	0.201402000
14	1.571677000	0.000086000	0.072899000
6	-1.489412000	-1.629316000	1.071501000
1	-1.022296000	-1.553582000	2.058615000
1	-2.517594000	-2.011963000	1.177822000
1	-0.939012000	-2.362073000	0.465267000
6	-1.489710000	1.629157000	1.071872000
1	-0.939349000	2.362110000	0.465832000
1	-2.517943000	2.011665000	1.178218000
1	-1.022633000	1.553260000	2.058993000
6	-2.684616000	0.000094000	-1.295748000
1	-2.569575000	-0.896881000	-1.915404000
1	-3.717440000	-0.000575000	-0.903002000
1	-2.570424000	0.897692000	-1.914662000
6	0.490875000	-0.000321000	-1.514202000
1	-0.093898000	0.903917000	-1.737149000
1	1.269938000	0.000115000	-2.297582000
1	-0.092953000	-0.905196000	-1.736987000
6	2.565742000	1.582275000	0.108545000
1	1.921018000	2.471071000	0.087302000
1	3.180178000	1.631672000	1.017197000
1	3.240076000	1.632818000	-0.757851000
6	2.565847000	-1.582028000	0.109094000
1	1.921142000	-2.470853000	0.088467000
1	3.239957000	-1.632976000	-0.757453000
1	3.180517000	-1.630948000	1.017613000
1	0.635420000	0.000250000	1.258176000

T-O-T

8	0.000000000	0.000000000	0.749339000
14	0.000000000	1.549541000	0.083508000
14	0.000000000	-1.549541000	0.083508000
6	1.729293000	1.972647000	-0.519355000
1	2.039350000	1.316484000	-1.344039000
1	1.775147000	3.010416000	-0.879924000
1	2.461129000	1.864291000	0.293110000
6	-1.213422000	1.636553000	-1.352695000
1	-2.229015000	1.368658000	-1.030372000
1	-1.247753000	2.653945000	-1.767833000
1	-0.923825000	0.956468000	-2.166248000
6	-0.519470000	2.715588000	1.455152000
1	0.176426000	2.652844000	2.302985000
1	-0.535424000	3.757303000	1.104297000
1	-1.523793000	2.464533000	1.822952000
6	-1.729293000	-1.972647000	-0.519355000
1	-2.039350000	-1.316484000	-1.344039000
1	-1.775147000	-3.010416000	-0.879924000
1	-2.461129000	-1.864291000	0.293110000
6	1.213422000	-1.636553000	-1.352695000
1	0.923825000	-0.956468000	-2.166248000
1	2.229015000	-1.368658000	-1.030372000
1	1.247753000	-2.653945000	-1.767833000
6	0.519470000	-2.715588000	1.455152000
1	-0.176426000	-2.652844000	2.302985000
1	0.535424000	-3.757303000	1.104297000
1	1.523793000	-2.464533000	1.822952000

$T_2(Me_2(H)Si)O+$

8	0.003802000	-0.121402000	-0.159720000
14	-0.863204000	1.470757000	0.008678000
14	1.823970000	-0.298139000	-0.029940000
14	-0.896303000	-1.684447000	-0.034604000
6	-0.161064000	2.626051000	-1.274700000
1	0.082904000	2.092804000	-2.203462000
1	-0.943070000	3.359399000	-1.519411000
1	0.720480000	3.181476000	-0.940936000
6	-0.602509000	1.958637000	1.787068000
1	-1.085690000	2.928623000	1.971813000
1	-1.057320000	1.225753000	2.466631000
1	0.456635000	2.063373000	2.051788000
6	-2.660032000	1.170940000	-0.388878000
1	-3.168571000	0.421405000	0.226133000
1	-3.161037000	2.132385000	-0.195380000
1	-2.814152000	0.938490000	-1.449586000
6	2.607000000	1.390499000	-0.094458000
1	3.673262000	1.215579000	0.119081000
1	2.549673000	1.851978000	-1.086126000
1	2.245509000	2.097253000	0.660586000
6	2.314514000	-1.290401000	-1.527757000
1	1.990422000	-2.336234000	-1.495602000
1	1.923483000	-0.825680000	-2.443487000
1	3.411821000	-1.284185000	-1.602454000
6	2.109724000	-1.093235000	1.632042000
1	1.700760000	-2.106693000	1.713733000
1	3.194207000	-1.162516000	1.800729000
1	1.693298000	-0.474923000	2.438954000
6	-1.727373000	-1.736047000	1.629402000
1	-2.525678000	-0.996322000	1.756266000
1	-2.176564000	-2.732122000	1.754939000
1	-0.990387000	-1.602722000	2.432149000
6	-1.949215000	-1.902852000	-1.549711000
1	-2.908515000	-1.377817000	-1.505667000
1	-1.406999000	-1.588031000	-2.451267000
1	-2.159414000	-2.977788000	-1.651742000
1	0.235758000	-2.648202000	-0.065845000

T_3O+

8	0.000000000	0.000000000	0.195571000
14	-0.760015000	1.652994000	0.016528000
14	-1.051527000	-1.484690000	0.016528000
14	1.811542000	-0.168305000	0.016528000
6	-1.986037000	1.833854000	1.412082000
1	-1.647013000	1.299645000	2.310007000
1	-2.035849000	2.901119000	1.670759000
1	-2.999291000	1.503342000	1.166773000
6	-1.441396000	1.686944000	-1.719187000
1	-1.945558000	2.651175000	-1.877661000
1	-0.629930000	1.614801000	-2.455576000
1	-2.170536000	0.897858000	-1.932928000
6	0.535648000	2.977176000	0.231220000
1	1.399843000	2.927106000	-0.437873000
1	0.000000000	3.909761000	-0.008865000
1	0.879898000	3.065456000	1.267761000
6	-2.846134000	-1.024703000	0.231220000
1	-3.385952000	-1.954880000	-0.008865000
1	-3.094712000	-0.770714000	1.267761000
1	-3.234870000	-0.251253000	-0.437873000
6	-0.595146000	-2.636886000	1.412082000
1	0.197713000	-3.349133000	1.166773000
1	-0.302019000	-2.076178000	2.310007000
1	-1.494518000	-3.213656000	1.670759000
6	-0.740238000	-2.091758000	-1.719187000
1	0.307701000	-2.328669000	-1.932928000
1	-1.323206000	-3.010490000	-1.877661000
1	-1.083493000	-1.352936000	-2.455576000
6	2.310486000	-1.952473000	0.231220000
1	3.385952000	-1.954880000	-0.008865000
1	2.214813000	-2.294742000	1.267761000
1	1.835027000	-2.675853000	-0.437873000
6	2.181634000	0.404814000	-1.719187000
1	1.862836000	1.430811000	-1.932928000
1	3.268763000	0.359315000	-1.877661000
1	1.713424000	-0.261865000	-2.455576000
6	2.581183000	0.803032000	1.412082000
1	2.801578000	1.845791000	1.166773000
1	1.949032000	0.776533000	2.310007000
1	3.530367000	0.312537000	1.670759000

H_3O+

8	0.000000000	0.000000000	0.077194000
1	0.000000000	0.937847000	-0.205850000
1	-0.812200000	-0.468924000	-0.205850000
1	0.812200000	-0.468924000	-0.205850000

TH_2O+

8	0.004158000	-0.313956000	1.699006000
14	-0.000296000	0.030417000	-0.201010000
6	1.588089000	-0.807051000	-0.648667000
1	1.578852000	-1.873521000	-0.389578000
1	1.723675000	-0.731875000	-1.738636000
1	2.460515000	-0.325609000	-0.186098000
6	-0.014216000	1.880432000	-0.300376000
1	-0.019692000	2.171643000	-1.362358000
1	-0.911636000	2.315390000	0.159215000
1	0.878925000	2.329353000	0.154035000
6	-1.577452000	-0.829550000	-0.645827000
1	-2.455992000	-0.359966000	-0.182635000
1	-1.715317000	-0.757092000	-1.735704000
1	-1.553135000	-1.895625000	-0.386090000
1	-0.784508000	-0.131502000	2.230322000
1	0.790672000	-0.118378000	2.228843000

T_2HO+

8	-0.000010000	-0.000491000	0.743152000
14	-1.692135000	0.008857000	0.016532000
14	1.692116000	-0.008852000	0.016573000
6	-1.995712000	-1.741162000	-0.524760000
1	-1.874762000	-2.449165000	0.305705000
1	-3.030951000	-1.829601000	-0.886063000
1	-1.333557000	-2.042484000	-1.345766000
6	-1.573824000	1.246165000	-1.363624000
1	-2.565916000	1.346664000	-1.827761000
1	-1.276427000	2.237685000	-0.999246000
1	-0.881043000	0.929549000	-2.153901000
6	-2.705378000	0.553343000	1.476411000
1	-2.410459000	1.547223000	1.838884000
1	-3.760850000	0.616567000	1.173684000
1	-2.655932000	-0.163373000	2.308136000
6	1.573879000	-1.244395000	-1.365175000
1	2.565736000	-1.343597000	-1.830080000
1	1.277335000	-2.236603000	-1.001970000
1	0.880395000	-0.927192000	-2.154607000
6	2.705356000	-0.555245000	1.475746000
1	2.656536000	0.160674000	2.308188000
1	2.409989000	-1.549329000	1.837295000
1	3.760711000	-0.618781000	1.172659000
6	1.995719000	1.741814000	-0.522603000
1	1.874642000	2.448866000	0.308654000
1	3.031001000	1.830700000	-0.883670000
1	1.333660000	2.044045000	-1.343356000
1	-0.000001000	-0.001116000	1.708578000

T-S-T

16	0.000000000	0.000000000	1.289863000
14	0.000000000	1.731985000	-0.023697000
14	0.000000000	-1.731985000	-0.023697000
6	0.523095000	-3.152978000	1.090953000
1	-0.161150000	-3.253752000	1.944336000
1	0.516815000	-4.102150000	0.535489000
1	1.535223000	-2.991158000	1.484880000
6	-1.730074000	-2.048596000	-0.693173000
1	-1.749799000	-2.981743000	-1.275761000
1	-2.448127000	-2.143890000	0.132142000
1	-2.069280000	-1.233903000	-1.344920000
6	1.227026000	-1.544619000	-1.440188000
1	0.935458000	-0.754365000	-2.144155000
1	2.231613000	-1.316037000	-1.060921000
1	1.280932000	-2.487751000	-2.003787000
6	-1.227026000	1.544619000	-1.440188000
1	-0.935458000	0.754365000	-2.144155000
1	-2.231613000	1.316037000	-1.060921000
1	-1.280932000	2.487751000	-2.003787000
6	1.730074000	2.048596000	-0.693173000
1	1.749799000	2.981743000	-1.275761000
1	2.448127000	2.143890000	0.132142000
1	2.069280000	1.233903000	-1.344920000
6	-0.523095000	3.152978000	1.090953000
1	0.161150000	3.253752000	1.944336000
1	-0.516815000	4.102150000	0.535489000
1	-1.535223000	2.991158000	1.484880000

$T_2(Me_2(H)Si)S+$

16	0.009600000	-0.128616000	-0.817859000
14	-1.112508000	1.657966000	0.023374000
14	2.136376000	-0.186996000	-0.008028000
14	-0.936377000	-2.013317000	-0.030114000
6	-0.530394000	3.062589000	-1.056915000
1	-0.709848000	2.850617000	-2.118630000
1	-1.098525000	3.967021000	-0.793090000
1	0.533673000	3.285989000	-0.917689000
6	-0.679358000	1.866467000	1.828616000
1	-1.242335000	2.726849000	2.219933000
1	-0.953522000	0.989878000	2.427078000
1	0.386364000	2.079878000	1.977075000
6	-2.907337000	1.233282000	-0.275574000
1	-3.264646000	0.405856000	0.348923000
1	-3.513007000	2.116808000	-0.025670000
1	-3.093653000	0.992879000	-1.330183000
6	2.761921000	1.560046000	-0.212319000
1	3.835885000	1.566708000	0.025238000
1	2.649209000	1.913462000	-1.245092000
1	2.271770000	2.268714000	0.466100000
6	2.960330000	-1.398887000	-1.160735000
1	2.517504000	-2.400429000	-1.091191000
1	2.905658000	-1.061187000	-2.203436000
1	4.022469000	-1.484417000	-0.887261000
6	2.088079000	-0.761726000	1.768296000
1	1.719447000	-1.791176000	1.855371000
1	3.116600000	-0.750552000	2.158820000
1	1.483583000	-0.105697000	2.405865000
6	-1.502272000	-1.808005000	1.737806000
1	-2.313506000	-1.075982000	1.831541000
1	-1.891911000	-2.777492000	2.081560000
1	-0.680982000	-1.520911000	2.403956000
6	-2.287523000	-2.426201000	-1.244817000
1	-3.086860000	-1.674504000	-1.232489000
1	-1.893174000	-2.504188000	-2.265487000
1	-2.731191000	-3.394927000	-0.973192000
1	0.231846000	-2.937884000	-0.131716000

T_3S+

16	0.000000000	0.000000000	0.787240000
14	0.000000000	2.129692000	-0.003374000
14	-1.844367000	-1.064846000	-0.003374000
14	1.844367000	-1.064846000	-0.003374000
6	1.482790000	-2.881657000	0.237269000
1	0.701419000	-3.257923000	-0.433037000
1	2.405631000	-3.435452000	0.009534000
1	1.208998000	-3.109074000	1.275075000
6	3.205500000	-0.481641000	1.132207000
1	4.117625000	-1.051558000	0.900540000
1	3.437484000	0.581792000	1.007778000
1	2.952294000	-0.666970000	2.183912000
6	2.102479000	-0.608619000	-1.797651000
1	2.953594000	-1.192260000	-2.178841000
1	1.229893000	-0.852346000	-2.415060000
1	2.348921000	0.451386000	-1.932233000
6	-1.185636000	3.016865000	1.132207000
1	-0.898534000	2.890247000	2.183912000
1	-1.148136000	4.091747000	0.900540000
1	-2.222588000	2.686052000	1.007778000
6	1.754193000	2.724963000	0.237269000
1	1.772373000	3.801064000	0.009534000
1	2.088038000	2.601560000	1.275075000
1	2.470734000	2.236408000	-0.433037000
6	-0.524160000	2.125110000	-1.797651000
1	0.123206000	1.491291000	-2.415060000
1	-1.565372000	1.808532000	-1.932233000
1	-0.444270000	3.154018000	-2.178841000
6	-1.578319000	-1.516490000	-1.797651000
1	-2.509325000	-1.961758000	-2.178841000
1	-1.353099000	-0.638946000	-2.415060000
1	-0.783548000	-2.259918000	-1.932233000
6	-2.019864000	-2.535224000	1.132207000
1	-1.214895000	-3.267844000	1.007778000
1	-2.053761000	-2.223277000	2.183912000
1	-2.969489000	-3.040189000	0.900540000
6	-3.236983000	0.156695000	0.237269000
1	-4.178004000	-0.365612000	0.009534000
1	-3.297036000	0.507514000	1.275075000
1	-3.172153000	1.021514000	-0.433037000

H_3S+

16	0.000000000	0.000000000	0.115609000
1	0.000000000	1.157676000	-0.616582000
1	-1.002577000	-0.578838000	-0.616582000
1	1.002577000	-0.578838000	-0.616582000

TH_2S+

16	1.827491000	-0.002902000	-0.095364000
14	-0.551655000	-0.000545000	0.001337000
6	-0.905374000	1.520271000	-1.006645000
1	-0.503081000	1.446520000	-2.025218000
1	-1.998599000	1.623540000	-1.090495000
1	-0.527752000	2.435546000	-0.532426000
6	-0.937543000	0.108227000	1.816157000
1	-2.033214000	0.107762000	1.930333000
1	-0.550972000	-0.751485000	2.378544000
1	-0.563130000	1.036115000	2.267485000
6	-0.901492000	-1.631076000	-0.819181000
1	-0.530022000	-2.483228000	-0.235272000
1	-1.994271000	-1.741528000	-0.899369000
1	-0.490963000	-1.680834000	-1.835957000
1	2.072030000	-0.951973000	0.849204000
1	2.069756000	1.029089000	0.758290000

T_2HS+

16	-0.000813000	-1.200214000	0.125782000
14	1.902900000	0.092058000	-0.002815000
14	-1.907316000	0.095226000	-0.008748000
6	2.095183000	0.644468000	1.766026000
1	2.152287000	-0.210476000	2.451985000
1	3.036128000	1.207416000	1.856027000
1	1.282273000	1.306181000	2.088901000
6	1.606964000	1.463449000	-1.231069000
1	2.545083000	2.028397000	-1.340898000
1	1.340210000	1.076055000	-2.222609000
1	0.836550000	2.170231000	-0.899718000
6	3.156349000	-1.166708000	-0.564982000
1	2.947327000	-1.535348000	-1.577779000
1	4.147590000	-0.689179000	-0.586172000
1	3.212460000	-2.021968000	0.120436000
6	-1.618369000	1.413617000	1.277601000
1	-2.548257000	1.990049000	1.392531000
1	-1.377815000	0.983308000	2.258488000
1	-0.829115000	2.118331000	0.988578000
6	-3.222490000	-1.142130000	0.446255000
1	-3.260430000	-1.978817000	-0.263524000
1	-3.071118000	-1.544296000	1.456088000
1	-4.203066000	-0.643302000	0.424074000
6	-2.010235000	0.701780000	-1.768315000
1	-2.050111000	-0.130909000	-2.482962000
1	-2.943530000	1.274017000	-1.882037000
1	-1.179214000	1.364052000	-2.036782000
1	-0.006822000	-1.669147000	-1.148365000

Carborate reaction – Dication formation

PBE1PBE/def2svp empiricaldispersion=gd3bj

toluene

6	-0.195022000	-1.200757000	-0.009683000
6	-0.915123000	-0.000360000	-0.012185000
6	-0.195304000	1.200619000	-0.009677000
6	1.197925000	1.203654000	0.001948000
6	1.901426000	0.000145000	0.009408000
6	1.198514000	-1.203297000	0.001936000
1	-0.737352000	-2.150660000	-0.019348000
1	1.738398000	2.153572000	0.001895000
1	2.993970000	0.000537000	0.015559000
1	1.738841000	-2.153315000	0.001890000
6	-2.416557000	-0.000087000	0.009633000
1	-2.827461000	0.887238000	-0.493500000
1	-2.827950000	-0.894032000	-0.481177000
1	-2.795060000	0.007163000	1.045767000
1	-0.738541000	2.149989000	-0.019361000

Me_4Si

14	0.000070000	0.000040000	-0.000434000
6	0.026887000	0.791991000	1.709219000
1	0.018240000	1.891420000	1.638217000
1	-0.849980000	0.487022000	2.302458000
1	0.929405000	0.498769000	2.269062000
6	1.515896000	0.554264000	-0.972635000
1	2.445726000	0.258697000	-0.460880000
1	1.529246000	0.105720000	-1.978888000
1	1.532251000	1.649293000	-1.092883000
6	-1.561336000	0.529587000	-0.912810000
1	-1.606967000	0.079846000	-1.917556000
1	-2.465502000	0.219652000	-0.364808000
1	-1.600077000	1.624109000	-1.032697000
6	0.018468000	-1.875901000	0.176584000
1	-0.858325000	-2.230489000	0.741712000
1	0.004397000	-2.368402000	-0.808856000
1	0.921123000	-2.215847000	0.709041000

T^+

6	-0.603968000	1.725414000	0.000867000
14	0.000061000	0.000007000	0.000027000
1	-1.277405000	1.880795000	0.863237000
1	-1.223779000	1.893310000	-0.899297000
1	0.204339000	2.469283000	0.028282000
6	-1.192353000	-1.385743000	-0.000854000
6	1.796209000	-0.339719000	-0.000106000
1	-1.025580000	-2.008789000	0.897261000
1	-0.992698000	-2.044485000	-0.865443000
1	-2.240620000	-1.057223000	-0.024449000
1	2.035928000	-1.412019000	-0.005599000
1	2.258839000	0.134749000	0.884694000
1	2.260784000	0.144563000	-0.878508000

E = oxogen

Naked

T-O-T

8	0.000000000	0.000000000	0.595815000
14	0.000000000	1.581470000	0.068980000
14	0.000000000	-1.581470000	0.068980000
6	1.735906000	2.043681000	-0.471694000
1	2.065675000	1.422695000	-1.319392000
1	1.789885000	3.098683000	-0.784768000
1	2.451534000	1.897521000	0.352661000
6	-1.192397000	1.756344000	-1.371973000
1	-2.209421000	1.450949000	-1.079248000
1	-1.240949000	2.798866000	-1.725236000
1	-0.882655000	1.129840000	-2.223933000
6	-0.545654000	2.644641000	1.508682000
1	0.133540000	2.515707000	2.365760000
1	-0.554193000	3.712218000	1.237368000
1	-1.559262000	2.367432000	1.837713000
6	-1.735906000	-2.043681000	-0.471694000
1	-2.065675000	-1.422695000	-1.319392000
1	-1.789885000	-3.098683000	-0.784768000
1	-2.451534000	-1.897521000	0.352661000
6	1.192397000	-1.756344000	-1.371973000
1	0.882655000	-1.129840000	-2.223933000
1	2.209421000	-1.450949000	-1.079248000
1	1.240949000	-2.798866000	-1.725236000
6	0.545654000	-2.644641000	1.508682000
1	-0.133540000	-2.515707000	2.365760000
1	0.554193000	-3.712218000	1.237368000
1	1.559262000	-2.367432000	1.837713000

T_3O^+

8	0.000516000	-0.000701000	-0.151179000
14	-0.772137000	1.644750000	-0.015624000
14	1.811780000	-0.154520000	-0.015468000
14	-1.039128000	-1.491150000	-0.015661000
6	-0.126424000	2.674537000	-1.425966000
1	0.050742000	2.061286000	-2.323176000
1	-0.909851000	3.404905000	-1.684519000
1	0.787263000	3.235713000	-1.197033000
6	-0.363475000	2.216303000	1.707816000
1	-0.796880000	3.218557000	1.856860000
1	-0.817237000	1.547745000	2.455290000
1	0.711535000	2.289918000	1.918881000
6	-2.617836000	1.513285000	-0.220278000
1	-3.141454000	0.829118000	0.458758000
1	-2.978056000	2.530646000	0.013995000
1	-2.916751000	1.300845000	-1.255581000
6	2.616023000	1.512386000	-0.215128000
1	3.677804000	1.319412000	0.019453000
1	2.580531000	1.880084000	-1.249529000
1	2.281314000	2.305118000	0.465292000
6	2.384782000	-1.224152000	-1.427248000
1	2.414195000	-2.296213000	-1.199624000
1	1.767966000	-1.069819000	-2.326226000
1	3.409513000	-0.909463000	-1.682023000
6	2.101456000	-0.797207000	1.706818000
1	1.632828000	-1.767817000	1.915998000
1	3.186555000	-0.917416000	1.857814000
1	1.743711000	-0.072918000	2.454463000
6	-0.001657000	-3.023690000	-0.216074000
1	-0.704675000	-3.844594000	0.011133000
1	0.338277000	-3.173370000	-1.249742000
1	0.848014000	-3.137692000	0.468427000
6	-1.742781000	-1.420475000	1.705743000
1	-2.349044000	-0.528875000	1.913157000
1	-2.390205000	-2.299546000	1.856382000
1	-0.938014000	-1.472893000	2.454853000
6	-2.251036000	-1.449387000	-1.428477000
1	-3.192303000	-0.934248000	-1.203170000
1	-1.806033000	-0.996035000	-2.327865000
1	-2.495385000	-2.493557000	-1.681545000

monocat_w

14	1.530485000	-0.003069000	-0.000407000
8	-0.225908000	-0.004483000	0.003775000
6	1.994452000	0.871498000	1.571514000
1	1.609045000	1.902189000	1.591866000
1	3.092065000	0.926700000	1.652995000
1	1.624945000	0.338691000	2.460902000
6	1.984238000	0.929214000	-1.541966000
1	1.618725000	0.424088000	-2.449133000
1	3.081022000	0.998569000	-1.623314000
1	1.588360000	1.956095000	-1.525646000
6	2.015778000	-1.795630000	-0.035739000
1	1.644860000	-2.335282000	0.849239000
1	3.113750000	-1.886769000	-0.041499000
1	1.638306000	-2.299233000	-0.938928000
14	-1.796813000	-0.005744000	0.007515000
6	-2.696619000	-1.596262000	-0.004784000
1	-3.254895000	-1.704031000	0.942075000
1	-3.444582000	-1.598465000	-0.815655000
1	-2.015954000	-2.450619000	-0.121826000
6	-2.669234000	1.600820000	0.002995000
1	-2.139009000	2.336241000	0.627244000
1	-2.680882000	1.994816000	-1.029397000
1	-3.711586000	1.498419000	0.339241000

monocat_u

8	-0.221057000	-0.334621000	0.011579000
14	-1.769387000	-0.049239000	0.002762000
14	1.511919000	-0.023921000	-0.000486000
6	-2.907204000	-1.477412000	0.002792000
1	-2.368927000	-2.416903000	0.190448000
1	-3.699637000	-1.338914000	0.756638000
1	-3.407652000	-1.542379000	-0.979724000
6	-2.386546000	1.672834000	-0.005205000
1	-2.843899000	1.897080000	0.975020000
1	-1.578939000	2.394207000	-0.192598000
1	-3.180022000	1.792615000	-0.761296000
6	2.095764000	-0.658739000	1.642652000
1	1.882006000	-1.732070000	1.758461000
1	3.186485000	-0.525728000	1.725933000
1	1.631859000	-0.115472000	2.480035000
6	2.134145000	-0.987030000	-1.458958000
1	1.920333000	-2.061240000	-1.351787000
1	1.690510000	-0.633219000	-2.402103000
1	3.226759000	-0.871141000	-1.543014000
6	1.681303000	1.819770000	-0.193742000
1	2.748263000	2.094583000	-0.220238000
1	1.232935000	2.177425000	-1.133968000
1	1.228155000	2.365824000	0.648464000

monocat_methyl bridged

8	-1.418239000	-0.018724000	-0.067312000
14	3.201970000	-0.892207000	-0.017302000
14	-3.100000000	-0.314561000	-0.007450000
14	-0.340543000	1.196033000	0.011645000
6	4.428009000	0.264519000	0.773369000
1	4.111365000	0.633174000	1.761753000
1	5.355275000	-0.313477000	0.938484000
1	4.691717000	1.120548000	0.135150000
6	3.539139000	-1.248118000	-1.809422000
1	4.502213000	-1.782739000	-1.867061000
1	2.773372000	-1.904152000	-2.254784000
1	3.630543000	-0.338643000	-2.422993000
6	2.856287000	-2.419333000	0.987747000
1	2.685525000	-2.179765000	2.050883000
1	1.984240000	-2.980162000	0.619853000
1	3.733172000	-3.084196000	0.936945000
6	-3.898130000	0.920802000	-1.162797000
1	-4.997028000	0.861157000	-1.117245000
1	-3.620509000	1.954040000	-0.894983000
1	-3.599050000	0.746814000	-2.208802000
6	-3.633958000	-0.040716000	1.762551000
1	-3.435497000	0.989418000	2.099535000
1	-4.716931000	-0.214450000	1.871328000
1	-3.122185000	-0.731888000	2.451033000
6	-3.349200000	-2.072060000	-0.562672000
1	-2.868214000	-2.789710000	0.119759000
1	-4.423053000	-2.312492000	-0.601306000
1	-2.936715000	-2.231207000	-1.571867000
6	-0.287462000	2.237784000	-1.519496000
1	-1.213605000	2.825340000	-1.616460000
1	0.556174000	2.946186000	-1.514819000
1	-0.206412000	1.604216000	-2.416694000
6	-0.348898000	2.117832000	1.618678000
1	-0.340011000	1.444445000	2.488533000
1	0.506323000	2.808541000	1.697417000
1	-1.262762000	2.731574000	1.679584000
6	1.428040000	0.153489000	-0.002807000
1	1.974756000	1.077334000	-0.230333000
1	1.254747000	-0.134546000	1.041284000
1	1.014058000	-0.467194000	-0.807720000

monocat_SiMe4_adduct

8	1.587070000	0.423759000	-0.547554000
14	-2.988246000	-0.551440000	0.005266000
14	2.612328000	-0.855877000	-0.053037000
14	0.552014000	1.574091000	-0.080958000
6	-2.475170000	-2.291428000	-0.418263000
1	-1.799830000	-2.716390000	0.340105000
1	-3.370210000	-2.933496000	-0.457709000
1	-1.983826000	-2.352077000	-1.401598000
6	-4.076712000	0.238180000	-1.282835000
1	-5.038954000	-0.298095000	-1.321805000
1	-4.297903000	1.291178000	-1.050009000
1	-3.631867000	0.186764000	-2.288529000
6	-3.639584000	-0.375308000	1.741357000
1	-3.860939000	0.672304000	1.997367000
1	-4.580919000	-0.941261000	1.834313000
1	-2.939551000	-0.779416000	2.488817000
6	1.538763000	-2.002773000	0.969155000
1	2.119888000	-2.875142000	1.308217000
1	1.150684000	-1.507896000	1.874888000
1	0.689982000	-2.388277000	0.381906000
6	3.981212000	-0.104494000	0.963931000
1	4.548026000	0.636616000	0.379274000
1	3.594722000	0.390781000	1.868377000
1	4.690514000	-0.881423000	1.290935000
6	3.215725000	-1.661600000	-1.614411000
1	3.775875000	-0.945743000	-2.235230000
1	3.887653000	-2.502864000	-1.382398000
1	2.379706000	-2.052917000	-2.214077000
6	0.764286000	2.177808000	1.656225000
1	-0.034199000	2.875087000	1.952650000
1	0.800613000	1.351548000	2.382080000
1	1.721179000	2.721493000	1.722320000
6	0.282491000	2.862666000	-1.373998000
1	0.105806000	2.414064000	-2.362793000
1	-0.552810000	3.535354000	-1.127383000
1	1.195311000	3.476348000	-1.448045000
6	-1.284132000	0.519412000	-0.021137000
1	-1.739760000	1.475200000	0.266794000
1	-0.774776000	-0.081985000	0.742102000
1	-1.057576000	0.330556000	-1.078060000

dicat

14	0.982478000	-0.568930000	0.691940000
14	0.287622000	2.519386000	1.646102000
8	0.166136000	0.997749000	0.584573000
6	0.607164000	-1.391186000	2.281416000
1	1.132195000	-0.876446000	3.103135000
1	0.972333000	-2.430558000	2.272183000
1	-0.470014000	-1.398421000	2.502838000
6	2.691204000	-0.511706000	0.043565000
1	2.760855000	0.059280000	-0.893712000
1	3.062263000	-1.534022000	-0.137520000
1	3.366130000	-0.055144000	0.785178000
6	-0.875260000	2.172983000	3.041959000
1	-1.914361000	2.043659000	2.703773000
1	-0.867861000	3.036040000	3.729367000
1	-0.579554000	1.292690000	3.632087000
6	-0.258540000	3.850187000	0.482578000
1	0.394629000	3.939050000	-0.398624000
1	-0.206843000	4.814105000	1.017387000
1	-1.305061000	3.739812000	0.156774000
6	2.080878000	2.540780000	2.100091000
1	2.385729000	1.673445000	2.706890000
1	2.264835000	3.430538000	2.726437000
1	2.739876000	2.626283000	1.222891000
14	-0.982478000	0.568930000	-0.691940000
14	-0.287622000	-2.519386000	-1.646102000
8	-0.166136000	-0.997749000	-0.584573000
6	-0.607164000	1.391186000	-2.281416000
1	-1.132195000	0.876446000	-3.103135000
1	-0.972333000	2.430558000	-2.272183000
1	0.470014000	1.398421000	-2.502838000
6	-2.691204000	0.511706000	-0.043565000
1	-2.760855000	-0.059280000	0.893712000
1	-3.062263000	1.534022000	0.137520000
1	-3.366130000	0.055144000	-0.785178000
6	0.875260000	-2.172983000	-3.041959000
1	1.914361000	-2.043659000	-2.703773000
1	0.867861000	-3.036040000	-3.729367000
1	0.579554000	-1.292690000	-3.632087000
6	0.258540000	-3.850187000	-0.482578000
1	-0.394629000	-3.939050000	0.398624000
1	0.206843000	-4.814105000	-1.017387000
1	1.305061000	-3.739812000	-0.156774000
6	-2.080878000	-2.540780000	-2.100091000
1	-2.385729000	-1.673445000	-2.706890000
1	-2.264835000	-3.430538000	-2.726437000
1	-2.739876000	-2.626283000	-1.222891000

Toluene adducts

T(+)_toluene_para

6	-0.501788000	-1.222115000	-1.009658000
6	-1.712926000	-1.214370000	-0.345515000
6	-2.334904000	-0.000019000	0.008570000
6	-1.712924000	1.214354000	-0.345464000
6	-0.501787000	1.222130000	-1.009596000
6	0.170973000	0.000012000	-1.294937000
1	-0.043727000	-2.169170000	-1.304754000
1	-2.209590000	-2.158565000	-0.110652000
1	-2.209590000	2.158539000	-0.110560000
1	-0.043715000	2.169196000	-1.304636000
1	0.955918000	0.000031000	-2.063332000
14	1.619299000	-0.000011000	0.293649000
6	0.716852000	-0.000648000	1.914975000
1	0.098353000	-0.897947000	2.056683000
1	1.483813000	-0.000102000	2.708572000
1	0.097100000	0.895784000	2.056700000
6	2.593626000	1.549485000	-0.037893000
1	3.485833000	1.525781000	0.610792000
1	2.950012000	1.611103000	-1.077340000
1	2.032893000	2.463561000	0.204964000
6	2.594432000	-1.548834000	-0.038646000
1	2.951761000	-1.609217000	-1.077841000
1	3.486049000	-1.525454000	0.610866000
1	2.033867000	-2.463390000	0.202773000
6	-3.624328000	-0.000009000	0.753997000
1	-4.220698000	-0.896429000	0.536732000
1	-3.420585000	0.000727000	1.839274000
1	-4.221239000	0.895805000	0.535678000

T(+)_toluene_ortho

6	1.167394000	1.028461000	-0.304617000
6	2.146479000	0.500834000	0.542827000
6	2.421408000	-0.864226000	0.553988000
6	1.732708000	-1.757057000	-0.280497000
6	0.737728000	-1.271244000	-1.105583000
6	0.383551000	0.111015000	-1.070373000
1	2.734395000	1.175910000	1.169366000
1	3.208312000	-1.243702000	1.211222000
1	1.992905000	-2.817038000	-0.288770000
1	0.197961000	-1.941611000	-1.777964000
1	-0.205688000	0.514400000	-1.905615000
14	-1.362526000	-0.115727000	0.203161000
6	-0.811310000	-0.678558000	1.881672000
1	-0.227247000	0.083209000	2.416224000
1	-1.729327000	-0.864380000	2.465848000
1	-0.244438000	-1.619292000	1.856314000
6	-2.394923000	-1.354627000	-0.724575000
1	-3.405903000	-1.337733000	-0.283210000
1	-2.503502000	-1.102367000	-1.790458000
1	-2.010331000	-2.380664000	-0.635036000
6	-2.142448000	1.571863000	0.198217000
1	-2.347126000	1.948369000	-0.814920000
1	-3.116849000	1.458560000	0.704853000
1	-1.567925000	2.322801000	0.756761000
6	0.990015000	2.502720000	-0.438898000
1	1.879177000	2.920129000	-0.940632000
1	0.115260000	2.771624000	-1.043009000
1	0.922085000	2.996885000	0.541801000

T(+)_toluene_meta

6	-0.957576000	-0.598679000	-0.978870000
6	-2.048824000	-0.370953000	-0.143402000
6	-2.186445000	0.917757000	0.405813000
6	-1.284739000	1.948996000	0.135590000
6	-0.197860000	1.715420000	-0.694276000
6	0.007967000	0.415007000	-1.221204000
1	-0.830454000	-1.576590000	-1.450159000
1	-3.040174000	1.120889000	1.059160000
1	-1.447438000	2.940481000	0.562979000
1	0.501373000	2.518096000	-0.938081000
1	0.702945000	0.289441000	-2.062042000
14	1.510946000	-0.272543000	0.202650000
6	0.755764000	-0.332428000	1.894400000
1	-0.017834000	-1.106732000	1.988986000
1	1.571717000	-0.591114000	2.591590000
1	0.346040000	0.634737000	2.217214000
6	2.859838000	0.991676000	0.006853000
1	3.754352000	0.603518000	0.523558000
1	3.138528000	1.155718000	-1.045061000
1	2.602575000	1.956746000	0.466566000
6	1.973766000	-1.932136000	-0.496106000
1	2.229925000	-1.885210000	-1.565332000
1	2.876252000	-2.273293000	0.039554000
1	1.192230000	-2.690497000	-0.345575000
6	-3.050779000	-1.441554000	0.156107000
1	-3.018504000	-1.718996000	1.221807000
1	-4.072729000	-1.092969000	-0.054862000
1	-2.868739000	-2.347252000	-0.436829000

$T_3O(+)$_toluene

8	-1.605449000	0.121436000	-0.004028000
14	-1.142091000	1.828352000	0.426508000
14	-1.537060000	-0.411124000	-1.745266000
14	-1.693290000	-1.140767000	1.306747000
6	-2.358209000	2.955079000	-0.422551000
1	-3.358644000	2.498015000	-0.476811000
1	-2.451978000	3.863090000	0.194649000
1	-2.059273000	3.268373000	-1.429892000
6	0.642109000	1.972981000	-0.068716000
1	0.999125000	2.982857000	0.190476000
1	1.263786000	1.250298000	0.482053000
1	0.831577000	1.823049000	-1.139694000
6	-1.321265000	2.103090000	2.259910000
1	-0.765278000	1.421462000	2.915487000
1	-0.896076000	3.110555000	2.414329000
1	-2.369868000	2.147631000	2.584002000
6	-1.439276000	1.068650000	-2.872355000
1	-1.273553000	0.625565000	-3.870270000
1	-2.382715000	1.628597000	-2.925575000
1	-0.609643000	1.764124000	-2.693950000
6	-3.142660000	-1.280919000	-2.107983000
1	-3.135192000	-2.355902000	-1.892839000
1	-3.980006000	-0.816969000	-1.563553000
1	-3.349300000	-1.157726000	-3.183278000
6	0.024984000	-1.408032000	-1.875961000
1	0.054155000	-2.296772000	-1.232354000
1	0.133865000	-1.751530000	-2.917638000
1	0.906104000	-0.792613000	-1.636707000
6	-2.203417000	-2.784833000	0.595488000
1	-2.107422000	-3.477527000	1.450264000
1	-3.258246000	-2.802627000	0.288970000
1	-1.581528000	-3.191001000	-0.211810000
6	0.032506000	-1.240805000	1.986646000
1	0.404919000	-0.305063000	2.423680000
1	0.052605000	-2.006399000	2.779299000
1	0.745794000	-1.550736000	1.207625000
6	-3.044883000	-0.613663000	2.474918000
1	-2.713122000	0.035574000	3.293776000
1	-3.863768000	-0.112013000	1.935898000
1	-3.466778000	-1.526741000	2.924836000
6	3.545073000	-1.410185000	1.213578000
6	3.436391000	-2.106084000	0.010258000
6	3.571966000	-1.414479000	-1.193992000
6	3.812713000	-0.041572000	-1.191805000
6	3.932313000	0.669651000	0.009984000
6	3.786040000	-0.036262000	1.210895000
1	3.455129000	-1.942074000	2.164104000
1	3.265708000	-3.185320000	0.009857000
1	3.503234000	-1.951497000	-2.143438000
1	3.932656000	0.488117000	-2.141390000
1	3.884930000	0.496831000	2.160977000
6	4.255871000	2.134994000	0.008260000
1	3.906225000	2.631018000	0.925154000
1	5.345857000	2.290383000	-0.048142000
1	3.812940000	2.649371000	-0.857157000

monocat_toluene

6	-1.309058000	-1.268698000	-0.514466000
6	-2.549110000	-1.537521000	0.034864000
6	-3.597277000	-0.601896000	-0.055924000
6	-3.370979000	0.612416000	-0.734945000
6	-2.139363000	0.892094000	-1.296105000
6	-1.059177000	-0.018454000	-1.142022000
1	-0.501221000	-1.999951000	-0.440683000
1	-2.725653000	-2.493082000	0.533922000
1	-4.190580000	1.327113000	-0.839228000
1	-1.987300000	1.821276000	-1.850422000
1	-0.186899000	0.071014000	-1.802523000
14	0.058409000	0.907357000	0.469038000
6	-0.977466000	0.870620000	1.995190000
1	-1.069516000	-0.147246000	2.397262000
1	-0.465729000	1.488561000	2.751891000
1	-1.977487000	1.298917000	1.838551000
6	0.439249000	2.584050000	-0.223085000
1	1.196599000	3.053059000	0.427066000
1	0.859845000	2.533418000	-1.238616000
1	-0.446195000	3.236780000	-0.233060000
6	-4.920525000	-0.884677000	0.569440000
1	-5.140946000	-1.960800000	0.583139000
1	-4.915242000	-0.540987000	1.618576000
1	-5.734995000	-0.355101000	0.057111000
8	1.328866000	-0.090445000	0.538575000
14	2.898971000	-0.479162000	-0.027261000
6	2.724958000	-0.805666000	-1.861736000
1	2.392421000	0.091794000	-2.408515000
1	3.696214000	-1.098810000	-2.291528000
1	2.019854000	-1.626366000	-2.069583000
6	3.991510000	0.994835000	0.305561000
1	4.005544000	1.249090000	1.376811000
1	5.028566000	0.776312000	0.004469000
1	3.666982000	1.882993000	-0.258871000
6	3.411711000	-1.993288000	0.920981000
1	2.722807000	-2.832706000	0.740495000
1	4.420868000	-2.316345000	0.620542000
1	3.430973000	-1.792987000	2.003203000

monocat_toluene iso1

6	-2.321194000	-0.336119000	-1.075748000
6	-3.462027000	0.081519000	-0.395016000
6	-3.373416000	1.272325000	0.351914000
6	-2.200908000	2.023743000	0.423740000
6	-1.063719000	1.599135000	-0.251708000
6	-1.103226000	0.383788000	-0.975798000
1	-2.358955000	-1.236673000	-1.693885000
1	-4.260151000	1.622838000	0.888229000
1	-2.182039000	2.950419000	1.000732000
1	-0.137261000	2.175116000	-0.211443000
1	-0.300429000	0.166871000	-1.691981000
14	-0.050512000	-0.827950000	0.539740000
6	-0.951839000	-0.684263000	2.141171000
1	-2.025923000	-0.896660000	2.046525000
1	-0.522033000	-1.440034000	2.820157000
1	-0.809147000	0.303209000	2.599673000
6	-0.049770000	-2.500305000	-0.255547000
1	0.295278000	-2.473989000	-1.299413000
1	0.650988000	-3.138499000	0.308608000
1	-1.043011000	-2.971022000	-0.212918000
6	-4.746424000	-0.684601000	-0.462331000
1	-5.025423000	-1.070870000	0.530770000
1	-5.570791000	-0.038283000	-0.799297000
1	-4.676368000	-1.535985000	-1.151704000
8	1.379528000	-0.082385000	0.537599000
14	2.919826000	0.237899000	-0.140471000
6	3.254471000	-1.092552000	-1.408424000
1	3.260278000	-2.095651000	-0.954125000
1	2.511185000	-1.083204000	-2.221712000
1	4.242127000	-0.936873000	-1.871224000
6	2.780224000	1.923564000	-0.932230000
1	2.508479000	2.692296000	-0.191761000
1	3.745708000	2.220377000	-1.372054000
1	2.036711000	1.939361000	-1.745201000
6	4.122773000	0.207147000	1.275465000
1	3.859877000	0.959479000	2.034646000
1	4.142262000	-0.779862000	1.762399000
1	5.142341000	0.427147000	0.921481000

Monocot_toluene iso2

6	2.575325000	0.539830000	-0.757514000
6	3.607704000	-0.184326000	-0.155104000
6	3.440558000	-1.528194000	0.174735000
6	2.236521000	-2.197868000	-0.078602000
6	1.183427000	-1.499124000	-0.639524000
6	1.315130000	-0.108766000	-0.918739000
1	4.573452000	0.297587000	0.015659000
1	4.274036000	-2.073978000	0.624678000
1	2.134320000	-3.258605000	0.157016000
1	0.228045000	-1.989415000	-0.836357000
1	0.576504000	0.358991000	-1.583693000
14	0.185115000	0.533990000	0.849128000
6	1.006796000	-0.139446000	2.356700000
1	2.079046000	0.097781000	2.399157000
1	0.525197000	0.342057000	3.224384000
1	0.865586000	-1.224228000	2.450230000
6	0.157979000	2.380695000	0.716431000
1	-0.227427000	2.735405000	-0.249796000
1	-0.535502000	2.735803000	1.497993000
1	1.139074000	2.835798000	0.910991000
6	2.806830000	1.923609000	-1.265170000
1	3.444919000	1.870204000	-2.163195000
1	1.877056000	2.428435000	-1.554459000
1	3.343433000	2.543995000	-0.532512000
8	-1.235665000	-0.161204000	0.523580000
14	-2.765757000	-0.205015000	-0.241486000
6	-3.993855000	-0.646450000	1.082077000
1	-3.748580000	-1.615142000	1.543620000
1	-4.016667000	0.115950000	1.875782000
1	-5.008230000	-0.721475000	0.659410000
6	-3.073473000	1.484952000	-0.976983000
1	-2.321316000	1.747388000	-1.738208000
1	-4.056997000	1.508038000	-1.473105000
1	-3.078294000	2.271062000	-0.205838000
6	-2.630910000	-1.514826000	-1.565790000
1	-3.591123000	-1.624165000	-2.094727000
1	-1.872048000	-1.260610000	-2.323034000
1	-2.382356000	-2.497410000	-1.134726000

Monocat_toluene iso3

6	-1.216130000	-0.907045000	-1.051191000
6	-2.251982000	-1.541473000	-0.391021000
6	-3.397083000	-0.827547000	0.010999000
6	-3.483357000	0.547419000	-0.287885000
6	-2.459047000	1.193784000	-0.952898000
6	-1.269377000	0.492717000	-1.291713000
1	-0.333404000	-1.468296000	-1.364527000
1	-2.192737000	-2.614194000	-0.192941000
1	-4.385052000	1.099024000	-0.011933000
1	-2.553322000	2.251794000	-1.209040000
1	-0.592919000	0.919605000	-2.044260000
14	0.077776000	1.137830000	0.264671000
6	-0.559774000	0.520009000	1.887626000
1	-0.509435000	-0.574670000	1.964700000
1	0.082538000	0.945385000	2.676963000
1	-1.589476000	0.851403000	2.084985000
6	0.089844000	2.974369000	0.047623000
1	0.900913000	3.378869000	0.675924000
1	0.302422000	3.267415000	-0.991042000
1	-0.852487000	3.439225000	0.372734000
6	-4.494236000	-1.511363000	0.752727000
1	-4.565574000	-2.573972000	0.483573000
1	-4.293518000	-1.462865000	1.837257000
1	-5.465470000	-1.028401000	0.579986000
8	1.439692000	0.443604000	-0.258633000
14	2.815024000	-0.547632000	-0.022666000
6	2.353931000	-2.251445000	-0.636837000
1	2.077462000	-2.238266000	-1.703166000
1	3.210516000	-2.936514000	-0.532992000
1	1.521710000	-2.683596000	-0.058756000
6	4.179169000	0.220890000	-1.024940000
1	4.404744000	1.239907000	-0.675019000
1	5.102303000	-0.374368000	-0.943144000
1	3.908434000	0.276563000	-2.090469000
6	3.176711000	-0.552107000	1.808927000
1	2.346186000	-0.981027000	2.391413000
1	4.071994000	-1.158668000	2.019708000
1	3.375425000	0.464788000	2.182528000

Monocat_toluene iso 4

6	1.309212000	-1.268435000	0.515092000
6	2.549137000	-1.537517000	-0.034425000
6	3.597385000	-0.601952000	0.055823000
6	3.371321000	0.612598000	0.734517000
6	2.139857000	0.892531000	1.295868000
6	1.059572000	-0.017996000	1.142326000
1	0.501311000	-1.999652000	0.441680000
1	2.725492000	-2.493252000	-0.533213000
1	4.190999000	1.327269000	0.838391000
1	1.987999000	1.821862000	1.849989000
1	0.187413000	0.071837000	1.802940000
14	-0.058415000	0.907235000	-0.468920000
6	0.977218000	0.870117000	-1.995225000
1	1.069705000	-0.147955000	-2.396673000
1	0.465091000	1.487360000	-2.752227000
1	1.977073000	1.298959000	-1.838999000
6	-0.439343000	2.584054000	0.222810000
1	-1.195389000	3.053634000	-0.428443000
1	-0.861514000	2.533448000	1.237688000
1	0.446463000	3.236267000	0.234279000
6	4.920509000	-0.884980000	-0.569693000
1	5.139918000	-1.961275000	-0.585202000
1	4.915913000	-0.539358000	-1.618196000
1	5.735334000	-0.357071000	-0.056184000
8	-1.328661000	-0.090861000	-0.537861000
14	-2.899120000	-0.479137000	0.027310000
6	-3.991301000	0.994958000	-0.306272000
1	-4.005784000	1.248225000	-1.377754000
1	-5.028277000	0.777030000	-0.004480000
1	-3.666222000	1.883542000	0.257162000
6	-2.726034000	-0.805506000	1.861871000
1	-2.020211000	-1.625477000	2.070137000
1	-2.394927000	0.092237000	2.409038000
1	-3.697316000	-1.099798000	2.290839000
6	-3.411585000	-1.993266000	-0.921105000
1	-2.722990000	-2.832813000	-0.740032000
1	-4.421044000	-2.316121000	-0.621476000
1	-3.429946000	-1.793012000	-2.003356000

Monocat_toluene iso5

6	2.023280000	0.345662000	1.343983000
6	1.872473000	-1.022014000	1.248002000
6	1.907977000	-1.666694000	-0.005461000
6	2.134160000	-0.899472000	-1.162077000
6	2.292973000	0.471035000	-1.081578000
6	2.155238000	1.135579000	0.167302000
1	2.015941000	0.833029000	2.321629000
1	1.742645000	-1.618334000	2.154083000
1	2.199953000	-1.398070000	-2.131300000
1	2.491977000	1.054175000	-1.983664000
1	2.517753000	2.167841000	0.260881000
14	0.086551000	1.732488000	-0.055833000
6	0.132885000	2.848367000	-1.531181000
1	0.276976000	2.283971000	-2.463253000
1	-0.841657000	3.360615000	-1.596139000
1	0.908746000	3.624815000	-1.450955000
6	-0.293456000	2.582092000	1.546563000
1	-1.276346000	3.071951000	1.443486000
1	-0.355273000	1.874106000	2.385234000
1	0.439745000	3.366282000	1.789662000

6	1.741649000	-3.146305000	-0.084957000
1	0.818451000	-3.464215000	0.424014000
1	1.715369000	-3.506281000	-1.120929000
1	2.574580000	-3.648299000	0.434258000
8	-0.710534000	0.353076000	-0.305970000
14	-2.017742000	-0.714834000	-0.052232000
6	-1.815572000	-1.444088000	1.656195000
1	-1.805638000	-0.660480000	2.430068000
1	-2.655258000	-2.119664000	1.884658000
1	-0.887356000	-2.028110000	1.747338000
6	-3.576516000	0.299698000	-0.185177000
1	-3.655597000	0.785961000	-1.169979000
1	-4.466002000	-0.337739000	-0.058554000
1	-3.619509000	1.079491000	0.591713000
6	-1.882217000	-1.999563000	-1.393968000
1	-0.929473000	-2.547065000	-1.335737000
1	-2.697175000	-2.735851000	-1.311709000
1	-1.949148000	-1.535687000	-2.390031000

Toluene_dicat_toluene

14	0.073165000	-0.142927000	1.322198000
14	-1.661377000	-2.499799000	-0.177155000
8	-0.691037000	-0.935479000	-0.063205000
6	1.372115000	-1.204642000	2.038272000
1	0.914929000	-2.084076000	2.520981000
1	1.922863000	-0.657750000	2.819439000
1	2.097342000	-1.545176000	1.282321000
6	-1.129050000	0.731678000	2.382339000
1	-1.940118000	1.186418000	1.792777000
1	-0.616015000	1.518030000	2.960154000
1	-1.572925000	0.034613000	3.110175000
6	-0.366660000	-3.804504000	-0.397732000
1	0.207883000	-3.673841000	-1.327031000
1	-0.857894000	-4.790195000	-0.457249000
1	0.336207000	-3.843677000	0.448139000
6	-2.733080000	-2.186527000	-1.651382000
1	-3.375260000	-1.303531000	-1.503831000
1	-3.395690000	-3.056519000	-1.794225000
1	-2.160642000	-2.079300000	-2.586453000
6	-2.536083000	-2.524128000	1.453147000
1	-1.851485000	-2.632329000	2.309297000
1	-3.208987000	-3.397218000	1.480952000
1	-3.159678000	-1.626702000	1.584163000
14	-0.072959000	0.145224000	-1.320175000
14	1.663402000	2.500978000	0.178903000
8	0.691939000	0.937290000	0.065216000
6	-1.372528000	1.207291000	-2.034558000
1	-0.915791000	2.086404000	-2.518271000
1	-1.924786000	0.660388000	-2.814657000
1	-2.096428000	1.548258000	-1.277543000
6	1.128569000	-0.729040000	-2.381329000
1	1.940168000	-1.183609000	-1.792339000
1	0.615235000	-1.515576000	-2.958628000
1	1.571730000	-0.031968000	-3.109580000
6	0.369473000	3.806469000	0.399439000
1	-0.205273000	3.676061000	1.328648000
1	0.861223000	4.791893000	0.459094000
1	-0.333221000	3.846084000	-0.446556000
6	2.735094000	2.187104000	1.653019000
1	3.375934000	1.303083000	1.505782000
1	3.399081000	3.056173000	1.795076000
1	2.162791000	2.081300000	2.588331000
6	2.537948000	2.524559000	-1.451494000
1	1.853400000	2.634486000	-2.307463000
1	3.212448000	3.396429000	-1.478903000
1	3.159941000	1.626101000	-1.583112000
6	5.330149000	0.022400000	-0.237784000
6	5.316796000	-0.514376000	1.059471000
6	4.805834000	-1.786878000	1.307343000
6	4.286330000	-2.552671000	0.260769000
6	4.285056000	-2.030066000	-1.032404000
6	4.797313000	-0.753205000	-1.275816000
1	5.755780000	0.059013000	1.881397000
1	4.842905000	-2.197361000	2.319720000
1	3.927776000	-3.568362000	0.446408000
1	3.931415000	-2.640854000	-1.867915000
1	4.833943000	-0.372169000	-2.301063000
6	-4.289562000	2.550344000	-0.264728000
6	-4.287008000	2.027983000	1.028534000

6	-4.798245000	0.750834000	1.272575000
6	-5.331285000	-0.025309000	0.235054000
6	-5.319118000	0.511199000	-1.062335000
6	-4.809175000	1.783979000	-1.310816000
1	-3.931848000	3.566251000	-0.450797000
1	-3.933071000	2.639192000	1.863617000
1	-4.833921000	0.369976000	2.297923000
1	-5.758134000	-0.062728000	-1.883866000
1	-4.846996000	2.194169000	-2.323277000
6	5.967979000	1.353687000	-0.507546000
1	5.668946000	2.112482000	0.232094000
1	7.065023000	1.271775000	-0.445046000
1	5.730306000	1.728836000	-1.512685000
6	-5.968119000	-1.356933000	0.505481000
1	-5.669439000	-2.115566000	-0.234461000
1	-5.729288000	-1.731954000	1.510398000
1	-7.065273000	-1.275589000	0.444088000

CB(-)_toluene

6	0.836700000	-0.205821000	0.016219000
1	1.917269000	-0.353257000	0.029415000
5	-2.358658000	0.240999000	-0.019295000
5	-1.360353000	0.510070000	-1.482203000
5	-1.599686000	-1.153947000	-0.848798000
5	-1.608166000	-1.068884000	0.945306000
5	-1.373847000	0.647145000	1.420742000
5	-1.220534000	1.623684000	-0.079370000
5	0.236359000	1.070334000	-0.936941000
5	0.005472000	-0.629307000	-1.408086000
5	-0.146120000	-1.596125000	0.078232000
5	-0.007532000	-0.492408000	1.467188000
5	0.228193000	1.154351000	0.839633000
17	-4.123033000	0.488170000	-0.039045000
17	-2.042375000	1.036777000	-3.041868000
17	-2.536112000	-2.379408000	-1.741198000
17	-2.552944000	-2.205523000	1.940549000
17	-2.070168000	1.318476000	2.917323000
17	-1.755846000	3.321365000	-0.162446000
17	1.332473000	2.108130000	-1.878387000
17	0.867802000	-1.285886000	-2.816548000
17	0.562050000	-3.223813000	0.160395000
17	0.845497000	-1.011502000	2.937663000
17	1.315011000	2.276936000	1.689996000
6	4.385154000	0.755341000	0.009223000
6	4.252035000	0.051087000	1.212718000
1	4.275703000	0.598420000	2.158406000
6	4.049838000	-1.328607000	1.220353000
1	3.912690000	-1.850350000	2.169542000
6	3.977321000	-2.033547000	0.020123000
1	3.785826000	-3.107996000	0.023541000
6	4.102708000	-1.345405000	-1.186059000
1	4.009948000	-1.881114000	-2.132852000
6	4.304291000	0.033386000	-1.189001000
1	4.371636000	0.567238000	-2.140331000
6	4.543490000	2.246431000	-0.002940000
1	5.174347000	2.576377000	-0.841758000
1	4.988461000	2.611825000	0.933824000
1	3.559234000	2.728848000	-0.115035000

[CHB11Cl11]–

6	-0.000385000	0.000561000	-1.697241000
1	-0.000713000	0.001145000	-2.787828000
5	0.000275000	-0.000436000	1.527036000
5	-1.363903000	-0.690014000	0.592124000
5	0.234752000	-1.510117000	0.591194000
5	1.508897000	-0.243845000	0.591395000
5	0.698010000	1.359322000	0.592077000
5	-1.077474000	1.083644000	0.592282000
5	-1.496074000	0.242162000	-0.918031000
5	-0.692765000	-1.347623000	-0.918245000
5	1.067747000	-1.074809000	-0.918578000
5	1.352149000	0.683811000	-0.917954000
5	-0.232449000	1.497635000	-0.917428000
17	0.000610000	-0.000828000	3.308347000
17	-2.798492000	-1.415854000	1.360107000
17	0.481615000	-3.099455000	1.358141000
17	3.096318000	-0.500755000	1.358724000
17	1.432837000	2.789497000	1.359521000
17	-2.210718000	2.224235000	1.360006000
17	-2.981540000	0.483301000	-1.861915000
17	-1.380863000	-2.685466000	-1.862815000
17	2.127810000	-2.142326000	-1.863150000
17	2.695207000	1.362603000	-1.861989000
17	-0.462361000	2.984860000	-1.861335000

T(+)_CB

6	-2.332540000	-0.000057000	0.628542000
1	-3.353966000	-0.000098000	1.013831000
5	0.619182000	0.000043000	-0.480245000
5	-0.640695000	0.905899000	-1.335592000
5	-0.031566000	1.462972000	0.270424000
5	0.336049000	-0.000188000	1.261423000
5	-0.031603000	-1.463066000	0.270014000
5	-0.640720000	-0.905518000	-1.335851000
5	-2.148353000	0.000176000	-1.068034000
5	-1.778249000	1.449696000	-0.082605000
5	-1.178764000	0.896404000	1.506755000
5	-1.178788000	-0.896799000	1.506506000
5	-1.778290000	-1.449618000	-0.083012000
17	2.260541000	0.000260000	-1.359096000
17	-0.310130000	1.826481000	-2.815291000
17	0.909763000	2.961732000	0.441857000
17	1.598941000	-0.000356000	2.522972000
17	0.909591000	-2.961955000	0.441041000
17	-0.310201000	-1.825703000	-2.815806000
17	-3.545450000	0.000344000	-2.145064000
17	-2.806954000	2.879485000	-0.186774000
17	-1.604444000	1.773252000	2.978051000
17	-1.604504000	-1.774047000	2.977553000
17	-2.807033000	-2.879355000	-0.187570000
14	4.211127000	-0.000020000	-0.192864000
6	4.240406000	1.593856000	0.740792000
1	3.468021000	1.628027000	1.520305000
1	5.231330000	1.690457000	1.216422000
1	4.094394000	2.445262000	0.060711000
6	5.300992000	0.000037000	-1.699355000
1	5.146301000	0.895786000	-2.318958000
1	6.351671000	-0.000099000	-1.363188000
1	5.146130000	-0.895555000	-2.319142000
6	4.240194000	-1.594063000	0.740511000
1	4.093915000	-2.445324000	0.060308000
1	5.231163000	-1.690942000	1.215991000
1	3.467902000	-1.628211000	1.520119000

T(+)_CB iso1

6	1.775136000	0.000019000	1.433888000
1	2.435232000	0.000031000	2.303246000
5	-0.157950000	-0.000008000	-1.147650000
5	1.385288000	-0.903404000	-1.143758000
5	0.021906000	-1.461480000	-0.113227000
5	-0.762337000	-0.000031000	0.512395000
5	0.021847000	1.461456000	-0.113221000
5	1.385255000	0.903439000	-1.143755000
5	2.518966000	0.000035000	-0.105526000
5	1.686199000	-1.449902000	0.526958000
5	0.340127000	-0.899870000	1.551127000
5	0.340091000	0.899851000	1.551132000
5	1.686142000	1.449936000	0.526965000
17	-1.205446000	-0.000047000	-2.593984000
17	1.930252000	-1.840780000	-2.543642000
17	-0.885282000	-2.961518000	-0.415624000
17	-2.524947000	-0.000051000	1.101419000
17	-0.885396000	2.961463000	-0.415605000
17	1.930187000	1.840845000	-2.543631000
17	4.275597000	0.000071000	-0.264260000
17	2.606729000	-2.880029000	0.997051000
17	-0.129491000	-1.764844000	3.019760000
17	-0.129556000	1.764796000	3.019772000
17	2.606618000	2.880097000	0.997062000
14	-4.243532000	-0.000015000	-0.396292000
6	-4.111182000	-1.596040000	-1.315729000
1	-4.074388000	-2.444544000	-0.617688000
1	-5.012001000	-1.697865000	-1.944882000
1	-3.222700000	-1.628605000	-1.959700000
6	-4.111300000	1.596092000	-1.315608000
1	-3.223115000	1.628585000	-1.959989000
1	-5.012413000	1.698153000	-1.944302000
1	-4.074021000	2.444503000	-0.617481000
6	-5.575698000	-0.000073000	0.901594000
1	-5.530737000	0.895881000	1.538285000
1	-6.552540000	-0.000021000	0.388914000
1	-5.530774000	-0.896098000	1.538187000

T(+)_iso2

6	0.388796000	-0.001303000	-1.638496000
1	0.137174000	-0.002706000	-2.700681000
5	1.129071000	-0.009743000	1.507605000
5	2.376268000	0.269531000	0.249471000
5	1.108318000	1.514990000	0.547064000
5	-0.423013000	0.657301000	0.914408000
5	-0.113068000	-1.118091000	0.840823000
5	1.621762000	-1.358669000	0.429217000
5	1.898884000	-0.664011000	-1.193869000
5	1.585615000	1.098935000	-1.119851000
5	-0.134970000	1.340769000	-0.712580000
5	-0.829805000	-0.268405000	-0.521145000
5	0.377395000	-1.519589000	-0.829606000
17	1.507670000	-0.004538000	3.237047000
17	4.078148000	0.559910000	0.635956000
17	1.465976000	3.099934000	1.252036000
17	-1.631791000	1.365411000	2.021645000
17	-1.062049000	-2.266794000	1.813357000
17	2.524086000	-2.770588000	1.000440000
17	2.983918000	-1.313446000	-2.424382000
17	2.359963000	2.186189000	-2.274424000
17	-1.089588000	2.632179000	-1.467147000
17	-2.493314000	-0.658397000	-1.243223000
17	-0.089203000	-2.986673000	-1.703173000
14	-4.394725000	0.022920000	-0.141091000
6	-4.367837000	1.867926000	-0.185429000
1	-4.274810000	2.234233000	-1.217564000
1	-5.327402000	2.224445000	0.227320000
1	-3.549019000	2.281867000	0.417631000
6	-4.351047000	-0.852800000	1.481747000
1	-3.527180000	-0.500712000	2.116474000
1	-5.307171000	-0.652931000	1.995476000
1	-4.250491000	-1.938098000	1.338831000
6	-5.555414000	-0.738500000	-1.377126000
1	-5.428309000	-1.828943000	-1.446769000
1	-6.586638000	-0.542258000	-1.037052000
1	-5.444124000	-0.297000000	-2.378503000

T_3O_CB

6	3.866720000	0.040086000	0.041603000
1	4.957840000	0.064927000	0.042862000
5	0.692754000	-0.035887000	0.039315000
5	1.608229000	-0.978448000	1.228033000
5	1.567282000	0.819137000	1.322009000
5	1.554998000	1.461953000	-0.356427000
5	1.588735000	0.066277000	-1.485790000
5	1.623173000	-1.441328000	-0.510128000
5	3.128505000	-1.449234000	0.435999000
5	3.093915000	-0.058051000	1.562115000
5	3.061303000	1.442841000	0.586356000
5	3.073595000	0.980075000	-1.142925000
5	3.115305000	-0.807208000	-1.236203000
17	-1.091917000	-0.067935000	0.069884000
17	0.791326000	-1.989772000	2.448251000
17	0.711885000	1.667132000	2.636266000
17	0.684014000	2.963626000	-0.769501000
17	0.755474000	0.130832000	-3.063634000
17	0.838820000	-2.934339000	-1.095575000
17	4.095770000	-2.878070000	0.824361000
17	4.027385000	-0.113113000	3.063455000
17	3.959167000	2.869079000	1.123159000
17	3.983588000	1.946014000	-2.312468000
17	4.067210000	-1.603671000	-2.496544000
8	-4.027528000	-0.003344000	-0.108935000
14	-3.815966000	-1.571884000	-1.026671000
14	-3.838040000	1.665984000	-0.843463000
6	-2.771703000	1.733624000	-2.360957000
1	-3.245662000	1.323326000	-3.260753000
1	-2.642619000	2.818602000	-2.522888000
1	-1.766108000	1.312873000	-2.237076000
6	-5.585402000	2.100314000	-1.344246000
1	-5.556787000	3.056318000	-1.891807000
1	-5.985795000	1.342376000	-2.035783000
1	-6.287192000	2.212205000	-0.508683000
6	-3.085225000	2.786184000	0.439856000
1	-3.549418000	2.793450000	1.432443000
1	-2.011536000	2.570166000	0.545998000
1	-3.174828000	3.805965000	0.028510000
6	-2.911725000	-1.367028000	-2.632648000
1	-3.459581000	-0.798728000	-3.394460000
1	-1.888501000	-0.982566000	-2.530646000
1	-2.828234000	-2.407056000	-2.996803000
6	-5.575077000	-2.063185000	-1.425995000
1	-6.086636000	-1.263035000	-1.983081000
1	-5.540004000	-2.947872000	-2.082543000
1	-6.184176000	-2.322656000	-0.551443000
6	-2.898497000	-2.775961000	0.067984000
1	-2.710506000	-2.427163000	1.091020000
1	-3.448715000	-3.728818000	0.121053000
1	-1.906194000	-2.960397000	-0.371700000
14	-4.522289000	-0.099827000	1.637187000
6	-5.811694000	1.217601000	1.951035000
1	-6.726721000	1.022224000	1.372436000
1	-6.075241000	1.121201000	3.017871000
1	-5.501250000	2.255750000	1.784942000
6	-3.016935000	0.068594000	2.707987000
1	-2.298895000	-0.752958000	2.573615000
1	-2.457100000	0.998834000	2.545981000
1	-3.364268000	0.054086000	3.754865000

6	-5.416650000	-1.708317000	1.962730000
1	-5.582978000	-1.708322000	3.053844000
1	-6.406665000	-1.735751000	1.486573000
1	-4.871998000	-2.628467000	1.722427000

$T_3O(+)$_CB iso1

6	-0.563230000	-0.123195000	-0.338800000
1	0.494868000	-0.192412000	-0.579977000
5	-3.695295000	0.092379000	0.389995000
5	-2.513659000	1.310683000	0.972187000
5	-2.927780000	1.215525000	-0.780931000
5	-3.146013000	-0.517159000	-1.207814000
5	-2.868460000	-1.496116000	0.279418000
5	-2.474686000	-0.366739000	1.626277000
5	-0.966294000	0.466838000	1.197614000
5	-1.245672000	1.434356000	-0.264535000
5	-1.620533000	0.316339000	-1.594725000
5	-1.592694000	-1.341751000	-0.950617000
5	-1.182140000	-1.245724000	0.777344000
17	-5.417942000	0.212703000	0.794185000
17	-2.951247000	2.707309000	1.977618000
17	-3.804118000	2.516991000	-1.610712000
17	-4.258492000	-1.040701000	-2.487387000
17	-3.684767000	-3.046692000	0.560849000
17	-2.872995000	-0.729455000	3.318407000
17	0.328162000	0.940692000	2.340457000
17	-0.264064000	2.882840000	-0.593762000
17	-0.943651000	0.635643000	-3.209411000
17	-0.952940000	-2.668072000	-1.943238000
17	-0.112099000	-2.483124000	1.485021000
8	4.075646000	0.031162000	-0.036995000
14	4.448866000	-0.304130000	1.713437000
14	3.876804000	1.720749000	-0.714118000
6	3.008910000	2.858189000	0.462445000
1	3.662597000	3.295017000	1.225485000
1	2.641266000	3.679797000	-0.174913000
1	2.116960000	2.420553000	0.933407000
6	5.638691000	2.266163000	-0.989568000
1	5.633726000	3.313846000	-1.330892000
1	6.208305000	2.233258000	-0.047434000
1	6.173348000	1.668976000	-1.739777000
6	2.836659000	1.615391000	-2.253709000
1	3.118621000	0.887385000	-3.021736000
1	1.770663000	1.488397000	-2.010963000
1	2.919617000	2.616548000	-2.711508000
6	4.315739000	1.233910000	2.747157000
1	5.014602000	2.035410000	2.471075000
1	3.296452000	1.632350000	2.819965000
1	4.604561000	0.887029000	3.755059000
6	6.242344000	-0.836289000	1.745089000
1	6.837049000	-0.290072000	0.996086000
1	6.650722000	-0.577146000	2.734945000
1	6.396258000	-1.911962000	1.596344000
6	3.190614000	-1.529909000	2.297072000
1	3.100830000	-2.449160000	1.706276000
1	3.457853000	-1.819692000	3.326923000
1	2.195596000	-1.059522000	2.335117000
14	4.137249000	-1.348071000	-1.226852000
6	5.273532000	-0.832199000	-2.616450000
1	6.294806000	-0.673097000	-2.238188000
1	5.315350000	-1.687744000	-3.311249000
1	4.970816000	0.046210000	-3.197867000
6	2.390014000	-1.746923000	-1.679503000
1	1.814539000	-2.050347000	-0.790760000
1	1.840483000	-0.954563000	-2.203032000
1	2.402941000	-2.620482000	-2.351494000

6	4.948676000	-2.849074000	-0.469138000
1	4.781844000	-3.641720000	-1.219152000
1	6.034645000	-2.733623000	-0.356375000
1	4.519695000	-3.210046000	0.472974000

Monocat_cb

1	-3.722508000	1.222323000	1.064900000
5	-0.151969000	-0.474515000	-0.499302000
5	-1.093118000	-1.360589000	0.708459000
5	-1.725641000	-1.147606000	-0.966107000
5	-1.209312000	0.481638000	-1.554687000
5	-0.262102000	1.273337000	-0.241978000
5	-0.181363000	0.134800000	1.155278000
5	-1.842563000	-0.121580000	1.739009000
5	-2.791314000	-0.902689000	0.438650000
5	-2.862903000	0.220995000	-0.950933000
5	-1.966111000	1.703364000	-0.504095000
5	-1.334233000	1.491827000	1.155753000
17	1.327188000	-1.206643000	-1.349865000
17	-0.657553000	-2.967824000	1.332557000
17	-1.882487000	-2.525390000	-2.072587000
17	-0.829975000	0.772130000	-3.263452000
17	1.053147000	2.411285000	-0.616106000
17	1.220330000	0.101637000	2.254063000
17	-2.298320000	-0.344111000	3.429761000
17	-4.187520000	-1.901530000	0.847209000
17	-4.334908000	0.337875000	-1.916558000
17	-2.544134000	3.285198000	-1.031513000
17	-1.282402000	2.859143000	2.271187000
14	3.237596000	-1.809670000	-0.200801000
8	3.904882000	-0.444729000	0.311959000
6	4.041645000	-2.541119000	-1.700530000
1	4.154529000	-1.796581000	-2.502188000
1	5.047332000	-2.890865000	-1.414403000
1	3.476325000	-3.401535000	-2.088161000
6	2.749065000	-2.977852000	1.126651000
1	2.143643000	-3.805280000	0.732484000
1	3.681060000	-3.385108000	1.553666000
1	2.185236000	-2.467928000	1.918386000
14	4.782131000	0.997254000	0.056989000
6	4.195967000	2.215520000	1.332291000
1	4.398064000	1.839082000	2.346753000
1	4.716033000	3.179399000	1.214783000
1	3.114191000	2.388969000	1.240837000
6	4.470296000	1.530285000	-1.706379000
1	4.950430000	2.501107000	-1.907865000
1	4.886358000	0.802105000	-2.420846000
1	3.393475000	1.641802000	-1.903303000
6	6.577834000	0.536990000	0.310635000
1	6.907443000	-0.221035000	-0.417318000
1	7.224805000	1.420391000	0.188408000
1	6.744087000	0.137871000	1.323314000

Monocat_cb iso1

6	-0.299068000	0.473122000	-0.235054000
1	-1.335189000	0.757827000	-0.402607000
5	2.763437000	-0.436289000	0.240631000
5	1.589583000	-1.511959000	-0.588531000
5	2.154185000	-0.009459000	-1.399779000
5	2.331549000	1.267130000	-0.134097000
5	1.877993000	0.553159000	1.458272000
5	1.422734000	-1.163953000	1.180768000
5	0.027959000	-1.120993000	0.112537000
5	0.435831000	-0.463606000	-1.463041000
5	0.892100000	1.234113000	-1.184801000
5	0.717817000	1.573718000	0.564482000
5	0.165945000	0.090153000	1.372659000
17	4.440125000	-0.941052000	0.501332000
17	1.947913000	-3.147500000	-1.176148000
17	3.166616000	-0.056533000	-2.852050000
17	3.534353000	2.558742000	-0.270253000
17	2.600645000	1.092920000	2.982870000
17	1.607409000	-2.445275000	2.394349000
17	-1.278917000	-2.422415000	0.258985000
17	-0.523817000	-0.936676000	-2.883080000
17	0.435189000	2.463670000	-2.371209000
17	0.068718000	3.134356000	1.099913000
17	-1.033398000	0.130159000	2.684836000
8	-3.299747000	-0.049924000	-0.191891000
14	-4.114870000	1.463104000	-0.097080000
14	-3.419959000	-1.649566000	-0.021472000
6	-5.849790000	1.120863000	-0.707968000
1	-6.455766000	2.040566000	-0.689664000
1	-5.840463000	0.749901000	-1.745034000
1	-6.364159000	0.378570000	-0.076185000
6	-3.182614000	2.617226000	-1.218588000
1	-3.781639000	3.523528000	-1.402000000
1	-2.227114000	2.941711000	-0.778363000
1	-2.967733000	2.149557000	-2.191651000
6	-4.082058000	2.005895000	1.682723000
1	-4.609137000	2.966766000	1.795817000
1	-4.568487000	1.273243000	2.345107000
1	-3.044651000	2.143338000	2.023246000
6	-4.142487000	-2.207011000	1.580576000
1	-5.210349000	-1.935538000	1.612234000
1	-4.059029000	-3.295962000	1.711991000
1	-3.625494000	-1.703180000	2.410767000
6	-3.855965000	-2.544104000	-1.570825000
1	-3.183731000	-2.219952000	-2.379129000
1	-3.774443000	-3.634365000	-1.450485000
1	-4.893014000	-2.298900000	-1.852142000

Monocat_cb iso2

6	-1.790414000	1.059736000	1.389544000
1	-2.037704000	1.694570000	2.242236000
5	-1.069900000	-0.813774000	-1.141895000
5	-1.571088000	-1.517598000	0.433784000
5	-2.757178000	-0.619768000	-0.576111000
5	-1.894015000	0.760332000	-1.352613000
5	-0.177470000	0.718669000	-0.819091000
5	-0.020943000	-0.660122000	0.276311000
5	-1.005832000	-0.412358000	1.720451000
5	-2.705392000	-0.373187000	1.191181000
5	-2.904381000	1.025768000	0.093711000
5	-1.325005000	1.847215000	-0.055362000
5	-0.156021000	0.962398000	0.948258000
17	-0.667206000	-1.862748000	-2.520618000
17	-1.638441000	-3.269335000	0.709255000
17	-4.117469000	-1.438379000	-1.359138000
17	-2.346420000	1.387218000	-2.946032000
17	1.159821000	1.305183000	-1.839924000
17	1.509304000	-1.669322000	0.552778000
17	-0.544923000	-0.902192000	3.356110000
17	-3.979627000	-0.801414000	2.334314000
17	-4.383898000	1.984836000	0.149549000
17	-1.223317000	3.607373000	-0.151099000
17	1.095295000	1.831523000	1.856136000
14	3.336296000	-1.542772000	-0.860147000
8	3.984008000	-0.097028000	-0.607450000
6	2.745648000	-1.854823000	-2.567256000
1	2.123260000	-1.028569000	-2.934109000
1	3.639970000	-1.945458000	-3.206515000
1	2.168421000	-2.787850000	-2.622623000
6	4.220429000	-2.934925000	-0.016650000
1	3.665567000	-3.881124000	-0.100349000
1	5.199535000	-3.066625000	-0.506318000
1	4.398001000	-2.716803000	1.046648000
14	4.832260000	1.009231000	0.378678000
6	4.524747000	0.515687000	2.155001000
1	4.971247000	-0.466032000	2.380089000
1	4.978190000	1.248278000	2.841759000
1	3.448093000	0.471882000	2.377289000
6	4.199278000	2.702726000	-0.053915000
1	4.705853000	3.472098000	0.550098000
1	4.387665000	2.923128000	-1.115859000
1	3.116685000	2.778396000	0.122659000
6	6.635577000	0.801366000	-0.070617000
1	6.803941000	1.003770000	-1.139789000
1	7.261146000	1.501716000	0.505714000
1	6.989810000	-0.218671000	0.146833000

Monocat_cb iso3

6	-0.337471000	0.235886000	1.148742000
1	0.373826000	0.438851000	1.946285000
5	-2.508233000	-0.389739000	-1.167125000
5	-1.240798000	0.857264000	-1.392566000
5	-0.795099000	-0.895935000	-1.328711000
5	-1.850484000	-1.696822000	-0.115835000
5	-2.945250000	-0.439340000	0.575037000
5	-2.566299000	1.138199000	-0.214258000
5	-0.891454000	1.560772000	0.210527000
5	0.138113000	0.305771000	-0.451362000
5	-0.185203000	-1.246762000	0.313185000
5	-1.502274000	-0.970223000	1.477007000
5	-1.935524000	0.767693000	1.415736000
17	-3.688325000	-0.733396000	-2.440450000
17	-1.006839000	1.817224000	-2.866389000
17	-0.091619000	-1.724254000	-2.733047000
17	-2.326514000	-3.395445000	-0.269436000
17	-4.573995000	-0.828626000	1.147254000
17	-3.787250000	2.396093000	-0.463577000
17	-0.223026000	3.185194000	0.507606000
17	1.841114000	0.649203000	-1.068949000
17	1.176707000	-2.323462000	0.698938000
17	-1.477857000	-1.815869000	3.027725000
17	-2.335547000	1.628115000	2.906434000
14	3.374046000	1.644743000	0.335123000
8	4.626371000	0.695129000	-0.018771000
6	2.730996000	1.481714000	2.054726000
1	2.540114000	0.431332000	2.317244000
1	3.520887000	1.855244000	2.727962000
1	1.831588000	2.094236000	2.211828000
6	3.472461000	3.318862000	-0.417345000
1	2.480268000	3.793154000	-0.415481000
1	4.167173000	3.943544000	0.166762000
1	3.844429000	3.256440000	-1.450094000
14	5.298791000	-0.882158000	0.016731000
6	7.130935000	-0.625262000	-0.206358000
1	7.549555000	-0.013386000	0.607426000
1	7.660470000	-1.591052000	-0.210966000
1	7.343256000	-0.118617000	-1.160287000
6	4.531267000	-1.837400000	-1.386944000
1	4.965998000	-2.848602000	-1.437279000
1	3.443884000	-1.948073000	-1.257880000
1	4.717803000	-1.344307000	-2.353494000
6	4.889083000	-1.623385000	1.682014000
1	3.804702000	-1.776144000	1.795958000
1	5.370353000	-2.608851000	1.787239000
1	5.247459000	-0.989119000	2.507922000

Monocat_cb iso4

6	0.713847000	0.838356000	1.264202000
1	0.178644000	1.348621000	2.066890000
5	2.292580000	-0.665455000	-1.121999000
5	2.321873000	1.123713000	-0.964055000
5	3.162677000	0.090444000	0.252285000
5	2.165019000	-1.385176000	0.526509000
5	0.715668000	-1.261888000	-0.521538000
5	0.806436000	0.284554000	-1.443066000
5	0.767083000	1.625611000	-0.262519000
5	2.209169000	1.503694000	0.778638000
5	2.114694000	-0.035889000	1.692046000
5	0.610191000	-0.869495000	1.217932000
5	-0.168178000	0.155659000	0.013775000
17	3.137252000	-1.505832000	-2.431750000
17	3.195910000	2.176589000	-2.087938000
17	4.926073000	0.064439000	0.398781000
17	2.868483000	-2.955684000	0.946674000
17	-0.074484000	-2.717688000	-1.190817000
17	0.044755000	0.455427000	-3.042005000
17	-0.084157000	3.161952000	-0.493359000
17	2.836311000	2.954983000	1.562818000
17	2.647678000	-0.103335000	3.373601000
17	-0.389022000	-1.733633000	2.404151000
17	-1.990094000	0.423809000	0.067822000
14	-3.470416000	-1.191771000	-0.767648000
8	-4.768809000	-0.273621000	-0.480684000
6	-3.281045000	-2.617435000	0.377494000
1	-3.483584000	-2.308813000	1.412444000
1	-4.017981000	-3.384861000	0.086787000
1	-2.273607000	-3.050761000	0.318992000
6	-3.069359000	-1.314994000	-2.552829000
1	-2.047923000	-1.684776000	-2.714596000
1	-3.784066000	-2.016687000	-3.014380000
1	-3.179832000	-0.332071000	-3.031534000
14	-5.592519000	0.795570000	0.563041000
6	-7.407237000	0.506360000	0.257151000
1	-7.689045000	-0.530737000	0.495498000
1	-8.019076000	1.176141000	0.881804000
1	-7.662528000	0.698264000	-0.796270000
6	-5.057459000	2.519790000	0.090946000
1	-5.563936000	3.268205000	0.721006000
1	-3.971901000	2.660891000	0.208762000
1	-5.314585000	2.733435000	-0.958116000
6	-5.092936000	0.360420000	2.312360000
1	-4.004675000	0.445623000	2.459090000
1	-5.577500000	1.038834000	3.032480000
1	-5.395737000	-0.666822000	2.569501000

Monocat_cb iso5

6	1.791224000	1.060281000	1.389215000
1	2.039021000	1.695357000	2.241588000
5	1.069814000	-0.814037000	-1.141419000
5	1.895188000	0.759318000	-1.352863000
5	2.757094000	-0.621109000	-0.575382000
5	1.570131000	-1.517427000	0.434783000
5	0.020770000	-0.658852000	0.276513000
5	0.178494000	0.719288000	-0.819572000
5	1.326814000	1.847318000	-0.056271000
5	2.905442000	1.024614000	0.093599000
5	2.705232000	-0.373559000	1.191779000
5	1.005507000	-0.411009000	1.720734000
5	0.156805000	0.964023000	0.947604000
17	0.666953000	-1.863641000	-2.519636000
17	2.348609000	1.384837000	-2.946537000
17	4.116728000	-1.441319000	-1.357932000
17	1.635966000	-3.269112000	0.711115000
17	-1.510242000	-1.666634000	0.553628000
17	-1.158514000	1.306209000	-1.840601000
17	1.226851000	3.607540000	-0.153025000
17	4.385754000	1.982522000	0.149229000
17	3.978815000	-0.802146000	2.335496000
17	0.543787000	-0.899560000	3.356576000
17	-1.094505000	1.834506000	1.854184000
14	-3.336350000	-1.541819000	-0.860726000
8	-3.984766000	-0.096399000	-0.607774000
6	-4.219736000	-2.934649000	-0.017502000
1	-4.400962000	-2.715007000	1.044873000
1	-5.197064000	-3.069533000	-0.509863000
1	-3.662478000	-3.879742000	-0.097879000
6	-2.745058000	-1.853382000	-2.567709000
1	-2.169147000	-2.787235000	-2.623345000
1	-3.639111000	-1.942175000	-3.207587000
1	-2.121231000	-1.027788000	-2.933625000
14	-4.834361000	1.008523000	0.378608000
6	-6.637415000	0.796716000	-0.069931000
1	-6.988470000	-0.224615000	0.146540000
1	-7.264839000	1.494473000	0.507420000
1	-6.806841000	0.999660000	-1.138799000
6	-4.204228000	2.702985000	-0.054146000
1	-4.710287000	3.471220000	0.551708000
1	-3.121336000	2.779624000	0.120245000
1	-4.395231000	2.924256000	-1.115405000
6	-4.524576000	0.515832000	2.154724000
1	-3.447594000	0.474404000	2.375875000
1	-4.978932000	1.247366000	2.841888000
1	-4.968580000	-0.466881000	2.380343000

Monocat_cb iso6

6	1.790960000	1.059798000	1.389570000
1	2.038508000	1.694579000	2.242236000
5	1.069837000	-0.813669000	-1.141793000
5	1.894982000	0.759907000	-1.352566000
5	2.757089000	-0.620658000	-0.575666000
5	1.570238000	-1.517577000	0.434101000
5	0.020698000	-0.659164000	0.276192000
5	0.178284000	0.719423000	-0.819348000
5	1.326440000	1.847358000	-0.055573000
5	2.905244000	1.024869000	0.093960000
5	2.705084000	-0.373791000	1.191606000
5	1.005393000	-0.411756000	1.720502000
5	0.156599000	0.963441000	0.947886000
17	0.667023000	-1.862597000	-2.520523000
17	2.348204000	1.386194000	-2.946007000
17	4.116986000	-1.440386000	-1.358259000
17	1.636370000	-3.269324000	0.709938000
17	-1.510023000	-1.667534000	0.552855000
17	-1.158578000	1.306615000	-1.840384000
17	1.226083000	3.607591000	-0.151622000
17	4.385489000	1.982799000	0.149968000
17	3.978789000	-0.802759000	2.335055000
17	0.543789000	-0.901074000	3.356144000
17	-1.094747000	1.833351000	1.855041000
14	-3.336272000	-1.541623000	-0.860903000
8	-3.984179000	-0.095880000	-0.608269000
6	-4.220366000	-2.933989000	-0.017642000
1	-4.399557000	-2.715218000	1.045261000
1	-5.198678000	-3.066982000	-0.508545000
1	-3.664533000	-3.879764000	-0.099856000
6	-2.745040000	-1.853550000	-2.567852000
1	-2.167562000	-2.786467000	-2.622951000
1	-3.639066000	-1.944340000	-3.207482000
1	-2.122616000	-1.027201000	-2.934464000
14	-4.833692000	1.008520000	0.378790000
6	-4.524313000	0.514711000	2.154686000
1	-3.447396000	0.471664000	2.375828000
1	-4.977509000	1.246755000	2.842083000
1	-4.969869000	-0.467361000	2.380019000
6	-6.636816000	0.797469000	-0.069871000
1	-7.263706000	1.496075000	0.507049000
1	-6.806039000	0.999979000	-1.138853000
1	-6.988730000	-0.223438000	0.147180000
6	-4.203503000	2.703171000	-0.053147000
1	-4.393413000	2.924208000	-1.114654000
1	-4.710570000	3.471273000	0.552032000
1	-3.120836000	2.780247000	0.122388000

System T-O-T/T(+)/CB

Monocat_cb_sime4

6	1.200548000	2.272543000	0.405110000
1	1.815999000	3.153004000	0.599520000
5	-0.585395000	-0.276018000	-0.150881000
5	1.182462000	-0.416195000	-0.164811000
5	0.295986000	-0.132786000	1.377659000
5	-1.121514000	0.931085000	1.017331000
5	-1.096840000	1.310917000	-0.746435000
5	0.322112000	0.474679000	-1.479888000
5	1.791637000	1.153282000	-0.741633000
5	1.772677000	0.785617000	1.006981000
5	0.360907000	1.607881000	1.734459000
5	-0.491250000	2.491787000	0.432904000
5	0.395373000	2.211860000	-1.096298000
17	-1.522552000	-1.803471000	-0.634282000
17	2.006360000	-1.955074000	-0.479604000
17	0.279109000	-1.354561000	2.668634000
17	-2.647572000	0.822446000	1.928961000
17	-2.586290000	1.581137000	-1.679941000
17	0.265650000	-0.151782000	-3.138171000
17	3.325193000	1.407991000	-1.578166000
17	3.282938000	0.670111000	1.913358000
17	0.476153000	2.310317000	3.349200000
17	-1.223044000	4.062973000	0.764178000
17	0.545023000	3.511496000	-2.279807000
14	-3.326879000	-2.591245000	0.577765000
8	-4.511514000	-1.534612000	0.348274000
6	-2.788882000	-2.787609000	2.319887000
1	-2.614125000	-1.814889000	2.797419000
1	-3.603964000	-3.307912000	2.850591000
1	-1.873281000	-3.390485000	2.389624000
6	-3.469749000	-4.139618000	-0.428806000
1	-2.575095000	-4.772471000	-0.333021000
1	-4.336249000	-4.710505000	-0.055931000
1	-3.639760000	-3.918621000	-1.492714000
14	-5.696354000	-0.813370000	-0.648844000
6	-5.115475000	-0.992652000	-2.415768000
1	-5.088982000	-2.051103000	-2.720381000
1	-5.800203000	-0.469591000	-3.102200000
1	-4.112463000	-0.560908000	-2.550780000
6	-5.868361000	0.947825000	-0.081261000
1	-6.615854000	1.480701000	-0.689971000
1	-6.194485000	0.983278000	0.969505000
1	-4.909801000	1.480565000	-0.160523000
6	-7.259144000	-1.797916000	-0.353158000
1	-7.554705000	-1.761383000	0.706890000
1	-8.092315000	-1.390625000	-0.947959000
1	-7.130285000	-2.853735000	-0.639232000
14	6.451284000	-1.648432000	-0.324008000
6	5.544850000	-1.871558000	-1.959757000
1	6.111125000	-1.416295000	-2.788218000
1	5.407878000	-2.939712000	-2.193424000
1	4.550112000	-1.401838000	-1.930877000
6	5.492704000	-2.481290000	1.067526000
1	5.371021000	-3.559069000	0.872669000
1	6.020000000	-2.370724000	2.028796000
1	4.489897000	-2.041991000	1.178252000
6	8.157982000	-2.445047000	-0.454382000
1	8.750932000	-1.984263000	-1.260860000

1	8.723907000	-2.331937000	0.484405000
1	8.079190000	-3.522814000	-0.670197000
6	6.653401000	0.186119000	0.049096000
1	5.672375000	0.677785000	0.132832000
1	7.193344000	0.340365000	0.997174000
1	7.220710000	0.692483000	-0.748349000

Iso1

6	0.790355000	-0.135342000	0.650621000
1	-0.214785000	-0.169136000	1.065017000
5	3.797978000	-0.094396000	-0.539217000
5	2.879898000	1.421598000	-0.273554000
5	3.443047000	0.461453000	1.137411000
5	3.294202000	-1.284096000	0.708572000
5	2.637305000	-1.401266000	-0.967030000
5	2.380072000	0.269911000	-1.576361000
5	1.188180000	1.000030000	-0.507744000
5	1.808744000	1.158314000	1.131137000
5	2.060572000	-0.498704000	1.731256000
5	1.567698000	-1.640126000	0.441786000
5	1.009063000	-0.688560000	-0.955967000
17	5.441469000	-0.064584000	-1.195567000
17	3.471423000	3.049188000	-0.660529000
17	4.687844000	1.066823000	2.241845000
17	4.388131000	-2.507634000	1.371229000
17	3.036173000	-2.739804000	-2.056165000
17	2.464046000	0.718063000	-3.291021000
17	0.057484000	2.307208000	-1.162240000
17	1.169599000	2.443057000	2.181205000
17	1.695982000	-0.871768000	3.419622000
17	0.720735000	-3.134171000	0.862371000
17	-0.397156000	-1.214161000	-1.911838000
14	-2.154864000	2.265015000	-0.513919000
6	-2.227975000	1.350342000	1.091367000
1	-3.175308000	1.644386000	1.571122000
1	-2.268290000	0.263077000	0.949398000
1	-1.407586000	1.635322000	1.766378000
6	-2.951730000	1.480646000	-1.981389000
1	-2.320790000	0.659579000	-2.351476000
1	-3.909045000	1.041067000	-1.656092000
1	-3.116195000	2.202983000	-2.793275000
6	-2.334927000	4.103064000	-0.343564000
1	-2.080030000	4.625773000	-1.277541000
1	-3.385665000	4.335716000	-0.104238000
1	-1.698525000	4.488298000	0.466875000
8	-5.022859000	-0.374179000	-0.154200000
14	-4.577401000	-1.988472000	-0.362009000
14	-6.225235000	0.499097000	0.617695000
6	-5.933768000	2.312220000	0.216753000
6	-7.913471000	-0.003196000	-0.018511000
1	-8.171110000	-1.029750000	0.281866000
1	-8.691843000	0.666887000	0.380134000
1	-7.951803000	0.051180000	-1.117856000
6	-6.099682000	0.222617000	2.467641000
1	-5.101277000	0.490762000	2.848117000
1	-6.842020000	0.826957000	3.012849000
1	-6.279682000	-0.833798000	2.721660000
6	-4.204711000	-2.238065000	-2.176936000
1	-3.376739000	-1.594627000	-2.509277000
1	-3.910608000	-3.281523000	-2.372004000
1	-5.086116000	-2.011066000	-2.796634000
6	-5.986996000	-3.106093000	0.170551000
1	-6.265557000	-2.944363000	1.223908000
1	-6.883175000	-2.953855000	-0.450274000
1	-5.686494000	-4.161171000	0.068122000
6	-3.063688000	-2.330819000	0.685170000
1	-2.165321000	-1.850616000	0.269515000

1	-3.203821000	-1.996420000	1.725065000
1	-2.848592000	-3.410789000	0.709644000
1	-6.762088000	2.923361000	0.608866000
1	-5.009454000	2.694635000	0.676025000
1	-5.881850000	2.480912000	-0.870223000

Iso2

6	-0.880893000	0.104428000	0.285587000
1	-1.977710000	0.033540000	0.290566000
5	2.284897000	0.304363000	0.254256000
5	1.401950000	0.521478000	1.774908000
5	1.504074000	-1.098374000	0.991677000
5	1.464768000	-0.852805000	-0.795546000
5	1.355142000	0.914120000	-1.120289000
5	1.311412000	1.767669000	0.468958000
5	-0.160108000	1.250466000	1.329054000
5	-0.045233000	-0.505045000	1.648855000
5	0.000640000	-1.346246000	0.077369000
5	-0.098390000	-0.112991000	-1.213627000
5	-0.186852000	1.491231000	-0.442038000
17	4.117958000	0.595860000	0.397071000
17	2.222725000	0.855310000	3.311370000
17	2.411079000	-2.439387000	1.726573000
17	2.254127000	-1.980634000	-1.931498000
17	2.114558000	1.643991000	-2.552687000
17	2.037592000	3.373129000	0.671295000
17	-1.122424000	2.279773000	2.389498000
17	-0.886115000	-1.214557000	3.027276000
17	-0.765505000	-2.922706000	-0.136483000
17	-1.002841000	-0.451648000	-2.692401000
17	-1.150359000	2.780162000	-1.166530000
14	5.590721000	-0.609946000	-0.844984000
6	5.397869000	-2.365611000	-0.304482000
1	6.185930000	-2.958919000	-0.798982000
1	5.523746000	-2.455185000	0.783996000
1	4.418695000	-2.776957000	-0.582231000
6	7.103896000	0.219366000	-0.151779000
1	7.197948000	0.063160000	0.933070000
1	7.990246000	-0.225572000	-0.634888000
1	7.113654000	1.299340000	-0.360765000
6	5.247846000	-0.168281000	-2.606034000
1	5.315132000	0.918807000	-2.755792000
1	6.014140000	-0.656330000	-3.232249000
1	4.256450000	-0.510597000	-2.930472000
8	-4.415260000	-0.180814000	0.001421000
14	-4.806849000	-1.814938000	0.000310000
14	-5.025371000	1.276201000	-0.572063000
6	-4.421024000	2.622626000	0.573791000
6	-4.444840000	1.512995000	-2.337879000
1	-4.868710000	0.731492000	-2.988668000
1	-4.769670000	2.490376000	-2.729648000
1	-3.349441000	1.461835000	-2.417387000
6	-6.904830000	1.274036000	-0.546152000
1	-7.298483000	1.039210000	0.454795000
1	-7.270255000	2.276732000	-0.822468000
1	-7.334636000	0.556152000	-1.260501000
6	-4.167606000	-2.573847000	-1.588829000
1	-3.088264000	-2.404845000	-1.712169000
1	-4.348121000	-3.660653000	-1.608611000
1	-4.681385000	-2.130079000	-2.456622000
6	-6.666740000	-2.072649000	0.095116000
1	-7.106230000	-1.551683000	0.959742000
1	-7.191644000	-1.736792000	-0.811446000
1	-6.870070000	-3.149412000	0.216323000
6	-4.040922000	-2.575261000	1.525790000
1	-2.946836000	-2.481377000	1.542029000

1	-4.436088000	-2.088174000	2.431090000
1	-4.286355000	-3.648029000	1.582030000
1	-4.810263000	3.602230000	0.252930000
1	-4.775275000	2.434881000	1.599561000
1	-3.325003000	2.690811000	0.601821000

Iso3

6	-3.505427000	0.812606000	1.089125000
1	-4.320949000	1.207915000	1.697510000
5	-1.139319000	-0.325135000	-0.671017000
5	-2.680341000	0.107478000	-1.435908000
5	-1.583127000	1.384142000	-0.788898000
5	-0.783977000	0.730602000	0.696859000
5	-1.400180000	-0.940276000	0.970454000
5	-2.563220000	-1.332599000	-0.348624000
5	-3.935210000	-0.206451000	-0.214407000
5	-3.334531000	1.457303000	-0.478030000
5	-2.169320000	1.842329000	0.824296000
5	-2.059515000	0.418875000	1.900876000
5	-3.150752000	-0.845964000	1.259888000
17	0.109654000	-1.119936000	-1.794571000
17	-2.961211000	-0.067619000	-3.178779000
17	-0.790338000	2.554333000	-1.868096000
17	0.861724000	1.200872000	1.190408000
17	-0.404274000	-2.200030000	1.734052000
17	-2.711065000	-2.986069000	-0.975900000
17	-5.620158000	-0.600780000	-0.558747000
17	-4.419041000	2.708942000	-1.087226000
17	-2.103997000	3.469803000	1.505035000
17	-1.877597000	0.640210000	3.642614000
17	-4.053452000	-1.876587000	2.372090000
14	2.328341000	-0.482380000	-1.854046000
8	2.967209000	-1.046368000	-0.490269000
6	2.378598000	1.336386000	-2.072464000
1	1.996444000	1.863604000	-1.189424000
1	3.435688000	1.607595000	-2.224766000
1	1.794508000	1.637805000	-2.952614000
6	2.720488000	-1.503321000	-3.348042000
1	2.159023000	-1.168947000	-4.232749000
1	3.796822000	-1.386638000	-3.558127000
1	2.519211000	-2.570904000	-3.177664000
14	3.415811000	-2.410309000	0.440049000
6	2.295762000	-3.824244000	-0.047516000
1	2.484201000	-4.139707000	-1.086234000
1	2.486667000	-4.696591000	0.598029000
1	1.233052000	-3.560983000	0.054867000
6	3.255408000	-1.882066000	2.215309000
1	3.507431000	-2.708570000	2.897989000
1	3.932380000	-1.041135000	2.430696000
1	2.226775000	-1.555959000	2.427747000
6	5.180923000	-2.812633000	-0.031852000
1	5.872115000	-1.991224000	0.206017000
1	5.521775000	-3.707035000	0.514222000
1	5.263415000	-3.029803000	-1.108512000
14	6.186064000	1.614184000	0.408870000
6	7.271180000	3.148358000	0.304908000
1	8.301373000	2.891311000	0.010991000
1	6.878660000	3.864302000	-0.434766000
1	7.318129000	3.664205000	1.277152000
6	4.438528000	2.089118000	0.916209000
1	3.770504000	1.214486000	0.887834000
1	4.419020000	2.496390000	1.939451000
1	4.017453000	2.856651000	0.247759000
6	6.906546000	0.429438000	1.685028000
1	6.266652000	-0.454345000	1.832304000
1	7.906850000	0.076413000	1.387726000

1	7.005444000	0.929738000	2.661626000
6	6.144261000	0.776315000	-1.285376000
1	7.140396000	0.403642000	-1.571915000
1	5.452264000	-0.079862000	-1.279071000
1	5.819695000	1.483050000	-2.066530000

Iso4

6	1.047159000	2.480015000	0.772944000
1	1.618609000	3.273439000	1.258374000
5	-0.604687000	0.178133000	-0.629331000
5	0.947151000	0.693874000	-1.316398000
5	0.884836000	-0.211360000	0.241260000
5	-0.599631000	0.307096000	1.129808000
5	-1.446872000	1.540270000	0.124318000
5	-0.499132000	1.777226000	-1.390276000
5	1.093795000	2.427488000	-0.936938000
5	1.936946000	1.213070000	0.066256000
5	0.995090000	0.968627000	1.565403000
5	-0.435066000	2.041560000	1.494797000
5	-0.371101000	2.944275000	-0.050095000
17	-1.545051000	-1.079538000	-1.619019000
17	1.641209000	-0.040155000	-2.774304000
17	1.545070000	-1.854719000	0.387763000
17	-1.496685000	-0.791618000	2.206492000
17	-3.215523000	1.723460000	0.141456000
17	-1.302643000	2.149902000	-2.927253000
17	2.023403000	3.602494000	-1.867679000
17	3.702514000	1.189583000	0.119876000
17	1.829022000	0.688981000	3.095503000
17	-1.025656000	2.824634000	2.961753000
17	-0.899375000	4.626595000	-0.108708000
14	-2.535152000	-2.931469000	-0.654262000
8	-3.742376000	-2.378962000	0.246138000
6	-1.229591000	-3.836221000	0.262469000
1	-0.882803000	-3.263533000	1.132277000
1	-1.674965000	-4.783676000	0.609519000
1	-0.370418000	-4.059537000	-0.384859000
6	-3.053492000	-3.638311000	-2.286155000
1	-2.187044000	-3.884770000	-2.917592000
1	-3.615554000	-4.567553000	-2.095675000
1	-3.711115000	-2.948489000	-2.835199000
14	-5.309223000	-1.714161000	0.391155000
6	-5.625193000	-0.707563000	-1.150905000
1	-5.684214000	-1.354080000	-2.040979000
1	-6.581855000	-0.166990000	-1.072342000
1	-4.831253000	0.037801000	-1.308136000
6	-5.321784000	-0.725289000	1.964618000
1	-6.309723000	-0.266259000	2.127213000
1	-5.099041000	-1.372634000	2.826664000
1	-4.566767000	0.073177000	1.932358000
6	-6.467812000	-3.180480000	0.479901000
1	-6.229634000	-3.824358000	1.340771000
1	-7.510696000	-2.843139000	0.591878000
1	-6.412532000	-3.792430000	-0.434288000
14	5.924841000	-2.304815000	0.156241000
6	7.026837000	-0.796586000	-0.096424000
1	7.718236000	-0.662682000	0.750829000
1	7.631316000	-0.898164000	-1.012007000
1	6.426826000	0.121632000	-0.191435000
6	4.964839000	-2.132428000	1.767118000
1	4.332301000	-1.232184000	1.768415000
1	4.307101000	-3.000745000	1.931944000
1	5.654948000	-2.066553000	2.623829000
6	7.012787000	-3.844551000	0.259539000
1	7.600486000	-3.980193000	-0.662653000
1	7.720173000	-3.776515000	1.101652000

1	6.404336000	-4.751623000	0.405968000
6	4.756905000	-2.482003000	-1.310363000
1	4.094430000	-3.353691000	-1.187875000
1	4.118600000	-1.593470000	-1.427585000
1	5.325349000	-2.619207000	-2.244467000

Iso5

6	-0.368117000	-0.666823000	-1.427211000
1	-0.383715000	-1.250739000	-2.349277000
5	-0.344245000	1.071880000	1.297328000
5	-1.448290000	1.472513000	-0.053611000
5	-1.701414000	-0.029855000	0.909400000
5	-0.088752000	-0.713076000	1.312254000
5	1.116753000	0.367540000	0.578035000
5	0.321551000	1.725110000	-0.240310000
5	-0.621247000	1.019609000	-1.573093000
5	-1.862242000	-0.054283000	-0.868777000
5	-1.038827000	-1.396829000	-0.030437000
5	0.714512000	-1.153004000	-0.218050000
5	0.976840000	0.343540000	-1.171603000
17	-0.259934000	2.016342000	2.796255000
17	-2.579876000	2.832390000	0.006308000
17	-3.086200000	-0.241768000	1.990884000
17	0.263636000	-1.611939000	2.801069000
17	2.691061000	0.527569000	1.541814000
17	1.030308000	3.352128000	-0.351910000
17	-0.871644000	1.770245000	-3.150823000
17	-3.345181000	-0.365647000	-1.775776000
17	-1.706581000	-3.028921000	-0.088274000
17	1.792683000	-2.544697000	-0.429612000
17	2.315559000	0.423639000	-2.334301000
14	4.676362000	1.166921000	0.556059000
8	5.108411000	-0.061407000	-0.382690000
6	5.584353000	1.262534000	2.166889000
1	5.578785000	0.294890000	2.689981000
1	6.632838000	1.531174000	1.956685000
1	5.159839000	2.030364000	2.830488000
6	4.378736000	2.743743000	-0.330934000
1	3.923945000	3.495331000	0.328550000
1	5.361636000	3.110998000	-0.671231000
1	3.730587000	2.598555000	-1.204931000
14	5.754887000	-1.630568000	-0.571798000
6	5.077958000	-2.280734000	-2.176740000
1	5.396195000	-1.642254000	-3.015160000
1	5.439199000	-3.303236000	-2.368909000
1	3.978849000	-2.296375000	-2.156095000
6	5.249011000	-2.628154000	0.924623000
1	5.553462000	-3.680347000	0.806532000
1	5.730207000	-2.248149000	1.839940000
1	4.158292000	-2.609540000	1.068083000
6	7.610476000	-1.406536000	-0.636698000
1	7.997538000	-0.975718000	0.300223000
1	8.113260000	-2.374746000	-0.790356000
1	7.899949000	-0.743354000	-1.466642000
14	-7.038147000	-0.670290000	-0.046722000
6	-7.204468000	-0.629137000	-1.925686000
1	-7.634955000	-1.568100000	-2.309216000
1	-7.859391000	0.197267000	-2.245572000
1	-6.225001000	-0.485044000	-2.407561000
6	-5.968171000	-2.132927000	0.464515000
1	-4.962095000	-2.069978000	0.023794000
1	-5.847517000	-2.171191000	1.558722000
1	-6.425424000	-3.082265000	0.141552000
6	-8.756718000	-0.877072000	0.707264000
1	-9.418823000	-0.043874000	0.421558000
1	-9.230733000	-1.813735000	0.372121000

1	-8.705640000	-0.902263000	1.807736000
6	-6.297848000	0.951762000	0.554436000
1	-6.184863000	0.955161000	1.650070000
1	-5.301932000	1.128600000	0.121576000
1	-6.944774000	1.800471000	0.279117000

Iso6

6	0.790170000	-0.135514000	0.650292000
1	-0.215077000	-0.169417000	1.064420000
5	3.798108000	-0.094180000	-0.538732000
5	2.879774000	1.421687000	-0.273320000
5	3.442655000	0.461617000	1.137793000
5	3.294137000	-1.283951000	0.708912000
5	2.637732000	-1.401215000	-0.966869000
5	2.380435000	0.269935000	-1.576267000
5	1.188178000	0.999895000	-0.507954000
5	1.808280000	1.158284000	1.131080000
5	2.060131000	-0.498713000	1.731248000
5	1.567759000	-1.640218000	0.441648000
5	1.009412000	-0.688726000	-0.956268000
17	5.441770000	-0.064147000	-1.194645000
17	3.471221000	3.049335000	-0.660171000
17	4.687120000	1.067094000	2.242544000
17	4.388083000	-2.507321000	1.371856000
17	3.037132000	-2.739688000	-2.055890000
17	2.464839000	0.718131000	-3.290892000
17	0.057392000	2.306794000	-1.162810000
17	1.168670000	2.442962000	2.180948000
17	1.695127000	-0.871839000	3.419517000
17	0.720899000	-3.134374000	0.862048000
17	-0.396542000	-1.214303000	-1.912554000
14	-2.154975000	2.264589000	-0.514555000
6	-2.228466000	1.349831000	1.090646000
1	-3.176219000	1.643704000	1.569695000
1	-2.268457000	0.262533000	0.948764000
1	-1.408554000	1.635069000	1.766121000
6	-2.951948000	1.480362000	-1.982050000
1	-2.320941000	0.659526000	-2.352531000
1	-3.909071000	1.040529000	-1.656509000
1	-3.116875000	2.202843000	-2.793713000
6	-2.334831000	4.102658000	-0.344153000
1	-2.079615000	4.625320000	-1.278073000
1	-3.385540000	4.335589000	-0.104996000
1	-1.698442000	4.487714000	0.466387000
8	-5.022884000	-0.374051000	-0.154016000
14	-4.577525000	-1.988258000	-0.362534000
14	-6.224953000	0.499152000	0.618414000
6	-5.933748000	2.312262000	0.217282000
6	-7.913433000	-0.003298000	-0.017040000
1	-8.170988000	-1.029693000	0.283964000
1	-8.691689000	0.666997000	0.381466000
1	-7.952071000	0.050471000	-1.116403000
6	-6.098652000	0.222388000	2.468258000
1	-5.100762000	0.492236000	2.848866000
1	-6.842136000	0.825067000	3.013743000
1	-6.276661000	-0.834486000	2.721788000
6	-4.205956000	-2.237466000	-2.177748000
1	-3.377639000	-1.594572000	-2.510286000
1	-3.912786000	-3.281091000	-2.373332000
1	-5.087489000	-2.009559000	-2.796931000
6	-5.986592000	-3.106205000	0.170705000
1	-6.264011000	-2.945292000	1.224488000
1	-6.883444000	-2.953506000	-0.449034000
1	-5.686198000	-4.161204000	0.067137000
6	-3.063064000	-2.330482000	0.683564000
1	-2.165449000	-1.848664000	0.268154000

1	-3.203128000	-1.997603000	1.723955000
1	-2.846669000	-3.410222000	0.706478000
1	-6.762207000	2.923319000	0.609231000
1	-5.009521000	2.695055000	0.676427000
1	-5.881896000	2.480708000	-0.869742000

Iso7

6	-0.718344000	0.202125000	0.331217000
1	-1.125762000	1.188819000	0.539690000
5	0.416336000	-2.756757000	-0.287725000
5	0.829941000	-1.354354000	-1.340755000
5	1.478964000	-1.449760000	0.337732000
5	0.206685000	-2.164568000	1.401492000
5	-1.232041000	-2.511321000	0.377857000
5	-0.844947000	-2.012732000	-1.320558000
5	-0.561089000	-0.251168000	-1.319614000
5	0.850526000	0.094360000	-0.299964000
5	0.481708000	-0.401441000	1.378670000
5	-1.176950000	-1.046715000	1.404347000
5	-1.757915000	-0.957097000	-0.251682000
17	1.034310000	-4.380271000	-0.627037000
17	1.878053000	-1.474653000	-2.763652000
17	3.202667000	-1.692439000	0.659178000
17	0.599099000	-3.140781000	2.825238000
17	-2.395515000	-3.807014000	0.715183000
17	-1.615964000	-2.797689000	-2.712106000
17	-1.075995000	0.861532000	-2.606629000
17	1.767618000	1.583177000	-0.601036000
17	1.064936000	0.579844000	2.729262000
17	-2.291471000	-0.672245000	2.737197000
17	-3.551509000	-0.637541000	-0.568731000
14	-4.188895000	1.547011000	-0.251289000
8	-2.849559000	2.347389000	0.159573000
6	-5.374927000	1.299178000	1.135974000
1	-4.840027000	0.892323000	2.007044000
1	-5.814946000	2.271039000	1.412954000
1	-6.189345000	0.612993000	0.860782000
6	-4.838002000	1.915235000	-1.937018000
1	-5.650150000	1.229350000	-2.220055000
1	-5.231649000	2.944648000	-1.958838000
1	-4.024047000	1.832581000	-2.672344000
14	-2.098249000	3.888878000	0.319034000
6	-1.346134000	4.318654000	-1.327386000
1	-2.099995000	4.344276000	-2.129294000
1	-0.869458000	5.310726000	-1.277823000
1	-0.575357000	3.583187000	-1.602663000
6	-0.827555000	3.699589000	1.663864000
1	-0.469794000	4.691921000	1.981866000
1	-1.244294000	3.189927000	2.546072000
1	0.051773000	3.130302000	1.325337000
6	-3.481012000	5.054999000	0.797555000
1	-3.952699000	4.749058000	1.744759000
1	-3.096485000	6.078171000	0.933673000
1	-4.258859000	5.100016000	0.017988000
14	5.783723000	1.868267000	-0.090233000
6	5.080760000	3.505691000	-0.714990000
1	5.270661000	4.317612000	0.005447000
1	5.538165000	3.793399000	-1.675291000
1	3.992592000	3.438098000	-0.868439000
6	5.003012000	1.441739000	1.568616000
1	3.910368000	1.343077000	1.487488000
1	5.390161000	0.484797000	1.952518000
1	5.223551000	2.220278000	2.316972000
6	7.646517000	2.066336000	0.142936000
1	8.142470000	2.327430000	-0.805776000
1	7.875260000	2.859951000	0.872402000

1	8.099313000	1.131764000	0.511398000
6	5.464963000	0.513998000	-1.356561000
1	5.862565000	-0.452288000	-1.008147000
1	4.389985000	0.375499000	-1.545193000
1	5.952744000	0.753640000	-2.315265000

Iso8

6	3.428124000	-1.063971000	0.316059000
1	4.388606000	-1.556237000	0.479441000
5	0.650244000	0.370750000	-0.157913000
5	2.095485000	1.332060000	0.173095000
5	1.909914000	0.463447000	-1.398106000
5	1.061306000	-1.092451000	-1.072194000
5	0.715529000	-1.181340000	0.698644000
5	1.349210000	0.320704000	1.467247000
5	3.112907000	0.335167000	1.238258000
5	3.457218000	0.423233000	-0.519009000
5	2.825053000	-1.063309000	-1.280716000
5	2.086707000	-2.071095000	0.002098000
5	2.267342000	-1.204914000	1.559816000
17	-1.076962000	0.973199000	-0.439714000
17	2.162932000	3.111759000	0.276022000
17	1.694564000	1.334778000	-2.932255000
17	-0.035722000	-1.804769000	-2.273221000
17	-0.739961000	-1.989555000	1.320553000
17	0.551201000	1.050853000	2.878221000
17	4.284798000	0.968109000	2.395899000
17	4.967267000	1.141742000	-1.082407000
17	3.709305000	-1.824018000	-2.604204000
17	2.240154000	-3.827575000	-0.054751000
17	2.599252000	-2.104345000	3.040833000
8	-4.148224000	-0.181221000	-0.063516000
14	-4.630032000	-0.249228000	1.542368000
14	-4.369880000	-1.117989000	-1.444057000
6	-3.525260000	-2.774320000	-1.247930000
1	-4.002685000	-3.385497000	-0.467253000
1	-3.572293000	-3.339445000	-2.192815000
1	-2.465306000	-2.646759000	-0.983599000
6	-6.213512000	-1.356109000	-1.715348000
1	-6.402833000	-1.912928000	-2.647071000
1	-6.669224000	-1.927349000	-0.890718000
1	-6.735169000	-0.388646000	-1.786791000
6	-3.633480000	-0.171667000	-2.883737000
1	-4.115771000	0.810282000	-3.006587000
1	-2.550395000	-0.024500000	-2.757695000
1	-3.782510000	-0.736309000	-3.817937000
6	-4.580313000	-2.015458000	2.163927000
1	-5.296896000	-2.654549000	1.624537000
1	-3.575817000	-2.447522000	2.041175000
1	-4.838682000	-2.054476000	3.234167000
6	-6.374338000	0.433882000	1.661441000
1	-7.080539000	-0.173127000	1.074066000
1	-6.725611000	0.445633000	2.705637000
1	-6.419174000	1.465687000	1.278300000
6	-3.457834000	0.831625000	2.522529000
1	-2.413944000	0.503827000	2.406425000
1	-3.537922000	1.881887000	2.201197000
1	-3.706412000	0.796150000	3.595086000
14	-1.570332000	3.204495000	-0.478644000
6	-3.373820000	2.986748000	-0.862564000
1	-3.778983000	2.060986000	-0.421598000
1	-3.930688000	3.846778000	-0.456243000
1	-3.537823000	2.944820000	-1.948876000
6	-0.584477000	3.905618000	-1.876302000
1	0.493709000	3.907805000	-1.671104000
1	-0.769336000	3.339842000	-2.800759000

1	-0.921485000	4.945147000	-2.031163000
6	-1.211167000	3.808105000	1.231135000
1	-1.561383000	4.852583000	1.296302000
1	-1.748645000	3.211545000	1.981109000
1	-0.137767000	3.781270000	1.459654000

Iso9

6	-3.579732000	0.033170000	0.726942000
1	-4.582378000	0.088343000	1.154145000
5	-0.642712000	-0.134347000	-0.533730000
5	-1.561930000	1.391099000	-0.544610000
5	-0.946272000	0.719681000	0.994494000
5	-1.069805000	-1.064112000	0.924510000
5	-1.772847000	-1.503211000	-0.668816000
5	-2.075927000	0.018854000	-1.581843000
5	-3.281936000	0.965478000	-0.672183000
5	-2.581351000	1.400655000	0.912455000
5	-2.277992000	-0.111429000	1.818100000
5	-2.791965000	-1.479994000	0.792295000
5	-3.411354000	-0.814726000	-0.747772000
17	1.010543000	-0.227180000	-1.223723000
17	-0.903185000	2.901663000	-1.232752000
17	0.352396000	1.546057000	1.915750000
17	0.102174000	-2.118100000	1.762053000
17	-1.343743000	-3.019932000	-1.492036000
17	-1.957355000	0.095795000	-3.352092000
17	-4.542269000	1.981063000	-1.383394000
17	-3.131266000	2.848432000	1.768260000
17	-2.530941000	-0.158257000	3.568791000
17	-3.567100000	-2.888237000	1.528249000
17	-4.805441000	-1.566522000	-1.531855000
14	4.096038000	-0.326529000	1.384696000
8	3.738630000	-1.510067000	0.352127000
6	3.125499000	-0.366434000	2.949850000
1	2.053425000	-0.502771000	2.748307000
1	3.476688000	-1.226648000	3.542821000
1	3.267554000	0.545323000	3.549185000
6	5.932473000	-0.117618000	1.620524000
1	6.174257000	0.741500000	2.265472000
1	6.331643000	-1.022554000	2.106437000
1	6.459416000	0.002351000	0.661499000
14	3.906064000	-2.646729000	-0.892602000
6	4.071460000	-1.686220000	-2.492200000
1	4.918979000	-0.982237000	-2.451948000
1	4.256090000	-2.372845000	-3.333796000
1	3.152899000	-1.124468000	-2.719138000
6	2.421971000	-3.759560000	-0.854521000
1	2.516361000	-4.557047000	-1.608660000
1	2.316614000	-4.233336000	0.133348000
1	1.495722000	-3.201847000	-1.055232000
6	5.492890000	-3.576908000	-0.531042000
1	5.437726000	-4.084944000	0.444685000
1	5.677564000	-4.347051000	-1.296897000
1	6.364433000	-2.903024000	-0.518462000
14	3.326270000	3.115460000	-0.657183000
6	2.687110000	2.648229000	-2.337762000
1	2.673595000	3.553112000	-2.967400000
1	3.330778000	1.903346000	-2.830531000
1	1.662855000	2.252345000	-2.285869000
6	3.580037000	1.412687000	0.392358000
1	4.268610000	1.029378000	-0.371792000
1	3.988044000	1.952424000	1.256369000
1	2.523049000	1.094302000	0.383773000
6	5.067415000	3.804164000	-0.745754000
1	5.052143000	4.751154000	-1.309461000
1	5.480950000	4.022650000	0.251270000

1	5.757512000	3.123205000	-1.268229000
6	2.199240000	4.206884000	0.337285000
1	1.198989000	3.763129000	0.444169000
1	2.606585000	4.415786000	1.338331000
1	2.089109000	5.168715000	-0.190054000

Iso10

6	3.542089000	-0.805834000	0.350583000
1	4.553681000	-1.176628000	0.526085000
5	0.609918000	0.280233000	-0.161311000
5	1.930598000	1.410425000	0.165709000
5	1.862142000	0.500602000	-1.395232000
5	1.202013000	-1.140815000	-1.044647000
5	0.859770000	-1.241740000	0.725299000
5	1.302941000	0.336179000	1.469732000
5	3.055195000	0.557742000	1.247545000
5	3.396291000	0.661473000	-0.507983000
5	2.951477000	-0.903095000	-1.247170000
5	2.333210000	-1.970153000	0.048711000
5	2.398650000	-1.065197000	1.592004000
17	-1.179285000	0.669839000	-0.471465000
17	1.784298000	3.183479000	0.290219000
17	1.574054000	1.294008000	-2.959332000
17	0.199211000	-2.008290000	-2.227203000
17	-0.469558000	-2.240437000	1.350432000
17	0.432093000	1.006786000	2.866617000
17	4.133316000	1.346337000	2.400506000
17	4.811792000	1.548135000	-1.077624000
17	3.928029000	-1.571132000	-2.555869000
17	2.694320000	-3.696628000	0.020848000
17	2.826643000	-1.895121000	3.088855000
8	-4.083350000	-0.218370000	-0.067801000
14	-4.628855000	-0.025734000	1.513965000
14	-4.260315000	-1.379906000	-1.282082000
6	-3.452761000	-2.981149000	-0.756328000
1	-3.883882000	-3.369727000	0.179123000
1	-3.591265000	-3.749974000	-1.533394000
1	-2.371901000	-2.844855000	-0.604179000
6	-6.090782000	-1.630768000	-1.619117000
1	-6.231221000	-2.273808000	-2.502812000
1	-6.602622000	-2.112603000	-0.772984000
1	-6.591200000	-0.670192000	-1.820681000
6	-3.449052000	-0.735291000	-2.843089000
1	-3.923073000	0.192274000	-3.197420000
1	-2.370073000	-0.563886000	-2.717701000
1	-3.567722000	-1.489963000	-3.637483000
6	-5.802299000	-1.408549000	1.996709000
1	-6.739054000	-1.378071000	1.420132000
1	-5.344298000	-2.400259000	1.859181000
1	-6.063614000	-1.311423000	3.062859000
6	-5.549421000	1.610473000	1.589451000
1	-6.369253000	1.634594000	0.854277000
1	-5.988796000	1.755274000	2.589397000
1	-4.891669000	2.470309000	1.390251000
6	-3.171145000	-0.009610000	2.678993000
1	-2.449049000	0.786206000	2.448307000
1	-3.515719000	0.139579000	3.714950000
1	-2.616268000	-0.958198000	2.636615000
14	-1.884963000	2.831993000	-0.727466000
6	-3.591125000	2.565288000	-1.391244000
1	-4.101275000	1.718198000	-0.912458000
1	-4.162111000	3.489043000	-1.196797000
1	-3.557761000	2.399194000	-2.477169000
6	-0.802789000	3.628738000	-1.996997000
1	0.242807000	3.739523000	-1.687834000
1	-0.840071000	3.068243000	-2.941736000

1	-1.231973000	4.630846000	-2.174805000
6	-1.792113000	3.472652000	1.006943000
1	-2.082558000	4.536662000	0.996793000
1	-2.481068000	2.931170000	1.670241000
1	-0.772485000	3.393915000	1.409367000

Iso11

6	0.415152000	-3.257182000	0.444609000
1	0.474466000	-4.314105000	0.711032000
5	0.248663000	-0.192898000	-0.321481000
5	-1.129292000	-1.261257000	-0.635507000
5	-0.680679000	-0.811083000	1.050688000
5	1.120621000	-0.688113000	1.128820000
5	1.776316000	-1.070416000	-0.505983000
5	0.388102000	-1.416457000	-1.599956000
5	-0.444150000	-2.864071000	-0.979643000
5	-1.095129000	-2.493471000	0.645125000
5	0.283169000	-2.140919000	1.729627000
5	1.788307000	-2.299960000	0.775461000
5	1.338175000	-2.747666000	-0.897661000
17	0.067481000	1.547545000	-0.932857000
17	-2.635012000	-0.686051000	-1.380424000
17	-1.753196000	0.185803000	2.058670000
17	1.957871000	0.445739000	2.217366000
17	3.272802000	-0.333247000	-1.121860000
17	0.441591000	-0.985922000	-3.319987000
17	-1.205826000	-4.108397000	-1.971448000
17	-2.509729000	-3.351889000	1.257830000
17	0.246020000	-2.664740000	3.414620000
17	3.238710000	-2.976850000	1.518866000
17	2.344410000	-3.874821000	-1.808728000
14	0.596057000	3.398211000	0.359748000
8	2.197525000	3.396447000	0.463119000
6	-0.282347000	3.261081000	1.963125000
1	0.115715000	2.442055000	2.575107000
1	-0.124434000	4.216183000	2.492175000
1	-1.358893000	3.110099000	1.808670000
6	-0.107261000	4.586023000	-0.873021000
1	-1.192992000	4.443634000	-0.981694000
1	0.073143000	5.609711000	-0.505408000
1	0.372114000	4.486568000	-1.857813000
14	3.743986000	3.597216000	-0.230856000
6	3.589186000	3.201421000	-2.050361000
1	2.958262000	3.942877000	-2.566087000
1	4.578048000	3.215415000	-2.535749000
1	3.155912000	2.201659000	-2.203701000
6	4.886918000	2.464901000	0.700296000
1	5.909974000	2.523016000	0.296494000
1	4.922285000	2.745537000	1.764186000
1	4.544015000	1.422488000	0.632449000
6	4.180453000	5.397570000	0.029761000
1	4.181966000	5.653686000	1.100646000
1	5.184692000	5.614459000	-0.368265000
1	3.467912000	6.062739000	-0.483124000
14	-5.370768000	2.051250000	-0.147385000
6	-5.751050000	1.427002000	-1.882448000
1	-6.733299000	0.928972000	-1.912646000
1	-5.773023000	2.256448000	-2.607388000
1	-4.996978000	0.700902000	-2.222581000
6	-3.678746000	2.894768000	-0.145177000
1	-3.648123000	3.706075000	-0.890784000
1	-3.467013000	3.330340000	0.844681000
1	-2.890501000	2.164996000	-0.383959000
6	-6.673338000	3.321808000	0.349109000
1	-7.680105000	2.874114000	0.352306000
1	-6.481159000	3.717568000	1.359370000

1	-6.688399000	4.173546000	-0.349783000
6	-5.377232000	0.626529000	1.078057000
1	-4.617978000	-0.125930000	0.817066000
1	-5.162954000	0.977956000	2.099920000
1	-6.360698000	0.130103000	1.092871000

Iso12

6	-3.666514000	0.045707000	0.461977000
1	-4.723599000	0.106775000	0.726155000
5	-0.575463000	-0.135909000	-0.316785000
5	-1.482188000	1.381364000	-0.514857000
5	-1.101329000	0.758514000	1.121638000
5	-1.221284000	-1.028620000	1.080822000
5	-1.660865000	-1.517150000	-0.590533000
5	-1.821999000	-0.022631000	-1.581256000
5	-3.156891000	0.939716000	-0.898108000
5	-2.709588000	1.425461000	0.766004000
5	-2.550503000	-0.058418000	1.748015000
5	-2.898579000	-1.459634000	0.690410000
5	-3.269079000	-0.842797000	-0.945846000
17	1.160001000	-0.248232000	-0.762347000
17	-0.749942000	2.881816000	-1.148053000
17	0.075730000	1.587668000	2.188586000
17	-0.214393000	-2.065793000	2.125140000
17	-1.074257000	-3.044691000	-1.288864000
17	-1.407352000	0.006795000	-3.308783000
17	-4.291849000	1.929269000	-1.824808000
17	-3.394543000	2.891984000	1.479948000
17	-3.079129000	-0.063469000	3.436013000
17	-3.775253000	-2.851424000	1.338197000
17	-4.523100000	-1.622424000	-1.917391000
8	4.155152000	-1.239478000	-0.119838000
14	3.316827000	3.269459000	-0.583785000
14	3.867032000	-2.773093000	-0.807791000
6	3.217149000	-2.506533000	-2.534585000
1	3.840868000	-1.789350000	-3.090565000
1	3.229101000	-3.460267000	-3.086264000
1	2.181660000	-2.136062000	-2.522596000
6	5.543706000	-3.609740000	-0.844321000
1	5.469638000	-4.611480000	-1.296756000
1	6.266095000	-3.026235000	-1.436445000
1	5.953689000	-3.732571000	0.170794000
6	2.670465000	-3.704231000	0.274809000
1	3.055394000	-3.823278000	1.299987000
1	1.693365000	-3.201611000	0.327105000
1	2.503002000	-4.712924000	-0.135981000
6	4.979117000	4.122726000	-0.709601000
1	5.409927000	4.339581000	0.280250000
1	5.706866000	3.530972000	-1.286446000
1	4.851267000	5.085533000	-1.230861000
6	2.134783000	4.203636000	0.501029000
1	2.601549000	4.477315000	1.459872000
1	1.840898000	5.135048000	-0.009780000
1	1.218163000	3.628777000	0.697687000
6	2.676445000	2.750004000	-2.248640000
1	3.424010000	2.158805000	-2.799961000
1	1.747468000	2.167542000	-2.169296000
1	2.455883000	3.652526000	-2.841778000
14	4.260510000	-0.269791000	1.175699000
6	6.031603000	-0.025325000	1.691816000
1	6.668067000	0.259311000	0.840508000
1	6.133250000	0.732970000	2.483525000
1	6.418382000	-0.977352000	2.090209000
6	3.100096000	-0.604398000	2.568319000
1	2.062042000	-0.712475000	2.222590000
1	3.394623000	-1.546632000	3.058258000

1	3.136824000	0.196747000	3.322142000
6	3.749049000	1.563312000	0.410128000
1	2.703728000	1.208408000	0.364513000
1	4.123873000	2.072881000	1.305219000
1	4.454488000	1.284854000	-0.384049000

Iso13

6	3.403352000	-0.536529000	0.104468000
1	4.481633000	-0.691733000	0.172729000
5	0.279483000	-0.077372000	-0.087362000
5	1.265039000	0.992869000	0.910399000
5	1.390840000	1.042930000	-0.888999000
5	1.180422000	-0.635425000	-1.510221000
5	0.938123000	-1.726163000	-0.092731000
5	0.985775000	-0.723562000	1.401426000
5	2.607850000	-0.003264000	1.518030000
5	2.854677000	1.078682000	0.114130000
5	2.804797000	0.079697000	-1.370382000
5	2.526277000	-1.618433000	-0.883776000
5	2.410035000	-1.669368000	0.901160000
17	-1.548483000	0.077819000	-0.355713000
17	0.616451000	2.359780000	1.847983000
17	0.860737000	2.449013000	-1.840305000
17	0.379338000	-0.956559000	-3.060959000
17	-0.082981000	-3.175114000	-0.202514000
17	0.031455000	-1.163907000	2.835563000
17	3.484054000	0.290724000	3.021710000
17	3.970551000	2.440901000	0.233364000
17	3.877112000	0.452802000	-2.720878000
17	3.323543000	-2.930026000	-1.753413000
17	3.087922000	-3.030052000	1.796483000
8	-2.381957000	2.987596000	0.572069000
14	-4.085486000	-3.585982000	-0.261890000
14	-2.374779000	4.365617000	-0.434688000
6	-3.868019000	5.368312000	0.082037000
1	-3.807916000	5.648305000	1.145283000
1	-3.933157000	6.297772000	-0.506026000
1	-4.803956000	4.809119000	-0.075187000
6	-0.787917000	5.271242000	-0.093532000
1	-0.723371000	6.184383000	-0.706192000
1	-0.733829000	5.565276000	0.965871000
1	0.082645000	4.638215000	-0.316724000
6	-2.548514000	3.767038000	-2.196389000
1	-1.781242000	3.015514000	-2.435837000
1	-3.541979000	3.323967000	-2.369992000
1	-2.432095000	4.603374000	-2.903871000
6	-5.961221000	-3.788909000	-0.258464000
1	-6.469888000	-2.812321000	-0.209028000
1	-6.307034000	-4.298361000	-1.172123000
1	-6.296865000	-4.385238000	0.605060000
6	-3.270794000	-5.278557000	-0.348777000
1	-3.569718000	-5.907063000	0.505082000
1	-3.558401000	-5.805440000	-1.272443000
1	-2.173351000	-5.194039000	-0.337593000
6	-3.589155000	-2.568211000	-1.768349000
1	-2.498911000	-2.427364000	-1.819884000
1	-3.903442000	-3.083138000	-2.690538000
1	-4.065256000	-1.574525000	-1.771998000
14	-2.869843000	1.495264000	0.903942000
6	-4.434493000	1.009412000	0.040391000
1	-4.379854000	1.230840000	-1.035570000
1	-4.668491000	-0.056304000	0.173711000
1	-5.258619000	1.602183000	0.470648000
6	-2.667719000	0.981432000	2.652596000
1	-1.660769000	1.218299000	3.020100000
1	-3.408375000	1.542871000	3.246292000

1	-2.851585000	-0.095150000	2.772926000
6	-3.579767000	-2.725554000	1.341181000
1	-2.499857000	-2.515064000	1.367510000
1	-3.816653000	-3.365977000	2.205969000
1	-4.128505000	-1.780252000	1.480333000

CB_dicat_CB

14	0.216494000	-0.002979000	1.338778000
14	3.075853000	-0.890076000	0.000000000
8	1.303602000	-0.437840000	0.000000000
6	0.863063000	1.511047000	2.126245000
1	1.606984000	1.218728000	2.886430000
1	0.057133000	2.052984000	2.645146000
1	1.317947000	2.219421000	1.420229000
6	-0.341628000	-1.323883000	2.480317000
1	-1.028299000	-2.057835000	2.039815000
1	-0.808108000	-0.836467000	3.351894000
1	0.527986000	-1.895537000	2.842481000
6	3.943656000	0.751615000	0.000000000
1	3.706508000	1.353564000	-0.889700000
1	5.032251000	0.577762000	0.000000000
1	3.706508000	1.353564000	0.889700000
6	3.307671000	-1.838560000	-1.573558000
1	2.744707000	-2.783261000	-1.584347000
1	4.377809000	-2.090012000	-1.658552000
1	3.054063000	-1.240499000	-2.463246000
6	3.307671000	-1.838560000	1.573558000
1	3.054063000	-1.240499000	2.463246000
1	4.377809000	-2.090012000	1.658552000
1	2.744707000	-2.783261000	1.584347000
14	0.216494000	-0.002979000	-1.338778000
14	-2.756026000	0.326141000	0.000000000
8	-0.917718000	0.259766000	0.000000000
6	-0.341628000	-1.323883000	-2.480317000
1	-0.808108000	-0.836467000	-3.351894000
1	0.527986000	-1.895537000	-2.842481000
1	-1.028299000	-2.057835000	-2.039815000
6	0.863063000	1.511047000	-2.126245000
1	1.317947000	2.219421000	-1.420229000
1	1.606984000	1.218728000	-2.886430000
1	0.057133000	2.052984000	-2.645146000
6	-3.307643000	-1.440214000	0.000000000
1	-2.982085000	-2.006076000	0.884147000
1	-4.410621000	-1.447034000	0.000000000
1	-2.982085000	-2.006076000	-0.884147000
6	-3.155179000	1.214269000	1.577929000
1	-2.795378000	2.253339000	1.584445000
1	-4.252446000	1.236252000	1.683973000
1	-2.767879000	0.680162000	2.460033000
6	-3.155179000	1.214269000	-1.577929000
1	-2.767879000	0.680162000	-2.460033000
1	-4.252446000	1.236252000	-1.683973000
1	-2.795378000	2.253339000	-1.584445000
6	0.483010000	8.092469000	0.000000000
1	0.794240000	9.138704000	0.000000000
5	-0.427666000	5.044557000	0.000000000
5	-1.357056000	6.258038000	0.900662000
5	-1.357056000	6.258038000	-0.900662000
5	0.283625000	5.775584000	-1.454480000
5	1.289004000	5.473568000	0.000000000
5	0.283625000	5.775584000	1.454480000
5	-0.189939000	7.487561000	1.446534000
5	-1.200038000	7.784968000	0.000000000
5	-0.189939000	7.487561000	-1.446534000
5	1.442168000	7.000082000	-0.894771000
5	1.442168000	7.000082000	0.894771000
17	-0.945602000	3.326704000	0.000000000

17	-2.807836000	5.821707000	1.833998000
17	-2.807836000	5.821707000	-1.833998000
17	0.529172000	4.874578000	-2.975690000
17	2.576565000	4.220659000	0.000000000
17	0.529172000	4.874578000	2.975690000
17	-0.363303000	8.508669000	2.878159000
17	-2.371372000	9.107214000	0.000000000
17	-0.363303000	8.508669000	-2.878159000
17	2.878361000	7.533144000	-1.777496000
17	2.878361000	7.533144000	1.777496000
6	-0.396693000	-8.050216000	0.000000000
1	-0.569665000	-9.128106000	0.000000000
5	0.103516000	-4.922258000	0.000000000
5	-1.257216000	-5.606253000	-0.898377000
5	-1.257216000	-5.606253000	0.898377000
5	0.430417000	-5.876029000	1.454400000
5	1.472862000	-6.045423000	0.000000000
5	0.430417000	-5.876029000	-1.454400000
5	-0.739040000	-7.214780000	-1.449934000
5	-1.780125000	-7.047685000	0.000000000
5	-0.739040000	-7.214780000	1.449934000
5	0.943070000	-7.484897000	0.896651000
5	0.943070000	-7.484897000	-0.896651000
17	0.419215000	-3.163233000	0.000000000
17	-2.387503000	-4.591957000	-1.841954000
17	-2.387503000	-4.591957000	1.841954000
17	1.048530000	-5.111376000	2.943658000
17	3.156677000	-5.457732000	0.000000000
17	1.048530000	-5.111376000	-2.943658000
17	-1.348302000	-8.055348000	-2.879102000
17	-3.411703000	-7.724732000	0.000000000
17	-1.348302000	-8.055348000	2.879102000
17	1.996057000	-8.594323000	1.779892000
17	1.996057000	-8.594323000	-1.779892000

CB_dicat_CB iso1

14	-0.022476000	0.217739000	-1.222344000
14	-0.900015000	3.082797000	0.103047000
8	-0.439001000	1.313510000	0.118085000
6	1.473017000	0.876365000	-2.035278000
1	1.157016000	1.620871000	-2.785759000
1	2.008132000	0.076979000	-2.570836000
1	2.194014000	1.334230000	-1.344422000
6	-1.350375000	-0.358731000	-2.344347000
1	-2.036209000	-1.096301000	-1.908410000
1	-0.868098000	-0.772295000	-3.244867000
1	-1.979298000	0.490213000	-2.657479000
6	0.737793000	3.958150000	0.101575000
1	1.337330000	3.731452000	0.995686000
1	0.559459000	5.045960000	0.091346000
1	1.344033000	3.715441000	-0.783700000
6	-1.855795000	3.323824000	1.671165000
1	-2.790389000	2.744266000	1.687798000
1	-2.126721000	4.390493000	1.738104000
1	-1.255244000	3.095269000	2.565929000
6	-1.846536000	3.291631000	-1.474153000
1	-1.246628000	3.031879000	-2.360782000
1	-2.106853000	4.358491000	-1.571900000
1	-2.787252000	2.721825000	-1.479084000
14	0.028813000	0.240295000	1.454032000
14	0.320611000	-2.744546000	0.145454000
8	0.262750000	-0.906006000	0.123877000
6	-1.273705000	-0.299866000	2.626152000
1	-0.776948000	-0.804032000	3.471069000
1	-1.795596000	0.584031000	3.026933000
1	-2.045575000	-0.947486000	2.192309000
6	1.562590000	0.883282000	2.204574000
1	2.253805000	1.335900000	1.480064000
1	1.294339000	1.628015000	2.972441000
1	2.116003000	0.074508000	2.707011000
6	-1.445154000	-3.286669000	0.261376000
1	-2.092573000	-2.902604000	-0.539822000
1	-1.463198000	-4.387332000	0.191585000
1	-1.916166000	-3.017864000	1.217170000
6	1.125210000	-3.157178000	-1.473082000
1	2.154228000	-2.774337000	-1.539158000
1	1.170177000	-4.255330000	-1.560231000
1	0.535827000	-2.798673000	-2.331478000
6	1.292673000	-3.139432000	1.674823000
1	0.836061000	-2.705356000	2.578530000
1	1.270700000	-4.233436000	1.811086000
1	2.344358000	-2.826080000	1.608188000
6	8.093927000	0.475063000	-0.067864000
1	9.140858000	0.783311000	-0.087143000
5	5.043641000	-0.427155000	-0.012567000
5	6.233973000	-1.347240000	-0.953166000
5	6.274479000	-1.372220000	0.847401000
5	5.809252000	0.261526000	1.435232000
5	5.477938000	1.288304000	0.001969000
5	5.744464000	0.302485000	-1.472667000
5	7.454796000	-0.176012000	-1.509868000
5	7.781240000	-1.206933000	-0.084445000
5	7.519139000	-0.216366000	1.382197000
5	7.024442000	1.424512000	0.864451000
5	6.984881000	1.449479000	-0.924413000

17	3.324927000	-0.940901000	0.020988000
17	5.772311000	-2.783460000	-1.896399000
17	5.854929000	-2.835274000	1.769642000
17	4.943467000	0.487824000	2.979902000
17	4.230213000	2.580233000	0.046876000
17	4.811445000	0.572463000	-2.970112000
17	8.442907000	-0.332079000	-2.966346000
17	9.099418000	-2.381976000	-0.130121000
17	8.571334000	-0.412775000	2.788132000
17	7.581373000	2.846701000	1.755123000
17	7.502787000	2.896373000	-1.798631000
6	-8.047551000	-0.393320000	-0.139350000
1	-9.124199000	-0.562079000	-0.202414000
5	-4.922558000	0.093948000	0.043967000
5	-5.662463000	-1.268218000	0.896416000
5	-5.559714000	-1.260307000	-0.897925000
5	-5.789592000	0.429831000	-1.461298000
5	-6.038907000	1.467648000	-0.016102000
5	-5.957454000	0.419617000	1.442560000
5	-7.298957000	-0.744276000	1.355269000
5	-7.052369000	-1.780429000	-0.086223000
5	-7.130244000	-0.733964000	-1.540023000
5	-7.426293000	0.946892000	-0.996379000
5	-7.529839000	0.940962000	0.793730000
17	-3.165060000	0.400842000	0.158297000
17	-4.702775000	-2.401948000	1.888617000
17	-4.502649000	-2.397579000	-1.786776000
17	-4.945076000	1.053587000	-2.906475000
17	-5.447856000	3.150076000	0.022471000
17	-5.276909000	1.029031000	2.973758000
17	-8.225352000	-1.355291000	2.729552000
17	-7.734730000	-3.409143000	-0.133957000
17	-7.888235000	-1.333902000	-3.018530000
17	-8.477467000	2.007414000	-1.939946000
17	-8.684785000	1.995837000	1.613921000

CB_dicat_CB iso2

14	0.000099000	-0.000046000	-1.333759000
14	-0.637887000	2.920558000	0.000930000
8	-0.410045000	1.092131000	0.000752000
6	1.456897000	0.587845000	-2.250340000
1	1.137055000	1.254429000	-3.067370000
1	1.990935000	-0.259218000	-2.711419000
1	2.190265000	1.128701000	-1.635757000
6	-1.456887000	-0.587880000	-2.250105000
1	-2.190018000	-1.128916000	-1.635406000
1	-1.137253000	-1.254260000	-3.067379000
1	-1.991162000	0.259225000	-2.710838000
6	1.077242000	3.624091000	0.001927000
1	1.664458000	3.351007000	0.890701000
1	0.989900000	4.723832000	0.000824000
1	1.666514000	3.349382000	-0.884990000
6	-1.545327000	3.192287000	1.589533000
1	-2.507081000	2.660251000	1.642053000
1	-1.774218000	4.267073000	1.678690000
1	-0.921888000	2.931840000	2.459937000
6	-1.544018000	3.192725000	-1.588353000
1	-0.919893000	2.932518000	-2.458333000
1	-1.772844000	4.267533000	-1.677396000
1	-2.505739000	2.660710000	-1.641784000
14	-0.000008000	-0.000074000	1.335246000
14	0.637983000	-2.920671000	0.000927000
8	0.410295000	-1.092189000	0.000747000
6	-1.457153000	-0.588039000	2.251276000
1	-1.137671000	-1.254537000	3.068514000
1	-1.991592000	0.258968000	2.711997000
1	-2.190085000	-1.129030000	1.636284000
6	1.456783000	0.587672000	2.251973000
1	2.190094000	1.128728000	1.637483000
1	1.136885000	1.253991000	3.069198000
1	1.990907000	-0.259496000	2.712757000
6	-1.077158000	-3.624185000	0.002263000
1	-1.667193000	-3.348372000	-0.883803000
1	-0.989726000	-4.723921000	-0.000378000
1	-1.663638000	-3.352339000	0.891897000
6	1.543862000	-3.192802000	-1.588511000
1	2.505486000	-2.660615000	-1.642030000
1	1.772851000	-4.267574000	-1.677545000
1	0.919553000	-2.932720000	-2.458396000
6	1.545767000	-3.192385000	1.589325000
1	0.922793000	-2.931380000	2.459891000
1	1.774259000	-4.267232000	1.678734000
1	2.507751000	-2.660704000	1.641223000
6	4.680521000	-0.053140000	-0.000141000
1	3.594003000	-0.131283000	-0.000024000
5	7.907014000	0.140410000	-0.000401000
5	6.941746000	0.556162000	-1.459175000
5	6.880232000	1.611920000	-0.000070000
5	6.942014000	0.555684000	1.458700000
5	7.046724000	-1.152849000	0.900598000
5	7.046553000	-1.152539000	-0.901655000
5	5.491585000	-0.473057000	-1.437862000
5	5.393308000	1.214984000	-0.888343000
5	5.393506000	1.214671000	0.888344000
5	5.491847000	-0.473535000	1.437317000
5	5.559819000	-1.514247000	-0.000455000

17	9.673711000	0.248072000	-0.000547000
17	7.652307000	1.098833000	-2.989468000
17	7.523930000	3.263128000	0.000136000
17	7.652820000	1.097845000	2.989057000
17	7.866005000	-2.408850000	1.845128000
17	7.865639000	-2.408245000	-1.846751000
17	4.571272000	-1.017500000	-2.867121000
17	4.388262000	2.398144000	-1.769419000
17	4.388555000	2.397521000	1.769955000
17	4.571830000	-1.018270000	2.866650000
17	4.711704000	-3.080628000	-0.000758000
6	-4.680555000	0.053120000	-0.000078000
1	-3.594039000	0.131288000	0.000062000
5	-7.907036000	-0.140312000	-0.000456000
5	-7.046737000	1.152821000	0.900687000
5	-7.046522000	1.152697000	-0.901562000
5	-6.941739000	-0.555959000	-1.459248000
5	-6.880316000	-1.611869000	-0.000236000
5	-6.942093000	-0.555770000	1.458614000
5	-5.491907000	0.473405000	1.437378000
5	-5.559795000	1.514281000	-0.000279000
5	-5.491552000	0.473222000	-1.437785000
5	-5.393367000	-1.214873000	-0.888424000
5	-5.393569000	-1.214771000	0.888269000
17	-9.673740000	-0.247927000	-0.000658000
17	-7.866004000	2.408749000	1.845331000
17	-7.865546000	2.408514000	-1.846565000
17	-7.652247000	-1.098461000	-2.989627000
17	-7.524063000	-3.263054000	-0.000218000
17	-7.652975000	-1.098057000	2.988897000
17	-4.571893000	1.018027000	2.866760000
17	-4.711577000	3.080605000	-0.000353000
17	-4.571179000	1.017716000	-2.866975000
17	-4.388282000	-2.397955000	-1.769584000
17	-4.388690000	-2.397761000	1.769740000

CB_dicat_CB iso3

14	0.015727000	0.419912000	0.721312000
14	1.254393000	3.408191000	0.191340000
8	0.658647000	1.747717000	-0.294979000
6	-1.485952000	1.031375000	1.546165000
1	-1.185534000	1.592899000	2.446635000
1	-2.113295000	0.187641000	1.882787000
1	-2.124047000	1.664216000	0.917044000
6	1.189360000	-0.532050000	1.751894000
1	1.828647000	-1.231091000	1.197513000
1	0.599732000	-1.073744000	2.508940000
1	1.876626000	0.148682000	2.280185000
6	-0.311666000	4.406935000	0.272142000
1	-0.812430000	4.473993000	-0.705198000
1	-0.066266000	5.434873000	0.585280000
1	-1.030706000	4.004683000	1.001177000
6	2.373575000	3.919906000	-1.193151000
1	3.265749000	3.280678000	-1.262693000
1	2.717802000	4.946549000	-0.985426000
1	1.856124000	3.951991000	-2.165108000
6	2.056026000	3.143794000	1.836360000
1	1.356965000	2.747359000	2.589487000
1	2.396098000	4.124904000	2.207000000
1	2.941355000	2.494149000	1.770906000
14	0.221874000	1.068777000	-1.865687000
14	-0.445861000	-2.100704000	-1.299968000
8	-0.249639000	-0.312671000	-0.881225000
6	1.553214000	0.681132000	-3.066035000
1	1.076899000	0.377173000	-4.012535000
1	2.137682000	1.593695000	-3.266178000
1	2.264153000	-0.083799000	-2.730784000
6	-1.168930000	2.039111000	-2.565351000
1	-1.934704000	2.325027000	-1.832458000
1	-0.752146000	2.943603000	-3.039324000
1	-1.668733000	1.456342000	-3.355172000
6	1.283306000	-2.718521000	-1.543929000
1	1.965585000	-2.501082000	-0.709204000
1	1.235512000	-3.816417000	-1.640178000
1	1.747887000	-2.336739000	-2.464149000
6	-1.305115000	-2.772527000	0.190478000
1	-2.239946000	-2.238395000	0.419248000
1	-1.586601000	-3.817477000	-0.020553000
1	-0.655816000	-2.790656000	1.078814000
6	-1.429196000	-2.091139000	-2.871194000
1	-0.998202000	-1.429371000	-3.639361000
1	-1.406304000	-3.112105000	-3.286701000
1	-2.478092000	-1.817930000	-2.693161000
6	-6.361032000	-0.354790000	-1.447715000
1	-6.172039000	-0.480374000	-2.514560000
5	-6.913861000	0.018181000	1.714657000
5	-8.239528000	-0.367401000	0.567466000
5	-6.979033000	-1.601341000	0.934430000
5	-5.415623000	-0.742419000	1.105228000
5	-5.698269000	1.009500000	0.850310000
5	-7.444664000	1.249084000	0.517134000
5	-7.829809000	0.387285000	-0.996878000
5	-7.544839000	-1.361327000	-0.741096000
5	-5.811550000	-1.588837000	-0.407248000
5	-5.049275000	0.005676000	-0.449842000
5	-6.270013000	1.231594000	-0.820193000

17	-7.193949000	0.223272000	3.453980000
17	-9.923067000	-0.569324000	1.081229000
17	-7.326177000	-3.092512000	1.829833000
17	-4.119195000	-1.362959000	2.179039000
17	-4.701825000	2.267776000	1.639050000
17	-8.285679000	2.740784000	0.977497000
17	-8.978153000	0.923685000	-2.228582000
17	-8.407389000	-2.553412000	-1.720583000
17	-4.918444000	-2.985108000	-1.059789000
17	-3.407136000	0.163551000	-1.149293000
17	-5.827280000	2.592135000	-1.871942000
6	7.343343000	-0.672418000	-1.273705000
1	7.901308000	-0.907998000	-2.181588000
5	5.724397000	0.014574000	1.431779000
5	4.983832000	0.086780000	-0.173658000
5	5.414780000	-1.490143000	0.510951000
5	6.959459000	-1.279235000	1.399140000
5	7.449350000	0.453554000	1.252154000
5	6.203110000	1.295415000	0.274964000
5	6.195072000	0.576232000	-1.352527000
5	5.710740000	-1.135179000	-1.207470000
5	6.944642000	-1.984382000	-0.245112000
5	8.194659000	-0.787852000	0.206124000
5	7.732050000	0.797186000	-0.480434000
17	4.760692000	0.416899000	2.882749000
17	3.281308000	0.586400000	-0.394888000
17	4.162794000	-2.671415000	0.999972000
17	7.363871000	-2.258332000	2.819531000
17	8.366117000	1.282717000	2.521673000
17	5.740263000	3.003423000	0.510674000
17	5.825486000	1.463476000	-2.846208000
17	4.877888000	-1.922354000	-2.568672000
17	7.394576000	-3.639978000	-0.666068000
17	9.892821000	-1.268194000	0.233220000
17	8.960070000	1.885488000	-1.133845000

CB_dicat_CB iso4

14	0.000000000	0.000000000	1.334473000
14	-2.920764000	0.637820000	0.000000000
8	-1.092204000	0.410051000	0.000000000
6	-0.587800000	-1.456936000	2.250925000
1	-1.254450000	-1.137238000	3.067960000
1	0.259254000	-1.990988000	2.711995000
1	-1.128594000	-2.190249000	1.636217000
6	0.587800000	1.456936000	2.250925000
1	1.128594000	2.190249000	1.636217000
1	1.254450000	1.137238000	3.067960000
1	-0.259254000	1.990988000	2.711995000
6	-3.623921000	-1.077460000	0.000000000
1	-3.349897000	-1.665632000	-0.887666000
1	-4.723683000	-0.990250000	0.000000000
1	-3.349897000	-1.665632000	0.887666000
6	-3.192634000	1.544649000	-1.588837000
1	-2.660692000	2.506448000	-1.641537000
1	-4.267437000	1.773418000	-1.678053000
1	-2.932086000	0.921024000	-2.459069000
6	-3.192634000	1.544649000	1.588837000
1	-2.932086000	0.921024000	2.459069000
1	-4.267437000	1.773418000	1.678053000
1	-2.660692000	2.506448000	1.641537000
14	0.000000000	0.000000000	-1.334473000
14	2.920764000	-0.637820000	0.000000000
8	1.092204000	-0.410051000	0.000000000
6	0.587800000	1.456936000	-2.250925000
1	1.254450000	1.137238000	-3.067960000
1	-0.259254000	1.990988000	-2.711995000
1	1.128594000	2.190249000	-1.636217000
6	-0.587800000	-1.456936000	-2.250925000
1	-1.128594000	-2.190249000	-1.636217000
1	-1.254450000	-1.137238000	-3.067960000
1	0.259254000	-1.990988000	-2.711995000
6	3.623921000	1.077460000	0.000000000
1	3.349897000	1.665632000	0.887666000
1	4.723683000	0.990250000	0.000000000
1	3.349897000	1.665632000	-0.887666000
6	3.192634000	-1.544649000	1.588837000
1	2.660692000	-2.506448000	1.641537000
1	4.267437000	-1.773418000	1.678053000
1	2.932086000	-0.921024000	2.459069000
6	3.192634000	-1.544649000	-1.588837000
1	2.932086000	-0.921024000	-2.459069000
1	4.267437000	-1.773418000	-1.678053000
1	2.660692000	-2.506448000	-1.641537000
6	0.053234000	-4.680570000	0.000000000
1	0.131399000	-3.594057000	0.000000000
5	-0.140382000	-7.907016000	0.000000000
5	-0.555892000	-6.941885000	1.459002000
5	-1.611803000	-6.880240000	0.000000000
5	-0.555892000	-6.941885000	-1.459002000
5	1.152723000	-7.046646000	-0.901051000
5	1.152723000	-7.046646000	0.901051000
5	0.473355000	-5.491730000	1.437658000
5	-1.214735000	-5.393427000	0.888360000
5	-1.214735000	-5.393427000	-0.888360000
5	0.473355000	-5.491730000	-1.437658000
5	1.514284000	-5.559805000	0.000000000

17	-0.248091000	-9.673711000	0.000000000
17	-1.098357000	-7.652574000	2.989274000
17	-3.263043000	-7.523912000	0.000000000
17	-1.098357000	-7.652574000	-2.989274000
17	2.408552000	-7.865847000	-1.845936000
17	2.408552000	-7.865847000	1.845936000
17	1.017985000	-4.571514000	2.866864000
17	-2.397809000	-4.388433000	1.769626000
17	-2.397809000	-4.388433000	-1.769626000
17	1.017985000	-4.571514000	-2.866864000
17	3.080631000	-4.711619000	0.000000000
6	-0.053234000	4.680570000	0.000000000
1	-0.131399000	3.594057000	0.000000000
5	0.140382000	7.907016000	0.000000000
5	-1.152723000	7.046646000	-0.901051000
5	-1.152723000	7.046646000	0.901051000
5	0.555892000	6.941885000	1.459002000
5	1.611803000	6.880240000	0.000000000
5	0.555892000	6.941885000	-1.459002000
5	-0.473355000	5.491730000	-1.437658000
5	-1.514284000	5.559805000	0.000000000
5	-0.473355000	5.491730000	1.437658000
5	1.214735000	5.393427000	0.888360000
5	1.214735000	5.393427000	-0.888360000
17	0.248091000	9.673711000	0.000000000
17	-2.408552000	7.865847000	-1.845936000
17	-2.408552000	7.865847000	1.845936000
17	1.098357000	7.652574000	2.989274000
17	3.263043000	7.523912000	0.000000000
17	1.098357000	7.652574000	-2.989274000
17	-1.017985000	4.571514000	-2.866864000
17	-3.080631000	4.711619000	0.000000000
17	-1.017985000	4.571514000	2.866864000
17	2.397809000	4.388433000	1.769626000
17	2.397809000	4.388433000	-1.769626000

CB_dicat_CB iso5

14	-0.063821000	0.669374000	0.787239000
14	1.331164000	3.465402000	-0.206532000
8	0.678261000	1.772169000	-0.413234000
6	-1.542152000	1.502317000	1.453045000
1	-1.212636000	2.199768000	2.242063000
1	-2.220091000	0.769667000	1.923440000
1	-2.135762000	2.041539000	0.704921000
6	1.027458000	-0.133027000	2.021121000
1	1.671785000	-0.924874000	1.618038000
1	0.381705000	-0.534781000	2.818460000
1	1.701634000	0.611978000	2.473143000
6	-0.190227000	4.526031000	-0.350553000
1	-0.672003000	4.435333000	-1.335618000
1	0.097926000	5.582293000	-0.223113000
1	-0.937503000	4.293756000	0.422742000
6	2.506197000	3.689310000	-1.619805000
1	3.366723000	3.006782000	-1.563671000
1	2.898011000	4.718783000	-1.572611000
1	2.009461000	3.585084000	-2.597515000
6	2.067800000	3.476710000	1.491659000
1	1.325273000	3.248057000	2.272410000
1	2.433466000	4.497252000	1.692551000
1	2.927344000	2.796470000	1.581026000
14	0.290474000	0.829565000	-1.860095000
14	-0.560403000	-2.156998000	-0.778271000
8	-0.287978000	-0.327857000	-0.665533000
6	1.661458000	0.190338000	-2.891514000
1	1.227926000	-0.209393000	-3.822741000
1	2.338251000	1.016770000	-3.162744000
1	2.283311000	-0.570104000	-2.402609000
6	-1.036285000	1.699856000	-2.781557000
1	-1.804350000	2.146544000	-2.136827000
1	-0.571970000	2.481723000	-3.405269000
1	-1.544660000	0.995794000	-3.459498000
6	1.145640000	-2.871304000	-0.901145000
1	1.850900000	-2.502627000	-0.141795000
1	1.061837000	-3.961818000	-0.757092000
1	1.603697000	-2.713634000	-1.887551000
6	-1.417251000	-2.527825000	0.815502000
1	-2.281246000	-1.872056000	1.001596000
1	-1.825414000	-3.549635000	0.752106000
1	-0.728325000	-2.506129000	1.673047000
6	-1.532039000	-2.385416000	-2.338417000
1	-1.042369000	-1.914032000	-3.205899000
1	-1.566286000	-3.467569000	-2.547577000
1	-2.564895000	-2.022162000	-2.253009000
6	-7.700367000	0.150389000	-1.061725000
1	-8.462609000	0.335167000	-1.820510000
5	-5.473991000	-0.396849000	1.194466000
5	-6.986536000	-1.345136000	1.146186000
5	-5.750938000	-1.554547000	-0.139962000
5	-5.044894000	0.046358000	-0.470268000
5	-5.801367000	1.256021000	0.593068000
5	-7.018266000	0.401842000	1.601479000
5	-8.252260000	-0.267572000	0.501484000
5	-7.474420000	-1.471194000	-0.566066000
5	-6.267104000	-0.616141000	-1.559419000
5	-6.299059000	1.112271000	-1.110541000
5	-7.525897000	1.330214000	0.163807000

17	-4.226972000	-0.715016000	2.445055000
17	-7.346107000	-2.629833000	2.314633000
17	-4.794109000	-3.049267000	-0.341774000
17	-3.347130000	0.214672000	-1.030773000
17	-4.898024000	2.688439000	1.163874000
17	-7.412304000	0.939469000	3.245162000
17	-9.979563000	-0.389001000	0.849552000
17	-8.420437000	-2.784204000	-1.275767000
17	-5.963379000	-1.084134000	-3.242053000
17	-6.038460000	2.348505000	-2.353916000
17	-8.523964000	2.788689000	0.178242000
6	7.982727000	-0.781279000	0.397377000
1	9.027980000	-1.071457000	0.518991000
5	4.950817000	0.057433000	0.046181000
5	5.619316000	-1.277464000	-0.900868000
5	5.365807000	-1.449500000	0.870962000
5	5.715835000	0.137593000	1.638992000
5	6.187266000	1.289043000	0.341299000
5	6.124651000	0.415572000	-1.227922000
5	7.331215000	-0.889500000	-1.178016000
5	6.862096000	-2.039477000	0.115457000
5	6.922745000	-1.167192000	1.681228000
5	7.428274000	0.521166000	1.354167000
5	7.680599000	0.692536000	-0.412685000
17	3.244791000	0.555246000	-0.144078000
17	4.636237000	-2.191283000	-2.079968000
17	4.112484000	-2.539382000	1.532519000
17	4.815872000	0.693499000	3.076375000
17	5.770193000	3.020717000	0.448542000
17	5.643917000	1.254653000	-2.728358000
17	8.304190000	-1.446817000	-2.542890000
17	7.371547000	-3.728321000	0.023694000
17	7.493632000	-1.997303000	3.131808000
17	8.498444000	1.356372000	2.484081000
17	8.998532000	1.697608000	-1.023408000

CB_dicat_CB iso6

14	-0.013172000	0.260847000	-1.103949000
14	-0.780820000	3.101413000	0.334801000
8	-0.388469000	1.316376000	0.283748000
6	1.508728000	0.891117000	-1.887503000
1	1.224888000	1.657491000	-2.628558000
1	2.029404000	0.087267000	-2.430871000
1	2.233834000	1.316606000	-1.180178000
6	-1.366464000	-0.219282000	-2.238898000
1	-2.081426000	-0.942597000	-1.825841000
1	-0.905402000	-0.621465000	-3.155559000
1	-1.960797000	0.664490000	-2.521366000
6	0.885004000	3.918403000	0.382202000
1	1.465163000	3.636535000	1.273306000
1	0.743961000	5.011291000	0.413029000
1	1.493938000	3.689548000	-0.504860000
6	-1.741845000	3.323260000	1.904013000
1	-2.702653000	2.788855000	1.888394000
1	-1.959917000	4.398783000	2.011406000
1	-1.163858000	3.030227000	2.794809000
6	-1.708696000	3.407360000	-1.238018000
1	-1.110492000	3.163128000	-2.130111000
1	-1.929230000	4.486140000	-1.294439000
1	-2.667787000	2.870187000	-1.276557000
14	0.037607000	0.169469000	1.570752000
14	0.225156000	-2.766788000	0.144226000
8	0.228994000	-0.927490000	0.196166000
6	-1.290217000	-0.365825000	2.717043000
1	-0.818435000	-0.921381000	3.543807000
1	-1.778504000	0.521971000	3.150027000
1	-2.084009000	-0.966827000	2.255759000
6	1.593669000	0.717991000	2.351970000
1	2.304833000	1.171514000	1.647119000
1	1.350902000	1.443035000	3.146665000
1	2.108176000	-0.132782000	2.825323000
6	-1.556514000	-3.253481000	0.237658000
1	-2.189507000	-2.813484000	-0.546170000
1	-1.614181000	-4.348857000	0.121905000
1	-2.018044000	-3.007891000	1.204321000
6	1.027064000	-3.142250000	-1.484259000
1	2.070472000	-2.797221000	-1.528817000
1	1.029402000	-4.236663000	-1.617904000
1	0.459431000	-2.725677000	-2.331012000
6	1.177167000	-3.247086000	1.662763000
1	0.717135000	-2.848021000	2.580797000
1	1.137702000	-4.345726000	1.748452000
1	2.233871000	-2.947225000	1.620275000
6	7.428508000	-0.380243000	1.349234000
1	7.999550000	-0.585199000	2.256352000
5	5.761404000	0.234210000	-1.340742000
5	7.482214000	-0.244316000	-1.408579000
5	6.234634000	-1.436319000	-0.899911000
5	5.030748000	-0.557161000	0.072941000
5	5.484692000	1.155205000	0.167234000
5	7.010918000	1.368270000	-0.747250000
5	8.259103000	0.388064000	0.067577000
5	7.783613000	-1.335010000	-0.027298000
5	6.262971000	-1.528954000	0.879851000
5	5.802716000	0.066179000	1.533800000
5	7.031327000	1.254078000	1.033374000

17	4.818568000	0.564373000	-2.820383000
17	8.369197000	-0.408892000	-2.934170000
17	5.759569000	-2.842848000	-1.880471000
17	3.313938000	-1.076731000	0.101633000
17	4.235052000	2.441500000	0.291471000
17	7.397166000	2.887459000	-1.577959000
17	9.962894000	0.823883000	0.228529000
17	9.012459000	-2.602002000	0.042851000
17	5.927420000	-2.983701000	1.836409000
17	5.034249000	0.176038000	3.134806000
17	7.502560000	2.544816000	2.146751000
6	-7.442699000	1.003403000	0.793902000
1	-8.079773000	1.690230000	1.353810000
5	-5.590012000	-1.041985000	-0.874669000
5	-7.111529000	-1.588677000	-0.112103000
5	-7.150962000	-0.468208000	-1.530271000
5	-5.771861000	0.669783000	-1.368726000
5	-4.921005000	0.251472000	0.130214000
5	-5.708696000	-1.131760000	0.910986000
5	-7.347004000	-0.609016000	1.364943000
5	-8.231343000	-0.197696000	-0.134356000
5	-7.409891000	1.188230000	-0.910554000
5	-6.020304000	1.626651000	0.109796000
5	-5.979808000	0.521463000	1.510649000
17	-4.527638000	-2.151563000	-1.794451000
17	-7.690479000	-3.260651000	-0.217622000
17	-7.770150000	-0.974095000	-3.111835000
17	-4.883295000	1.366500000	-2.754013000
17	-3.164710000	0.538739000	0.297272000
17	-4.769046000	-2.299680000	1.884804000
17	-8.223457000	-1.114417000	2.813059000
17	-9.993156000	-0.301352000	-0.166701000
17	-8.347578000	2.456360000	-1.706115000
17	-5.504623000	3.314144000	0.316957000
17	-5.424675000	1.134517000	3.082153000

CB_dicat (+)

14	3.834577000	0.155867000	-1.330755000
14	2.982029000	3.056437000	-0.000912000
8	3.468118000	1.269581000	-0.001696000
6	5.494989000	0.617559000	-1.972404000
1	5.400690000	1.502754000	-2.623187000
1	5.903011000	-0.194334000	-2.594577000
1	6.213407000	0.849139000	-1.174352000
6	2.571850000	-0.284384000	-2.579005000
1	1.816902000	-0.995201000	-2.212979000
1	3.090771000	-0.689729000	-3.462731000
1	2.008666000	0.609172000	-2.893198000
6	4.644306000	3.891455000	0.025318000
1	5.225565000	3.640618000	0.925627000
1	4.495349000	4.983853000	0.030989000
1	5.249448000	3.653151000	-0.862622000
6	2.030853000	3.273486000	1.567596000
1	1.077356000	2.724671000	1.564928000
1	1.788042000	4.345701000	1.658269000
1	2.617149000	3.010279000	2.462430000
6	2.071664000	3.291296000	-1.591357000
1	2.687381000	3.051794000	-2.472882000
1	1.818710000	4.362179000	-1.669139000
1	1.125568000	2.730932000	-1.625186000
14	3.770183000	0.146035000	1.338261000
14	4.050243000	-2.835070000	0.000031000
8	3.984187000	-1.001266000	0.001873000
6	2.450094000	-0.309098000	2.517408000
1	2.922385000	-0.635718000	3.457574000
1	1.792330000	0.547729000	2.735425000
1	1.780615000	-1.091925000	2.130958000
6	5.398444000	0.606980000	2.058124000
1	6.158182000	0.835082000	1.298361000
1	5.270113000	1.494873000	2.699516000
1	5.772138000	-0.201423000	2.705839000
6	2.315566000	-3.422669000	-0.170029000
1	1.866500000	-3.157197000	-1.137668000
1	2.312878000	-4.523440000	-0.099258000
1	1.640351000	-3.042858000	0.611790000
6	5.139058000	-3.162638000	-1.467182000
1	6.133456000	-2.702803000	-1.364375000
1	5.287639000	-4.250655000	-1.564698000
1	4.677299000	-2.828442000	-2.410035000
6	4.847032000	-3.174613000	1.641743000
1	4.219445000	-2.848235000	2.486005000
1	4.975062000	-4.263824000	1.754186000
1	5.844887000	-2.718757000	1.727449000
6	-4.101629000	-0.313899000	0.028829000
1	-5.184339000	-0.456538000	0.048392000
5	-0.971610000	0.096625000	-0.027102000
5	-1.702189000	-1.241005000	-0.915079000
5	-1.934112000	0.454977000	-1.463938000
5	-2.048421000	1.499200000	-0.005839000
5	-1.882171000	0.450617000	1.443408000
5	-1.670086000	-1.242830000	0.881934000
5	-3.140465000	-1.727596000	0.009022000
5	-3.305120000	-0.677615000	-1.438562000
5	-3.518156000	1.013099000	-0.878201000
5	-3.485883000	1.010060000	0.917415000
5	-3.252115000	-0.682151000	1.465340000

17	0.801805000	0.362562000	-0.068029000
17	-0.723641000	-2.385233000	-1.882035000
17	-1.163677000	1.054862000	-2.958681000
17	-1.405494000	3.164144000	-0.014199000
17	-1.069515000	1.050782000	2.918313000
17	-0.662365000	-2.397508000	1.811409000
17	-3.854406000	-3.339554000	0.021287000
17	-4.181000000	-1.260836000	-2.852773000
17	-4.606064000	2.099435000	-1.739724000
17	-4.540663000	2.093123000	1.823440000
17	-4.074130000	-1.268889000	2.910329000

CB_dicat(+) iso1

14	3.727182000	0.026098000	-1.423140000
14	3.035727000	3.038555000	-0.273984000
8	3.545394000	1.260368000	-0.155355000
6	5.291443000	0.411974000	-2.311003000
1	5.113318000	1.248812000	-3.006871000
1	5.600079000	-0.449419000	-2.924087000
1	6.118039000	0.687002000	-1.642406000
6	2.320961000	-0.510064000	-2.454596000
1	1.710461000	-1.298507000	-1.988744000
1	2.719409000	-0.858431000	-3.420730000
1	1.615278000	0.315490000	-2.644203000
6	4.678544000	3.884306000	-0.483744000
1	5.342977000	3.735457000	0.380797000
1	4.510243000	4.969747000	-0.578720000
1	5.203803000	3.557958000	-1.394101000
6	2.225283000	3.396573000	1.347794000
1	1.307367000	2.808138000	1.491947000
1	1.929788000	4.459282000	1.340407000
1	2.905676000	3.266441000	2.204024000
6	1.972944000	3.103187000	-1.781623000
1	2.505475000	2.798096000	-2.696495000
1	1.676450000	4.155416000	-1.929327000
1	1.043924000	2.523295000	-1.673209000
14	4.073066000	0.268632000	1.208710000
14	4.067416000	-2.836462000	0.145823000
8	4.061826000	-1.006231000	-0.016126000
6	2.958154000	-0.035050000	2.629047000
1	3.559661000	-0.454344000	3.451861000
1	2.527516000	0.916706000	2.979399000
1	2.110174000	-0.691238000	2.393359000
6	5.795651000	0.755586000	1.625311000
1	6.416879000	0.937040000	0.737962000
1	5.780433000	1.674361000	2.234408000
1	6.271291000	-0.026929000	2.237365000
6	2.359295000	-3.314689000	0.637479000
1	1.584261000	-2.954476000	-0.056904000
1	2.295932000	-4.415785000	0.652974000
1	2.093382000	-2.961165000	1.643658000
6	4.585581000	-3.363949000	-1.555540000
1	5.560511000	-2.945496000	-1.848065000
1	4.688393000	-4.461742000	-1.564384000
1	3.832584000	-3.117255000	-2.320103000
6	5.359688000	-3.084076000	1.455854000
1	5.056171000	-2.658010000	2.425547000
1	5.496561000	-4.165226000	1.622389000
1	6.337273000	-2.669934000	1.166344000
6	-3.170961000	-0.586991000	1.527284000
1	-3.634318000	-0.908741000	2.462102000
5	-1.837364000	0.360454000	-1.250345000
5	-0.893925000	0.058554000	0.213048000
5	-1.630709000	-1.307233000	-0.634161000
5	-3.229985000	-0.760723000	-1.235975000
5	-3.441398000	0.968083000	-0.746385000
5	-1.968760000	1.467945000	0.149520000
5	-1.854220000	0.480675000	1.624786000
5	-1.648560000	-1.226761000	1.142993000
5	-3.105656000	-1.739593000	0.257887000
5	-4.217370000	-0.338618000	0.193457000
5	-3.443950000	1.035315000	1.042420000

17	-1.033412000	0.900994000	-2.756664000
17	0.886900000	0.290119000	0.259825000
17	-0.629401000	-2.524096000	-1.491390000
17	-3.941624000	-1.418935000	-2.715959000
17	-4.374989000	2.113263000	-1.718728000
17	-1.296463000	3.122097000	0.109805000
17	-1.154092000	1.042144000	3.157056000
17	-0.772184000	-2.330487000	2.229550000
17	-3.725188000	-3.376533000	0.475280000
17	-5.952473000	-0.596100000	0.347325000
17	-4.400263000	2.134210000	2.034966000

CB_dicat(+) iso2

14	3.693156000	0.035347000	-1.375845000
14	3.586939000	2.929575000	0.198456000
8	3.682087000	1.092636000	0.054684000
6	5.363044000	0.167071000	-2.139455000
1	5.419404000	1.083574000	-2.749599000
1	5.527111000	-0.678650000	-2.826443000
1	6.178868000	0.185829000	-1.404387000
6	2.264561000	0.016755000	-2.502543000
1	1.451844000	-0.633549000	-2.142487000
1	2.607155000	-0.321355000	-3.492999000
1	1.821352000	1.018259000	-2.607265000
6	5.379090000	3.412983000	0.116231000
1	5.967622000	2.985999000	0.942275000
1	5.461088000	4.509707000	0.191283000
1	5.843747000	3.117548000	-0.836695000
6	2.803015000	3.231520000	1.847392000
1	1.770646000	2.852653000	1.890630000
1	2.762464000	4.323713000	1.997059000
1	3.388907000	2.823819000	2.685833000
6	2.602496000	3.469734000	-1.271348000
1	3.064502000	3.188228000	-2.230438000
1	2.567538000	4.572281000	-1.254216000
1	1.564557000	3.105869000	-1.235317000
14	3.891050000	-0.181025000	1.271231000
14	3.599411000	-3.067157000	-0.310166000
8	3.717130000	-1.235242000	-0.137586000
6	2.653556000	-0.421593000	2.590371000
1	3.101319000	-1.077487000	3.354976000
1	2.421243000	0.541271000	3.072330000
1	1.699659000	-0.842543000	2.238953000
6	5.649681000	-0.129767000	1.810522000
1	6.354379000	-0.023628000	0.974831000
1	5.795807000	0.717794000	2.500503000
1	5.903545000	-1.042520000	2.372083000
6	2.267604000	-3.578733000	0.853283000
1	1.317027000	-3.063410000	0.643051000
1	2.090642000	-4.658770000	0.715139000
1	2.533735000	-3.428791000	1.909878000
6	3.204661000	-3.290651000	-2.106197000
1	3.938141000	-2.827870000	-2.784780000
1	3.227466000	-4.373548000	-2.314875000
1	2.192480000	-2.938346000	-2.353249000
6	5.312969000	-3.609256000	0.157895000
1	5.548614000	-3.388445000	1.210222000
1	5.389324000	-4.702480000	0.037807000
1	6.084109000	-3.153553000	-0.481617000
6	-3.450714000	1.174957000	0.830209000
1	-4.102517000	1.892225000	1.333007000
5	-1.560392000	-0.957296000	-0.665497000
5	-3.039897000	-1.483739000	0.184940000
5	-3.154868000	-0.519544000	-1.340252000
5	-1.820287000	0.681756000	-1.332265000
5	-0.919829000	0.454254000	0.179178000
5	-1.637948000	-0.867455000	1.116120000
5	-3.281997000	-0.364534000	1.561155000
5	-4.215183000	-0.145945000	0.049757000
5	-3.466729000	1.182865000	-0.885448000
5	-2.070048000	1.778767000	0.047678000
5	-1.953277000	0.824862000	1.554683000

17	-0.458686000	-2.138099000	-1.462348000
17	-3.534602000	-3.181542000	0.267382000
17	-3.775481000	-1.213354000	-2.845159000
17	-1.012638000	1.268704000	-2.813565000
17	0.836174000	0.829690000	0.265968000
17	-0.644398000	-1.919069000	2.172040000
17	-4.093520000	-0.757457000	3.076106000
17	-5.967185000	-0.320979000	0.073097000
17	-4.464015000	2.324022000	-1.785008000
17	-1.603759000	3.489733000	0.063422000
17	-1.374290000	1.604501000	3.039115000

B(C6F5)4–

5	0.000004000	0.000026000	0.000015000
6	-1.044664000	0.582297000	1.134435000
6	1.044451000	1.134548000	-0.582456000
6	-1.044628000	-0.582563000	-1.134271000
6	1.044860000	-1.134185000	0.582353000
6	-1.318259000	1.922856000	1.397845000
6	-1.854777000	-0.325202000	1.819861000
6	1.317406000	1.398351000	-1.923069000
6	1.855019000	1.819675000	0.324859000
6	-1.317522000	-1.923204000	-1.397982000
6	-1.855412000	0.324663000	-1.819260000
6	1.855217000	-1.819370000	-0.325106000
6	1.318338000	-1.397669000	1.922922000
6	-2.296018000	2.339141000	2.303622000
9	-0.658876000	2.904896000	0.786497000
6	-2.837980000	0.043603000	2.731191000
9	-1.700167000	-1.634078000	1.617622000
6	2.295004000	2.304212000	-2.339548000
9	0.657524000	0.787325000	-2.904974000
6	2.838085000	2.731074000	-0.044142000
9	1.700979000	1.617105000	1.633753000
6	-2.295247000	-2.339794000	-2.303656000
9	-0.657424000	-2.905035000	-0.787068000
6	-2.838618000	-0.044448000	-2.730464000
9	-1.701492000	1.633572000	-1.616709000
6	2.838553000	-2.730545000	0.043735000
9	1.700706000	-1.617073000	-1.633985000
6	2.296218000	-2.303298000	2.339243000
9	0.658745000	-0.786508000	2.904936000
6	-3.061941000	1.395115000	2.974324000
9	-2.508848000	3.633731000	2.521485000
9	-3.567283000	-0.871618000	3.362407000
6	3.061416000	2.974594000	-1.395687000
9	2.507229000	2.522454000	-3.634173000
9	3.567842000	3.361992000	0.870924000
6	-3.061868000	-1.396019000	-2.973918000
9	-2.507389000	-3.634445000	-2.521826000
9	-3.568584000	0.870538000	-3.361258000
6	3.062392000	-2.973764000	1.395250000
9	3.568084000	-3.361536000	-0.871461000
9	2.508939000	-2.521228000	3.633840000
9	-3.997752000	1.776408000	3.836861000
9	3.997083000	3.837201000	-1.777173000
9	-3.997659000	-1.777608000	-3.836344000
9	3.998325000	-3.836152000	1.776580000

[Me_2Si=O-T][B(C6F5)4]

5	1.405395000	0.029610000	-0.011724000
6	2.429160000	-1.150660000	-0.501748000
6	0.512142000	0.688678000	-1.239228000
6	2.385831000	1.086841000	0.761241000
6	0.158672000	-0.524203000	0.933256000
6	2.812464000	-1.408598000	-1.815099000
6	3.105037000	-1.890675000	0.469144000
6	0.386413000	2.046082000	-1.535335000
6	-0.359770000	-0.140490000	-1.948016000
6	2.428678000	1.308814000	2.134078000
6	3.382306000	1.729575000	0.025235000
6	-0.759083000	0.417484000	1.411186000
6	-0.216064000	-1.861240000	1.076090000
6	3.781978000	-2.355005000	-2.151586000
9	2.269003000	-0.751918000	-2.837912000
6	4.074056000	-2.844248000	0.181615000
9	2.804438000	-1.709586000	1.757464000
6	-0.517917000	2.544026000	-2.477678000
9	1.100913000	2.967292000	-0.902093000
6	-1.289165000	0.313758000	-2.873235000
9	-0.367815000	-1.460200000	-1.719483000
6	3.371293000	2.136052000	2.744841000
9	1.554268000	0.722586000	2.956759000
6	4.341013000	2.563507000	0.589295000
9	3.435258000	1.560580000	-1.295098000
6	-2.003882000	0.090196000	1.936663000
9	-0.506867000	1.713716000	1.306977000
6	-1.451488000	-2.247138000	1.597393000
9	0.537094000	-2.858879000	0.649936000
6	4.418305000	-3.075234000	-1.148030000
9	4.105633000	-2.565916000	-3.420096000
9	4.669158000	-3.529207000	1.148676000
6	-1.367022000	1.673955000	-3.146902000
9	-0.607249000	3.847138000	-2.701134000
9	-2.163058000	-0.526317000	-3.429710000
6	4.335172000	2.766214000	1.967280000
9	3.359797000	2.317097000	4.059043000
9	5.256389000	3.158543000	-0.161877000
6	-2.327194000	-1.254871000	1.991406000
9	-2.880684000	1.012148000	2.307421000
9	-1.838705000	-3.516533000	1.625641000
9	5.339396000	-3.976300000	-1.451656000
9	-2.284689000	2.135453000	-3.984982000
9	5.238502000	3.553411000	2.529941000
9	-3.641613000	-1.625859000	2.299427000
14	-4.920257000	-1.760997000	0.789718000
6	-3.847339000	-2.410284000	-0.550459000
1	-4.498229000	-2.603859000	-1.420620000
1	-3.071814000	-1.700158000	-0.871812000
1	-3.367506000	-3.358082000	-0.267629000
6	-6.067238000	-2.885429000	1.673895000
1	-6.489225000	-2.391090000	2.560867000
1	-6.899385000	-3.153492000	1.003214000
1	-5.558392000	-3.812194000	1.977419000
8	-5.379213000	-0.238056000	0.695661000
14	-5.434475000	1.320497000	-0.048603000
6	-5.709539000	2.530084000	1.335062000
1	-5.788776000	3.552634000	0.933242000
1	-6.639883000	2.306431000	1.878955000

1	-4.872977000	2.510003000	2.048258000
6	-3.828907000	1.560439000	-0.956982000
1	-3.685946000	0.785645000	-1.724880000
1	-3.831120000	2.527837000	-1.484243000
1	-2.959202000	1.559492000	-0.283740000
6	-6.881467000	1.231112000	-1.223225000
1	-7.023935000	2.200502000	-1.726921000
1	-6.716292000	0.474709000	-2.006482000
1	-7.815528000	0.987345000	-0.694018000

[T][B(C6F5)4]

5	0.838275000	0.038193000	-0.006297000
6	1.640125000	-1.355184000	-0.310334000
6	0.042661000	0.653904000	-1.322125000
6	1.992980000	1.012203000	0.617874000
6	-0.476715000	-0.150007000	0.987525000
6	1.976182000	-1.848637000	-1.568021000
6	2.173729000	-2.062757000	0.767305000
6	0.117293000	1.967111000	-1.787432000
6	-0.942799000	-0.121304000	-1.937520000
6	2.099053000	1.400624000	1.949760000
6	3.064726000	1.381795000	-0.195784000
6	-1.196038000	1.003458000	1.325436000
6	-1.098929000	-1.366476000	1.273294000
6	2.767987000	-2.983598000	-1.751136000
9	1.555808000	-1.252385000	-2.681603000
6	2.963231000	-3.198487000	0.633495000
9	1.906653000	-1.658101000	2.011482000
6	-0.704496000	2.468889000	-2.800742000
9	0.964463000	2.843546000	-1.264413000
6	-1.794749000	0.340758000	-2.932888000
9	-1.144377000	-1.386932000	-1.546479000
6	3.174902000	2.136177000	2.446545000
9	1.157766000	1.076615000	2.840865000
6	4.156405000	2.115891000	0.253906000
9	3.062578000	1.032237000	-1.481396000
6	-2.493188000	0.980158000	1.822928000
9	-0.692290000	2.201320000	1.080692000
6	-2.398081000	-1.447548000	1.776989000
9	-0.542238000	-2.530805000	0.994625000
6	3.266606000	-3.660846000	-0.644892000
9	3.053746000	-3.415505000	-2.971669000
9	3.427891000	-3.839199000	1.697411000
6	-1.674598000	1.655143000	-3.372848000
9	-0.585129000	3.725725000	-3.200521000
9	-2.743065000	-0.447240000	-3.437616000
6	4.211210000	2.495106000	1.593024000
9	3.219996000	2.486880000	3.725083000
9	5.140300000	2.450795000	-0.568167000
6	-3.060142000	-0.264664000	2.035186000
9	-3.219815000	2.076789000	1.996000000
9	-3.036862000	-2.603835000	1.903369000
9	4.018481000	-4.738777000	-0.803105000
9	-2.478763000	2.121908000	-4.315922000
9	5.240264000	3.192506000	2.047622000
9	-4.446928000	-0.323666000	2.267269000
14	-5.623613000	-0.391400000	0.703978000
6	-4.436725000	-0.210563000	-0.690494000
1	-5.018605000	-0.262282000	-1.628056000
1	-3.921186000	0.761040000	-0.682128000
1	-3.683565000	-1.010912000	-0.738103000
6	-6.326934000	-2.065986000	1.003872000
1	-6.905989000	-2.107278000	1.937851000
1	-6.999909000	-2.329416000	0.170849000
1	-5.523993000	-2.817379000	1.042274000
6	-6.639325000	1.084295000	1.130502000
1	-5.994297000	1.968254000	1.249253000
1	-7.346141000	1.289197000	0.309233000
1	-7.219673000	0.933798000	2.052314000

TS3

6	3.715399000	-0.235282000	0.272329000
1	4.794080000	-0.310797000	0.419587000
5	0.561392000	-0.018295000	-0.158221000
5	1.568779000	1.436300000	-0.015681000
5	1.687694000	0.396851000	-1.471494000
5	1.496175000	-1.309900000	-0.946108000
5	1.257998000	-1.318372000	0.837897000
5	1.290590000	0.385797000	1.409988000
5	2.912310000	1.038895000	1.071423000
5	3.156299000	1.044816000	-0.702443000
5	3.114379000	-0.647198000	-1.274679000
5	2.848144000	-1.699282000	0.145580000
5	2.722118000	-0.657314000	1.596538000
17	-1.219717000	0.047897000	-0.387783000
17	0.923227000	3.105982000	-0.105580000
17	1.126562000	0.957404000	-3.068257000
17	0.719884000	-2.520579000	-1.991860000
17	0.262982000	-2.563674000	1.629376000
17	0.304232000	0.942893000	2.789896000
17	3.785766000	2.194809000	2.085648000
17	4.269303000	2.207603000	-1.434635000
17	4.197465000	-1.164915000	-2.571748000
17	3.665743000	-3.262164000	0.253374000
17	3.415828000	-1.188570000	3.133058000
8	-4.262333000	-0.551433000	-0.024393000
14	-4.229596000	-0.288628000	1.624991000
14	-4.255738000	-1.785253000	-1.177199000
6	-3.135491000	-3.185642000	-0.657886000
1	-3.504902000	-3.700548000	0.241924000
1	-3.081064000	-3.930921000	-1.467998000
1	-2.111333000	-2.834869000	-0.460811000
6	-6.028694000	-2.373045000	-1.352465000
1	-6.114100000	-3.158539000	-2.120083000
1	-6.400502000	-2.791640000	-0.403586000
1	-6.695522000	-1.544775000	-1.639619000
6	-3.657851000	-1.021594000	-2.781447000
1	-4.212247000	-0.100608000	-3.024187000
1	-2.582293000	-0.792544000	-2.727478000
1	-3.806667000	-1.725125000	-3.615994000
6	-3.243422000	-1.570324000	2.542572000
1	-3.695176000	-2.569602000	2.449516000
1	-2.208760000	-1.622014000	2.172905000
1	-3.207421000	-1.316933000	3.614375000
6	-5.993082000	-0.194077000	2.249131000
1	-6.493237000	-1.170371000	2.149853000
1	-6.026934000	0.093154000	3.312228000
1	-6.578013000	0.542084000	1.675582000
6	-3.419979000	1.418224000	1.817883000
1	-2.696894000	1.505826000	0.971352000
1	-4.151097000	2.237469000	1.827562000
1	-2.775454000	1.509529000	2.704982000
14	-2.944957000	2.931403000	-0.669245000
6	-4.743174000	2.562987000	-0.840920000
1	-4.871829000	1.465754000	-0.798640000
1	-5.366222000	3.032793000	-0.068568000
1	-5.066815000	2.902598000	-1.839098000
6	-1.985235000	2.614953000	-2.200191000
1	-0.897865000	2.618545000	-2.048458000

1	-2.288298000	1.681769000	-2.693454000
1	-2.253203000	3.461931000	-2.863805000
6	-2.316463000	4.211179000	0.497471000
1	-2.029016000	5.098739000	-0.092741000
1	-3.059798000	4.491563000	1.255127000
1	-1.399791000	3.844256000	0.986851000

TS4

6	3.643441000	-0.506454000	0.279535000
1	4.700826000	-0.740380000	0.415499000
5	0.571150000	0.189247000	-0.119479000
5	1.763338000	1.486240000	0.044954000
5	1.722588000	0.456147000	-1.442605000
5	1.288499000	-1.216225000	-0.939549000
5	1.059586000	-1.225511000	0.849122000
5	1.346710000	0.445222000	1.456163000
5	3.042770000	0.854045000	1.109898000
5	3.270996000	0.864730000	-0.666217000
5	2.983065000	-0.792672000	-1.266188000
5	2.570667000	-1.824446000	0.133930000
5	2.609448000	-0.804767000	1.604890000
17	-1.271282000	0.294274000	-0.268669000
17	1.417875000	3.235863000	0.050022000
17	1.280679000	1.070984000	-3.050820000
17	0.335735000	-2.278378000	-1.999242000
17	-0.103715000	-2.330656000	1.614999000
17	0.476398000	1.107877000	2.859285000
17	4.079769000	1.848461000	2.135102000
17	4.535760000	1.865169000	-1.383790000
17	3.961874000	-1.436927000	-2.585665000
17	3.140001000	-3.493293000	0.202936000
17	3.217560000	-1.463343000	3.124924000
8	-3.956235000	-0.080080000	-0.064611000
14	-4.532689000	-0.148071000	1.535071000
14	-4.068537000	-1.262552000	-1.292737000
6	-3.276399000	-2.848455000	-0.700996000
1	-3.797429000	-3.280595000	0.165648000
1	-3.292520000	-3.598536000	-1.507756000
1	-2.224990000	-2.684650000	-0.421954000
6	-5.876049000	-1.501625000	-1.735713000
1	-5.956633000	-2.134998000	-2.633718000
1	-6.448740000	-1.987867000	-0.933698000
1	-6.355689000	-0.536545000	-1.964503000
6	-3.186365000	-0.690476000	-2.849342000
1	-3.617290000	0.217986000	-3.294923000
1	-2.103598000	-0.551869000	-2.724421000
1	-3.316277000	-1.496189000	-3.591224000
6	-5.769449000	-1.538801000	1.780595000
1	-6.696648000	-1.376989000	1.211069000
1	-5.369510000	-2.529927000	1.521997000
1	-6.038971000	-1.564775000	2.849240000
6	-5.447538000	1.448844000	1.914284000
1	-6.324956000	1.571336000	1.259278000
1	-5.818960000	1.398059000	2.950731000
1	-4.830155000	2.354545000	1.834018000
6	-3.091328000	-0.399778000	2.691433000
1	-2.309606000	0.367711000	2.601653000
1	-3.443971000	-0.404022000	3.735296000
1	-2.602924000	-1.365579000	2.494348000
14	-2.414686000	2.319952000	-0.676878000
6	-4.172366000	2.390323000	-1.276805000
1	-4.919834000	2.114066000	-0.530329000
1	-4.289977000	3.457956000	-1.540843000
1	-4.333803000	1.804919000	-2.189348000
6	-1.374038000	2.982798000	-2.059660000
1	-0.307286000	3.064429000	-1.825585000
1	-1.492400000	2.368920000	-2.964626000

1	-1.762580000	3.991710000	-2.282212000
6	-2.151555000	3.068795000	0.998001000
1	-2.602358000	4.075450000	0.991673000
1	-2.626992000	2.485331000	1.797013000
1	-1.079408000	3.158923000	1.221216000

TS5

6	-3.826329000	-0.158378000	0.091103000
1	-4.915571000	-0.189052000	0.153037000
5	-0.647336000	-0.073159000	-0.090965000
5	-1.453428000	-0.755868000	1.338834000
5	-1.517929000	-1.613597000	-0.242095000
5	-1.625961000	-0.371315000	-1.542638000
5	-1.632552000	1.243351000	-0.757836000
5	-1.521519000	1.006090000	1.015098000
5	-2.977481000	0.136598000	1.542411000
5	-2.976179000	-1.475965000	0.768280000
5	-3.082596000	-1.240274000	-1.003582000
5	-3.151690000	0.517759000	-1.321860000
5	-3.085553000	1.369092000	0.252129000
17	1.140256000	-0.078023000	-0.222935000
17	-0.542752000	-1.399429000	2.727391000
17	-0.660835000	-3.153201000	-0.488220000
17	-0.876348000	-0.611615000	-3.139287000
17	-0.921251000	2.680551000	-1.556745000
17	-0.679593000	2.177114000	2.073313000
17	-3.825551000	0.388591000	3.073495000
17	-3.829834000	-2.820664000	1.534524000
17	-4.042666000	-2.352451000	-1.986208000
17	-4.172155000	1.150854000	-2.619921000
17	-4.034905000	2.840228000	0.504772000
8	4.284759000	-0.334784000	0.030179000
14	3.090884000	2.629299000	-0.441074000
14	4.761172000	-1.401026000	-1.194431000
6	5.792474000	-0.386087000	-2.390333000
1	6.681409000	0.028061000	-1.888369000
1	6.142762000	-1.017468000	-3.222279000
1	5.228456000	0.450351000	-2.830401000
6	5.831390000	-2.754323000	-0.460196000
1	6.205551000	-3.414452000	-1.259108000
1	6.705028000	-2.336633000	0.064019000
1	5.270655000	-3.383481000	0.247796000
6	3.258783000	-2.129937000	-2.028671000
1	2.638417000	-2.693218000	-1.314446000
1	2.600857000	-1.371729000	-2.479138000
1	3.572885000	-2.822882000	-2.825992000
6	4.893590000	2.789373000	-0.070601000
1	5.375692000	1.802988000	-0.127612000
1	5.324340000	3.427388000	-0.863190000
1	5.092709000	3.256756000	0.902666000
6	1.946768000	3.870854000	0.285481000
1	2.285518000	4.229352000	1.266603000
1	1.923597000	4.719509000	-0.423466000
1	0.919748000	3.480791000	0.360984000
6	2.680642000	1.963062000	-2.105380000
1	1.595872000	1.934705000	-2.275966000
1	3.152036000	2.661761000	-2.822846000
1	3.106218000	0.966025000	-2.269817000
14	4.043366000	-0.442897000	1.691581000
6	5.703771000	-0.386043000	2.558016000
1	6.295418000	0.483332000	2.230362000
1	5.570523000	-0.312931000	3.649478000
1	6.291934000	-1.294311000	2.355795000
6	3.094505000	-1.974200000	2.161289000
1	2.137158000	-2.044880000	1.625682000
1	3.684372000	-2.880738000	1.956261000

1	2.870128000	-1.961777000	3.239773000
6	3.097946000	1.143822000	2.111323000
1	2.286947000	0.989905000	2.841401000
1	3.737347000	1.966115000	2.460587000
1	2.501653000	1.453855000	1.217592000

TS6

6	-3.646109000	0.143028000	0.065725000
1	-4.733740000	0.222739000	0.107281000
5	-0.465671000	-0.090912000	-0.053390000
5	-1.263036000	1.501246000	-0.039738000
5	-1.277119000	0.459374000	1.424391000
5	-1.431190000	-1.248743000	0.887826000
5	-1.497358000	-1.263803000	-0.906408000
5	-1.391083000	0.436436000	-1.481914000
5	-2.820480000	1.305294000	-0.872057000
5	-2.750677000	1.319677000	0.914783000
5	-2.852022000	-0.369662000	1.486791000
5	-2.987540000	-1.427822000	0.050131000
5	-2.964229000	-0.393361000	-1.406943000
17	1.325376000	-0.251056000	-0.196928000
17	-0.385275000	3.051773000	-0.088739000
17	-0.389404000	0.933626000	2.903241000
17	-0.730888000	-2.618854000	1.797446000
17	-0.811452000	-2.608325000	-1.851750000
17	-0.596829000	0.846980000	-3.017896000
17	-3.690125000	2.582742000	-1.728369000
17	-3.547389000	2.608330000	1.824833000
17	-3.747750000	-0.756259000	2.961067000
17	-4.017914000	-2.862829000	0.107606000
17	-3.978708000	-0.802445000	-2.794513000
14	3.715166000	-0.873818000	1.621735000
8	4.014564000	-1.665411000	0.275695000
6	2.424832000	-1.360003000	2.814885000
1	1.744348000	-2.115382000	2.399120000
1	2.916211000	-1.732236000	3.729993000
1	1.812499000	-0.481096000	3.078962000
6	5.150188000	0.130083000	2.184121000
1	4.874390000	0.800287000	3.009403000
1	5.915278000	-0.581958000	2.541875000
1	5.585155000	0.714265000	1.361680000
14	3.665615000	-2.888249000	-0.914478000
6	3.213442000	-2.021958000	-2.495569000
1	3.932483000	-1.221903000	-2.729982000
1	3.239793000	-2.749685000	-3.322792000
1	2.203614000	-1.589835000	-2.454998000
6	2.325344000	-3.973229000	-0.219597000
1	2.112041000	-4.792388000	-0.925083000
1	2.628632000	-4.429663000	0.735843000
1	1.385301000	-3.421958000	-0.070891000
6	5.296152000	-3.787775000	-1.058408000
1	5.598034000	-4.232106000	-0.097350000
1	5.217101000	-4.603911000	-1.794221000
1	6.096016000	-3.109170000	-1.392536000
14	3.660349000	3.228357000	-0.465068000
6	2.893600000	2.440180000	-1.983616000
1	3.271173000	2.931365000	-2.894971000
1	3.137568000	1.368690000	-2.053006000
1	1.797008000	2.525362000	-1.976735000
6	3.006987000	2.440672000	1.128354000
1	3.077752000	1.338550000	1.048187000
1	3.573170000	2.769549000	2.013182000
1	1.939029000	2.651744000	1.283353000
6	5.530999000	2.960540000	-0.525470000
1	5.972882000	3.493020000	-1.382889000
1	6.030876000	3.326551000	0.385851000

1	5.777681000	1.892944000	-0.651641000
6	3.276046000	5.066221000	-0.402775000
1	2.187447000	5.228925000	-0.364005000
1	3.723332000	5.544921000	0.482573000
1	3.661823000	5.579852000	-1.297641000

E = sulfur

Naked

T-S-T

16	0.000000000	0.000000000	1.273612000
14	0.000000000	1.733089000	-0.022817000
14	0.000000000	-1.733089000	-0.022817000
6	1.728533000	2.068257000	-0.677001000
1	2.081134000	1.260941000	-1.335005000
1	1.744861000	3.009529000	-1.250817000
1	2.440535000	2.160829000	0.157252000
6	-1.213990000	1.552062000	-1.446551000
1	-2.225401000	1.333140000	-1.072116000
1	-1.255591000	2.494697000	-2.016675000
1	-0.924831000	0.752765000	-2.145240000
6	-0.536200000	3.142462000	1.093266000
1	0.146581000	3.245084000	1.950705000
1	-0.535600000	4.095194000	0.539193000
1	-1.550098000	2.969723000	1.484963000
6	-1.728533000	-2.068257000	-0.677001000
1	-2.081134000	-1.260941000	-1.335005000
1	-1.744861000	-3.009529000	-1.250817000
1	-2.440535000	-2.160829000	0.157252000
6	1.213990000	-1.552062000	-1.446551000
1	0.924831000	-0.752765000	-2.145240000
1	2.225401000	-1.333140000	-1.072116000
1	1.255591000	-2.494697000	-2.016675000
6	0.536200000	-3.142462000	1.093266000
1	-0.146581000	-3.245084000	1.950705000
1	0.535600000	-4.095194000	0.539193000
1	1.550098000	-2.969723000	1.484963000

T3S(+)

16	-0.001063000	0.000368000	-0.742099000
14	0.706647000	2.018315000	0.005444000
14	1.396870000	-1.619653000	0.004360000
14	-2.102850000	-0.398394000	0.004477000
6	2.094544000	2.454241000	-1.153558000
1	1.753389000	2.443766000	-2.199515000
1	2.431369000	3.478030000	-0.919488000
1	2.962460000	1.788150000	-1.060300000
6	1.219359000	1.854931000	1.789462000
1	1.523284000	2.851519000	2.151131000
1	0.393798000	1.507067000	2.426835000
1	2.077795000	1.182084000	1.924648000
6	-0.749666000	3.155397000	-0.230291000
1	-1.597710000	2.927734000	0.430158000
1	-0.410862000	4.176997000	0.011157000
1	-1.096733000	3.161785000	-1.274031000
6	3.108599000	-0.924160000	-0.233299000
1	3.825215000	-1.727186000	0.008763000
1	3.285840000	-0.628349000	-1.277837000
1	3.335324000	-0.074374000	0.425281000
6	1.079174000	-3.038942000	-1.154784000
1	0.068403000	-3.457015000	-1.059760000
1	1.239077000	-2.737346000	-2.200744000
1	1.797599000	-3.842654000	-0.921737000
6	1.004057000	-1.987524000	1.788566000
1	-0.006726000	-2.396338000	1.927534000
1	1.716483000	-2.750965000	2.143637000
1	1.118683000	-1.101870000	2.429376000
6	-2.357668000	-2.228574000	-0.232245000
1	-3.412102000	-2.447183000	0.006962000
1	-2.187115000	-2.531840000	-1.275883000
1	-1.737435000	-2.848918000	0.429721000
6	-2.224944000	0.127404000	1.787965000
1	-2.075487000	1.207941000	1.923545000
1	-3.241414000	-0.109676000	2.144249000
1	-1.513899000	-0.411788000	2.429644000
6	-3.172329000	0.586540000	-1.155823000
1	-3.030612000	1.671191000	-1.059118000
1	-2.990045000	0.298744000	-2.201998000
1	-4.227657000	0.364989000	-0.924555000

Monocat_SiM4

16	1.676996000	0.659682000	-1.195748000
14	-3.054488000	-0.690344000	-0.008399000
14	2.600529000	-0.925102000	0.043027000
14	0.320350000	1.696587000	0.019892000
6	-2.571094000	-2.087778000	-1.139878000
1	-1.712524000	-2.656247000	-0.751293000
1	-3.416639000	-2.789328000	-1.228218000
1	-2.327792000	-1.734560000	-2.153630000
6	-4.440501000	0.360219000	-0.672136000
1	-5.358573000	-0.247493000	-0.727673000
1	-4.655603000	1.221519000	-0.021269000
1	-4.229559000	0.730088000	-1.687242000
6	-3.292259000	-1.216737000	1.762152000
1	-3.483331000	-0.361381000	2.428289000
1	-4.166579000	-1.884966000	1.826567000
1	-2.423817000	-1.774753000	2.145181000
6	1.248536000	-1.894354000	0.918245000
1	1.701499000	-2.772788000	1.406544000
1	0.744994000	-1.320501000	1.711871000
1	0.500161000	-2.269120000	0.202943000
6	3.796307000	-0.151187000	1.247727000
1	4.532144000	0.470168000	0.715516000
1	3.298755000	0.473142000	2.003214000
1	4.345445000	-0.946431000	1.778129000
6	3.459742000	-1.969035000	-1.238045000
1	4.208988000	-1.379745000	-1.788137000
1	3.981902000	-2.805979000	-0.746705000
1	2.747896000	-2.387106000	-1.965345000
6	0.701233000	1.891350000	1.829045000
1	-0.107812000	2.438726000	2.337860000
1	0.860210000	0.935894000	2.348222000
1	1.622666000	2.487554000	1.926598000
6	-0.216751000	3.259949000	-0.820436000
1	-0.492438000	3.084338000	-1.870894000
1	-1.061556000	3.735962000	-0.299383000
1	0.628913000	3.966545000	-0.813943000
6	-1.444570000	0.534132000	0.030157000
1	-1.905305000	1.298185000	0.667255000
1	-0.806299000	-0.236156000	0.480644000
1	-1.431024000	0.674192000	-1.057401000

Monocat

16	0.243119000	-1.200874000	0.002572000
14	1.879323000	-0.005265000	-0.007875000
14	-1.648392000	0.078750000	0.000400000
6	3.513179000	-0.842647000	0.004265000
1	3.413785000	-1.933039000	0.094005000
1	4.052430000	-0.604339000	-0.929014000
1	4.123768000	-0.457505000	0.838445000
6	1.848072000	1.835667000	-0.004893000
1	0.879670000	2.257871000	-0.301537000
1	2.087601000	2.187173000	1.014696000
1	2.637769000	2.217496000	-0.672364000
6	-1.565535000	1.169761000	1.507184000
1	-0.767113000	1.924275000	1.459487000
1	-2.522351000	1.711635000	1.595217000
1	-1.437128000	0.574053000	2.423336000
6	-1.673274000	0.987711000	-1.624365000
1	-0.863394000	1.723895000	-1.729671000
1	-1.622698000	0.286971000	-2.471156000
1	-2.627053000	1.536324000	-1.701966000
6	-2.929303000	-1.259663000	0.119772000
1	-3.928730000	-0.793678000	0.128606000
1	-2.885107000	-1.944127000	-0.740597000
1	-2.823211000	-1.846783000	1.044235000

Dicat

14	1.163917000	-0.647661000	0.809770000
14	0.315393000	3.006401000	1.967037000
16	0.636860000	1.441780000	0.235885000
6	0.992134000	-0.991322000	2.611084000
1	1.726109000	-0.407028000	3.188929000
1	1.208206000	-2.058930000	2.786019000
1	-0.019734000	-0.780275000	2.983095000
6	2.752596000	-1.086386000	-0.012838000
1	2.780700000	-0.759834000	-1.061276000
1	2.931572000	-2.172435000	0.039347000
1	3.576353000	-0.588160000	0.525859000
6	-0.923462000	2.337428000	3.171584000
1	-1.923951000	2.201889000	2.738057000
1	-1.021419000	3.095685000	3.969377000
1	-0.599938000	1.406306000	3.656977000
6	-0.259528000	4.449027000	0.958268000
1	0.461608000	4.723155000	0.174118000
1	-0.352486000	5.314805000	1.637602000
1	-1.249442000	4.289307000	0.505384000
6	2.054541000	3.106669000	2.596743000
1	2.389938000	2.179524000	3.085041000
1	2.085227000	3.901926000	3.362504000
1	2.769109000	3.384036000	1.807713000
14	-1.163917000	0.647661000	-0.809770000
14	-0.315393000	-3.006401000	-1.967037000
16	-0.636860000	-1.441780000	-0.235885000
6	-0.992134000	0.991322000	-2.611084000
1	-1.726109000	0.407028000	-3.188929000
1	-1.208206000	2.058930000	-2.786019000
1	0.019734000	0.780275000	-2.983095000
6	-2.752596000	1.086386000	0.012838000
1	-2.780700000	0.759834000	1.061276000
1	-2.931572000	2.172435000	-0.039347000
1	-3.576353000	0.588160000	-0.525859000
6	0.923462000	-2.337428000	-3.171584000
1	1.923951000	-2.201889000	-2.738057000
1	1.021419000	-3.095685000	-3.969377000
1	0.599938000	-1.406306000	-3.656977000
6	0.259528000	-4.449027000	-0.958268000
1	-0.461608000	-4.723155000	-0.174118000
1	0.352486000	-5.314805000	-1.637602000
1	1.249442000	-4.289307000	-0.505384000
6	-2.054541000	-3.106669000	-2.596743000
1	-2.389938000	-2.179524000	-3.085041000
1	-2.085227000	-3.901926000	-3.362504000
1	-2.769109000	-3.384036000	-1.807713000

Toluene

T3S(+)_toluene

16	2.125192000	0.336161000	-0.004503000
14	0.870697000	2.132119000	-0.581510000
14	1.527854000	-0.360642000	2.066417000
14	1.771057000	-1.336354000	-1.490273000
6	1.713462000	3.543632000	0.290738000
1	2.772536000	3.612258000	0.000515000
1	1.219365000	4.482358000	-0.010631000
1	1.652743000	3.470768000	1.384705000
6	-0.888177000	1.841966000	-0.050527000
1	-1.484725000	2.719567000	-0.350540000
1	-1.338702000	0.959564000	-0.526867000
1	-0.993200000	1.734842000	1.038056000
6	1.059564000	2.279505000	-2.429657000
1	0.594351000	1.453718000	-2.985513000
1	0.552832000	3.209498000	-2.737829000
1	2.115251000	2.365603000	-2.726522000
6	1.361933000	1.201382000	3.069546000
1	1.186881000	0.903034000	4.116860000
1	2.285083000	1.799081000	3.046871000
1	0.516239000	1.832081000	2.762902000
6	2.995574000	-1.358475000	2.626392000
1	3.144282000	-2.273932000	2.038551000
1	3.918409000	-0.760726000	2.586874000
1	2.831452000	-1.657036000	3.675242000
6	-0.058292000	-1.326809000	1.953752000
1	0.042730000	-2.240583000	1.351604000
1	-0.340003000	-1.636701000	2.974136000
1	-0.890826000	-0.736349000	1.545710000
6	2.401838000	-2.874637000	-0.647929000
1	2.356797000	-3.693199000	-1.385959000
1	3.451908000	-2.768520000	-0.337995000
1	1.799355000	-3.182119000	0.218088000
6	-0.044374000	-1.417590000	-1.889968000
1	-0.410560000	-0.507896000	-2.385937000
1	-0.204077000	-2.257296000	-2.587014000
1	-0.668513000	-1.599228000	-1.003544000
6	2.854836000	-0.872638000	-2.930166000
1	2.534746000	0.050869000	-3.430741000
1	3.902770000	-0.757560000	-2.615298000
1	2.813051000	-1.687509000	-3.672080000
6	-3.554316000	-1.567081000	-1.194176000
6	-3.367863000	-2.239978000	0.012845000
6	-3.552241000	-1.553831000	1.213343000
6	-3.917622000	-0.208696000	1.204351000
6	-4.114466000	0.478614000	-0.000813000
6	-3.920623000	-0.221654000	-1.198476000
1	-3.426371000	-2.096330000	-2.141813000
1	-3.097905000	-3.298779000	0.018878000
1	-3.423260000	-2.074222000	2.165742000
1	-4.072252000	0.316162000	2.151556000
1	-4.077548000	0.292705000	-2.150987000
6	-4.562302000	1.910753000	-0.006089000
1	-4.268660000	2.425745000	-0.932201000
1	-5.660680000	1.974421000	0.064552000
1	-4.152656000	2.468624000	0.848796000

Monocat_toluene

6	-1.983385000	-1.043083000	-1.131450000
6	-3.110921000	-1.399733000	-0.416538000
6	-3.980992000	-0.418718000	0.096711000
6	-3.698938000	0.941825000	-0.148111000
6	-2.579636000	1.314156000	-0.865067000
6	-1.653111000	0.330727000	-1.315673000
1	-1.317235000	-1.809646000	-1.535148000
1	-3.342387000	-2.455859000	-0.260791000
1	-4.392517000	1.704698000	0.213004000
1	-2.392139000	2.368695000	-1.080707000
1	-0.948990000	0.592904000	-2.116588000
14	-0.110091000	0.505019000	0.173328000
6	-0.838247000	0.022603000	1.809989000
1	-1.084496000	-1.046891000	1.849400000
1	-0.063601000	0.215453000	2.570887000
1	-1.722338000	0.621661000	2.072785000
6	0.355813000	2.304449000	0.059329000
1	1.267235000	2.475186000	0.653518000
1	0.570184000	2.609976000	-0.975228000
1	-0.434182000	2.947661000	0.475081000
6	-5.175079000	-0.810236000	0.896926000
1	-5.558675000	-1.795476000	0.598938000
1	-4.896978000	-0.878551000	1.963333000
1	-5.980425000	-0.067553000	0.817837000
16	1.258424000	-0.873021000	-0.641242000
14	3.252764000	-0.225481000	0.094073000
6	3.829919000	1.223018000	-0.932571000
1	3.213298000	2.121581000	-0.788351000
1	4.864828000	1.479324000	-0.651675000
1	3.823881000	0.967448000	-2.002793000
6	3.122345000	0.185642000	1.916236000
1	2.697225000	-0.657061000	2.482094000
1	4.133528000	0.377523000	2.311641000
1	2.523718000	1.086432000	2.120800000
6	4.278611000	-1.751890000	-0.204481000
1	4.286149000	-2.026849000	-1.270007000
1	5.319541000	-1.559042000	0.102564000
1	3.903895000	-2.609368000	0.373851000

Toluene_dicat_toluene

14	0.011701000	-0.106780000	1.561810000
14	-2.604941000	-2.596013000	-0.164829000
16	-0.514241000	-1.495111000	-0.101345000
6	1.443900000	-0.841296000	2.449068000
1	1.086327000	-1.744448000	2.972667000
1	1.830499000	-0.146573000	3.210202000
1	2.258571000	-1.136193000	1.771019000
6	-1.410575000	0.495292000	2.561291000
1	-2.231684000	0.871948000	1.933943000
1	-1.048902000	1.321747000	3.196616000
1	-1.785136000	-0.298231000	3.225774000
6	-1.990842000	-4.335306000	-0.324532000
1	-1.419225000	-4.492124000	-1.251255000
1	-2.867849000	-5.005164000	-0.357147000
1	-1.372185000	-4.639885000	0.532505000
6	-3.465597000	-1.924857000	-1.657978000
1	-3.763043000	-0.872755000	-1.537950000
1	-4.391890000	-2.515981000	-1.771967000
1	-2.887339000	-2.060949000	-2.583337000
6	-3.395948000	-2.183400000	1.456424000
1	-2.784327000	-2.484095000	2.319488000
1	-4.333489000	-2.764566000	1.507063000
1	-3.665476000	-1.119977000	1.525512000
14	-0.012061000	0.112739000	-1.560514000
14	2.611405000	2.595975000	0.165717000
16	0.518092000	1.499681000	0.102584000
6	-1.446893000	0.848224000	-2.442761000
1	-1.090835000	1.752117000	-2.966124000
1	-1.835554000	0.154500000	-3.203761000
1	-2.259918000	1.142164000	-1.762314000
6	1.407319000	-0.487994000	-2.564886000
1	2.229513000	-0.866645000	-1.940193000
1	1.043419000	-1.312775000	-3.201120000
1	1.780825000	0.306668000	-3.228597000
6	2.000820000	4.336503000	0.325456000
1	1.429945000	4.494585000	1.252421000
1	2.879172000	5.004623000	0.357546000
1	1.382353000	4.642180000	-0.531326000
6	3.471172000	1.923378000	1.658702000
1	3.766476000	0.870638000	1.539049000
1	4.398740000	2.512660000	1.771966000
1	2.893636000	2.061112000	2.584261000
6	3.401106000	2.181739000	-1.455741000
1	2.790079000	2.484225000	-2.318585000
1	4.340078000	2.760600000	-1.506349000
1	3.668015000	1.117683000	-1.525200000
6	5.658037000	-0.670608000	-0.281281000
6	5.504071000	-1.071253000	1.054331000
6	4.658496000	-2.125107000	1.397691000
6	3.939368000	-2.800701000	0.409496000
6	4.082553000	-2.414697000	-0.922594000
6	4.930720000	-1.358383000	-1.262090000
1	6.084631000	-0.570068000	1.834318000
1	4.585054000	-2.440255000	2.441842000
1	3.302959000	-3.649081000	0.672628000
1	3.563007000	-2.967703000	-1.709822000
1	5.064765000	-1.088647000	-2.314139000
6	-3.950352000	2.803128000	-0.401350000
6	-4.096705000	2.411233000	0.928681000

6	-4.940730000	1.349250000	1.260797000
6	-5.660793000	0.661647000	0.274554000
6	-5.503719000	1.068237000	-1.058910000
6	-4.662197000	2.127688000	-1.394906000
1	-3.317257000	3.655727000	-0.658770000
1	-3.583125000	2.963853000	1.720080000
1	-5.077400000	1.074781000	2.311278000
1	-6.078759000	0.567212000	-1.843083000
1	-4.586326000	2.447270000	-2.437534000
6	6.624070000	0.417249000	-0.647599000
1	6.582091000	1.258493000	0.061165000
1	7.658322000	0.037388000	-0.622997000
1	6.446524000	0.801446000	-1.661860000
6	-6.622726000	-0.432499000	0.632902000
1	-6.572935000	-1.271274000	-0.078293000
1	-6.447896000	-0.818941000	1.646783000
1	-7.658873000	-0.058111000	0.604605000

System T-S-T/T+

6	1.100160000	2.809991000	0.379631000
1	1.607054000	3.752275000	0.595469000
5	-0.371573000	0.080090000	-0.236791000
5	1.196508000	0.432329000	-0.985420000
5	1.073032000	0.092453000	0.782232000
5	-0.481386000	0.790809000	1.378875000
5	-1.317676000	1.562994000	-0.020565000
5	-0.285847000	1.339615000	-1.482182000
5	1.253099000	2.193790000	-1.206945000
5	2.082759000	1.432220000	0.184279000
5	1.059559000	1.650515000	1.632535000
5	-0.404464000	2.553922000	1.139576000
5	-0.284660000	2.889333000	-0.612456000
17	-1.090161000	-1.526567000	-0.826915000
17	1.993295000	-0.704000000	-2.092018000
17	1.759432000	-1.382669000	1.500210000
17	-1.379416000	0.101006000	2.750723000
17	-3.090275000	1.657061000	-0.133496000
17	-1.012360000	1.125571000	-3.088528000
17	2.191087000	3.037831000	-2.439515000
17	3.840708000	1.521103000	0.324901000
17	1.800466000	1.955427000	3.204336000
17	-1.109304000	3.751136000	2.227364000
17	-0.873249000	4.422269000	-1.258328000
14	-2.336133000	-2.871374000	0.575518000
16	-3.992663000	-1.780739000	1.190292000
6	-1.192449000	-3.353729000	1.940132000
1	-0.974480000	-2.510155000	2.607507000
1	-1.683383000	-4.154588000	2.517342000
1	-0.246813000	-3.735110000	1.528172000
6	-2.566396000	-4.202907000	-0.702451000
1	-1.590046000	-4.607158000	-1.010737000
1	-3.144045000	-5.016214000	-0.233764000
1	-3.109912000	-3.864889000	-1.594611000
14	-5.330486000	-1.615729000	-0.567317000
6	-4.403190000	-1.155888000	-2.123228000
1	-3.735833000	-1.947837000	-2.491404000
1	-5.149445000	-0.963721000	-2.912308000
1	-3.810167000	-0.240620000	-1.985311000
6	-6.480321000	-0.251936000	-0.017907000
1	-7.233368000	-0.058924000	-0.798898000
1	-7.008474000	-0.524753000	0.908265000
1	-5.922388000	0.678736000	0.162759000
6	-6.241223000	-3.242049000	-0.756277000
1	-6.741935000	-3.521102000	0.182988000
1	-7.011954000	-3.143441000	-1.538529000
1	-5.572988000	-4.065782000	-1.047635000
14	5.636147000	-2.607922000	-0.240975000
6	7.139321000	-3.746328000	-0.329637000
1	7.087562000	-4.538175000	0.434992000
1	7.211651000	-4.234529000	-1.314878000
1	8.072199000	-3.183187000	-0.165784000
6	5.782423000	-1.287369000	-1.573713000
1	5.837312000	-1.740919000	-2.576426000
1	4.915816000	-0.609538000	-1.555808000
1	6.690296000	-0.679941000	-1.429026000
6	4.078326000	-3.632652000	-0.526491000

1	3.960771000	-4.397992000	0.257815000
1	3.183209000	-2.992521000	-0.519334000
1	4.121268000	-4.148718000	-1.499275000
6	5.575150000	-1.804132000	1.460586000
1	6.489980000	-1.219990000	1.650579000
1	4.715503000	-1.123100000	1.548969000
1	5.490486000	-2.563115000	2.255000000

Iso1

6	0.696025000	-0.090802000	0.511699000
1	-0.340689000	-0.062774000	0.841891000
5	3.779761000	-0.217424000	-0.465539000
5	3.042453000	1.314851000	0.098647000
5	3.351847000	-0.009568000	1.272883000
5	3.019622000	-1.570614000	0.436691000
5	2.502375000	-1.212211000	-1.254498000
5	2.515506000	0.570370000	-1.465418000
5	1.338851000	1.203143000	-0.322312000
5	1.826923000	0.899864000	1.340170000
5	1.808880000	-0.867488000	1.545482000
5	1.294833000	-1.603906000	-0.003360000
5	0.980974000	-0.294094000	-1.167270000
17	5.464868000	-0.279474000	-1.003223000
17	3.867260000	2.885221000	0.139789000
17	4.560635000	0.144598000	2.557711000
17	3.882033000	-3.059420000	0.852524000
17	2.822919000	-2.316312000	-2.601492000
17	2.805224000	1.380464000	-3.016996000
17	0.451679000	2.771187000	-0.729506000
17	1.271701000	1.994794000	2.626195000
17	1.243256000	-1.559712000	3.070718000
17	0.258532000	-3.037296000	0.006533000
17	-0.404361000	-0.365879000	-2.277498000
14	-1.769280000	2.952369000	-0.176661000
6	-2.127317000	1.731793000	1.163054000
1	-3.044542000	2.089997000	1.661141000
1	-2.357277000	0.735799000	0.757443000
1	-1.330455000	1.702450000	1.920852000
6	-2.555366000	2.703196000	-1.827657000
1	-2.383383000	1.680293000	-2.190215000
1	-3.643002000	2.842830000	-1.723948000
1	-2.172781000	3.427129000	-2.561982000
6	-1.694228000	4.708121000	0.417930000
1	-1.321627000	5.393361000	-0.357734000
1	-2.713587000	5.029421000	0.690809000
1	-1.055101000	4.795084000	1.309314000
16	-4.381140000	-0.308093000	-0.837076000
14	-4.038002000	-2.348076000	-0.136421000
14	-6.043206000	0.471371000	0.307432000
6	-5.986325000	2.331473000	0.019793000
6	-7.670815000	-0.188252000	-0.348267000
1	-7.764775000	-1.272203000	-0.192070000
1	-8.514877000	0.304978000	0.161097000
1	-7.756421000	0.007856000	-1.427819000
6	-5.873322000	0.117304000	2.144989000
1	-4.912163000	0.481004000	2.538562000
1	-6.683197000	0.623704000	2.695121000
1	-5.944305000	-0.958174000	2.364802000
6	-3.073475000	-3.154199000	-1.518322000
1	-2.128031000	-2.627155000	-1.711180000
1	-2.828146000	-4.193938000	-1.247660000
1	-3.664195000	-3.167604000	-2.446817000
6	-5.665569000	-3.249068000	0.121674000
1	-6.259258000	-2.833171000	0.949025000
1	-6.276746000	-3.225764000	-0.793039000
1	-5.454037000	-4.303759000	0.362945000
6	-3.059906000	-2.305705000	1.463983000
1	-2.070042000	-1.850662000	1.316299000

1	-3.589406000	-1.753911000	2.254022000
1	-2.885566000	-3.333049000	1.823456000
1	-6.845398000	2.816348000	0.510885000
1	-5.069658000	2.781296000	0.430810000
1	-6.038281000	2.564105000	-1.055148000

Iso2

6	-0.762141000	0.083137000	0.131169000
1	-1.863116000	0.012973000	0.095174000
5	2.385687000	0.273958000	0.301650000
5	1.417890000	0.356376000	1.784321000
5	1.568586000	-1.188268000	0.864290000
5	1.633326000	-0.782767000	-0.894527000
5	1.538841000	1.009421000	-1.064598000
5	1.400160000	1.716446000	0.591144000
5	-0.117057000	1.128206000	1.313992000
5	-0.016973000	-0.649826000	1.480404000
5	0.120099000	-1.343701000	-0.154944000
5	0.097178000	-0.001104000	-1.333135000
5	-0.042375000	1.524691000	-0.426041000
17	4.207141000	0.548486000	0.579312000
17	2.153711000	0.547093000	3.387308000
17	2.438498000	-2.591512000	1.525017000
17	2.493568000	-1.808188000	-2.076650000
17	2.378705000	1.864995000	-2.378613000
17	2.115452000	3.293180000	0.979072000
17	-1.157610000	2.062238000	2.389486000
17	-0.956121000	-1.483978000	2.719182000
17	-0.660273000	-2.878958000	-0.556210000
17	-0.731096000	-0.201772000	-2.882811000
17	-0.988507000	2.864406000	-1.085146000
14	5.751549000	-0.530504000	-0.687993000
6	5.545940000	-2.327705000	-0.311485000
1	6.361310000	-2.875302000	-0.814216000
1	5.618395000	-2.508353000	0.770557000
1	4.584042000	-2.716088000	-0.670991000
6	7.220728000	0.240229000	0.152497000
1	7.262556000	-0.013039000	1.222157000
1	8.134402000	-0.153905000	-0.323750000
1	7.229513000	1.334770000	0.042455000
6	5.500411000	0.062602000	-2.419271000
1	5.548934000	1.159866000	-2.465947000
1	6.314106000	-0.351599000	-3.038836000
1	4.538027000	-0.266801000	-2.832087000
16	-4.184261000	-0.092596000	0.728924000
14	-4.823393000	-1.999960000	-0.094943000
14	-5.009920000	1.536202000	-0.451509000
6	-4.434147000	3.059124000	0.474022000
6	-4.363835000	1.515671000	-2.212662000
1	-4.768326000	0.666214000	-2.782304000
1	-4.670700000	2.442091000	-2.725917000
1	-3.266241000	1.458672000	-2.243074000
6	-6.887841000	1.477319000	-0.464357000
1	-7.287189000	1.351255000	0.553167000
1	-7.278422000	2.423848000	-0.873282000
1	-7.272505000	0.658885000	-1.089741000
6	-4.202506000	-2.242681000	-1.849277000
1	-3.122707000	-2.050116000	-1.928387000
1	-4.387829000	-3.283262000	-2.163377000
1	-4.723050000	-1.581219000	-2.557390000
6	-6.695449000	-2.155442000	-0.056863000
1	-7.093253000	-1.900863000	0.936978000
1	-7.179907000	-1.503468000	-0.797826000
1	-6.980741000	-3.195419000	-0.286587000
6	-4.061062000	-3.246422000	1.077036000
1	-2.966329000	-3.147586000	1.109126000

1	-4.447676000	-3.104639000	2.097727000
1	-4.307615000	-4.270355000	0.751931000
1	-4.777382000	3.969492000	-0.043887000
1	-4.844229000	3.066440000	1.495452000
1	-3.337529000	3.094778000	0.545581000

Iso3

6	-3.705496000	0.585972000	1.076759000
1	-4.579534000	0.842379000	1.678232000
5	-1.168684000	-0.151740000	-0.659930000
5	-2.760696000	-0.003725000	-1.430256000
5	-1.898007000	1.453301000	-0.804418000
5	-1.007665000	0.976091000	0.694355000
5	-1.317946000	-0.778001000	0.991226000
5	-2.397508000	-1.384006000	-0.319922000
5	-3.946230000	-0.512136000	-0.208251000
5	-3.640110000	1.228206000	-0.502267000
5	-2.570330000	1.827587000	0.797491000
5	-2.215136000	0.461001000	1.896614000
5	-3.065787000	-0.981929000	1.275854000
17	0.138701000	-0.694904000	-1.864571000
17	-2.998784000	-0.257628000	-3.170390000
17	-1.267031000	2.702999000	-1.901252000
17	0.490350000	1.784806000	1.216575000
17	-0.138949000	-1.843064000	1.791825000
17	-2.251030000	-3.048966000	-0.922368000
17	-5.534490000	-1.202074000	-0.548074000
17	-4.924447000	2.260762000	-1.133982000
17	-2.791566000	3.450160000	1.456255000
17	-2.085271000	0.737340000	3.635055000
17	-3.779729000	-2.137053000	2.403428000
14	2.307736000	0.043990000	-1.616058000
16	2.913947000	-0.561049000	0.277129000
6	2.232804000	1.870215000	-1.861982000
1	1.712527000	2.376656000	-1.039250000
1	3.270847000	2.236863000	-1.913647000
1	1.718224000	2.100681000	-2.806072000
6	2.947489000	-0.868946000	-3.103909000
1	2.398318000	-0.555184000	-4.004818000
1	4.005472000	-0.587519000	-3.231284000
1	2.887409000	-1.960842000	-3.010572000
14	3.343869000	-2.726710000	0.153977000
6	1.952796000	-3.651729000	-0.684222000
1	1.872626000	-3.447175000	-1.761416000
1	2.139632000	-4.732279000	-0.568070000
1	0.984505000	-3.420008000	-0.217314000
6	3.482165000	-3.167251000	1.963000000
1	3.696555000	-4.242677000	2.071314000
1	4.295352000	-2.605892000	2.448126000
1	2.543655000	-2.945988000	2.492200000
6	4.984345000	-2.946852000	-0.722809000
1	5.770920000	-2.364410000	-0.219761000
1	5.276324000	-4.009678000	-0.699869000
1	4.944928000	-2.631226000	-1.775293000
14	6.343914000	1.624778000	0.475313000
6	7.920117000	2.649741000	0.572327000
1	8.809258000	2.037453000	0.352686000
1	7.898613000	3.482478000	-0.148706000
1	8.049430000	3.080948000	1.577832000
6	4.859183000	2.716825000	0.860067000
1	3.922817000	2.137913000	0.854511000
1	4.959666000	3.172228000	1.858221000
1	4.763341000	3.537345000	0.130767000
6	6.428447000	0.205436000	1.711081000
1	5.492963000	-0.374579000	1.688799000
1	7.265114000	-0.475566000	1.486538000
1	6.566383000	0.580290000	2.737571000

6	6.180629000	0.910800000	-1.266897000
1	7.081208000	0.343589000	-1.551222000
1	5.324842000	0.219930000	-1.306196000
1	6.032021000	1.703304000	-2.018127000

Iso4

6	1.235698000	2.526516000	0.552463000
1	1.840432000	3.373366000	0.882009000
5	-0.514285000	0.071251000	-0.395850000
5	0.933061000	0.534190000	-1.310819000
5	1.074143000	-0.208868000	0.328191000
5	-0.304356000	0.384649000	1.332884000
5	-1.300424000	1.491524000	0.314781000
5	-0.540817000	1.583817000	-1.319292000
5	1.079020000	2.297535000	-1.133793000
5	2.067259000	1.201235000	-0.121684000
5	1.316956000	1.107073000	1.496125000
5	-0.137445000	2.150137000	1.488579000
5	-0.283823000	2.883918000	-0.135158000
17	-1.436729000	-1.326790000	-1.198783000
17	1.464304000	-0.337261000	-2.762316000
17	1.747045000	-1.837730000	0.561641000
17	-1.014132000	-0.574398000	2.652063000
17	-3.054917000	1.671254000	0.544075000
17	-1.535389000	1.779040000	-2.777912000
17	1.860007000	3.378666000	-2.288455000
17	3.826393000	1.207783000	-0.282775000
17	2.330518000	1.006301000	2.937279000
17	-0.563609000	3.079601000	2.926652000
17	-0.855863000	4.544784000	-0.303603000
14	-2.558972000	-2.896994000	0.063600000
16	-4.003222000	-1.904227000	1.177412000
6	-1.256882000	-3.737813000	1.064087000
1	-0.867006000	-3.091155000	1.860345000
1	-1.710138000	-4.636124000	1.514584000
1	-0.422800000	-4.042187000	0.415228000
6	-3.097662000	-3.887072000	-1.416120000
1	-2.218707000	-4.257485000	-1.965572000
1	-3.664387000	-4.756730000	-1.045582000
1	-3.740152000	-3.327743000	-2.108523000
14	-5.582081000	-1.286438000	-0.247593000
6	-4.875675000	-0.521101000	-1.799559000
1	-4.344072000	-1.234340000	-2.445339000
1	-5.718515000	-0.115956000	-2.384396000
1	-4.193978000	0.309349000	-1.566834000
6	-6.522517000	-0.036135000	0.771043000
1	-7.373728000	0.354579000	0.190424000
1	-6.915714000	-0.488141000	1.694228000
1	-5.875059000	0.809115000	1.047366000
6	-6.637202000	-2.786567000	-0.629211000
1	-7.014308000	-3.244793000	0.297385000
1	-7.504434000	-2.481246000	-1.237722000
1	-6.086138000	-3.553487000	-1.193049000
14	6.123279000	-2.221650000	-0.084793000
6	7.149740000	-0.747197000	-0.655181000
1	7.927992000	-0.493299000	0.082193000
1	7.650120000	-0.962359000	-1.612946000
1	6.517937000	0.142715000	-0.799903000
6	5.347218000	-1.854283000	1.590758000
1	4.704421000	-0.962228000	1.553762000
1	4.726037000	-2.697291000	1.932972000
1	6.127547000	-1.681660000	2.349579000
6	7.255950000	-3.722577000	0.087963000
1	7.740975000	-3.969629000	-0.870113000
1	8.050240000	-3.537294000	0.828907000

1	6.691602000	-4.610056000	0.417190000
6	4.801536000	-2.599619000	-1.372085000
1	4.181708000	-3.456912000	-1.064670000
1	4.129732000	-1.741431000	-1.523993000
1	5.263417000	-2.847272000	-2.341570000

Iso5

6	-0.542607000	-0.042443000	-1.661880000
1	-0.600661000	-0.338222000	-2.711023000
5	-0.386717000	0.843174000	1.434422000
5	-1.601551000	1.549302000	0.330511000
5	-1.720689000	-0.180823000	0.831436000
5	-0.056035000	-0.854430000	0.918510000
5	1.045612000	0.462189000	0.460352000
5	0.141212000	1.947984000	0.114336000
5	-0.875912000	1.598889000	-1.300555000
5	-2.014676000	0.294332000	-0.863133000
5	-1.075336000	-1.179669000	-0.500769000
5	0.651916000	-0.791198000	-0.717429000
5	0.776220000	0.931672000	-1.214392000
17	-0.225153000	1.319919000	3.136056000
17	-2.781505000	2.761400000	0.852222000
17	-3.008776000	-0.771939000	1.891419000
17	0.447834000	-2.118526000	2.060970000
17	2.625485000	0.522756000	1.425225000
17	0.823660000	3.558745000	0.435613000
17	-1.278392000	2.748212000	-2.577919000
17	-3.547646000	0.161539000	-1.729830000
17	-1.683156000	-2.764289000	-0.985425000
17	1.750315000	-1.997790000	-1.410751000
17	1.983460000	1.413317000	-2.421505000
14	4.593380000	1.154998000	0.409098000
16	4.953855000	-0.175680000	-1.144629000
6	5.596002000	1.012510000	1.968326000
1	5.620825000	-0.004637000	2.381485000
1	6.629055000	1.314857000	1.731458000
1	5.204070000	1.698909000	2.734550000
6	4.319982000	2.894977000	-0.138992000
1	3.936703000	3.501040000	0.694529000
1	5.292789000	3.300112000	-0.462803000
1	3.611335000	2.955568000	-0.974721000
14	5.545362000	-2.094343000	-0.207381000
6	5.416334000	-3.269058000	-1.652358000
1	6.070110000	-2.958430000	-2.481296000
1	5.720402000	-4.279867000	-1.335852000
1	4.383005000	-3.321754000	-2.025421000
6	4.393107000	-2.578616000	1.181768000
1	4.680929000	-3.587984000	1.520544000
1	4.448308000	-1.913506000	2.055154000
1	3.346193000	-2.618567000	0.848415000
6	7.316932000	-1.929685000	0.378237000
1	7.419818000	-1.213577000	1.206534000
1	7.679960000	-2.908100000	0.733845000
1	7.970696000	-1.601161000	-0.443636000
14	-7.042214000	-1.074340000	0.054833000
6	-7.373081000	-0.638494000	-1.750813000
1	-7.754871000	-1.509485000	-2.307346000
1	-8.118954000	0.168209000	-1.834294000
1	-6.452708000	-0.297077000	-2.249741000
6	-5.794744000	-2.481588000	0.142126000
1	-4.843962000	-2.207731000	-0.338962000
1	-5.570229000	-2.750233000	1.186394000
1	-6.187466000	-3.380077000	-0.361106000
6	-8.659037000	-1.642143000	0.847818000
1	-9.424411000	-0.850635000	0.803562000
1	-9.063237000	-2.530435000	0.336203000

1	-8.509061000	-1.904586000	1.907505000
6	-6.416866000	0.449811000	0.964687000
1	-6.196583000	0.222465000	2.019635000
1	-5.492872000	0.840291000	0.512910000
1	-7.172749000	1.251550000	0.942616000

Iso6

6	0.695840000	-0.090394000	0.511382000
1	-0.340957000	-0.061981000	0.841284000
5	3.779746000	-0.218197000	-0.465014000
5	3.042812000	1.314433000	0.098716000
5	3.351467000	-0.009906000	1.273228000
5	3.018928000	-1.570988000	0.437284000
5	2.502273000	-1.212709000	-1.254144000
5	2.516044000	0.569846000	-1.465349000
5	1.339255000	1.203211000	-0.322627000
5	1.826897000	0.900092000	1.340011000
5	1.808099000	-0.867214000	1.545601000
5	1.294275000	-1.603810000	-0.003239000
5	0.981173000	-0.294074000	-1.167498000
17	5.464995000	-0.280988000	-1.002179000
17	3.868184000	2.884512000	0.139718000
17	4.560059000	0.143961000	2.558284000
17	3.880793000	-3.059960000	0.853628000
17	2.822772000	-2.317164000	-2.600856000
17	2.806496000	1.379563000	-3.016982000
17	0.452700000	2.771410000	-0.730573000
17	1.271763000	1.995403000	2.625749000
17	1.241844000	-1.558989000	3.070814000
17	0.257387000	-3.036774000	0.006653000
17	-0.403829000	-0.365436000	-2.278143000
14	-1.768124000	2.953365000	-0.177381000
6	-2.126565000	1.733434000	1.162795000
1	-3.043656000	2.092356000	1.660638000
1	-2.357201000	0.737513000	0.757371000
1	-1.329873000	1.703768000	1.920738000
6	-2.554891000	2.703378000	-1.827910000
1	-2.383507000	1.680149000	-2.189825000
1	-3.642447000	2.843458000	-1.723796000
1	-2.172419000	3.426637000	-2.562957000
6	-1.692494000	4.709375000	0.416378000
1	-1.320298000	5.394228000	-0.359826000
1	-2.711589000	5.030966000	0.689860000
1	-1.052697000	4.796595000	1.307261000
16	-4.381149000	-0.307249000	-0.836082000
14	-4.038488000	-2.348173000	-0.137406000
14	-6.043261000	0.471461000	0.308474000
6	-5.985643000	2.331811000	0.022394000
6	-7.670791000	-0.186913000	-0.348710000
1	-7.765328000	-1.270965000	-0.193545000
1	-8.515028000	0.306238000	0.160435000
1	-7.755494000	0.010152000	-1.428164000
6	-5.874104000	0.115778000	2.145756000
1	-4.912687000	0.478396000	2.539715000
1	-6.683601000	0.622510000	2.696138000
1	-5.946034000	-0.959800000	2.364800000
6	-3.072781000	-3.152502000	-1.519515000
1	-2.127976000	-2.624131000	-1.711887000
1	-2.826141000	-4.192045000	-1.249317000
1	-3.663247000	-3.166191000	-2.448167000
6	-5.666350000	-3.249221000	0.118410000
1	-6.260556000	-2.833952000	0.945699000
1	-6.276816000	-3.224915000	-0.796750000
1	-5.455178000	-4.304174000	0.358839000
6	-3.061742000	-2.307680000	1.463868000
1	-2.072555000	-1.850609000	1.318034000

1	-3.592826000	-1.758536000	2.254708000
1	-2.886044000	-3.335549000	1.821176000
1	-6.844659000	2.816632000	0.513640000
1	-5.068876000	2.780814000	0.434078000
1	-6.037218000	2.565342000	-1.052365000

Dicat_CB(+)

14	3.934013000	0.220014000	-1.669046000
14	2.309740000	3.626527000	0.047243000
16	3.806061000	1.790203000	-0.093022000
6	5.512871000	0.559183000	-2.570825000
1	5.372701000	1.424506000	-3.239536000
1	5.777174000	-0.309112000	-3.196028000
1	6.345205000	0.772363000	-1.886523000
6	2.498051000	-0.133875000	-2.765906000
1	1.658378000	-0.661634000	-2.297372000
1	2.881600000	-0.710031000	-3.624085000
1	2.105013000	0.822715000	-3.148977000
6	3.600090000	4.966972000	0.059748000
1	4.283019000	4.883701000	0.917891000
1	3.079055000	5.936844000	0.141380000
1	4.192301000	4.985491000	-0.866951000
6	1.508213000	3.409687000	1.699763000
1	0.847693000	2.535522000	1.766829000
1	0.874198000	4.299279000	1.858686000
1	2.260149000	3.399832000	2.503880000
6	1.336223000	3.594626000	-1.521646000
1	1.999410000	3.667884000	-2.397163000
1	0.701912000	4.498303000	-1.513250000
1	0.662826000	2.733528000	-1.617398000
14	3.986233000	0.256641000	1.504700000
14	3.473007000	-3.386115000	0.066582000
16	4.444664000	-1.258717000	-0.067688000
6	2.550618000	-0.085092000	2.600549000
1	2.885235000	-0.786823000	3.381349000
1	2.260593000	0.853968000	3.098164000
1	1.663305000	-0.478350000	2.089500000
6	5.571129000	0.610091000	2.389377000
1	6.400971000	0.797002000	1.694276000
1	5.441179000	1.496052000	3.032550000
1	5.833392000	-0.240184000	3.039493000
6	2.103628000	-3.423398000	-1.162707000
1	2.451446000	-3.250235000	-2.191181000
1	1.659501000	-4.433556000	-1.129542000
1	1.296503000	-2.719870000	-0.915848000
6	4.966147000	-4.395366000	-0.381960000
1	5.798793000	-4.242065000	0.320077000
1	4.682984000	-5.461313000	-0.342924000
1	5.317579000	-4.184335000	-1.402911000
6	2.988074000	-3.510167000	1.850914000
1	2.118456000	-2.882197000	2.088088000
1	2.688709000	-4.558460000	2.025523000
1	3.821229000	-3.293087000	2.535815000
6	-4.161060000	-0.457187000	0.100637000
1	-5.231704000	-0.664211000	0.160014000
5	-1.047154000	0.142380000	-0.071924000
5	-1.785703000	-1.090607000	-1.106759000
5	-2.127803000	0.646194000	-1.383672000
5	-2.201080000	1.458481000	0.214786000
5	-1.890720000	0.223717000	1.481678000
5	-1.635470000	-1.353039000	0.662294000
5	-3.133360000	-1.790579000	-0.183980000
5	-3.442738000	-0.555012000	-1.446668000
5	-3.698955000	1.017365000	-0.632015000
5	-3.551099000	0.756814000	1.136142000
5	-3.200502000	-0.978309000	1.414357000
17	0.728144000	0.455103000	-0.139856000

17	-0.841851000	-2.029647000	-2.298013000
17	-1.505793000	1.485822000	-2.828849000
17	-1.701247000	3.161025000	0.412631000
17	-1.026440000	0.638797000	2.988383000
17	-0.512414000	-2.577388000	1.334688000
17	-3.758480000	-3.429204000	-0.372136000
17	-4.374990000	-0.975804000	-2.882130000
17	-4.888864000	2.156226000	-1.258843000
17	-4.593250000	1.637958000	2.251682000
17	-3.892657000	-1.815482000	2.803383000

CB_dicat_CB

14	0.049254000	1.025015000	1.568529000
14	1.979226000	4.206879000	0.035776000
16	0.270423000	2.622765000	-0.004145000
6	-1.590443000	1.301633000	2.360950000
1	-1.426965000	2.023525000	3.179199000
1	-1.983049000	0.370971000	2.800273000
1	-2.358552000	1.692343000	1.682280000
6	1.407067000	0.791359000	2.793221000
1	2.421785000	0.795646000	2.376684000
1	1.239336000	-0.196342000	3.256390000
1	1.316401000	1.550065000	3.584414000
6	0.890041000	5.719368000	0.079353000
1	0.238859000	5.785836000	-0.804756000
1	1.534393000	6.614709000	0.094237000
1	0.258347000	5.743608000	0.979995000
6	2.904059000	4.059276000	-1.564610000
1	3.473051000	3.125597000	-1.659806000
1	3.631131000	4.889623000	-1.578650000
1	2.236515000	4.193002000	-2.428830000
6	2.911385000	3.985394000	1.622062000
1	2.235851000	3.971528000	2.490310000
1	3.558285000	4.873706000	1.724581000
1	3.559115000	3.100025000	1.634158000
14	0.068584000	1.081648000	-1.631478000
14	-0.708977000	-2.630541000	-0.005080000
16	0.212181000	-0.479366000	-0.055606000
6	1.441639000	0.872413000	-2.843811000
1	1.077608000	0.148734000	-3.593199000
1	1.625002000	1.823687000	-3.367492000
1	2.381356000	0.505371000	-2.416081000
6	-1.546385000	1.424796000	-2.455515000
1	-2.311859000	1.843594000	-1.791539000
1	-1.329140000	2.141015000	-3.266213000
1	-1.967416000	0.512293000	-2.906107000
6	0.864740000	-3.611224000	0.031670000
1	1.498403000	-3.358494000	0.893742000
1	0.596766000	-4.679214000	0.099889000
1	1.471881000	-3.464420000	-0.872210000
6	-1.615481000	-2.689584000	1.610304000
1	-2.484625000	-2.020866000	1.656138000
1	-1.990320000	-3.720164000	1.732239000
1	-0.929044000	-2.489869000	2.447966000
6	-1.618267000	-2.816816000	-1.608904000
1	-0.960337000	-2.575293000	-2.458365000
1	-1.882300000	-3.884895000	-1.697680000
1	-2.547698000	-2.236496000	-1.668448000
6	-8.081617000	-0.217235000	0.095407000
1	-9.171195000	-0.247312000	0.153116000
5	-4.907374000	-0.125276000	-0.073209000
5	-5.759339000	-1.673588000	0.083165000
5	-5.862878000	-0.728044000	-1.440019000
5	-5.889887000	1.015555000	-1.010966000
5	-5.798754000	1.148674000	0.777997000
5	-5.716196000	-0.513290000	1.454606000
5	-7.225722000	-1.355863000	1.034034000
5	-7.316740000	-1.488214000	-0.749916000
5	-7.398178000	0.167675000	-1.423188000
5	-7.358909000	1.321653000	-0.055495000
5	-7.250433000	0.381040000	1.462807000

17	-3.118370000	-0.091175000	-0.158957000
17	-4.893937000	-3.234095000	0.148304000
17	-5.078948000	-1.301855000	-2.936968000
17	-5.157709000	2.250241000	-2.070866000
17	-4.976300000	2.525950000	1.560157000
17	-4.794105000	-0.868197000	2.943203000
17	-8.067164000	-2.530418000	2.052664000
17	-8.249115000	-2.795358000	-1.488650000
17	-8.413976000	0.499436000	-2.830801000
17	-8.334896000	2.794451000	-0.111089000
17	-8.118270000	0.922648000	2.904754000
6	7.635560000	-1.297779000	0.012017000
1	8.603223000	-1.802796000	0.020375000
5	4.824979000	0.167762000	-0.011198000
5	5.043269000	-1.356436000	-0.882545000
5	5.044094000	-1.326119000	0.913188000
5	5.835495000	0.199307000	1.439714000
5	6.323907000	1.112462000	-0.028576000
5	5.834336000	0.149419000	-1.464136000
5	6.733135000	-1.383411000	-1.434391000
5	6.245803000	-2.293725000	0.030465000
5	6.734929000	-1.333209000	1.461773000
5	7.522502000	0.168788000	0.882578000
5	7.521870000	0.138126000	-0.908869000
17	3.245952000	1.006927000	-0.029127000
17	3.721731000	-2.101503000	-1.827508000
17	3.730301000	-2.049733000	1.885101000
17	5.309017000	1.046765000	2.918473000
17	6.300319000	2.895082000	-0.056596000
17	5.305150000	0.943718000	-2.970834000
17	7.338205000	-2.251426000	-2.849085000
17	6.372176000	-4.055734000	0.062514000
17	7.342604000	-2.147738000	2.907045000
17	8.908106000	0.832318000	1.754576000
17	8.906698000	0.769627000	-1.804605000

CB_dicat_CB iso1

14	0.150916000	1.111905000	1.637128000
14	1.561455000	4.467772000	-0.000312000
16	0.097055000	2.677543000	0.021321000
6	-1.456948000	1.186027000	2.542822000
1	-1.275260000	1.812708000	3.431909000
1	-1.767881000	0.187778000	2.887484000
1	-2.287778000	1.604483000	1.961930000
6	1.563827000	1.158802000	2.819767000
1	2.562712000	1.165378000	2.373288000
1	1.475129000	0.255026000	3.446120000
1	1.434943000	2.031818000	3.478925000
6	0.290134000	5.829423000	0.004407000
1	-0.361312000	5.781759000	-0.880963000
1	0.809114000	6.802584000	-0.006537000
1	-0.343947000	5.792561000	0.902801000
6	2.522606000	4.398206000	-1.587172000
1	3.266513000	3.590071000	-1.607653000
1	3.064088000	5.357389000	-1.660833000
1	1.865382000	4.327862000	-2.466192000
6	2.553444000	4.415297000	1.568050000
1	1.913696000	4.347893000	2.460081000
1	3.090878000	5.377931000	1.624140000
1	3.301834000	3.611123000	1.580300000
14	0.130488000	1.089572000	-1.575156000
14	-0.484070000	-2.560107000	0.051921000
16	0.475745000	-0.410869000	0.040466000
6	1.525139000	1.120040000	-2.779927000
1	1.415350000	0.215393000	-3.401786000
1	1.390356000	1.991856000	-3.439557000
1	2.532764000	1.120069000	-2.353939000
6	-1.487585000	1.158765000	-2.460503000
1	-2.305169000	1.601359000	-1.878735000
1	-1.311113000	1.762647000	-3.366251000
1	-1.816965000	0.157421000	-2.777044000
6	1.014851000	-3.650093000	0.077638000
1	1.608893000	-3.535315000	0.994066000
1	0.643684000	-4.688974000	0.030254000
1	1.685102000	-3.480490000	-0.775767000
6	-1.415377000	-2.660964000	1.652590000
1	-2.309029000	-2.026957000	1.700725000
1	-1.750242000	-3.707943000	1.750683000
1	-0.745905000	-2.454770000	2.502402000
6	-1.374168000	-2.666374000	-1.571873000
1	-0.681241000	-2.473147000	-2.405853000
1	-1.719287000	-3.709727000	-1.671608000
1	-2.260263000	-2.023989000	-1.647005000
6	-7.916149000	-0.316349000	-0.045916000
1	-9.005820000	-0.374773000	-0.068061000
5	-4.745450000	-0.142506000	0.020114000
5	-5.559411000	-1.717197000	-0.021985000
5	-5.585586000	-0.638061000	-1.459378000
5	-5.691834000	1.058568000	-0.877332000
5	-5.726932000	1.028530000	0.919395000
5	-5.643676000	-0.686533000	1.448218000
5	-7.096011000	-1.518590000	0.846568000
5	-7.060557000	-1.489048000	-0.943973000
5	-7.142373000	0.219556000	-1.471154000
5	-7.228842000	1.245465000	-0.005909000
5	-7.199801000	0.171955000	1.426459000

17	-2.956724000	-0.055587000	0.056791000
17	-4.657122000	-3.257402000	-0.028782000
17	-4.693548000	-1.058363000	-2.947750000
17	-4.927308000	2.404140000	-1.766128000
17	-4.993416000	2.339512000	1.882352000
17	-4.809366000	-1.154788000	2.954541000
17	-7.973649000	-2.799327000	1.690849000
17	-7.902694000	-2.740505000	-1.864903000
17	-8.066271000	0.656897000	-2.913046000
17	-8.238854000	2.696295000	-0.002852000
17	-8.181592000	0.562194000	2.843317000
6	7.327408000	-1.557619000	-0.014360000
1	8.198875000	-2.214902000	-0.017469000
5	4.790166000	0.358491000	-0.005322000
5	4.758804000	-1.174804000	-0.894242000
5	4.771958000	-1.165182000	0.899808000
5	5.803072000	0.201934000	1.440789000
5	6.430145000	1.038830000	-0.021460000
5	5.780888000	0.186456000	-1.465063000
5	6.418431000	-1.474372000	-1.455218000
5	5.792805000	-2.306255000	0.001189000
5	6.439998000	-1.459064000	1.438980000
5	7.460758000	-0.101737000	0.872574000
5	7.447164000	-0.111280000	-0.918765000
17	3.388044000	1.473803000	0.000941000
17	3.350670000	-1.721213000	-1.855693000
17	3.377104000	-1.697989000	1.886478000
17	5.425842000	1.098884000	2.934336000
17	6.704713000	2.796115000	-0.032843000
17	5.381551000	1.067753000	-2.962149000
17	6.859534000	-2.415880000	-2.883605000
17	5.601588000	-4.064246000	0.011416000
17	6.902377000	-2.385553000	2.870488000
17	8.939910000	0.310072000	1.745026000
17	8.912881000	0.291328000	-1.817793000

Cb_dicat_cb iso2

14	-0.000141000	0.000339000	1.146626000
14	-0.586566000	-3.452483000	-0.350641000
16	-1.084815000	-1.173041000	-0.412110000
6	1.196496000	-1.003198000	2.110687000
1	0.655044000	-1.552917000	2.896597000
1	1.919584000	-0.329474000	2.603990000
1	1.780418000	-1.712773000	1.510383000
6	-1.197039000	1.004017000	2.110092000
1	-1.784534000	1.710365000	1.509376000
1	-0.655388000	1.557346000	2.893328000
1	-1.917195000	0.329697000	2.606848000
6	1.200061000	-3.750619000	-0.746968000
1	1.429162000	-3.603915000	-1.810080000
1	1.377681000	-4.817820000	-0.522895000
1	1.928612000	-3.184873000	-0.149769000
6	-1.733812000	-4.045640000	-1.680158000
1	-2.767585000	-3.708488000	-1.519201000
1	-1.719747000	-5.148810000	-1.677217000
1	-1.397171000	-3.712546000	-2.673656000
6	-1.016863000	-3.864104000	1.409665000
1	-0.177438000	-3.651031000	2.086772000
1	-1.210305000	-4.949627000	1.453773000
1	-1.927097000	-3.357679000	1.761189000
14	-0.000092000	0.000624000	-1.970010000
14	0.587676000	3.453346000	-0.350601000
16	1.085373000	1.173569000	-0.412112000
6	-1.191943000	0.993945000	-2.950760000
1	-0.642564000	1.762052000	-3.518626000
1	-1.683555000	0.317644000	-3.668921000
1	-1.986776000	1.461541000	-2.354624000
6	1.189727000	-0.991715000	-2.954400000
1	1.991232000	-1.453291000	-2.362430000
1	0.639661000	-1.764437000	-3.515342000
1	1.672338000	-0.316052000	-3.679236000
6	-1.199148000	3.752796000	-0.745560000
1	-1.927525000	3.183980000	-0.151039000
1	-1.377054000	4.818801000	-0.516094000
1	-1.428160000	3.611289000	-1.809426000
6	1.018402000	3.864438000	1.409755000
1	1.927261000	3.356084000	1.762038000
1	1.214290000	4.949527000	1.453814000
1	0.177982000	3.653510000	2.086314000
6	1.734579000	4.046026000	-1.680549000
1	1.399031000	3.710569000	-2.673622000
1	1.718619000	5.149177000	-1.679598000
1	2.768780000	3.710711000	-1.518519000
6	4.407774000	-0.087939000	0.185866000
1	3.320739000	-0.097846000	0.209273000
5	7.633354000	-0.026668000	0.091137000
5	6.747649000	-0.688504000	1.511775000
5	6.719955000	-1.562975000	-0.062335000
5	6.657955000	-0.337324000	-1.386621000
5	6.649774000	1.293661000	-0.630245000
5	6.707824000	1.077022000	1.160413000
5	5.224536000	0.222092000	1.647591000
5	5.231930000	-1.390892000	0.900580000
5	5.185013000	-1.178007000	-0.863114000
5	5.131475000	0.561954000	-1.207476000
5	5.172866000	1.421008000	0.342346000

17	9.402739000	0.006399000	0.037986000
17	7.549569000	-1.349164000	2.947443000
17	7.485383000	-3.146798000	-0.280614000
17	7.359017000	-0.638778000	-2.987088000
17	7.337318000	2.718212000	-1.431883000
17	7.458246000	2.276729000	2.227930000
17	4.300851000	0.531097000	3.141937000
17	4.308112000	-2.726924000	1.638267000
17	4.261119000	-2.334980000	-1.855850000
17	4.092234000	1.187545000	-2.521996000
17	4.231654000	2.919102000	0.563821000
6	-4.407757000	0.087690000	0.185180000
1	-3.320670000	0.097161000	0.208001000
5	-7.633428000	0.026215000	0.091889000
5	-6.650072000	-1.294145000	-0.629743000
5	-6.707336000	-1.077284000	1.160882000
5	-6.747087000	0.688268000	1.511955000
5	-6.720181000	1.562558000	-0.062200000
5	-6.658692000	0.336766000	-1.386356000
5	-5.132159000	-0.562373000	-1.207783000
5	-5.172682000	-1.421334000	0.342162000
5	-5.223903000	-0.222205000	1.647258000
5	-5.231599000	1.390672000	0.899959000
5	-5.185549000	1.177667000	-0.863621000
17	-9.402829000	-0.007022000	0.039538000
17	-7.337989000	-2.718755000	-1.430953000
17	-7.457195000	-2.276891000	2.228909000
17	-7.548376000	1.349137000	2.947899000
17	-7.485749000	3.146334000	-0.280256000
17	-7.360436000	0.637750000	-2.986611000
17	-4.093701000	-1.188401000	-2.522748000
17	-4.231063000	-2.919189000	0.563330000
17	-4.299880000	-0.531069000	3.141425000
17	-4.307842000	2.727061000	1.637079000
17	-4.262355000	2.334669000	-1.856887000

Cb_dicat_cb iso3

14	-0.336588000	0.502946000	1.305490000
14	1.644776000	3.885878000	0.333242000
16	0.002680000	2.286606000	-0.001573000
6	-1.561660000	0.941783000	2.608553000
1	-1.003793000	1.412240000	3.435620000
1	-2.031840000	0.019113000	2.984748000
1	-2.365103000	1.602761000	2.264139000
6	1.132719000	-0.354706000	2.012504000
1	1.753238000	-0.909582000	1.300853000
1	0.749168000	-1.036395000	2.788856000
1	1.789076000	0.378013000	2.507783000
6	0.541370000	5.370015000	0.557099000
1	-0.053784000	5.579695000	-0.344129000
1	1.173678000	6.251633000	0.757397000
1	-0.144999000	5.245463000	1.407636000
6	2.631168000	3.985870000	-1.233598000
1	3.229615000	3.088521000	-1.434314000
1	3.332859000	4.828942000	-1.113382000
1	1.983796000	4.216313000	-2.093665000
6	2.457898000	3.364293000	1.913516000
1	1.718909000	3.283537000	2.725805000
1	3.173812000	4.155065000	2.194132000
1	3.030561000	2.431951000	1.823696000
14	-0.401348000	1.095877000	-1.839616000
14	-0.446046000	-2.663054000	-0.773379000
16	-1.215619000	-0.487722000	-0.500185000
6	1.023595000	0.661192000	-2.931040000
1	0.611956000	0.110614000	-3.793095000
1	1.445941000	1.604702000	-3.314893000
1	1.836069000	0.089764000	-2.471711000
6	-1.740324000	1.939500000	-2.786492000
1	-2.602349000	2.208633000	-2.163246000
1	-1.312397000	2.846254000	-3.245790000
1	-2.092054000	1.276903000	-3.592500000
6	1.165027000	-2.597848000	-1.684894000
1	1.925583000	-1.989907000	-1.175913000
1	1.561752000	-3.627097000	-1.716957000
1	1.054583000	-2.255467000	-2.722823000
6	-0.278888000	-3.316047000	0.960185000
1	-1.139648000	-3.076705000	1.600190000
1	-0.246324000	-4.415087000	0.863626000
1	0.656280000	-3.000779000	1.440635000
6	-1.835164000	-3.444682000	-1.716901000
1	-1.969736000	-3.007278000	-2.715780000
1	-1.580099000	-4.511059000	-1.843033000
1	-2.790660000	-3.376221000	-1.176585000
6	-6.799458000	-0.640899000	-1.452388000
1	-7.098858000	-0.950941000	-2.454824000
5	-5.910678000	0.275506000	1.509544000
5	-7.640466000	0.087767000	1.073706000
5	-6.581073000	-1.359484000	1.198289000
5	-4.964408000	-0.911571000	0.568331000
5	-5.021444000	0.800039000	0.053204000
5	-6.672039000	1.425816000	0.365313000
5	-7.796735000	0.497545000	-0.656600000
5	-7.740084000	-1.213986000	-0.145632000
5	-6.091579000	-1.823887000	-0.452133000
5	-5.134546000	-0.494628000	-1.149550000
5	-6.184737000	0.937468000	-1.277036000

17	-5.365332000	0.780364000	3.127833000
17	-8.956082000	0.387316000	2.222000000
17	-6.766774000	-2.577595000	2.473722000
17	-3.530951000	-1.742657000	1.271816000
17	-3.633698000	1.938081000	0.123134000
17	-6.950279000	3.131646000	0.765976000
17	-9.239594000	1.139642000	-1.447466000
17	-9.126222000	-2.271515000	-0.427432000
17	-5.813579000	-3.478386000	-1.027881000
17	-3.976276000	-0.823429000	-2.470904000
17	-5.991053000	2.020743000	-2.667289000
6	7.059981000	-0.716719000	-1.401372000
1	7.576724000	-0.949923000	-2.333862000
5	5.554104000	-0.037764000	1.368505000
5	4.760416000	0.119151000	-0.209798000
5	5.148597000	-1.497995000	0.414900000
5	6.726896000	-1.385114000	1.260930000
5	7.291609000	0.325182000	1.147441000
5	6.056894000	1.253327000	0.234162000
5	5.965250000	0.585567000	-1.410873000
5	5.412028000	-1.105500000	-1.300586000
5	6.631801000	-2.038739000	-0.399745000
5	7.949135000	-0.916173000	0.046610000
5	7.537931000	0.706391000	-0.580935000
17	4.676348000	0.357790000	2.874092000
17	3.059614000	0.667074000	-0.368510000
17	3.862114000	-2.643200000	0.901811000
17	7.123833000	-2.423090000	2.641603000
17	8.280415000	1.076771000	2.411685000
17	5.725992000	2.982963000	0.530587000
17	5.602760000	1.531222000	-2.868435000
17	4.524900000	-1.821061000	-2.664581000
17	6.988788000	-3.701150000	-0.880253000
17	9.623693000	-1.473048000	0.007071000
17	8.794444000	1.758935000	-1.239290000

Cb_dicat_CB iso4

14	0.000000000	0.000000000	1.562064000
14	-3.456775000	0.612624000	0.000000000
16	-1.169526000	1.082637000	0.000000000
6	-1.002359000	-1.190599000	2.533112000
1	-1.701602000	-0.638089000	3.180747000
1	-0.319772000	-1.773284000	3.173721000
1	-1.555530000	-1.918866000	1.926033000
6	1.002359000	1.190599000	2.533112000
1	1.555530000	1.918866000	1.926033000
1	1.701602000	0.638089000	3.180747000
1	0.319772000	1.773284000	3.173721000
6	-3.769665000	-1.213231000	0.000000000
1	-3.401159000	-1.741192000	-0.888556000
1	-4.869346000	-1.321819000	0.000000000
1	-3.401159000	-1.741192000	0.888556000
6	-3.932014000	1.432402000	-1.596317000
1	-3.472598000	2.423893000	-1.718008000
1	-5.027111000	1.566073000	-1.600113000
1	-3.675799000	0.794442000	-2.455386000
6	-3.932014000	1.432402000	1.596317000
1	-3.675799000	0.794442000	2.455386000
1	-5.027111000	1.566073000	1.600113000
1	-3.472598000	2.423893000	1.718008000
14	0.000000000	0.000000000	-1.562064000
14	3.456775000	-0.612624000	0.000000000
16	1.169526000	-1.082637000	0.000000000
6	1.002359000	1.190599000	-2.533112000
1	1.701602000	0.638089000	-3.180747000
1	0.319772000	1.773284000	-3.173721000
1	1.555530000	1.918866000	-1.926033000
6	-1.002359000	-1.190599000	-2.533112000
1	-1.555530000	-1.918866000	-1.926033000
1	-1.701602000	-0.638089000	-3.180747000
1	-0.319772000	-1.773284000	-3.173721000
6	3.769665000	1.213231000	0.000000000
1	3.401159000	1.741192000	0.888556000
1	4.869346000	1.321819000	0.000000000
1	3.401159000	1.741192000	-0.888556000
6	3.932014000	-1.432402000	1.596317000
1	3.472598000	-2.423893000	1.718008000
1	5.027111000	-1.566073000	1.600113000
1	3.675799000	-0.794442000	2.455386000
6	3.932014000	-1.432402000	-1.596317000
1	3.675799000	-0.794442000	-2.455386000
1	5.027111000	-1.566073000	-1.600113000
1	3.472598000	-2.423893000	-1.718008000
6	-0.046062000	-4.465600000	0.000000000
1	-0.017451000	-3.377947000	0.000000000
5	-0.074225000	-7.693439000	0.000000000
5	-0.536258000	-6.749839000	1.461181000
5	-1.593673000	-6.742519000	0.000000000
5	-0.536258000	-6.749839000	-1.461181000
5	1.172603000	-6.770185000	-0.902946000
5	1.172603000	-6.770185000	0.902946000
5	0.417508000	-5.245769000	1.436997000
5	-1.272457000	-5.238050000	0.888618000
5	-1.272457000	-5.238050000	-0.888618000
5	0.417508000	-5.245769000	-1.436997000
5	1.454168000	-5.272043000	0.000000000

17	-0.093016000	-9.463868000	0.000000000
17	-1.043444000	-7.489657000	2.990213000
17	-3.210488000	-7.469458000	0.000000000
17	-1.043444000	-7.489657000	-2.990213000
17	2.471549000	-7.523481000	-1.845310000
17	2.471549000	-7.523481000	1.845310000
17	0.923966000	-4.262608000	2.840126000
17	-2.505622000	-4.298589000	1.772558000
17	-2.505622000	-4.298589000	-1.772558000
17	0.923966000	-4.262608000	-2.840126000
17	2.995197000	-4.378134000	0.000000000
6	0.046062000	4.465600000	0.000000000
1	0.017451000	3.377947000	0.000000000
5	0.074225000	7.693439000	0.000000000
5	-1.172603000	6.770185000	-0.902946000
5	-1.172603000	6.770185000	0.902946000
5	0.536258000	6.749839000	1.461181000
5	1.593673000	6.742519000	0.000000000
5	0.536258000	6.749839000	-1.461181000
5	-0.417508000	5.245769000	-1.436997000
5	-1.454168000	5.272043000	0.000000000
5	-0.417508000	5.245769000	1.436997000
5	1.272457000	5.238050000	0.888618000
5	1.272457000	5.238050000	-0.888618000
17	0.093016000	9.463868000	0.000000000
17	-2.471549000	7.523481000	-1.845310000
17	-2.471549000	7.523481000	1.845310000
17	1.043444000	7.489657000	2.990213000
17	3.210488000	7.469458000	0.000000000
17	1.043444000	7.489657000	-2.990213000
17	-0.923966000	4.262608000	-2.840126000
17	-2.995197000	4.378134000	0.000000000
17	-0.923966000	4.262608000	2.840126000
17	2.505622000	4.298589000	1.772558000
17	2.505622000	4.298589000	-1.772558000

Cb_dicat_cb iso5

14	-0.520634000	2.082079000	0.533205000
14	2.788614000	4.159659000	1.431844000
16	0.929421000	3.756747000	0.098620000
6	-2.034911000	2.936083000	1.122107000
1	-1.783730000	3.485784000	2.044512000
1	-2.813451000	2.194575000	1.360852000
1	-2.441006000	3.636738000	0.380927000
6	0.133373000	0.723128000	1.583341000
1	1.099444000	0.310637000	1.267380000
1	-0.630928000	-0.069572000	1.596548000
1	0.222382000	1.109950000	2.610775000
6	2.319052000	5.855180000	2.048377000
1	2.225003000	6.582154000	1.228294000
1	3.116002000	6.209803000	2.724221000
1	1.378246000	5.842108000	2.618381000
6	4.224583000	4.214262000	0.267486000
1	4.400985000	3.267068000	-0.257441000
1	5.129292000	4.413748000	0.865692000
1	4.107534000	5.037146000	-0.454247000
6	2.682817000	2.894199000	2.778791000
1	1.725732000	2.980881000	3.315434000
1	3.487950000	3.118539000	3.498126000
1	2.840231000	1.864540000	2.433991000
14	0.924893000	3.212587000	-2.052631000
14	-0.430227000	-0.538281000	-2.447236000
16	-0.624382000	1.683372000	-1.660091000
6	2.504008000	2.748176000	-2.884330000
1	2.280207000	2.607381000	-3.954473000
1	3.193857000	3.603886000	-2.798753000
1	3.007202000	1.857193000	-2.492164000
6	0.044685000	4.605519000	-2.901688000
1	-0.885988000	4.873052000	-2.382303000
1	0.697125000	5.492408000	-2.944979000
1	-0.202917000	4.311626000	-3.934615000
6	1.252485000	-0.590761000	-3.210208000
1	2.047411000	-0.404480000	-2.477002000
1	1.411191000	-1.617533000	-3.582349000
1	1.360517000	0.091087000	-4.065829000
6	-0.621200000	-1.550584000	-0.917552000
1	-1.531975000	-1.308181000	-0.351314000
1	-0.727724000	-2.598810000	-1.246179000
1	0.277184000	-1.491654000	-0.288299000
6	-1.809163000	-0.553511000	-3.681911000
1	-1.629318000	0.166066000	-4.494563000
1	-1.842995000	-1.564346000	-4.123516000
1	-2.786688000	-0.357964000	-3.220790000
6	-7.746426000	-0.516389000	-0.542786000
1	-8.720106000	-0.497661000	-1.035138000
5	-4.888177000	-0.578164000	0.914901000
5	-5.934203000	-2.025377000	0.889584000
5	-5.165077000	-1.439613000	-0.621738000
5	-5.151579000	0.348031000	-0.577247000
5	-5.914118000	0.882177000	0.949008000
5	-6.400064000	-0.584536000	1.864895000
5	-7.618688000	-1.444938000	0.889936000
5	-6.858019000	-1.969630000	-0.636331000
5	-6.366832000	-0.509666000	-1.540453000
5	-6.830241000	0.918941000	-0.575261000
5	-7.604116000	0.340430000	0.927231000

17	-3.276781000	-0.590091000	1.713628000
17	-5.460671000	-3.562033000	1.639983000
17	-3.907185000	-2.388188000	-1.463334000
17	-3.843207000	1.300158000	-1.357397000
17	-5.432372000	2.389174000	1.775676000
17	-6.417079000	-0.611802000	3.639274000
17	-9.011118000	-2.325672000	1.527896000
17	-7.481420000	-3.372181000	-1.513817000
17	-6.471725000	-0.462583000	-3.308585000
17	-7.415784000	2.380720000	-1.384774000
17	-8.973387000	1.229519000	1.605254000
6	7.067428000	-2.116170000	0.453676000
1	7.920291000	-2.781403000	0.600951000
5	4.577375000	-0.179798000	0.027312000
5	5.296792000	-1.045244000	-1.347120000
5	4.382802000	-1.938100000	-0.082377000
5	4.718290000	-1.152087000	1.500827000
5	5.844675000	0.217940000	1.208835000
5	6.191371000	0.287755000	-0.549670000
5	7.028928000	-1.218804000	-0.997480000
5	5.911714000	-2.587071000	-0.710787000
5	5.556430000	-2.654939000	1.044021000
5	6.456415000	-1.327353000	1.837672000
5	7.364825000	-0.439901000	0.574573000
17	3.131842000	0.875349000	-0.193105000
17	4.701360000	-0.860923000	-3.017513000
17	2.808674000	-2.700463000	-0.432347000
17	3.477596000	-1.068177000	2.779067000
17	5.834478000	1.693549000	2.215881000
17	6.480930000	1.832557000	-1.393764000
17	8.307713000	-1.359617000	-2.207503000
17	6.088058000	-4.080625000	-1.636877000
17	5.382790000	-4.214596000	1.852208000
17	7.172862000	-1.569636000	3.433631000
17	8.976101000	0.193425000	0.925656000

Cb_dicat_cb iso6

14	0.040597000	0.887525000	1.472562000
14	1.775461000	4.188079000	-0.080775000
16	0.191766000	2.483215000	-0.108342000
6	-1.640691000	1.074896000	2.191745000
1	-1.569447000	1.830430000	2.992384000
1	-1.992976000	0.133991000	2.641264000
1	-2.395835000	1.398713000	1.461502000
6	1.402654000	0.763436000	2.709011000
1	2.363555000	0.465345000	2.283434000
1	1.082650000	0.004196000	3.443159000
1	1.510313000	1.717718000	3.242839000
6	0.569805000	5.609434000	-0.068517000
1	-0.068730000	5.611444000	-0.959361000
1	1.137808000	6.553344000	-0.042971000
1	-0.080172000	5.594037000	0.823817000
6	2.730922000	4.110718000	-1.668241000
1	3.386489000	3.238158000	-1.743445000
1	3.344606000	5.018959000	-1.705041000
1	2.053300000	4.144113000	-2.548486000
6	2.711129000	4.057791000	1.517012000
1	2.037522000	4.087411000	2.371191000
1	3.360940000	4.948866000	1.561674000
1	3.369125000	3.177252000	1.572881000
14	0.043493000	0.912024000	-1.706577000
14	-0.383986000	-2.868447000	-0.152475000
16	0.302047000	-0.626666000	-0.129697000
6	1.402023000	0.791594000	-2.946742000
1	1.068032000	0.058876000	-3.701520000
1	1.534903000	1.757858000	-3.457816000
1	2.363914000	0.463576000	-2.527993000
6	-1.632586000	1.121314000	-2.436042000
1	-2.388983000	1.425576000	-1.705680000
1	-1.549272000	1.898572000	-3.214681000
1	-1.980355000	0.192960000	-2.914801000
6	1.275789000	-3.692757000	-0.160595000
1	1.880995000	-3.409639000	0.713316000
1	1.115141000	-4.783917000	-0.140646000
1	1.861463000	-3.443517000	-1.055715000
6	-1.305114000	-3.105881000	1.439483000
1	-2.291774000	-2.623632000	1.450529000
1	-1.458586000	-4.192952000	1.556073000
1	-0.705528000	-2.773586000	2.300954000
6	-1.295132000	-3.071312000	-1.756215000
1	-0.695315000	-2.704136000	-2.605002000
1	-1.427259000	-4.157651000	-1.907106000
1	-2.288524000	-2.605052000	-1.758507000
6	-7.335145000	-0.502630000	-1.420087000
1	-7.865771000	-0.702788000	-2.352418000
5	-5.791360000	0.094812000	1.344401000
5	-7.431347000	-0.612443000	1.334938000
5	-6.030574000	-1.578717000	0.752994000
5	-4.946248000	-0.466721000	-0.116106000
5	-5.622463000	1.173816000	-0.074332000
5	-7.174319000	1.100483000	0.822343000
5	-8.271955000	0.035505000	-0.097587000
5	-7.570903000	-1.611232000	-0.136890000
5	-6.029958000	-1.520678000	-1.026532000
5	-5.784547000	0.170668000	-1.534485000
5	-7.163240000	1.139169000	-0.960489000

17	-4.922499000	0.411596000	2.870288000
17	-8.302925000	-1.026078000	2.822672000
17	-5.383616000	-2.993719000	1.622067000
17	-3.169186000	-0.708020000	-0.152630000
17	-4.532321000	2.592147000	-0.064335000
17	-7.774437000	2.473591000	1.772488000
17	-10.018165000	0.250572000	-0.256823000
17	-8.617642000	-3.021068000	-0.337324000
17	-5.496396000	-2.835316000	-2.093996000
17	-5.033061000	0.511779000	-3.109588000
17	-7.798236000	2.441397000	-1.974488000
6	7.471164000	0.416185000	-0.826803000
1	8.258355000	0.927214000	-1.383745000
5	5.167639000	-1.118435000	0.826517000
5	6.442262000	-2.027482000	-0.035555000
5	6.849011000	-1.052616000	1.430337000
5	5.848730000	0.438165000	1.395025000
5	4.859588000	0.372506000	-0.077929000
5	5.198874000	-1.127757000	-0.963436000
5	6.900558000	-1.064412000	-1.471214000
5	7.914015000	-1.014782000	-0.000933000
5	7.550214000	0.497351000	0.882644000
5	6.308437000	1.376512000	-0.043523000
5	5.909487000	0.414746000	-1.491165000
17	3.865971000	-1.909388000	1.766737000
17	6.518036000	-3.799023000	-0.043700000
17	7.349484000	-1.809580000	2.952777000
17	5.240461000	1.258947000	2.858136000
17	3.240776000	1.124230000	-0.131380000
17	3.943167000	-1.906728000	-1.970582000
17	7.544159000	-1.708145000	-2.985432000
17	9.572076000	-1.617677000	-0.061367000
17	8.837852000	1.394539000	1.692459000
17	6.299740000	3.150125000	-0.144146000
17	5.500413000	1.246168000	-3.007062000

7 References

(1) M. Lehmann, A. Schulz, A. Villinger, *Angew. Chem. Int. Ed.* **2009**, *48*, 7444–7447.

(2) M. Lehmann, A. Schulz, A. Villinger, *Angew. Chem.* **2009**, *121*, 7580–7583.

(3) T. J. Curphey, *Phosphorus Sulfur Silicon Relat. Elem.* **2001**, *173*, 123–142.

(4) C. A. Reed, *Acc. Chem. Res.* **2010**, *43*, 121–128.

(5) G. B. Dunks, K. Palmer-Ordonez, *Inorg. Chem.* **1978**, *17*, 1514–1516.

(6) G. B. Dunks, K. Barker, E. Hedaya, C. Hefner, K. Palmer-Ordonez, P. Remec, *Inorg. Chem.* **1981**, *20*, 1692–1697.

(7) A. Franken, B. T. King, J. Rudolph, P. Rao, B. C. Noll, J. Michl, *Collect. Czechoslov. Chem. Commun.* **2001**, *66*, 1238–1249.

(8) Z. Xie, T. Jelínek, R. Bau, C. A. Reed, *J. Am. Chem. Soc.* **1994**, *116*, 1907–1913.

(9) A. Hepp, R. Labbow, F. Reiß, A. Schulz, A. Villinger, *Eur. J. Inorg. Chem.* **2018**, *2018*, 2905–2914.

(10) G. R. Fulmer, A. J. M. Miller, N. H. Sherden, H. E. Gottlieb, A. Nudelman, B. M. Stoltz, J. E. Bercaw, K. I. Goldberg, *Organometallics* **2010**, *29*, 2176–2179.

(11) G. M. Sheldrick, in *SHELXS-2013 - Program for the Solution of Crystal Structures*, University of Göttingen, Germany, **2013**.

(12) G. M. Sheldrick, in *SHELXL-2013 - Program for the Refinement of Crystal Structures*, University of Göttingen, Germany, **2013**.

(13) G. M. Sheldrick, in *SADABS Version 2*, University of Göttingen, Germany, **2004**.

(14) M. J. Frisch, G. W. Trucks, H. B. Schlegel, G. E. Scuseria, M. A. Robb, J. R. Cheeseman, G. Scalmani, V. Barone, B. Mennucci, G. A. Peterson, H. Nakatsuji, M. Caricato, X. Li, H. P. Hratchian, A. F. Izmaylov, J. Bloino, G. Zheng, J. L. Sonnenberg, M. Hada, J. C. and D. J. F. Gaussian 09, Revision C.01. Gaussian, Inc., Wallingford CT 2010.

(15) Weinhold, F.; Landis, C. R.; Glendening, E. D. What Is NBO Analysis and How Is It Useful? *Int. Rev. Phys. Chem.* **2016**, *35* (3), 399–440. https://doi.org/10.1080/0144235X.2016.1192262.

(16) Glendening, E. D.; Weinhold, F. Natural Resonance Theory: I. General Formalism. *J. Comput. Chem.* **1998**, *19* (6), 593–609. https://doi.org/10.1002/(SICI)1096-987X(19980430)19:6<593::AID-JCC3>3.3.CO;2-X.

(17) Glendening, E. D.; Badenhoop, J. K.; Weinhold, F. Applications, Natural

Resonance Theory: III. *J. Comp. Chem.* **1998**, *19*, 628–646.

(18) Zhang, L.; Ying, F.; Wu, W.; Hiberty, P. C.; Shaik, S. Topology of Electron Charge Density for Chemical Bonds from Valence Bond Theory: A Probe of Bonding Types. *Chem. - A Eur. J.* **2009**, *15* (12), 2979–2989. https://doi.org/10.1002/chem.200802134.

(19) Glendening, E. D.; Weinhold, F. Natural Resonance Theory: II. Natural Bond Order and Valency. *J. Comput. Chem.* **1997**, *19* (6), 610–627.

(20) L. Suidan, J. K. Badenhoop, E. D. Glendening, F. W. Common Textbook and Teaching Misrepresentations of Lewis Structures.

(21) Weinhold, F. Chemistry. A New Twist on Molecular Shape. *Nature* **2001**, *411* (6837), 539–541. https://doi.org/10.1038/35079225.

(22) Reed, A. E.; Curtiss, L. a.; Weinhold, F. Intermolecular Interactions from a Natural Bond Orbital, Donor-Acceptor Viewpoint. *Chem. Rev.* **1988**, *88* (6), 899–926. https://doi.org/10.1021/cr00088a005.

(23) Glendening, E. D.; Landis, C. R.; Weinhold, F. NBO 6.0: Natural Bond Orbital Analysis Program. *J. Comput. Chem.* **2013**, *34* (16), 1429–1437. https://doi.org/10.1002/jcc.23266.

(24) S. Grimme, S. Ehrlich, J. G. No Title. *J. Chem. Phys.* **1999**, *110*, 6158−6170.

(25) Goerigk, L.; Grimme, S. A Thorough Benchmark of Density Functional Methods for General Main Group Thermochemistry, Kinetics, and Noncovalent Interactions. *Phys. Chem. Chem. Phys.* **2011**, *13* (14), 6670. https://doi.org/10.1039/c0cp02984j.

(26) Liu, S. Steric Effect: A Quantitative Description from Density Functional Theory. *J. Chem. Phys.* **2007**, *126* (24). https://doi.org/10.1063/1.2747247.

(27) Lu, T.; Chen, F. Multiwfn: A Multifunctional Wavefunction Analyzer. *J. Comput. Chem.* **2012**, *33* (5), 580–592. https://doi.org/10.1002/jcc.22885.
